# Parkinson's disease motor symptoms rescue by CRISPRa‐reprogramming astrocytes into GABAergic neurons

**DOI:** 10.15252/emmm.202114797

**Published:** 2022-04-04

**Authors:** Jessica Giehrl‐Schwab, Florian Giesert, Benedict Rauser, Chu Lan Lao, Sina Hembach, Sandrine Lefort, Ignacio L Ibarra, Christina Koupourtidou, Malte Daniel Luecken, Dong‐Jiunn Jeffery Truong, Judith Fischer‐Sternjak, Giacomo Masserdotti, Nilima Prakash, Jovica Ninkovic, Sabine M Hölter, Daniela M Vogt Weisenhorn, Fabian J Theis, Magdalena Götz, Wolfgang Wurst

**Affiliations:** ^1^ Institute of Developmental Genetics Helmholtz Center Munich Neuherberg Germany; ^2^ Munich School of Life Sciences Weihenstephan Technical University of Munich Munich Germany; ^3^ Institute of Stem Cell Research Helmholtz Center Munich Neuherberg Germany; ^4^ Physiological Genomics Biomedical Center (BMC) Ludwig‐Maximilians‐Universität (LMU) Planegg‐Martinsried Germany; ^5^ Institute for Diabetes and Obesity Helmholtz Center Munich Neuherberg Germany; ^6^ Institute of Computational Biology Helmholtz Center Munich Neuherberg Germany; ^7^ Department for Cell Biology and Anatomy Biomedical Center Ludwig‐Maximilians‐Universität (LMU) Planegg‐Martinsried Germany; ^8^ Laboratory of Applied Genetics and Stem Cell Biology Department Hamm 2 Hamm‐Lippstadt University of Applied Sciences Hamm Germany; ^9^ German Mouse Clinic Helmholtz Center Munich Neuherberg Germany; ^10^ Department of Mathematics Technical University of Munich Garching Germany; ^11^ Munich Cluster for Systems Neurology (SyNergy) Munich Germany; ^12^ German Center for Neurodegenerative Diseases (DZNE) Site Munich Munich Germany

**Keywords:** astrocytes, CRISPRa, GABAergic neurons, Parkinson's disease, reprogramming, Biotechnology & Synthetic Biology, Genetics, Gene Therapy & Genetic Disease, Neuroscience

## Abstract

Direct reprogramming based on genetic factors resembles a promising strategy to replace lost cells in degenerative diseases such as Parkinson's disease. For this, we developed a knock‐in mouse line carrying a dual dCas9 transactivator system (dCAM) allowing the conditional *in vivo* activation of endogenous genes. To enable a translational application, we additionally established an AAV‐based strategy carrying intein‐split‐dCas9 in combination with activators (AAV‐dCAS). Both approaches were successful in reprogramming striatal astrocytes into induced GABAergic neurons confirmed by single‐cell transcriptome analysis of reprogrammed neurons *in vivo*. These GABAergic neurons functionally integrate into striatal circuits, alleviating voluntary motor behavior aspects in a 6‐OHDA Parkinson's disease model. Our results suggest a novel intervention strategy beyond the restoration of dopamine levels. Thus, the AAV‐dCAS approach might enable an alternative route for clinical therapies of Parkinson's disease.

## Graphical abstract

GABAergic neurons generated by CRISPR‐mediated direct reprogramming of striatal astrocytes rescue voluntary motor behavior in a toxin‐induced murine model for Parkinson's disease, suggesting a novel intervention strategy beyond the restoration of dopamine levels.

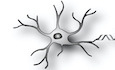

**The paper explained**

**Problem**

One central hallmark of Parkinson's disease, the second most common neurodegenerative disorder, is the degeneration of midbrain dopaminergic neurons projecting to the striatum. Motor symptoms like bradykinesia, rigidity, or gait alterations are currently treated either by pharmacological restoration of dopamine levels, electrophysiological pace‐making of downstream nuclei, or alternatively by replacing the lost neurons. For the latter, direct reprogramming by selective overexpression of single or combinations of transcription factors has successfully converted various somatic cell types into functional neurons.

**Results**

This study describes the direct reprogramming of astrocytes in the striatum of a toxin‐induced Parkinson's disease mouse model. CRISPRa induction of the reprogramming factors ALN in astrocytes of the striatum resulted in the generation of induced GABAergic neurons. For this, two different systems have been used: a novel transgenic mouse line carrying a conditional dCas9 activation system and an AAV system which allows the delivery of the dCas9 activators solely by adeno‐associated virus. With both systems, a significant amelioration of motor impairments could be achieved.

**Impact**

These results demonstrate on the one hand the applicability of an AAV‐mediated CRISPRa induction system which can be translated to various species. On the other hand, the phenotypic rescue presumably mediated by GABAergic neurons suggests that therapeutic approaches should consider the role of GABAergic modulation in the treatment of Parkinson's disease.

## Introduction

Parkinson's disease (PD) is the second most common neurodegenerative disorder, characterized by the degeneration of nigrostriatal dopaminergic neurons in the substantia nigra *pars compacta* (SN*pc*), leading to specific motor symptoms like tremor, bradykinesia, and rigidity (McGregor & Nelson, 2019). Current treatments focus on symptomatic disease management, either by pharmacological restoration of dopamine levels or by electrophysiological pace‐making of downstream nuclei, which initially ameliorates the motor symptoms. Alternative therapy options are aiming to replace lost neurons (Jamebozorgi *et al*, 2019; Bjorklund & Parmar, 2020; Parmar *et al*, 2020). To circumvent the need of external cell source and their associated difficulties, *in vivo* direct reprogramming of cells within the brain like astrocytes or oligodendrocytes into functional neurons has been experimentally explored. Pioneering work has successfully converted various somatic cell types into functional neurons by selective overexpression of single or combinations of transcription factors (TFs) or by knockdown of RNA‐binding protein (Heins *et al*, 2002; Berninger *et al*, 2007; Heinrich *et al*, 2011; Rivetti di Val Cervo *et al*, 2017; Qian *et al*, 2020; Zhou *et al*, 2020). Recent results have shown that *in vivo* reprogrammed neurons can mature and functionally integrate into existing neuronal networks (Torper *et al*, 2015; Mattugini *et al*, 2019; Vignoles *et al*, 2019; Zhou *et al*, 2020). To further develop the TF overexpression approach *in vivo*, more efficient genetic tools to adjustably induce multiple genes and deliver complex gene induction systems *in vivo* are needed. Toward this goal, we adapted a programmable, RNA‐guided CRISPR activation (CRISPRa) system to modulate endogenous gene expression aiming to reprogram astrocytes into neurons *in vivo*. We established a new conditional *Rosa26* knock‐in mouse line carrying a dual transactivator system (VPR and SAM), called dCas9 activator mouse (*dCAM*), for experimental modeling. To enable therapeutic applications independent of a genetically modified recipient, we additionally developed an adeno‐associated virus (AAV)‐based intein‐split‐dCas9 activator system (*AAV‐dCAS*). Expanding the previously applied AAV‐encoded dCas9‐VP64 system (Colasante *et al*, 2020), the intein‐split‐dCas9 in combination with the SAM activator system enables a high level of activation of multiple target genes by overcoming the AAV package size limitation (Truong *et al*, 2015; Moretti *et al*, 2020). This system can be broadly applied as a universal cellular reprogramming tool in any species of interest. Ultimately, with minor modifications, it would be suitable as a potential therapeutic intervention. We used both systems to directly reprogram adult striatal astrocytes into induced neurons *in vivo* in a unilateral 6‐OHDA (6‐hydroxydopamine) toxin‐induced mouse model of PD. We compared two transcription factor combinations *Ascl1*, *Lmx1a*, *Nr4a2* (*ALN*) and *Ascl1*, *Lmx1a*, *NeuroD1*, *miRNA218 (ALNe‐218)* to reprogram glial cells into neurons *in vivo* (Caiazzo *et al*, 2011; Torper *et al*, 2015; Pereira *et al*, 2017; Rivetti di Val Cervo *et al*, 2017). Interestingly, with the *ALN* combination, we obtained striatal GABAergic neurons capable of attenuating toxin‐induced motor behavior deficits.

## Results

### Generation of the conditional Rosa26 knock‐in dCas9 activator mouse (*dCAM*)

**Figure 1:**
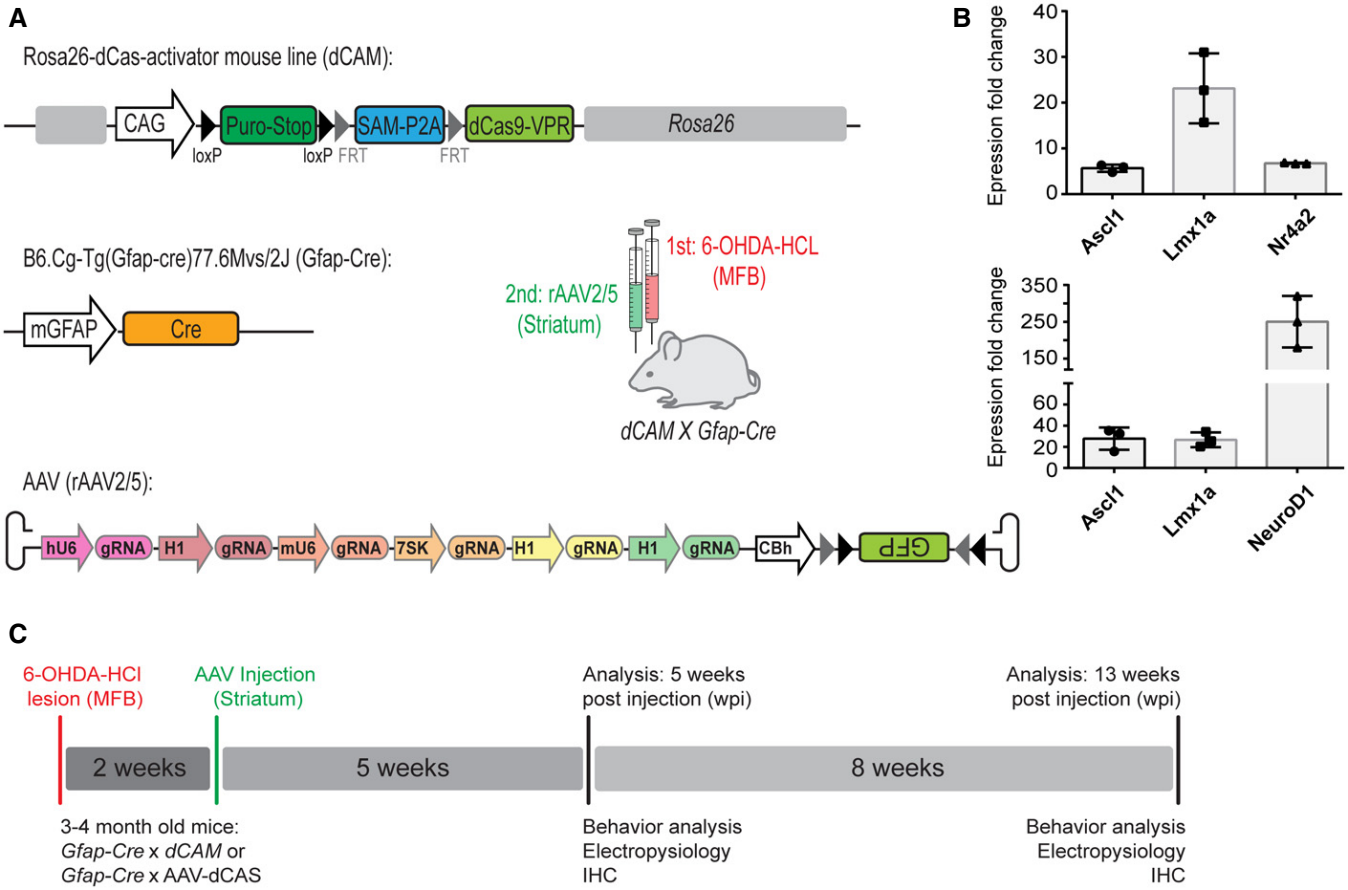
The Rosa26 knock‐in dCas9 activator mouse (*dCAM*) Knock‐in of a conditional dCas9‐VPR‐P2A‐SAM expression cassette into the *Gt(ROSA)26Sor* locus enables flexible multiplexed endogenous gene activation *in vitro* and *in vivo*. The cassette is composed of a ubiquitous CAG promoter, a stop cassette (S*top LoxP*‐puro‐stop‐*LoxP*), followed by the *FRT*‐flanked SAM activator, a P2A peptide and dCas9‐VPR. *dCAM* x *Gfap*‐*Cre* mice enable astrocyte‐specific dCas9 and activator expression. For *in vivo* activation, six sgRNAs driven by different Pol III promoters (H1, hU6, mU6, and 7SK) and the marker gene FLEx‐*GFP* or split‐FLEx‐*GFP* respectively, driven by a CBh promoter are delivered by AAV.Multiplexed activation of *Ascl1*, *Lmx1a*, *NeuroD1* and of *Ascl1*, *Lmx1a*, *Nr4a2* in primary astrocytic cultures. *n* = 3, representative experiment from 2 to 3 independent experiments, additional data in supplement. Activation levels are depicted as fold change between cells transfected with and without sgRNAs. All levels were normalized to β‐Actin. Error bars represent mean ± SD between technical replicates.Experimental Outline: 6‐hydroxydopamine (6‐OHDA‐HCl) is stereotactic injected into the medium forebrain bundle to induce nigrostriatal dopaminergic neurodegeneration. Two weeks later, an AAV expressing gRNAs and a fluorescent reporter is injected into the dorsal striatum. Animals were analyzed after 5 and 13 weeks post injection (wpi) including behavior tests, electrophysiological measurements, and immunohistochemistry. Abbreviations: Puro, puromycin resistance; SAM, synergistic activation mediator (MS2: MS2 bacteriophage coat protein; p65, p65 subunit of human NF‐ĸB; HSF1, Heat shock factor 1); P2A, 2A self‐cleaving peptide; dCas9, deadCas9 (nuclease‐deficient); VPR—VP64, 4× VP16 herpes simplex virus protein vmw65; p65, Rta, Regulator of transcriptional activation; CAG, CMV early enhancer/chicken β actin promoter; CBh, chicken ß–actin hybrid promoter. SgRNA expression is driven by the different Pol III promoters (H1, hU6, mU6, and 7SK). Source data are available online for this figure.

To enable the comprehensive and efficient application of CRISPR/Cas9 activation (CRISPRa) *in vivo*, we generated a novel conditional dCas9‐activator knock‐in mouse line in the safe harbor locus *Gt(ROSA)26Sor* (Fig 1A) by combining two previously described activation strategies: the dCas9‐VPR and the synergistic activation mediator (SAM) system (Chavez *et al*, 2015; Konermann *et al*, 2015). Conditionally controlled by a *LoxP*‐puro‐stop‐*LoxP* cassette, the ubiquitous CAG promoter drives the expression of the *FRT*‐flanked SAM components (aptamere‐fused activator domains of p65 and HSF1) separated via a P2A element from dCas9, C‐terminally coupled to the transcriptional activator domains VP64, p65, and Rta (VPR) (Appendix Fig S1A). Optionally, if a lower level of gene induction is required, the *FRT*‐flanked SAM components can be removed by flippase‐induced recombination, converting the dual dCAM activator mouse into a pure dCas‐VPR line (Appendix Fig S1B). The correct integration of the construct was confirmed via Southern blot analysis; animals of the F1 generation showed a normal Mendelian inheritance (Appendix Fig S1C). Appropriate astrocytic expression of the conditional system and cleavage of the P2A sequence between the SAM activator and the dCas9‐VPR was confirmed by western blot analysis (Appendix Fig S1A). For *in vivo* gene activation, the delivery of target‐specific sgRNAs, including stem loops for SAM‐aptamere binding, is required. For this, AAVs were utilized to deliver sgRNAs driven by individual Pol III promoters and a FLExed‐GFP as a reporter to visualize transduction (Fig 1A, Appendix Fig S17). In all experiments, the rAAV2/5 serotype has been selected due to its known tropism for astrocytes (Ortinski *et al*, 2010; Xie *et al*, 2010). In case more sgRNAs are required, additional AAVs can be used with a split‐FLExed‐*GFP* (Appendix Fig S1D and E) (Foglieni *et al*, 2017). The FLEx system (Cre‐ON) is a reporter system based on an inverted and *LoxP*‐flanked *GFP* gene cassette, which is re‐inverted and expressed in a Cre‐dependent manner, to specifically highlight AAV‐infected target cells (Torper *et al*, 2015). To verify the functionality *in vivo*, *Rosa26‐dCas9‐activator* (*dCAM*) mice were crossed with an astrocyte‐specific Cre line (*Gfap‐Cre*, B6.Cg‐Tg(Gfap‐cre)77.6Mvs/2J, (Gregorian *et al*, 2009)), resulting in cell type‐specific expression of the activator system. Western blot analysis from primary astrocytic lysates confirmed dCas9 expression exclusively in *dCAM* *×* *Gfap‐Cre* double‐positive animals (Appendix Fig S1C). The ability for multiplexed endogenous gene activation was confirmed in primary astrocytes (Fig 1B, Appendix Fig S2A and B). Thus, astrocyte‐specific expression was achieved, on the one hand, by the AAV2/5 serotype to transduce the specific gRNAs and, on the other hand, by Gfap‐Cre specific induction of the activator system.

### *dCAM*‐based reprogramming of astrocytes into induced neurons *in vivo*

**Figure 2:**
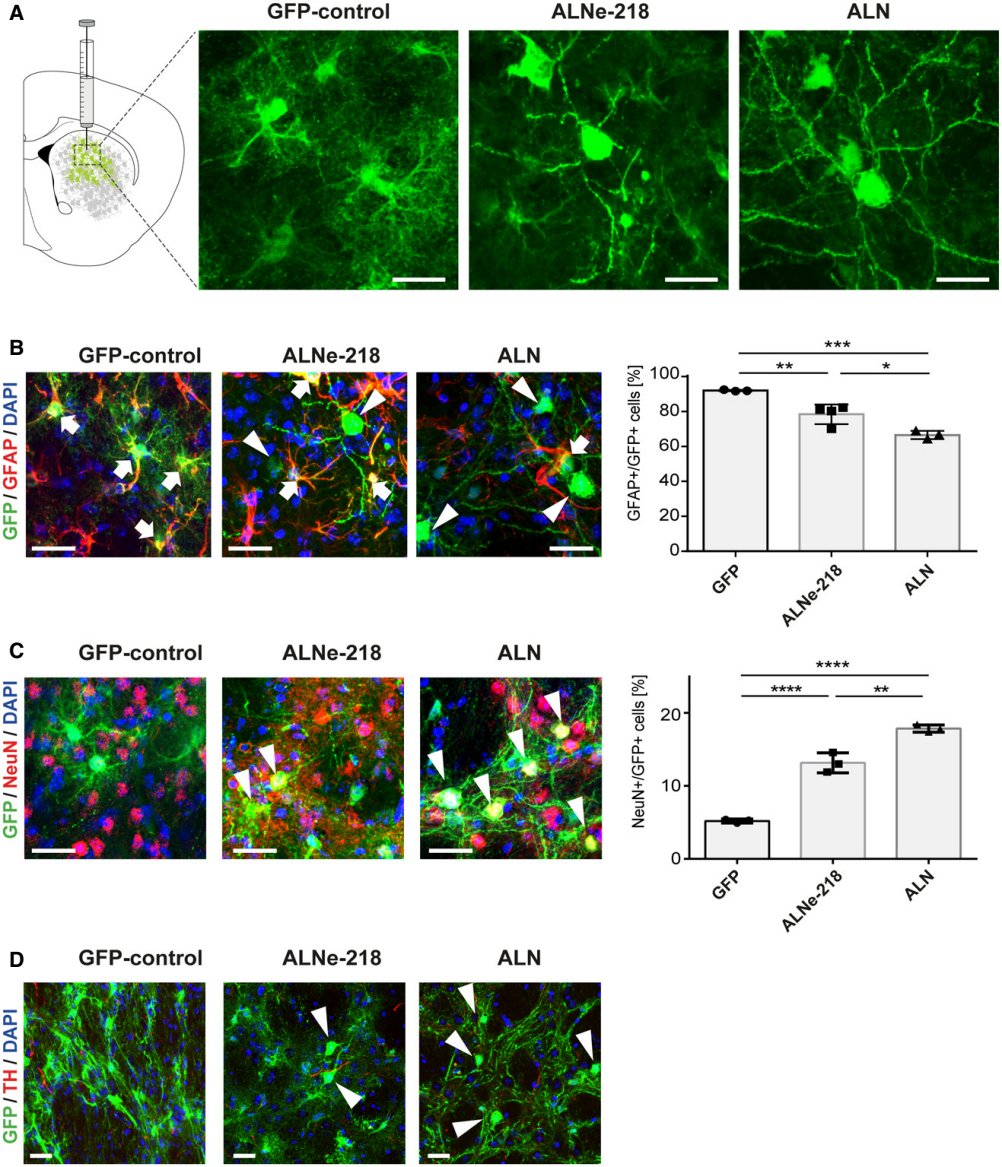
dCas9 activator mouse (*dCAM*) based reprogramming of astrocytes Representative photomicrographs taken from the dorsal striatum 13 weeks after AAV injection. In mice injected with *GFP* control virus, virtually all GFP‐positive cells depict an astrocytic morphology, many GFP‐positive cells in *ALNe‐218* and *ALN*‐treated animals show a neuron‐like morphology.Immunohistochemical analysis showing GFP^+^/GFAP^+^ double‐positive cells 13 wpi. Arrows indicate double‐positive GFP^+^/GFAP^+^ cells, arrowheads indicate GFP^+^/GFAP^−^ cells. Quantification of GFAP+/GFP+ cells shows a significant decrease upon ALNe‐218 and ALN treatment (*GFP* vs *ALNe‐218 P* = 0.0064, *GFP* vs *ALN P* = 0.0002 and *ALN* vs *ALNe‐218 P* = 0.0127). Multiple comparison ANOVA *F*(2,7) = 32.06.Double immunostaining for GFP and the neuronal marker NeuN. Arrowheads indicate double‐positive GFP^+^/NeuN^+^ cell 13 wpi. Quantification demonstrates a significant increase in NeuN^+^/GFP^+^ cells upon ALNe‐218 and ALN‐induction (*GFP* vs *ALNe‐218 P* < 0.0001, *GFP* vs *ALN P* < 0.0001, and *ALN* vs *ALNe‐218 P* = 0.0012). Multiple comparison ANOVA *F*(2,6) = 170.3.Double immunostaining for GFP and TH as a marker for dopaminergic neurons in the dorsal striatum of *GFP control*, *ALNe‐218‐* and *ALN*‐treated animals. Arrowheads indicate GFP^+^/TH^‐^ cells with neuronal morphology. No GFP^+^/TH^+^ cell could be detected in any of the experimental groups. Scale bars indicate 20 µm. Data information: Scale bars indicate 20 µm. Tukey's multiple comparisons test *P < 0.05, **P < 0.01, ***P < 0.001, ****P < 0.0001. *n* = 3–4 mice per condition. Error bars represent mean ± SD. Source data are available online for this figure.

To model advanced stages of PD in mice, the well‐established 6‐hydroxydopamine (6‐OHDA) toxin model was utilized. *dCAM* × *Gfap‐Cre* double transgenic mice were subjected to a unilateral injection of the neurotoxin into the medium forebrain bundle (MFB) at the age of 12–16 weeks, resulting in an efficient and reproducible lesion of the dopaminergic neurons, primarily in the ipsilateral SN*pc* and their projections into the striatum (Appendix Fig S3A) (Gregorian *et al*, 2009). This injury promotes reactive gliosis in the striatum, indicated by the up‐regulation of *Gfap* (Appendix Fig S3B and C) (Grealish *et al*, 2010; Guo *et al*, 2014; Schlachetzki *et al*, 2014). Two weeks after 6‐OHDA injection, sets of sgRNAs, either targeting the promoter regions of the transcription factors *Ascl1*, *Lmx1a*, *Nr4a2* (*ALN*) or targeting *Ascl1*, *Lmx1a*, *NeuroD1* and ectopically expressing miRNA218 (*ALNe‐218*), were delivered via stereotactic injection of high‐titer AAV2/5 into the dorsal striatum. The AAV FLEx‐*GFP* has been used as control for Gfap‐Cre specific induction of the activator system. Cre‐dependency of the construct has been confirmed *in vivo* by injection into Gfap‐Cre negative animals (Appendix Fig S19). The mice were comprehensively analyzed 5 and 13 weeks post injection, respectively (Fig 1C). The initial analysis of transduction efficiency showed comparable amounts of GFP^+^ cells between the experimental groups (Appendix Fig S4). Immunohistochemical (IHC) analysis revealed that in mice injected with FLEx‐*GFP* control virus 5 weeks post injection (wpi) 97.13 ± 0.45% of GFP‐positive cells were astrocytes, indicated by the expression of the astrocytic marker glial fibrillary acidic protein (*Gfap*) (Appendix Fig S5A and B). Similar to previous reports using FLEx‐GFP in combination with this *Cre* line (Mattugini *et al*, 2019), about 4% (3.9 ± 0.53%) were positive for the neuronal marker NeuN (RBFOX3, Appendix Fig S5C and D) in animals injected with the control virus. In contrast to the homogeneous astrocytic morphology of most GFP+ cells in controls, animals injected with the reprogramming AAVs (*ALN* and *ALNe‐218*) showed to a certain extent changes toward a neuronal morphology (Fig 2A). To assess the efficiency of reprogramming achieved by different sgRNA combinations, morphology and marker co‐expression of GFP+ cells were determined and quantified. At 5 weeks post AAV injection, both combinations (*ALN* and *ALNe‐218*) showed a slight but significantly decreased proportion of GFAP^+^/GFP^+^ double‐positive cells (Appendix Fig S5A and B) alongside an increased proportion of NeuN^+^/GFP^+^ cells to 14.77 ± 3.09% for *ALN* and 15.67 ± 0.96% for *ALNe‐218* (Appendix Fig S5C and D) compared to controls. After additional 8 weeks (13 wpi), the proportion of GFAP^+^/GFP^+^ cells decreased to 66.57 ± 2.35% for *ALN* and 78.45 ± 5.63% for *ALNe‐218* (Fig 2B). Conversely, the proportion of NeuN^+^ neurons among GFP^+^ transduced cells further increased to 17.87 ± 0.50% in striata treated with the *ALN*‐inducing sgRNA combination. Interestingly, such marked increase was not observed for the *ALNe‐218* sgRNAs (NeuN^+^/GFP^+^ 13.17 ± 1.36%) (Fig 2C). However, when testing for the expression of the dopaminergic marker tyrosine hydroxylase (TH), no TH^+^/GFP^+^ double‐positive cells could be observed (Fig 2D), indicating that the induced neurons did not acquire dopaminergic fate. Summarizing, in the *dCAM* paradigm, both combinations were able to induce cellular reprogramming, but the *ALN* combination appeared to be more efficient to induce neuronal conversion of striatal astrocytes.

### AAV‐based split‐dCas9‐activator system (*AAV‐dCAS*) for endogenous gene activation

**Figure 3:**
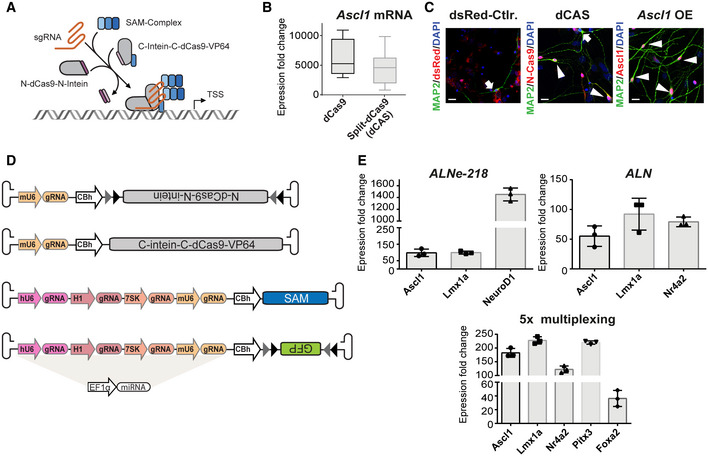
The AAV‐split‐dCas9 activator system (AAV‐dCAS) dCas9 is separated into a N‐ and a C‐terminal part (AAV‐N‐dCas9^aa1–573^‐N‐intein and AAV‐C‐dCas9^aa574–1368^‐VP64‐C‐intein), both portions are fused to the corresponding intein‐moieties. Upon co‐expression, intein‐mediated trans‐splicing leads to reconstitution of Cas9 protein.RT‐qPCR analysis of *Ascl1* induction comparing the activation capacity of full‐length versus split‐dCas9 in Neuro2A cells (data in fold change normalized to non‐activated control; dCas9 6,116 ± 847.3, split‐dCas9 4,415 ± 748.8, *n* = 3 biological replicates, box‐and‐whiskers plots indicate median, 25^th^–75^th^ percentile and min/max whiskers).Immunocytochemistry analysis of reprogrammed primary astrocytes cells 16 days after lentiviral transduction revealed successful *in vitro* reprogramming into neurons using CRISPRa. Astrocytes are infected with two lentiviruses expressing dCas9‐VPR in an intein‐split version similar to the AAV‐dCAS system but driven by a Tet‐O promoter. Tet‐O driven dsRed and Ascl1 cDNA expressing construct were used as negative and positive controls respectively. Arrows indicate single MAP2 positive background neurons, arrowheads indicate double‐positive induced neurons. Scale bar indicates 20 µm.Schematic representation of the AAV‐dCAS system: For induction of up to five endogenous genes plus a GFP reporter, a total of four different AAVs are utilized. dCas9 is delivered by two AAVs, a third AAV is needed for the delivery of the SAM activator. A fourth virus contains the reporter gene, while sgRNAs are distributed between the vectors. To ensure cell type specificity upon Cre expression, the *N‐dCas9* and the *GFP* are inverted and flanked by two different *LoxP* sites (*LoxX* and *Lox511*).Multiplexed activation of *Ascl1*/*Lmx1a*/*NeuroD1*, *Ascl1*/*Lmx1a*/*Nr4a2*, and *Ascl1*/*Lmx1a*/*Nr4a2*/*Pitx3*/*FoxA2* in primary astrocytic cells. *n* = 2–3 biological replicates, one representative run is shown, additional data in supplement. Activation levels are depicted as fold change between cells transfected with and without sgRNAs. All levels were normalized to β‐Actin. Error bars represent mean ± SD between technical replicates. Data information: Abbreviations: dN‐Cas9, N‐terminal dCas9‐residues 1–573; N‐intein, N‐terminal part of DNA polymerase III subunit alpha; dC‐Cas9, C‐terminal dCas9 residues 574–1,368; C‐intein, C‐terminal part of DNA polymerase III subunit alpha; VP64, 4x VP16 herpes simplex virus protein vmw65; p65, p65 subunit of human NF‐ĸB; HSF1, heat shock factor 1; MS2, MS2 bacteriophage coat protein; PAM, protospacer adjacent motif; TSS, transcriptional start site; OE, overexpression; CBh, chicken β‐actin hybrid promoter. SgRNA expression is driven by the different Pol III promoters (H1, hU6, mU6, and 7SK). Source data are available online for this figure.

To enable reprogramming via CRISPRa gene activation independent of transgenic recipients, we generated a universal tool, which allows the efficient and thorough delivering of the complete CRISPRa system via AAVs. To circumvent the low packaging capacity of AAVs, we applied a split‐intein approach to the dCas9‐SAM system suitable for AAV (*AAV‐dCAS*) integration. Since *in vitro* the SAM activator system alone is sufficient to provide robust gene induction (Appendix Fig S6), a split version of the fusion protein dCas9‐VP64 (4× VP16 activator domain, herpes simplex virus protein Vmw65) was generated: each part was fused to corresponding split‐intein moieties (AAV‐N‐dCas9^aa1–573^‐N‐intein and AAV‐C‐dCas9^aa574–1368^‐VP64‐C‐intein). Thus, upon co‐expression of these two AAV constructs, intein‐mediated trans‐splicing leads to the reconstitution of full‐length dCas9‐VP64 protein (Fig 3A). The additional elements of the SAM system were packed onto an independent AAV vector together with four sgRNAs driven by heterologous Pol III promoters (Fig 3D, Appendix Fig S17). The successful reconstitution into full‐length dCas9‐VP64 was confirmed by western blot analysis (Appendix Fig S7A and B). We measured comparable transcriptional activation efficiencies between full‐length and split version of dCas9 when targeting endogenous expression of *Ascl1* in Neuro2A cells (Fig 3B). In primary astrocytic cultures, by activation of endogenous *Ascl1*, cells could be reprogrammed into MAP2^+^ neurons (Fig 3C) using the split dCas9 activation system. Multiplexed gene activation was assessed in different combinations: up to five endogenous genes have been targeted in parallel showing robust activation on RNA and protein level (Fig 3E, Appendix Fig S7C and D).

### *AAV‐dCAS* based reprogramming of astrocytes into induced neurons *in vivo*

**Figure 4:**
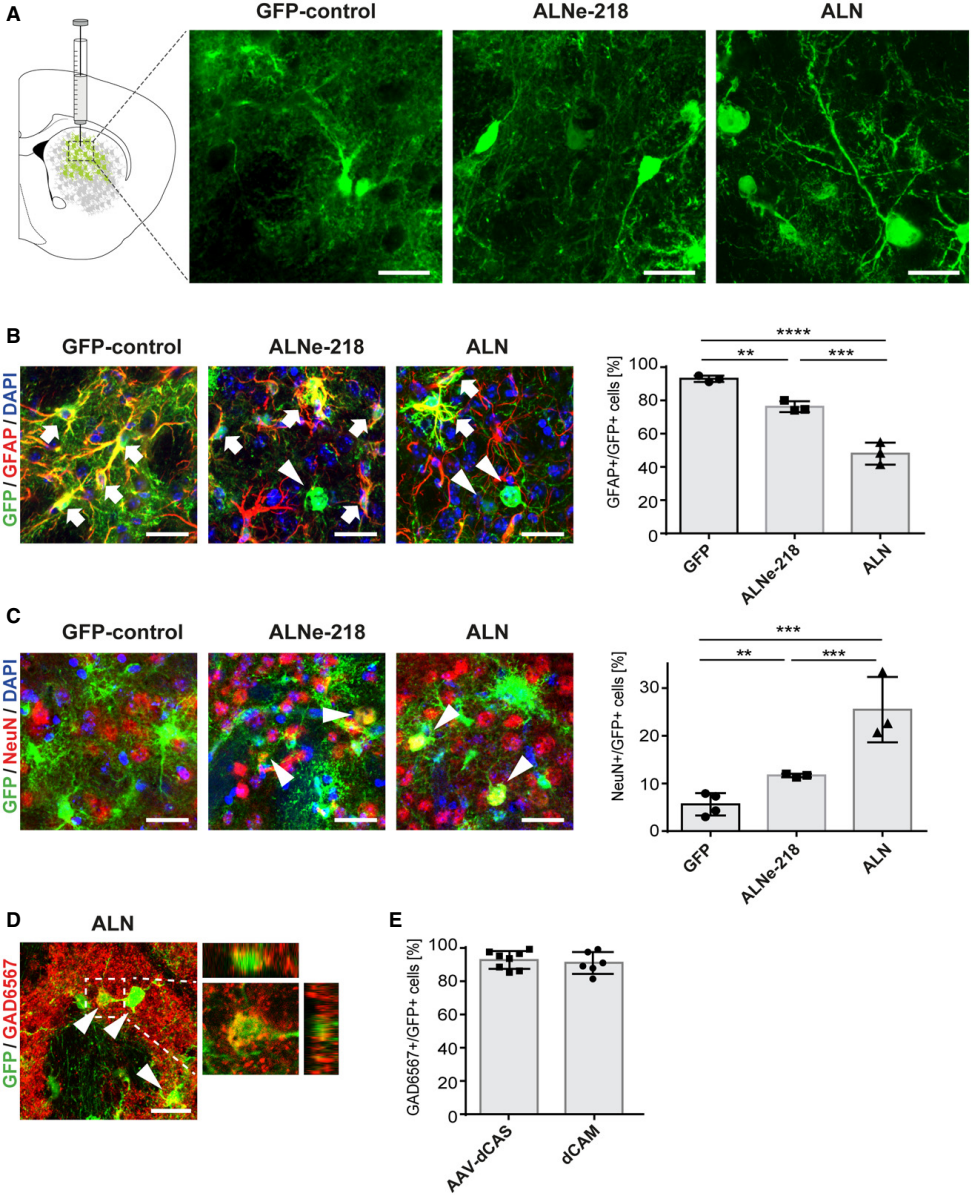
AAV‐split‐dCas9 activator system (AAV‐dCAS) based reprogramming of astrocytes Representative photomicrographs taken from the dorsal striatum 13 weeks after AAV injection. In the *GFP* control condition, virtually all GFP‐positive cells depict an astrocytic morphology, many GFP‐positive cells in *ALNe‐218‐* and *ALN*‐treated animals show a neuron‐like morphology.Immunohistochemical analysis showing GFP^+^/GFAP^+^ double‐positive cells 13 wpi. Arrows indicate double‐positive GFP^+^/GFAP^+^ cells, arrowheads indicate GFP^+^/GFAP^−^ cells. Quantification of GFAP^+^/GFP^+^ cells shows a significant decrease upon *ALNe‐218* and *ALN* treatment (*GFP* vs *ALNe‐218 P* = 0.0083, *GFP* vs *ALN P* < 0.0001, and *ALN* vs *ALNe‐218 P* = 0.0006, multiple comparison ANOVA *F*(2,6) = 79.76).Photomicrographs showing GFP^+^/NeuN^+^ neurons 13 wpi. Arrowheads indicating GFP^+^/NeuN^+^ induced neurons. Quantification demonstrates a significant increase in NeuN^+^/GFP^+^ cells upon *ALNe‐218* and *ALN*‐induction (*GFP* vs *ALN P* = 0.0008, *ALN* vs *ALNe‐218 P* = 0.0092, multiple comparison ANOVA *F*(2,7) = 21.74).In *ALN*‐induced neurons, confocal analysis is demonstrating a co‐localization of GFP and glutamic acid decarboxylase (Gad65/67), a marker specific for GABAergic neurons.Quantification of GAD6567^+^/GFP^+^ cells with neuronal morphology shows that the majority of *ALN*‐induced neurons are colocalizing with this GABAergic marker both in the AAV‐dCAS as well as in the dCAM setting. Data information: Scale bars indicate 20 µm. Error bars represent mean ± SD. *n* = 3–4 mice (B, C) and *n* = 6–8 mice (E) per condition. Tukey's multiple comparisons test **P < 0.01, ***P < 0.001, ****P < 0.0001. Source data are available online for this figure.

Next, we applied the *AAV‐dCAS* system for *in vivo* reprogramming of astrocytes similar to the *dCAM*‐based experiment. Also here, the transgenic *Gfap‐Cre* mouse line was employed to ensure astrocyte‐specific expression of the reprogramming tool. Experimental setup and timeframe were identical to the *dCAM* setting. The Cre‐dependent FLEx‐N‐dCas9 AAV ensures that exclusively GFAP‐positive astrocytes are expressing the complete and active complex. The initial analysis revealed cellular reprogramming from astrocytes into neurons 13 weeks after the injection of the *AAV‐dCAS* system. Identically to the *dCAM* experiment, a fraction of GFP^+^ cells in the *ALNe‐218‐* and *ALN*‐treated animal display neuronal morphology (Fig 4A). A detailed IHC analysis and quantification revealed that 5 weeks post injection (wpi), the proportion of different infected cell types was comparable to the results in the *dCAM* system (Appendix Fig S8A and B). Again, less than 5% (4.23 ± 1.55%) of the GFP^+^ cells were also NeuN positive in GFP control injected mice, whereas this population was markedly increased in both reprogramming conditions, *ALN* (14.67 ± 1.21%) and *ALNe‐218* (14.10 ± 0.89%), to about 14% (Appendix Fig S8C and D). At the later time point (13 wpi), the proportion of GFAP^+^/GFP^+^ cells decreased to 48.0 ± 6.65% for *ALN* and 76.23 ± 3.27% for *ALNe‐218* (Fig 4B), whereas the proportion of NeuN^+^/GFP^+^ reprogrammed cells increased to 25.47 ± 6.85% upon *ALN* activation, but not under the *ALNe‐218* condition (11.67 ± 0.35%; Fig 4C). Thus, the *AAV‐dCAS* approach recapitulates the results obtained with the *dCAM* model, highlighting a higher reprogramming efficiency of the *ALN* combination over time.

### Characterizing the neuronal subtype

To determine the specific neuronal subtype of the induced neurons, a comprehensive IHC analysis was performed including both the transcription factor combinations (*ALNe‐218* and *ALN*) and the reprogramming tools (*dCAM* and *AAV‐dCAS*). Surprisingly, like for the dCAM reprogramming approach, in none of the different conditions and time points, converted neurons were positive for the dopaminergic marker tyrosine hydroxylase (TH) as primarily intended (Appendix Fig S9). Checking for the most abundant neurotransmitter systems of the forebrain revealed that the majority of GFP+ cells were not positive for the glutamatergic marker vGLUT1 but colocalizing for the GABA(gamma‐aminobutyric acid)‐ergic marker Gad65/67 (Fig 4D, Appendix Fig S9). Quantification revealed that this is the case for almost all induced neurons not only in the AAV‐dCAS but also in the dCAM setting (*dCAM: GAD65*/*67^+^*/*GFP^+^* 91.93 ± 6.53%; *AAV‐dCAS: GAD65*/*67^+^*/*GFP* ^+^ 93.60 ± 5.35%; Fig 4E). The vast majority of striatal neurons are GABAergic medium spiny neurons positive for the marker Darpp32 (Dopamine‐ and cAMP‐Regulated Phosphoprotein). Nevertheless, only a minor fraction of GFP+ cells appeared to be Darpp32 positive (*dCAM*: *ALNe‐218* 4.7%, *ALN* 5.7%; *AAV‐dCAS: ALNe‐218* 4%, *ALN* 6.4%), indicating a distinct subtype of the induced neurons (Appendix Fig S10). For further specification, a set of different interneuron subtype markers like parvalbumin, neuropeptide Y, calretinin, and ChAT were tested; nevertheless, none of the markers showed a substantial degree of co‐expression in the induced neurons of both transcription factor combinations (Appendix Fig S11).

### Single‐cell RNA sequencing confirms the GABAergic fate of induced neurons in the *dCAM* model

**Figure 5:**
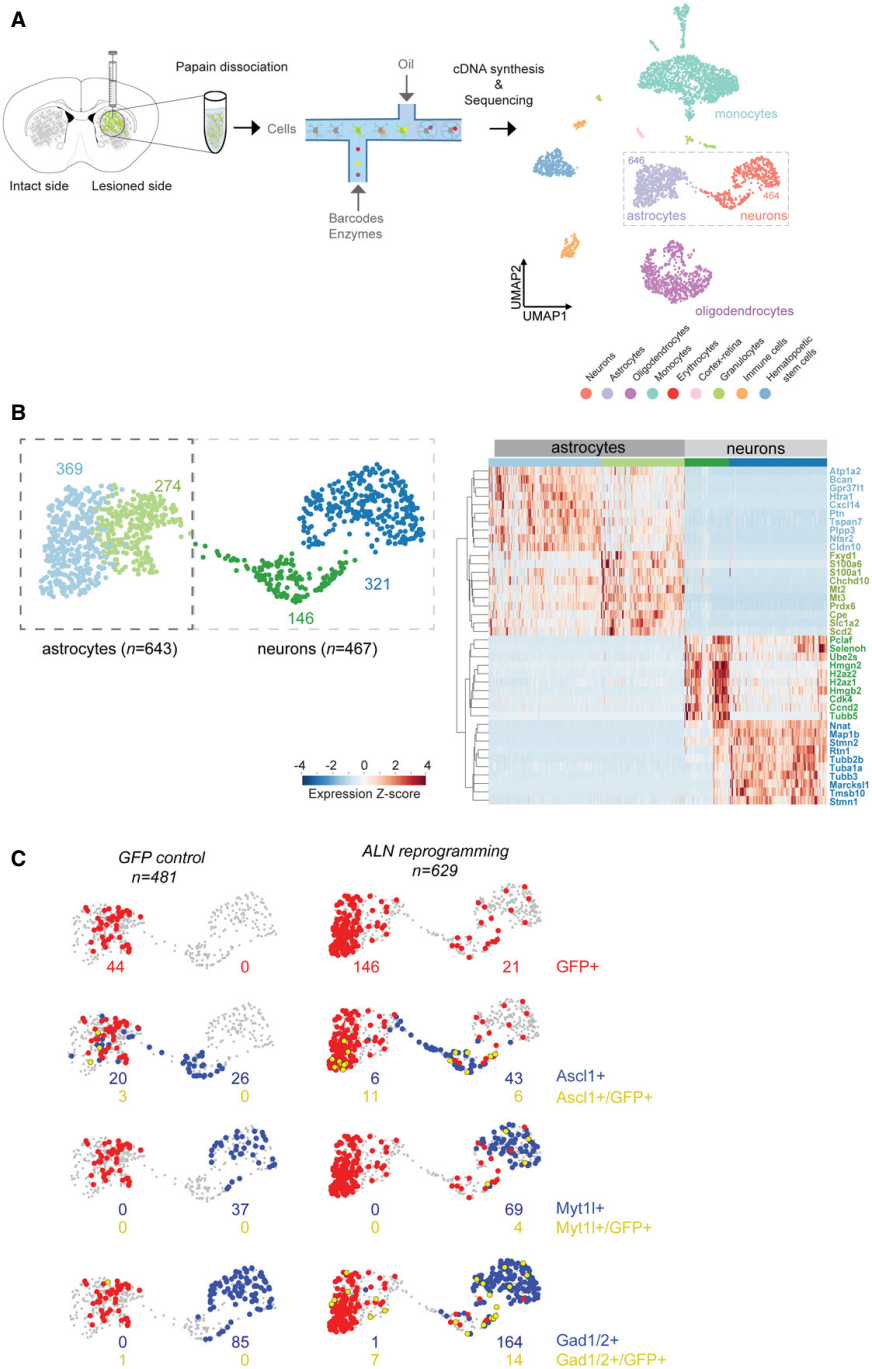
Analysis of striatal tissue from ALN‐reprogrammed dCAM mice by single‐cell RNA‐seq Scheme depicting experimental preparation of cells of 13 wpi mice striatal regions (*n* = 2, one technical replicate). Papain‐dissociated cells are prepared for scRNA‐seq using droplet‐based separation and barcoding. Uniform Manifold Approximation and Projection (UMAP) visualization of QC‐selected cells for *GFP* and *ALN* (*n* = 3,899). Color labeling highlights nine main cell groups based on Leiden clustering and identification based on marker genes. Rectangle highlights astrocytic and neuronal cell clusters. 4,273 highly variable genes (HVG) were detected.Subclustering of 1,110 cells identified four groups of astrocytic and neuronal identity. Layout is based on UMAP visualization presented in (A) Clustering of markers genes selected based on expression levels between clusters. Expression *Z*‐scores are hierarchically clustered by rows.
*GFP control* and *ALN‐reprogrammed* cells selected from the neuronal and astrocytic clusters are visualized based on detection of GFP (red cells), marker gene *Ascl1*, *Myt1l* and *Gad1*/*Gad2* (*Gad1*/*2*) (blue cells), and the co‐detection of both (yellow cells). Numbers of GFP‐positive cells (red), marker gene positive cells (blue), and double‐positive cells (yellow) are indicated for the astrocytic and neuronal clusters respectively.

To get further insights into the molecular characteristics of the induced neurons as well as of their surroundings, we performed single‐cell RNA sequencing (scRNA‐seq) using *GFP* control as well as *ALN‐*reprogrammed animals of the *dCAM* model 13 weeks after virus injection. For this, tissue blocks from the dorsal striatum were dissociated into single cell suspension and further processed for single‐cell library preparation and sequencing. Batch integration of the single cell data using Scanorama (Hie *et al*, 2019), unsupervised clustering, and marker gene annotation of all 3,899 QC‐controlled cells (Appendix Fig S12) revealed grouping into main expected striatal cell types such as oligodendrocytes (*n* = 733), astrocytes (*n* = 646), neurons (*n* = 464), and monocytes (*n* = 1,453; Fig 5A, Appendix Fig S13) (Traag *et al*, 2019). *Cre* expression is, besides a few cells in hematopoietic clusters, restricted to the astrocytic cluster (Appendix Fig S14B) and does not show aberrant activation in the neuronal cluster of the *ALN* condition. Astrocytic and neuronal cells (*n* = 1,110) were further subclustered, uncovering a total of four populations. Selection of marker genes based on cluster‐specific up‐regulation allowed unsupervised separation of neurons and astrocytes into four subclusters and revealed their cell identities (Fig 5B, Appendix Fig S14A). In the GFP control condition, besides a small fraction in the oligodendrocyte and monocyte cluster, the majority of *GFP* ^+^ cells were detected in the astrocyte subclusters. In the *ALN* condition, however, *GFP* ^+^ cells were mapped to neuronal subclusters as well (*n* = 21, Fig 5C). Despite the low number of neurons recovered in scRNA‐seq experiment versus other cell types (11.2%), we detected expression of all endogenously activated genes (*Ascl1*, *Lmx1a*, *Nr4a2*) co‐expressed with GFP in the astrocytic‐neuronal subclusters (Fig 5C, Appendix Fig S14B). The amount of *GFP^+^*/*Ascl1* ^+^ cells is markedly increased from three double‐positive cells in the GFP control to 17 cells in the *ALN* condition. Interestingly, 11 out of the 17 *GFP^+^*/*Ascl1* ^+^ cells are found in one of the astrocytic subclusters. These cells may represent astrocytes with ectopically induced expression of *Ascl1*, either locked in the astrocytic fate or in conversion process. Interestingly, the two neuronal subclusters are characterized among other genes, by high *Ascl1* or *Myt1l* expression (Fig 5C). The analysis for neurotransmitter subtypes revealed no glutamatergic and dopaminergic marker expression in the samples; however, the reprogrammed neurons were positive for *Gad1*/*Gad2* (14 out of 21 GFP^+^ cells in neuronal cluster are Gad1/2^+^) (Fig 5C, Appendix Fig S14C). Overall, our scRNA‐seq revealed that only in the *ALN* condition, GFP‐positive cells are located in the neuronal cluster, where they are equally distributed between the two subclusters. The majority of these cells co‐express *Gad1*/*Gad2* confirming a GABAergic fate.

### Electrophysiological properties of *AAV‐dCAS* induced neurons

**Figure 6:**
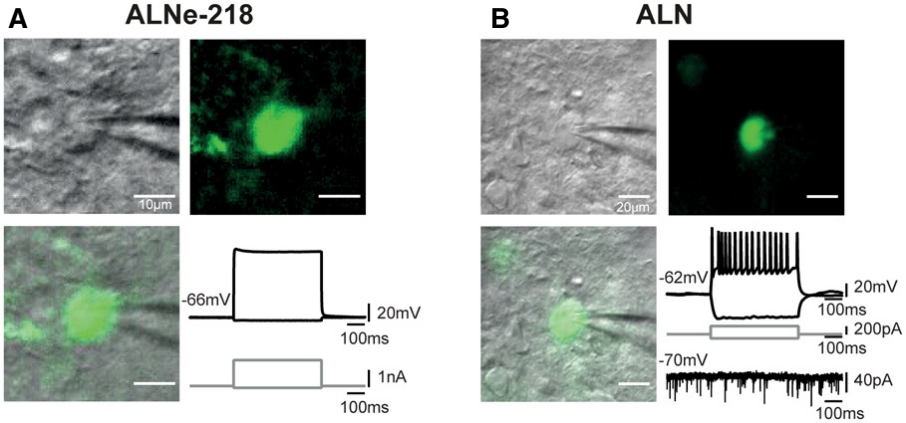
Electrophysiological characterization of *AAV‐dCAS*‐induced neurons Firing pattern of a neuron reprogrammed by the endogenous activation of *Ascl1*, *Lmx1a*, and *NeuroD1* and expression of miRNA218 (*ALNe‐218*). All analyzed cells (*n* = 10) showed electrophysiological properties of immature neuron/glia‐like cells (i.e., lack of APs and a relatively low *R*_in_).Firing pattern of a neuron reprogrammed by the endogenous activation of *Ascl1*, *Lmx1a*, *Nr4a2 (ALN)*. The majority, 14 out of 15 cells, exhibit action potentials, while one showed electrophysiological properties of immature neuron/glia‐like cells (i.e., lack of APs and a relatively low *R*_in_). Bottom right: example of spontaneous synaptic events recorded from an *ALN*‐reprogrammed neuron.

To verify the functionality of the obtained neurons, we investigated the electrophysiological properties of neurons reprogrammed with the *ALNe‐218* and *ALN* combination 13 weeks after initiating of the reprogramming process and found that *ALN*‐induced neurons exhibited mature electrophysiological properties characterized by depolarization‐induced action potentials (APs; Fig 6B, AP threshold = −33.49 ± 2.09 mV; *n* = 14). Further, induced neurons do receive synaptic inputs (Fig 6A, bottom right) indicating, albeit not proving, their integration within the striatal neuronal network. Interestingly, *ALNe‐218*‐induced neurons displayed properties similar to immature neurons (Fig 6B). This includes the inability to produce APs even with a somatic injection of a strong depolarizing current (> 1,500 pA) leading to a resting membrane potential above the normal AP threshold observed with the *ALN* at 13 wpi (Appendix Fig S15A). This is reminiscent of the cellular characteristics observed at 5 wpi under the *ALN* condition (Appendix Fig S15C). No significant difference in the resting membrane potential was observed between these conditions (*ALN*_Vm_ = −64.55 ± 1.53 mV vs *ALNe‐218*_Vm_ = −63.8 ± 2.99 mV; *n* = 15 and *n* = 10, respectively; *P* = 0.85, Kruskal–Wallis test). However, the input resistance (*R*_in_) showed significant differences between the two reprogramming conditions, with an *R*_in_ for *ALN‐*induced neurons of 314.69 ± 41.2 mΩ versus an *R*_in_ for *ALNe‐218* neurons of 105.39 ± 52.27 mΩ (*n* = 15 and *n* = 10, respectively; *P* = 0.002; Kruskal–Wallis test—Appendix Fig S15B). This is indicating that *ALN*‐reprogrammed cells acquire a higher amount or a different composition of ion channels reminiscent of a more mature morphology similar to endogenous neurons (Pereira *et al*, 2017).

### *ALN*‐based reprogramming rescues toxin‐induced motor phenotypes

**Figure 7:**
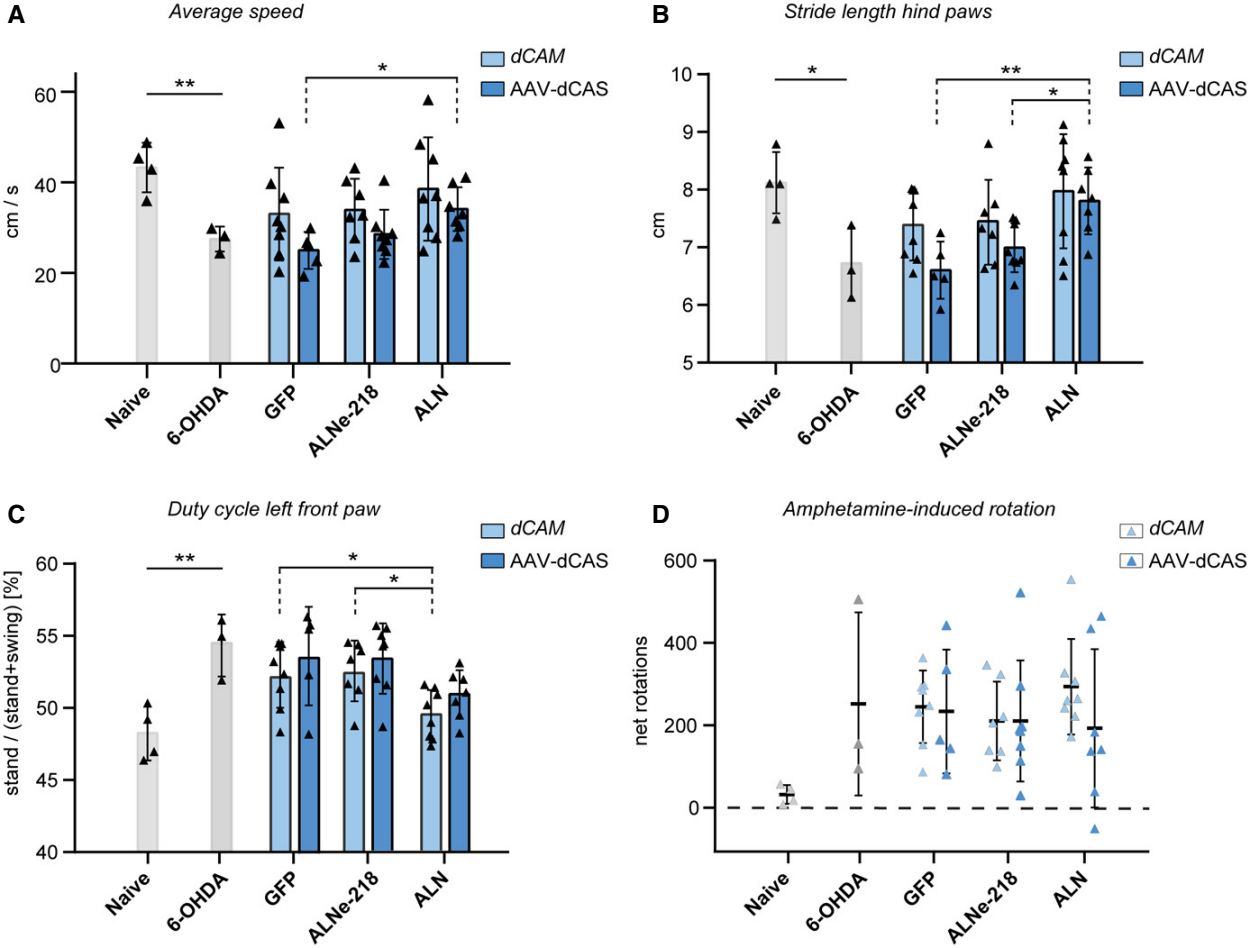
Rescue of motor behavior in dCAM and AAV‐dCAS animals 13 weeks after injection Gait analysis using the CatWalk XT system reveals motor defects in 6‐OHDA lesion model. Both dCAM and AAV‐dCAS animals transduce with AAV containing specific gRNAs show a significant improvement in different aspects of voluntary movement like the average speed (naive versus 6‐OHDA *P* = 0.0063. *AAV‐dCAS*: *GFP* versus *ALN P* = 0.015 multiple comparison ANOVA *F*(2,17) = 12.81).Stride length of hind paws (naive versus 6‐OHDA *P* = 0.0164. *AAV‐dCAS*: *GFP* versus *ALN P* = 0.005, *ALN* versus *ALNe‐218 P* = 0.0042, multiple comparison ANOVA *F*(4,22) = 9.9).Duty cycle of left front paws (naive versus 6‐OHDA *P* = 0.0096. *dCAM*: *GFP* versus *ALN P* = 0.036 and *ALN* versus *ALNe‐218 P* = 0.0252, multiple comparison ANOVA *F*(2,20) = 5.199).In contrast to this, dopamine‐dependent drug‐induced behavior does not show rescue effects: amphetamine‐induced rotation analysis: change in rotational behavior in lesioned animals upon treatment with dopamine releaser substance. Net rotation = ipsilateral rotation‐contralateral rotation (naïve vs 6‐OHDA *P* = 0.09). Data information: Statistics: naive versus 6‐OHDA unpaired t‐test (two‐tailed) *P < 0.05, **P < 0.01. *GFP* versus *ALN*, *GFP* versus *ALNe‐218*, and *ALN* versus *ALNe‐218* Tukey's multiple comparisons test *P < 0.05, **P < 0.01. *n* = 4–8 mice per condition. CatWalk error bars represent mean ± SD. Rotation analysis error bars represent mean ± SEM. Source data are available online for this figure.

To assess whether the newly induced neurons functionally integrate and are capable of ameliorating toxin‐induced phenotypes, we conducted a set of behavioral tests comprising gait analysis, drug‐induced rotation, and the vertical pole test. We examined the behavior comprehensively including both the transcription factor combinations (*ALNe‐218* and *ALN*) and the reprogramming tools (*dCAM* and *AAV‐dCAS*) allowing a comparative evaluation of all applied approaches. Motor behavior was assessed during voluntary movement using the automated CatWalk XT system (Brooks & Dunnett, 2009; Vandeputte *et al*, 2010; Dunnett & Torres, 2012; Glajch *et al*, 2012). At 5 wpi, besides the 6‐OHDA lesion effect between naive and 6‐OHDA lesioned animals, no appreciable differences in spontaneous motor behavior can be observed (Appendix Fig S16A). Eight weeks later, at 13 wpi, the reprogramming combination *ALN* induced a significant rescue, demonstrated by the average speed of the animal and in the stride length of the hind paws (Fig 7A and B). As arm swing is one characteristic parameter altered in PD patients, front paw usage was examined in detail (Mirelman *et al*, 2016). Indeed, a significant improvement in the duty cycle of the front paw can be observed in *ALN*‐treated animals (Fig 7C). Intriguingly, these findings were observed to a similar extent in both systems—using our *dCAM* knock‐in mouse model and the *AAV‐dCAS* setting. Based on these results, the *dCAM* group was additionally analyzed by the vertical pole test, examining striatum‐dependent motor coordination; also here a trend toward improved behavior was observed for the *ALN* combination (Appendix Fig S16B). Importantly, also the coordinated limb usage and axial symmetry, addressed by the phase dispersion between hind paws, converged to naive levels with the *ALN* condition (Appendix Fig S16C). Interestingly, when testing for dopamine receptor‐associated effects of the 6‐OHDA PD model, by the assessment of dopamine‐dependent drug‐induced circling behavior via the amphetamine‐induced behavior paradigm, no rescue in rotation behavior was observed in neither condition nor reprogramming model (Fig 7D). Since this test paradigm is based on the modulation of dopamine receptors, these results confirm that the induced GABAergic neurons reprogrammed by *ALN* leading to a partial rescue of 6‐OHDA motor behavior deficits independent of the dopaminergic system.

## Discussion

Parkinson's disease and the associated disturbance in movement coordination and behavior are provoked mainly by the loss of dopaminergic neurons in the SN*pc*. To date, the prevailing paradigm of disease treatment is the symptomatic management by direct interference of the dopaminergic system. Dopamine levels are restored by drug treatment or through transplantation of dopaminergic neurons (Stoker *et al*, 2017). As an alternative method, we have developed genetic tools to reprogram striatal astrocytes into mature neurons by the CRISPRa‐mediated activation of multiple endogenous transcription factors, such as *Ascl1*, *Lmx1a*, *Nr4a2* (*ALN*) or *Ascl1*, *Lmx1a*, *NeuroD1*, together with miRNA218 (*ALNe‐218*) (Caiazzo *et al*, 2011; Torper *et al*, 2015; Pereira *et al*, 2017; Rivetti di Val Cervo *et al*, 2017). The conventional reprogramming approaches use the ectopic expression of the gene coding sequences (cDNA), making multiplexing of several genes difficult if not impossible, especially when large genes have to be expressed. In contrast, the CRISPRa platform allows multiplexed activation of many endogenous genes by introducing additional sgRNAs to the dCAM or dCAS system. Every gene, regardless of its size or complexity, can be activated with a fixed cargo size comprising a polymerase III promoter and the respective gRNA. Furthermore, by this the endogenous transcriptional machinery can be co‐opted to execute complex genetic splicing patterns (Vierbuchen *et al*, 2010; Pang *et al*, 2011; Torper *et al*, 2015). Here, we describe two distinct approaches based on CRISPR‐mediated gene activation to achieve successful treatment of a murine toxin‐induced PD model. For the *dCAM* mouse line, we followed a Rosa26 knock‐in strategy of a Cre‐ and Flpe‐dependent dual activator system, harboring the VPR and SAM activator complexes where the defined integration and the optional twofold mode of activation are the prominent features differentiating our line from the SPH transgenic mouse line (Zhou *et al*, 2018). After confirming the technical and biological functionality of the *dCAM* approach, we expanded the toolbox by developing an AAV‐based split‐dCas9‐VP64/SAM system, making it versatile and applicable across species with minimal modifications (Truong *et al*, 2015; Moretti *et al*, 2020). In contrast to the *dCAM* mouse line, dCas9 in this AAV system is fused to the compact VP64 activation domain and is dependent on co‐expressed SAM system to ensure high levels of gene activation. Focusing on AAV‐mediated delivery is important to render these reprogramming approaches versatile for a broad range of applications, such as usage in other model organisms like non‐human primates, and ultimately, for cell reprogramming therapies in patients. To further support the applicability of the system as a therapeutic strategy, we performed *in vitro* experiments using the astrocyte‐specific promoter gfaABC1D (Lee *et al*, 2008), which is comprised of a 681 bp large fragment of the human GFAP promoter. Gold standard for the delivery of the sgRNAs are AAVs, as they exhibit low immunogenicity and ensure high and sustained expression (Grieger & Samulski, 2005; Zaiss & Muruve, 2005; Mattugini *et al*, 2019). Using the intein‐split approach to circumvent the limitations of the AAV packaging limit provides a higher degree of flexibility compared to approaches using compacted system, for example, based on engineered, short Cas9 versions (Zhang *et al*, 2021). By contrast, the AAV‐dCAS system is utilizing the well‐characterized spCas9 and would allow the implementation of more complex, cell‐type‐specific promoters. Due to this, the AAV‐dCAS system is an independent and highly versatile tool, which can be readily adapted for clinical application. Strikingly, with the split‐dCas9 AAV‐based system, we could fully recapitulate the results obtained with *dCAM*, confirming the functionality and robustness of the CRISPRa approach to reprogram striatal astrocytes into induced neurons by multiple gene activation *in vivo*. In light of the ongoing debate about direct reprogramming and the use of AAV virus (Chen, 2021; Wang *et al*, 2021; Wang & Zhang, 2022), we verified the FLEx system which is used in this study to control not only the GFP expression but in the AAV‐dCAS system also the dCas9 expression. Injection into Gfap‐Cre negative animals did not show leakiness of the reporter (Appendix Fig S19). Hence, unspecific promoter activation, reporter, or reprogramming factor expression is unlikely to occur in our system, which is, in contrast to other approaches, not based on AAV delivery of transcription factor cDNA but on the activation of endogenous genes. Effects of high viral titer application is unlikely since in the dCAM setting, both the GFP control and *ALN* animals received the identical amount of AAV. Batch differences of the AAV can be excluded as well, since in the dCAS setting both in GFP control and in *ALN* condition, the identical reporter AAV has been used (Appendix Fig S17). In addition, aberrant activation of the GFAP‐Cre promoter could not be observed in the scRNA‐seq analysis. Moreover, with both induction systems, we obtained similar results albeit the experimental setup differed substantially in regard to dCas9 delivery (lox‐stop‐lox dCas9 transgene vs AAV FLEx dCas9).

Thirteen weeks after injection, the combination *ALN* was capable to generate functional neurons with mature electrophysiological properties, whereas cells reprogrammed by *ALNe‐218* exhibited characteristics reminiscent of astrocytes or immature neurons. Furthermore, *ALN*‐induced neurons led to an improvement in voluntary motor behavior and a balancing of the axial symmetry. This behavioral rescue could be observed to a similar extent, both in *dCAM* as well as *AAV‐dCAS* animals, confirming the biological functionality of the reprogrammed neurons. Surprisingly, in contrast to published and our own *in vitro* experiments using a classical overexpression setup for *ALN* (Addis *et al*, 2011; Theodorou *et al*, 2015), these *de novo* induced neurons were not immunoreactive for the dopaminergic marker TH but for the GABAergic marker Gad65/67. Independent of reprogramming, we observe sporadic GFP^‐^/TH^+^ neurons in the striatum, which represent either naturally occurring TH‐positive interneurons within the striatum, or may emerge due to the 6‐OHDA toxin treatment (Tepper & Koos, 2016; Pereira *et al*, 2017; Mao *et al*, 2019). In this regard, the FLEx‐*GFP* marker employed in this study proved to be beneficial for the identification of induced neurons and its demarcation from reprogramming independent TH^+^ neurons. These discrepancies might be explained by the multitude of differences in the experimental setups. Differences in the respective target cells (Pereira *et al*, 2017) or differential levels of factor expression (Rivetti di Val Cervo *et al*, 2017) as a result of different expression systems like the overexpression from heterologous promoters versus CRISPRa gene induction are likely to influence the terminal neuronal fate. Interestingly, when performing *in vitro* reprogramming of primary murine astrocytes with the dCAS combination ALN, again virtually all induced neurons show GABAergic identity (Appendix Fig S18). Since the induction obtained by CRISPRa is lower compared to cDNA overexpression, it is possible that the unexpected GABAergic identity of the induced neurons is indeed based on the lower levels of factor expression. On the other hand, also the *in vivo* targeting of NG2 glia with a cDNA‐based *ALN* expression system has been shown to generate GABAergic instead of dopaminergic neurons in the striatum (Torper *et al*, 2015; Pereira *et al*, 2017) indicating a strong influence of the specific region and the identity of the targeted glial cells. The influence of the local environment is supported in part by a recent publication of Qian and colleagues utilizing solely the knockdown of the RNA‐binding protein PTB (Qian *et al*, 2020) for reprogramming cells into region‐specific neurons. Only a marginal part of the *ALN*‐induced neurons were positive for Darpp32, a marker for striatal medium spiny neurons representing the main neuronal class within the striatum. In addition, they did not exhibit standard electrophysiological properties of this particular neuronal subtype. This indicates that the reprogrammed neurons presumably differentiate into a distinct different subtype of GABAergic interneurons, capable of modulating striatal motor circuits (Cepeda *et al*, 2008; Gertler *et al*, 2008; Grande *et al*, 2013; Planert *et al*, 2013). Furthermore, their electrophysiological properties are distinct from PV^+^ interneurons, which have been shown by a recent publication to arise during *ALN* overexpression in NG2^+^ oligodendrocyte precursors, which may be explained by the different starter cell populations (Masserdotti *et al*, 2016; Pereira *et al*, 2017). Nevertheless, the electrophysiological characterization as presented here is just a first step. A further detailed characterization of the induced neurons, for example, with specific channel blockers, and their putative integration into the existing networks is needed to resolve these fundamental questions. However, the major impact of this study lies in the fact that the CRISPRa‐induced *ALN* combination in the striatum, using either *dCAM* or *AAV‐dCAS*, induces specific GABAergic neurons, capable of alleviating motor behavior symptoms in a 6‐OHDA model. Accordingly, the amphetamine‐induced rotation, monitoring dopamine receptor activity, remained unaltered supporting the dopamine independent mode of rescue. This is remarkable since the research focus so far has been on the restoration of the dopaminergic drive to alleviate motor symptoms. However, it has been reported that dopamine depletion in 6‐OHDA toxin‐treated PD rodent models has a strong effect on striatal circuits. Specifically, increased excitatory cholinergic and reduced inhibitory GABAergic signals have been observed (Salin *et al*, 2009). In addition, most of the basal striatal excitatory drive arising from cholinergic interneurons is balanced by a concomitant GABAergic inhibition; this signaling is impaired by dopamine deprivation (Lozovaya *et al*, 2018). Furthermore, integrity of the fast spiking striatal GABAergic interneurons has been shown to depend on dopaminergic input from the SN*pc* (Ortega‐de San Luis *et al*, 2018). Altogether, these reports as well as our own findings suggest that the imbalance in striatal microcircuitry—including impaired GABAergic signaling—contribute to the altered motor behavior in parkinsonian state. These observations are supported by Martinez‐Cerdeno *et al* (2010) transplanting GABAergic neuron precursors into the striatum of parkinsonian state rats rescuing in part motor behavior as well. In the basal ganglia circuit, the nigrostriatal dopaminergic inputs act excitatory on the direct pathway and inhibitory on the indirect pathway which results in a misbalance of these motor pathways. Therefore, restoration or reinforcing of GABAergic inhibition in the striatum is an attractive alternative or additional therapeutic concept for PD besides the dopamine replacement strategies (Damier *et al*, 2016).

This study is demonstrating that AAV‐mediated CRISPRa approaches are a suitable and functional tool, which can potentially be employed to any reprogramming approaches in any organ, tissue, and cell type *in vivo* by activating endogenous gene expression. In particular, we show that the *dCAM* mouse line and the universally applicable *AAV‐dCAS* system can rescue PD motor behavior deficits by the direct conversion of endogenous striatal astrocytes into functional GABAergic neurons via CRISPRa‐mediated induction of the reprogramming factors *Ascl1*, *Lmx1a*, and *Nr4a2*. Future experiments deciphering the affected striatal circuits will reveal which strategy, like dopaminergic replacement, GABAergic reprogramming, or a combination of both strategies, will achieve the best therapeutic outcome.

## Material and Methods

### Molecular cloning

#### Generation of the split‐dCas9

The nuclease‐inactivating point mutations D10A and N863A were introduced into the plasmids pAAV_crTLR#1_Nv1 and pAAV_crTLR#1_Cv1 from Truong *et al* (2015) using QuikChange II Site‐Directed Mutagenesis Kit (Agilent Technologies, 200523, USA). miRNA218 cloning was performed according to Rivetti di Val Cervo *et al* (2017).

#### Polymerase chain reaction

PCRs are performed using the Q5 High‐Fidelity 2× Master Mix (NEB, M0492S, USA). For the amplification of GC‐rich regions, the KAPA HiFi HotStart PCR Kit (Kapa Biosystems, KK2501, Swiss) was used. For colony PCR and genotyping reactions, VWR Red Taq DNA Polymerase Master Mix (VWR, 733‐2131, USA) was deployed. For STAgR cloning, the Phusion High‐Fidelity DNA Polymerase (Thermo Fisher, F530S, USA) was applied. Site‐directed mutagenesis was performed using QuikChange II Site‐Directed Mutagenesis Kit (Agilent Technologies, 200523, USA). All reactions were performed according to manufacturer's instructions.

If the PCR product was further employed in cloning steps, it was either PCR purified using QIAquick PCR purification kit (Quiagen, 28104, Netherlands), or gel purified followed by a gel purification step using QIAquick gel extraction kit (Quiagen, 28115, the Netherlands), both reactions were performed according to manufacturer's instructions.

#### Enzymatic digest

Restriction enzymes from New England Biolabs were used according to manufacturer's instructions. For plasmid digest, 500 ng to 1 μg of DNA was digested and subsequently gel purified using QIAquick gel extraction kit (Quiagen, 28115, the Netherlands). For the digest of PCR products, 500 ng of DNA was used followed by a PCR purification using QIAquick PCR purification kit (Quiagen, 28104, the Netherlands), both reactions were performed according to manufacturer's instructions.

#### DNA ligation

DNA fragments were ligated using T4 DNA Ligase (NEB, M0202S, USA) using 20 ng of vector DNA and a molar ratio of vector/insert of 1/3; reaction was performed for 20 min at room temperature. For the ligation of multiple PCR fragments, Gibson assembly was performed using NEBuilder^®^ HiFi DNA Assembly Master Mix (NEB, E2621S, USA); fragments were used in an equimolar ratio and reaction was performed for 1 h at 50°C.

#### sgRNA design and cloning

All sgRNAs were designed using the online tool benchling.com. sgRNAs were targeted to the region −250 bp to the transcriptional start site of the target gene. Two sgRNAs were used per gene. Multiplexed sgRNA cloning was performed using the string assembly sgRNA cloning strategy (STAgR) (Breunig *et al*, 2018). Sequences are listed in Appendix Table S1.

#### Transformation and plasmid purification

Chemically competent DH5α or NEB stable (plasmids for AAV production) bacteria were used for transformation. After a heat shock was performed, bacteria were spread on agar plates containing the suitable selection marker. Plates were incubated over night at 37°C and single colonies were picked for further analysis.

For plasmid purification, Plasmid Mini Kit (Qiagen, 12123, the Netherlands) or EndoFree Plasmid Maxi Kit (Qiagen, 12163, the Netherlands) was used according to manufacturer's instructions.

### Cell culture

All cells are incubated at 37°C with 7.5% CO_2_. Neuro2A cell line was purchased from ATCC (ATCC, CCL‐131, USA). Cells are cultures in DMEM/F12 GlutaMAX^TM^‐I medium with 10% FCS.

#### Isolation of primary cortical astrocytes

Primary cortical astrocytes were obtained from postnatal (P5‐P6) mice following a protocol adapted from Heinrich and colleagues (Heinrich *et al*, 2011). After tissue dissection, the cortices were dissociated and purified using the Adult Brain Dissociation Kit from Miltenyi (Miltenyi, 130107677, Germany). Instead of using the gentleMACS Octo Dissociator, the tissue was kept in the enzyme mixture for 30 min, every 10 min the mixture was pipetted up and down (five times) using a 10 ml serological pipette for tissue dissociation. Subsequently the protocol was performed according to manufacturer's instructions without conducting the red blood cell removal. For the purification of astrocytes, the cortical cell mixture was separated using the Anti‐ACSA‐2 MicroMead Kit (Miltenyi, 130097678, Germany). As soon as the cells reach a confluency of ~ 80% (day 7–10), 300,000 cells were seeded per six well.

#### Lipofection

Astrocytes were transfected using Lipofectamine 2000 (Invitrogen, 11668, USA) according to manufacturer's instructions. 30 min prior to the lipofection, cells are equilibrated in 1.5 ml OptiMEM with 10% glutamine. 3.6 µg of DNA is transfected per six well using a DNA/lipofectamine ratio of 1/3. 4 h later, the transfection media is removed and exchanged by conditioned astrocyte media. 48 h after the transfection, the RNA is isolated, respectively cells are fixed using 4% paraformaldehyde for immunocytochemistry.

#### FACS sorting

Astrocytes were trypsinated for 5 min using 0.05% trypsin‐EDTA (Thermo Fisher, 25300054, USA), reaction was stopped with PBS pH 7.4 with 5% fetal bovine serum (Thermo Fisher, A2153, USA). After centrifugation, cells were resuspended in PBS pH 7.4 with 0.5% fetal bovine serum and kept on ice until further processing. The green fluorescent protein was co‐transfected in order to enrich cells that were successfully transfected. Green cells were enriched with the BD FACSaria II controlled with the BD FACSDiva Software Version 6.1.3 (BD Biosciences, USA), cells were collected and further processed for RNA isolation.

#### RNA isolation, cDNA preparation

Given the low transfection efficiency, cells are sorted using the FACSARIA III (Biosciences) with a 100 µm nozzle according to GFP signal, expressed from a co‐transfected plasmid. RNA is isolated using PicoPure RNA Isolation Kit (Invitrogen, KIT0204, USA). cDNA is produced using SuperScript VILO cDNA Synthesis Kit (Thermo Fisher, 11754050, USA).

#### Real‐time qPCR

qPCR is performed using TaqMan Universal Master Mix (Thermo Fisher, 4304437, USA) and TaqMan probes, all probes are listed in the supplementary information. Reaction was performed according to manufacturer's instructions. RT–qPCR was carried out using an ABI Prism 7900 HT Real‐Time PCR System and SDS 2.4.1 software. TaqMan probes are listed in Appendix Table S2.

#### Immunocytochemistry

Cells were fixed using 4% paraformaldehyde. Primary and secondary antibodies were diluted in PBS containing 1% bovine serum albumin and 0.5% Triton X‐100. Primary antibody was incubated overnight at 4°C, secondary antibody was incubated for 1 h at room temperature. Primary antibodies: mouse‐anti‐ASCL1 1:1,000 (BD Bioscience, 556604, USA), rabbit‐anti‐LMX1A 1:2,000 (Merck‐Millipore, ab10533, Germany), mouse‐anti‐Nr4a2 1:2,000 (Santa Cruz, sc‐376984, USA), rabbit‐anti‐Flagtag 1:1,000 (Sigma, F1804, USA), rabbit‐anti‐MAP2 1:1,000 (Merck‐Millipore, AB5622, Germany). Secondary antibodies: Donkey anti‐mouse IgG Alexa Fluor 594 1:500 (Thermo Fisher Scientific, A‐21203, Germany), donkey anti‐rabbit IgG Alexa Fluor 594 1:500 (Thermo Fisher Scientific, A‐21207, Germany). Coverslips were mounted onto glass slides using Aqua‐Poly/Mount.

#### Lentivirus production

The lentiviral constructs were generated as a Tet‐O‐driven split‐Cas system with N‐dCas9 (SpCas92–573) fused to DnaE‐N‐Intein and C‐dCas9‐(SpCas9574–1368) fused to DnaE‐C‐Intein, similar to the AAV‐dCAM setup. N‐Cas9 was combined with gRNA Ascl1‐1 and SAM, C‐Cas9 was combined with gRNA Ascl1‐2 and VPR. For the N‐Cas vector, lenti‐sgRNA(MS2)zeo backbone containing gRNA gRNA Ascl1‐1 was digested using BamHI and EcoRI to remove Ef1a‐zeomycine. The Tet‐O‐promoter was amplified from Tet‐O‐FUW (Caiazzo *et al*, 2011) and cloned into the lenti‐sgRNA(MS2) backbone. The SAM complex was amplified from lenti MS2‐P65‐HSF1_Hygro plasmid (Addgene, 61426) and cloned together with a P2A sequence and the amplified N‐dCas9 into the lenti‐sgRNA(MS2)‐Tet‐O backbone. The obtained vector contained hU6_Ascl1‐2_tet‐O_SAM_P2A_N‐Cas_N‐intein. For the C‐Cas‐VPR construct, C‐dCas‐VPR was amplified from SP‐dCas9‐VPR (Addgene, 63798). The coding sequence was transferred to the lenti‐sgRNA(MS2)‐Tet‐O backbone containing gRNA Ascl1‐2. The obtained vector contained hU6_Ascl1‐2_Tet‐O_C‐intein_C‐Cas_VPR. As control, a dsRed expressing lentivirus Tet‐O_T2A_dsRed is utilized. Production and titer determination of replication incompetent, self‐inactivating lentiviruses was performed as described previously (Theodorou *et al*, 2015).

### Western Blot

Primary antibodies were diluted in TBS‐T containing 0.5% milk powder and incubated over night at 4°C. Primary antibodies: rabbit‐anti‐HA tag (C29F4) 1:500 (Cell Signaling, 3724, USA), mouse‐anti‐β‐Actin 1:10,000 (GeneTex, GTX26276, USA), anti‐mouse‐N‐Cas9 1:500 (Epigentek, A‐9000, USA), anti‐mouse‐C‐Cas9 1:1,000 (Novus biologicals, NBP2‐52398SS, USA), anti‐rabbit‐P2A 1:1,000 (Sigma‐Aldrich, ABS31, USA). Secondary antibodies were diluted in TBS‐T containing 5% milk powder and incubated for 1 h at room temperature. Secondary antibodies: Goat anti‐rabbit IgG HRPO 1:5,000 (Dianova, 111‐035‐003, USA), goat anti‐mouse IgG HRPO 1:5,000 (Dianova, 115‐035‐003, USA).

### Animals

For the analysis, the B6.Cg‐Tg(Gfap‐cre)77.6Mvs/2J (GFAP‐Cre) was purchased from Jackson Laboratories (024098), the line was further bred on a C57BL/6N background. The Rosa26‐dCas‐activator mouse line (dCAM) was produced on a B6N background. For the analysis, littermates of the B6.Cg‐Tg(Gfap‐cre)77.6Mvs/2J × dCAM/N line was used. When crossing the B6.Cg‐Tg(Gfap‐cre)77.6Mvs/2J line with transgenic animal carrying *LoxP* cassettes, it was payed attention to only breed female Cre mice, as it is known for this line to have Cre expression in the male germline.

#### Generation of CRISPR‐Activator mouse line via microinjection of one‐cell embryo

The Rosa26‐dCas‐activator mouse line was generated using CRISPR/Cas9‐based gene editing by microinjection into one‐cell embryos. For this, a gene‐specific guide RNA (Rosa26_gRNA 5′‐ACTCCAGTCTTTCTAGAAGA‐3′) was used as *in vitro* transcribed single gRNA (EnGen^®^ sgRNA Synthesis Kit, NEB, E3322, USA). Prior to pronuclear injection, gRNA (25 ng/µl) and targeting vector (50 ng/µl) were diluted in microinjection buffer (10 mM Tris, 0.1 mM EDTA, pH 7.2) together with recombinant Cas9 protein (50 ng/µl, IDT, Coralville, USA) and incubated for 10 min at room temperature and 10 min at 37°C to form the active ribonucleoprotein (RNP) complex. One‐cell embryos were obtained by mating of C57BL/6N males (obtained from Charles River, Sulzbach, Germany) with C57BL/6N females superovulated with five units PMSG (Pregnant Mare's Serum Gonadotropin) and five units Human Chorionic Gonadotropin. For microinjections, one‐cell embryos were injected into the larger pronucleus. Following injection, zygotes were transferred into pseudo‐pregnant CD1 female mice to obtain live pups. All mice showed normal development and appeared healthy. Handling of the animals was performed in accordance to institutional guidelines and approved by the animal welfare committee of the government of upper Bavaria. The mice were housed in standard cages in a specific pathogen‐free facility on a 12 h light/dark cycle with *ad libitum* access to food and water. Analysis of gene editing events was performed on genomic DNA isolated from ear biopsies of founder mice and F1 progeny, using the Wizard Genomic DNA Purification Kit (Promega, A1120, Germany) following the manufacturer's instructions.

#### Animal housing

Animal housing and handling protocols were approved by the committee for the Care and Use of Laboratory animals of the Government of Upper Bavaria (Germany) and were carried out in accordance with the European Communities' Council Directive 2010/63/EU. During the work, all efforts were made to minimize animal suffering. All mouse lines were kept in a controlled pathogen‐free (SPF) hygiene standard environment on a 12 h light/dark cycle. The mice had access to *ad libitum* standard feed and water always. All tests were approved for the ethical treatment of animals by the Government of Upper Bavaria.

#### 6‐OHDA lesion

Adult (3–4 months) mice were chosen for dopamine depletion of the left striatum, mice received a unilateral injection of 6‐hydroxydopamine‐HCl (6‐OHDA‐HCl) (Sigma‐Aldrich, H4381, USA) into the left medial forebrain bundle (MFB). All animals receive intraperitoneal injection of Medetomidin (0.5 mg/kg), Midazolam (5 mg/kg), Fentanyl (0.05 mg/kg) (MMF) as anesthesia. The mouse received pre‐emptive Metamizol (200 mg/kg s.c.) and a local subcutaneous injection of 2% Lidocain. The animal was positioned into the stereotactic frame containing an integrated warming base (Stoelting, 51730D, USA) to maintain normothermia. 6‐OHDA‐HCl was dissolved in 0.2% ascorbic acid (Sigma‐Aldrich, A4403, USA) in saline at a concentration of 2 µg/µl of free‐base 6‐OHDA‐HCl. Each mouse was injected 1.5 µl (0.2 μl/min) of solution into the left MFB according to the following coordinates: anteroposterior (AP) −1.2, mediolateral (ML) +1, dorsoventral (DV) −4.9 (all millimeters relative to bregma) with flat skull position. The needle was left in place for 3 min after the injection to allow the toxin to diffuse before slow withdrawal of the capillary. Mice were woken up from anesthesia by the subcutaneous injection of Atipamezol (2.5 mg/kg) and Flumazenil (0.5 mg/kg). Mice were left for recovery for 2 weeks before experimentation.

#### Stereotactic injection

The dopamine‐depleted animals were injected into the ipsilateral striatum with high‐titer recombinant adeno‐associated virus (AAV). Mice were anesthetized with MMF and received pre‐emptive pain treatment as for the 6‐OHDA‐HCl injection; subsequently they were positioned into the stereotactic frame with flat skull position. Each mouse received 1 μl rAAV2/5 (0.2 μl/min) into the left dorsal striatum according to the following coordinates: AP +1, ML +2.1, DV −3.5 (all millimeters relative to bregma). The needle was left in place for 3 min after the injection to allow the virus to diffuse before slow withdrawal of the capillary. For recovery, the antagonists Atipamezol (2.5 mg/kg) and Flumazenil (0.5 mg/kg) were injected subcutaneously.

### rAAV production

High‐titer preparations of rAAV2/5 were produced based on the protocol of Zolotukhin and colleagues (Zolotukhin *et al*, 1999) with minor modifications. In brief, HEK 293T cells were transfected with the CaPO_4_ precipitation method, the plasmids pRC5, Ad helper and pAAV were applied in an equimolar ratio. After 72 h, cell pellet was harvested with AAV release solution, 50 U/ml benzonase was added, then solution was incubated for 2 h at 37°C. Cells were frozen and thawed in liquid nitrogen to allow rAAV release. Purification of rAAV vector was done with iodixanol densities gradient (consisting of 15, 25, 40, and 56% iodixanol), followed by gradient spinning at 50,000 rpm for 2 h 17 min at 22°C in a Ti70 rotor (Beckman, Fullerton, CA, USA). rAAV was collected at 40% iodixanol with a 5 ml syringe. Virus was dialyzed (Slide‐A‐Lyzer 10,000 MWCO 5 ml) in buffer A overnight to remove iodixanol. Anion‐exchange chromatography column HiTrap Q FF sepharose column and Superloop were connected with the ÄKTAprime plus chromatography system to collect the eluted fraction. To measure rAAV concentration, the eluted fraction was spun and washed once in PBS‐MK Pluronic‐F68 buffer with a Millipore 30 K MWCO 6 ml filter unit. rAAVs were stored in a glass vial tube at 4°C. rAAVs were titered by SYBR Green qPCR with GFP or SV40 primer (D'Costa *et al*, 2016). Usual titer range was 3 × 10^14^ to 5 × 10^15^ gc/ml. (genome copies per milliliter). Total amounts per injection using 0.25 µl per AAV corresponds to 1 × 10^11^ up to 1 × 10^12^ gc per virus.

### Immunohistochemistry

For histological analysis, the mice were asphyxiated with CO_2_ and perfused transcardially with 4% ice‐cold paraformaldehyde (PFA; Sigma‐Aldrich, P6148, USA) in 0.1 M PBS with pH 7.4. After dissection the brain was post‐fixed in PFA overnight at 4°C followed by storage in 30% sucrose for minimum 24 h at 4°C. Brains were cut coronal into 40 µm thick serial sections on a cryostat (Thermo Fisher Scientific, HM 560 Kryostat, Microm, Germany). Free floating sections were stored at 4°C in cyro protection solution (50% PBS pH 7.4, 25% ethylene glycol [Carl Roth, 2441, Germany], 25% glycerol [Sigma‐Aldrich, G9012, USA]) until further processing.

In general, sections were blocked in PBS pH 7.4 with 2% fetal bovine serum (Thermo Fisher, A2153, USA) and 0.1% Triton X‐100 (Sigma‐Aldrich, T9284, USA) for 2 h. Subsequently, brain slices were incubated over night at 4°C in primary antibody diluted in blocking solution. Sections were three times washed for 15 min with PBS pH 7.4 before incubated with secondary antibody diluted in PBS pH 7.4 containing 0.1% Triton X‐100 (Sigma‐Aldrich, T9284, USA) for 1 h at room temperature. Slices were washed with 100 ng/ml DAPI‐PBS solution pH 7.4 (Sigma‐Aldrich, D8417, USA) for 5 min, followed by three 15 min washes with PBS pH 7.4. Slices were mounted on coverslips using Aqua‐Poly/Mount (Polysciences, 18606, USA). For the NeuN staining, the sections were undertaken an antigen retrieval protocol. In short, the sections were incubated in 0.01 M Na‐citrate buffer pH 6 at 80°C for 45 min and allowed to cool down to room temperature per se. Subsequently, brain slices were blocked in 3% milk solution containing 0.3% Triton X‐100 for 2 h. Sections are incubated overnight at 4°C in primary antibody diluted in blocking solution. Sections are washed three times for 1 h in PBS pH 7.4 containing 0.3% Triton X‐100 and incubated overnight at 4°C in secondary antibody diluted in blocking solution. Slices were washed with 100 ng/ml DAPI‐PBS solution pH 7.4 (Sigma‐Aldrich, D8417, USA) for 5 min, followed by three 15 min washes with PBS pH 7.4. Slices were mounted on coverslips using Aqua‐Poly/Mount (Polysciences, 18606, USA). Primary antibodies: rabbit‐anti‐tyrosine hydroxylase 1:500 (Pel‐Freeze, P40101, USA), mouse‐anti‐NeuN 1:1,000 (Abcam, ab104224, USA), anti‐chicken‐GFP 1:1,000 (Abcam, ab13970, USA), anti‐rabbit‐GFAP 1:1,000 (Abcam,ab7260, USA), anti‐mouse‐Parvalbumin 1:1,000 (Sigma‐Aldrich, P3088, USA), anti‐rabbit‐calretinin 1:1,000 (Swant, CR7697, Switzerland), anti‐goat‐CHAT 1:100 (Merck‐Millipore, AB144P, Germany), anti‐rabbit‐Gad65/67 1:500 (Abcam, ab49832, USA), anti‐mouse‐vGLUT1 1:1,000 (Atlas, AMAb91041, USA), anti‐rabbit‐DARPP32 1:500 (Abcam, ab40801, USA), anti‐rabbit‐MAP2 1:500 (Merck‐Millipore, ab5622, Germany), anti‐rabbit‐TUJ1 1:500 (Abcam, ab18207, USA). Secondary antibodies: Donkey anti‐mouse IgG Alexa Fluor 594 1:500 (Thermo Fisher Scientific, A‐21203, Germany), donkey anti‐rabbit IgG Alexa Fluor 594 1:500 (Thermo Fisher Scientific, A‐21207, Germany), donkey anti‐chicken IgY Alexa Fluor 488 1:250 (Dianova, 703‐546‐155, Germany).

### Image acquisition

All images were acquired on a confocal laser scanning (Zeiss LSM880) microscope or an Axioplan2 microscope and an AxioCam MRc camera (Carl Zeiss AG, Germany) if not differently indicated. Images were processed with AxioVision 4.6 (Carl Zeiss AG, Germany) and Adobe Photoshop CS6 (Adobe Systems Inc., USA) software.

### Cell counting

All stereological quantifications were performed using the Stereoinvestigator Zeiss Imager M2 with the software version 2019.1.3. The dorsal striatum of at least three animals was analyzed for quantification. Regions close to the subventricular zone were excluded from counting. For all quantifications, samples were randomized and experimenters were blinded to the treatment conditions.

### Behavior analysis

#### Catwalk

Mice were tested on an automated, video‐based gait analysis system, the CatWalk XT (Noldus, Wageningen, the Netherlands). The animals walk over an elevated glass walkway (width 8 cm, length 100 cm) enclosed by plexiglas walls (height 14 cm) in a dark room. A camera (Pulnix Camera RM‐765) situated below the middle of the walkway tracked the illuminated footprints, which were later analyzed with the CatWalk software Version 7.1. The software automatically calculates a wide number of parameters in several categories which describe gait in spatial and temporal aspects. For a more detailed description, see Hölter and Glasl (2012) and Zimprich *et al* (2018).

#### Drug‐induced rotation analyses

The mice were placed individually in plexiglas cylinders (diameter 12.5 cm, height 30 cm). Experiments were recorded from a ventral plane view, videos were analyzed with the automated 90° body rotation counts using Ethovision software (Ethovision XT 14, Netherlands). Mice were allowed to habituate for 15 min before monitoring for 45 min. Amphetamine was dissolved in saline at a concentration of 0.5 mg/ml; each mouse received an intraperitoneal injection of 5 mg/kg before being placed into the cylinder.

#### Vertical pole test

Mice were placed facing upwards onto a wooden, rough‐surfaced pole (length 50 cm, diameter 1 cm) with a square base plate. Mice were tested for the time they need to turn downwards (latency time) and the total time they need to reach the base of the pole (total time). Right before the test trials, the mice were trained in small groups with < 10 animals. Each mouse was coached three to five times before moving on to the next one. Then three test trials were performed with each mouse in the same sequential order, so that the time interval between training and testing was the same for each individual.

### Electrophysiology

#### Preparation of brain slices

Acute 220 µm thick brain coronal slices containing the dorsal striatum were cut on a vibratome (Leica VT1200, Germany) in a bubbled (95% O_2_/5% CO_2_) standard ice‐cold artificial cerebrospinal fluid (ACSF) containing (in mM): 126 NaCl, 2.5 KCl, 1.2 MgCl_2_, 2.4 CaCl_2_, 1.2 NaH_2_PO_4_, 21.4 NaHCO_3_, 11.1 glucose, complemented from slicing only with (in mM): 3 kynurenic acid, 26.2 NaHCO_3_, 225 sucrose, 1.25 glucose and 4.9 MgCl_2_. Slices were then transferred to a chamber containing standard ACSF oxygenated with 95% O_2_/5% CO_2_ at 35°C for 15 min and subsequently maintained at room temperature for at least another 15 min prior to use.

#### Whole‐cell recordings

Dorsal striatal “reprogrammed” cells (either neurons or glia) were visualized with a 20×/1.0NA WI objective, 4× post‐magnification, under video microscope (Olympus BX51WI, Germany) coupled with infrared gradient contrast and epifluorescence. Whole‐cell patch‐clamp recordings in current clamp mode were acquired from the somata of fluorescent cells with a Multiclamp 700B amplifier (Molecular Devices, Foster City, CA), digitized at 10 kHz and Bessel filtered at 4 kHz. Pipettes (4–6 mΩ) were filled with an intracellular solution containing (in mM): 100 K‐gluconate, 20 KCl, 4 Mg‐ATP, 0.3 Na‐GTP, 10 Na_2_‐Phosphocreatine, 10 Hepes, (pH 7.3, 290 mOsm). All recordings were carried out at 35°C and slices continually superfused with oxygenated (95% O_2_/5% CO_2_) ACSF. Passive membrane properties were assessed by injecting 500 ms depolarizing current steps. Putative spontaneous postsynaptic potentials were recorded with the same internal solution in voltage clamp mode while the cell being held at −70 mV. Data were analyzed with custom‐written routines in IgorPro.

### Single cell analysis

#### Tissue dissociation

Tissue blocks of approximately 5–7 mm³ were dissected from the dorsal mouse striatum (*n* = 2) and dissociated into single cell suspension using the papain kit (Worthington) according to manufacturer's instructions. Incubation with dissociating enzyme was performed for 90 min.

#### Library preparation and sequencing

Single cell suspensions were loaded onto 10× Genomics Single Cell 3′ Chips together with the reverse transcription mastermix according to manufacturer's instructions for the Chromium Single Cell 3′Library & Gel Bead Kit v2 (PN‐120237, 10×Genomics) to generate single cell gel beads in emulsion (GEMs). cDNA synthesis was done according to 10× Genomics guidelines. Libraries were pooled and sequenced on a NovaSeq6000 (Illumina) according to the Chromium Single Cell v.2 specifications and with an average read depth of 50,000 aligned reads per cell. Sequencing was performed in the genome analysis center of the Helmholtz Center Munich.

#### Alignment and data analysis

Transcriptome alignment of single cell data was done using Cell Ranger 3.1.0 against a modified version of the mouse transcriptome GrCm38 (Ensembl Release 99) that included both GFP and Cre sequences. Quality Control (QC) of mapped cells was done using recommendations by Luecken and Theis (2019), selecting 3,899 cells with at least 800 reads and 250 detected genes. Normalization and log transformation was performed using the counts per million (CPM) strategy with a target count depth of 10,000 using SCANPY's (Wolf *et al*, 2018) normalize_total and log1p functions. Highly variable gene selection was performed via the function highly_variable_genes using the Seurat^49^ flavor with default parametrization, obtaining 4,274 HVGs in at least one experimental group. Following cell count normalization experimental groups were integrated with Scanorama (Hie *et al*, 2019). Unsupervised clustering of cells was done using the Leiden algorithm (Traag *et al*, 2019) as implemented in SCANPY and with resolution parameter of 0.05. This allowed classification and counting of nine main cell types based on marker genes selected using *t*‐test between the normalized counts of each marker gene in a cell type against all others (function *rank_genes_groups* in SCANPY). 1,110 cells assigned to astrocytic and neuronal cell types were subclustered into four groups using Leiden with a resolution of 0.30. Marker genes in these four groups were detected using *t*‐test between each group against the other three. Detection of cells positive for GFP, Cre, and other marker genes was done using as criteria any cell with normalized counts greater than zero. Visualization of cell groups is done using Uniform Manifold Approximation and Projection (UMAP) (preprint: Melville *et al*, 2018), as implemented in SCANPY.

### Statistics

Statistical analysis was performed using Graphpad Prism 7 software. If not differently indicated, at least three biological replicates were analyzed. The normality of the distribution of data points was verified using Shapiro–Wilk test. Data was analyzed using either an unpaired *t*‐test or a multiple comparison ANOVA, followed by a *post hoc* Tukey's multiple comparisons test. When normality tests did not indicate normal distribution, non‐parametric Kruskal–Wallis test was performed. Asterisks are assigned as follows: *P < 0.5, **P < 0.01, ***P < 0.001, ****P < 0.0001.

## Author contributions

**Jessica Giehrl‐Schwab:** Conceptualization; Formal analysis; Validation; Investigation; Visualization; Writing—original draft. **Florian Giesert:** Conceptualization; Formal analysis; Supervision; Validation; Investigation; Visualization; Writing—original draft; Project administration; Writing—review and editing. **Benedict Rauser:** Conceptualization; Supervision; Investigation. **Chu Lan Lao:** Resources. **Sina Hembach:** Supervision; Investigation. **Sandrine Lefort:** Validation; Investigation; Visualization; Writing—original draft. **Ignacio Ibarra Del Río:** Resources; Data curation; Software; Formal analysis; Investigation; Visualization; Writing—original draft; Writing—review and editing. **Christina Koupourtidou:** Resources; Investigation; Methodology. **Malte Daniel Luecken:** Conceptualization; Resources; Data curation; Supervision. **Dong‐Jiunn Jeffery Truong:** Conceptualization; Resources. **Judith Fischer‐Sternjak:** Supervision; Project administration. **Giacomo Masserdotti:** Resources; Supervision. **Nilima Prakash:** Conceptualization; Supervision; Validation. **Jovica Ninkovic:** Resources; Supervision. **Sabine M Hölter:** Supervision; Project administration. **Daniela Vogt‐Weisenhorn:** Conceptualization; Supervision; Funding acquisition; Project administration. **Fabian J Theis:** Conceptualization; Supervision. **Magdalena Götz:** Conceptualization; Supervision. **Wolfgang Wurst:** Conceptualization; Supervision; Funding acquisition; Writing—original draft; Writing—review and editing.

In addition to the CRediT author contributions listed above, the contributions in detail are:

WW, JG‐S, FG, and BR conceived the experiments and discussed the data. WW, JG‐S, FG wrote the manuscript. JG‐S, FG, BR, and SH performed all experiments except those specified below. CLL provided viral expertise and produced the AAVs. SL performed all electrophysiological measurements and analysis. D‐JT provided split‐Cas9 expertise and plasmids. DMVW and JF‐S provided expertise for writing the animal procedure protocol. SMH provided expertise and scientific input for the animal behavior tests. JN provided expertise for the design of the single cell sequencing experiment. CKassisted the preparation of the single cell sequencing samples. ILI performed the computational analysis of the scRNA‐seq under supervision of ML and FJT; GM, MG, and NP discussed the data and reviewed the manuscript. All authors had input and gave final approval of the manuscript.

## Disclosure and competing interests statement

JG‐S, FG, BR, and WW have filed a patent based on this work.

## Supporting information



Appendix

Source Data for Appendix

Source Data for Figure 1

Source Data for Figure 2

Source Data for Figure 3

Source Data for Figure 4

Source Data for Figure 7

## Data Availability

Scripts and instructions for reproducibility of the presented analyses, including input and output of relevant steps are available at http://github.com/theislab/astrocytes_reprogramming_analysis. The scRNA‐seq raw and processed sequencing data has been deposited at the Gene Expression Omnibus. The raw sequencing data are available at the Gene Expression Omnibus (GEO) data repository under the accession number GSE149872 (http://www.ncbi.nlm.nih.gov/geo/query/acc.cgi?acc=GSE149872).
