# Supplementary material for: Parkinson's disease motor symptoms rescue by CRISPRa‐reprogramming astrocytes into GABAergic neurons
Source: EMBO Mol Med. 2022 Apr 4;14(5):e14797. doi: 10.15252/emmm.202114797 (PMC9081909; doi:10.15252/emmm.202114797)
Supplement: Supplementary file 1 — Appendix [file EMMM-14-e14797-s007.pdf]

# Appendix:

**Parkinson's disease motor symptoms rescue by CRISPRa-reprogramming astrocytes into GABAergic neurons**

J. Giehrl-Schwab, F. Giesert, B. Rauser, C.L. Lao, S. Hembach, S. Lefort, I.L. Ibarra, C. Koupourtidou, M.D. Luecken, D.J. Truong, J. Fischer-Sternjak, G. Masserdotti, Nilima Prakash J. Ninkovic, S.M. Hölter, D.M. Vogt Weisenhorn, F.J. Theis, M. Götz, W. Wurst

## Table of content

Appendix Figure S1: Design and evaluation of the dCAM line 2
Appendix Figure S2: Evaluation of dCAM x Gfap-Cre primary astrocytes 4
Appendix Figure S3: Evaluation of 6-ODHA induced lesion 5
Appendix Figure S4: Amount and regional distribution of GFP+ cells 6
Appendix Figure S5: In vivo reprogramming 5 wpi – dCAM 7
Appendix Figure S6: Activation of endogenous genes 8
Appendix Figure S7: Evaluation of AAV-dCAS system in vitro 9
Appendix Figure S8: In vivo reprogramming 5 wpi - AAV-dCAS 11
Appendix Figure S9: Neurotransmitter identities 12
Appendix Figure S10: Darpp32 staining and quantification 13 wpi 13
Appendix Figure S11: Phenotypical characterization of AAV-dCAS neurons 14
Appendix Figure S12: Quality control of single cell RNA-seq 15
Appendix Figure S13: Rank_genes_groups plots 16
Appendix Figure S14: scRNA-seq analysis 18
Appendix Figure S15: Additional electrophysiological measurements 19
Appendix Figure S16: Motor behavior analysis 20
Appendix Figure S17: AAV combinations 21
Appendix Figure S18: dCAS reprogramming of primary astrocytes 22
Appendix Figure S19: AAV-dCAS injection into Gfap-Cre negative mice 23
Appendix Table S1: Sequences of gRNAs 24
Appendix Table S2: TaqMan probes 25
Sequence data 25

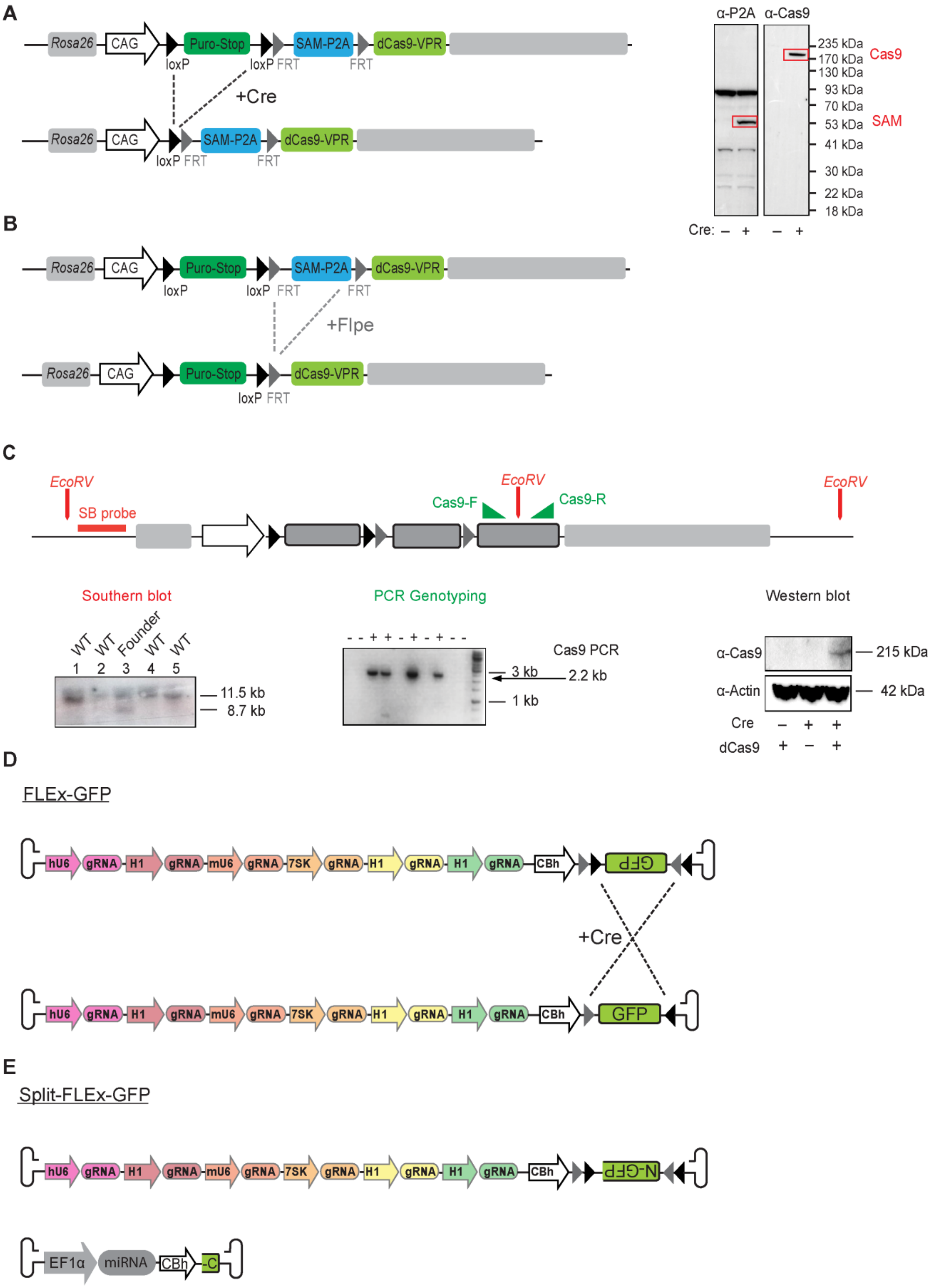
A
Rosa26
CAG
Puro-Stop
SAM-P2A
dCas9-VPR
loxP
loxP FRT
FRT
+Cre
loxP FRT
FRT
α-P2A
α-Cas9
235 kDa
170 kDa
130 kDa
93 kDa
70 kDa
53 kDa
41 kDa
30 kDa
22 kDa
18 kDa
Cas9
SAM
Cre: – + – +
B
+Flpe
C
EcoRV
SB probe
Cas9-F
Cas9-R
Southern blot
WT WT Founder WT WT
1 2 3 4 5
11.5 kb
8.7 kb
PCR Genotyping
- - + + - + - + - -
Cas9 PCR
3 kb
2.2 kb
1 kb
Western blot
α-Cas9
215 kDa
α-Actin
42 kDa
Cre – + +
dCas9 + – +
D
FLEx-GFP
hU6
gRNA
H1
mU6
7SK
CBh
GFP
+Cre
E
Split-FLEx-GFP
N-GFP
EF1α
miRNA
C

**Appendix Figure S1: Design and evaluation of the *dCAM* line.**

**A,** The *LoxP*-flanked puro-stop cassette ensures highly specific knock-in expression. Western blot analysis of targeting construct in Neuro2A cells. Left blot –Test of the P2A sequence for appropriate cleavage. Antibody binds 5´part of the P2A, SAM-5´-P2A and detects a 55 kDa peptide. No uncleaved fusion products are observede. Right blot – Test of the puro-stop-cassette. Without Cre no Cas9 protein is visible **B,** Variable activation levels can be achieved by removing the *FRT*-flanked SAM activator via flippase induced recombination. **C,** Rosa26 knock-in design, homology arms are used 5´arm 1 kb and 3´arm 4 kb long. Southern blot analysis of the founder animals. gDNA digest using EcoRV results in one wild type fragment of 11.5 kb and one 8.7 kb knock-in fragment indicating the heterozygous knock-in in mouse number 3, which was used for further breeding. Genotyping PCR of F1 generation using Cas9 F and Cas9 R primers, 4 (No. 3, 4, 6, 8) out of 10 animals show knock-in. Western blot from primary astrocytes of the *dCAM x GFAP-cre* line. dCas9 is only detected when Cre was expressed. **D**, For *in vivo* activation an AAV containing 6 sgRNAs and a reporter gene can be applied. **E,** If more than 6 sgRNAs shall be used for *in vivo* activation two AAVs containing 12 sgRNAs or 6 sgRNAs and a miRNA expression cassette can be applied with a split-reporter gene. AAVs contain sgRNAs, whose expression is driven by the different Pol III promoters (H1, hU6, mU6 and 7SK), and the marker gene FLEx-*GFP*, respectively split-FLEx-*GFP*, is expressed by the CBh promoter and also delivered by AAVs.

Abbreviations: Puro – puromycin resistance, SAM - synergistic activation mediator (MS2: MS2 bacteriophage coat protein, p65: p65 subunit of human NF-κB, HSF1: Heat shock factor 1), P2A – 2A self-cleaving peptide, dCas9- deadCas9 (nuclease-deficient), VPR - VP64: 4x VP16 herpes simplex virus protein vmw65, p65, Rta: Regulator of transcriptional activation, CAG - CMV early enhancer/chicken β actin promoter.

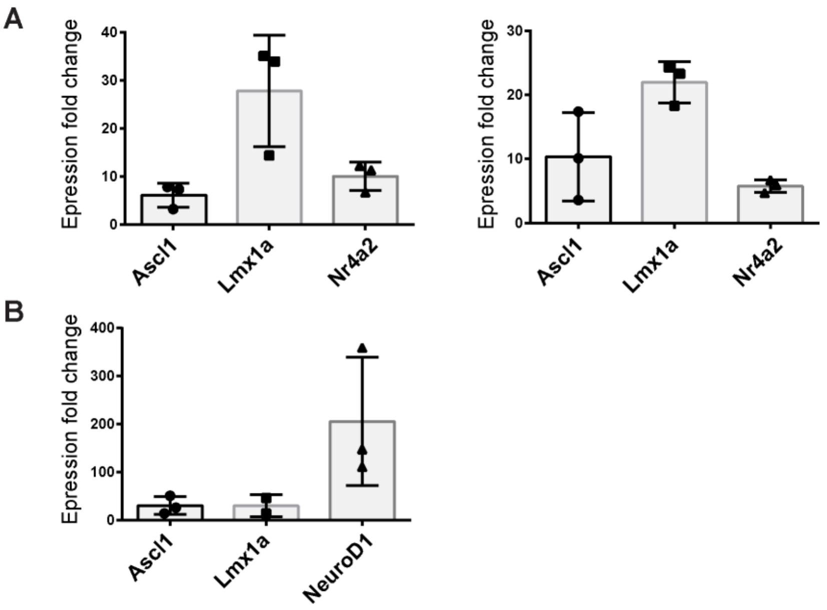


**Appendix Figure S2: Evaluation of *dCAM x Gfap-Cre* primary astrocytes for the activation capacity.**

**A,** Multiplexed activation of *Ascl1*, *Lmx1a* and *Nr4a2*; independent replicates: left: *Ascl1* 6 ± 3, *Lmx1a* 28 ± 12, *Nr4a2* 10 ± 3, right: *Ascl1* 10 ± 7, *Lmx1a* 22 ± 3, *Nr4a2* 6 ± 1) **B,** Multiplexed activation of *Ascl1*, *Lmx1a* and *NeuroD1*. (*Ascl1* 31 ± 19, *Lmx1a* 30 ± 23, *NeuroD1* 206 ± 134). Activation levels are depicted as fold change between cells transfected with and without sgRNAs. All levels were normalized to β-Actin. Error bars represent mean ± SD between technical replicates.

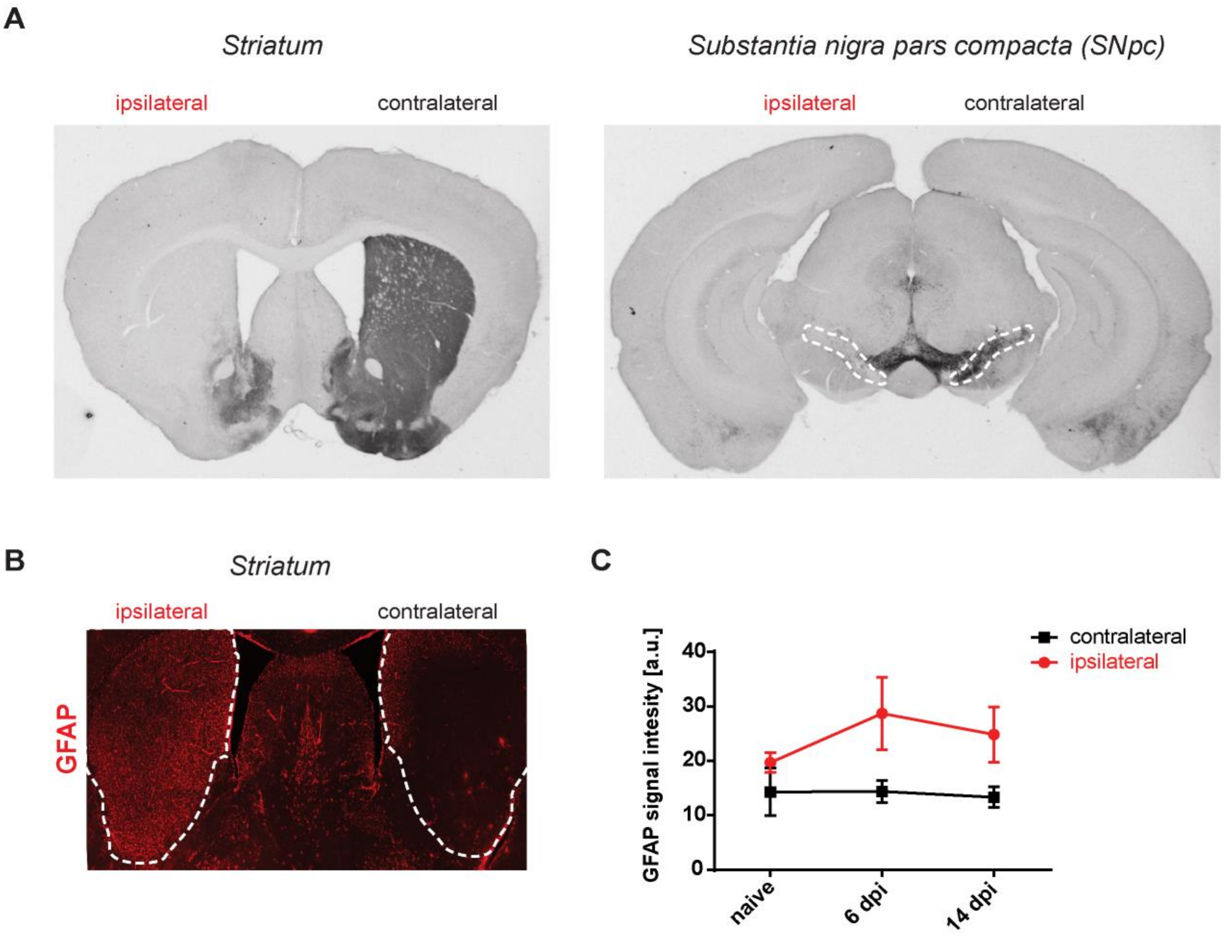


**Appendix Figure S3: Evaluation of 6-ODHA induced lesion.**

**A, B,** Immunohistochemistry 14 days after the 6-OHDA injection into the medium forebrain bundle. **A,** Immunohistochemical staining of dopaminergic lesion using the marker tyrosine hydroxylase (TH). **B,** Staining with the astrocytic marker GFAP to assess the reactive gliosis. **C,** Reactive gliosis was assessed via the signal intensity of GFAP stained striata. Naïve, 6 days post lesion (dpl) and 14 dpl animals were analyzed. Per condition data was collected from two animals, from each animal ten images were analyzed, randomly taken in striatal regions. Ipsilateral: Naïve 19.7 ± 1.8, 6 dpl 28.7 ± 6.7, 14 dpl 24.9 ± 6.0, contralateral: Naïve 14.3 ± 4.4, 6 dpl 14.4 ± 2.0, 14 dpl 13.3 ± 1.9.

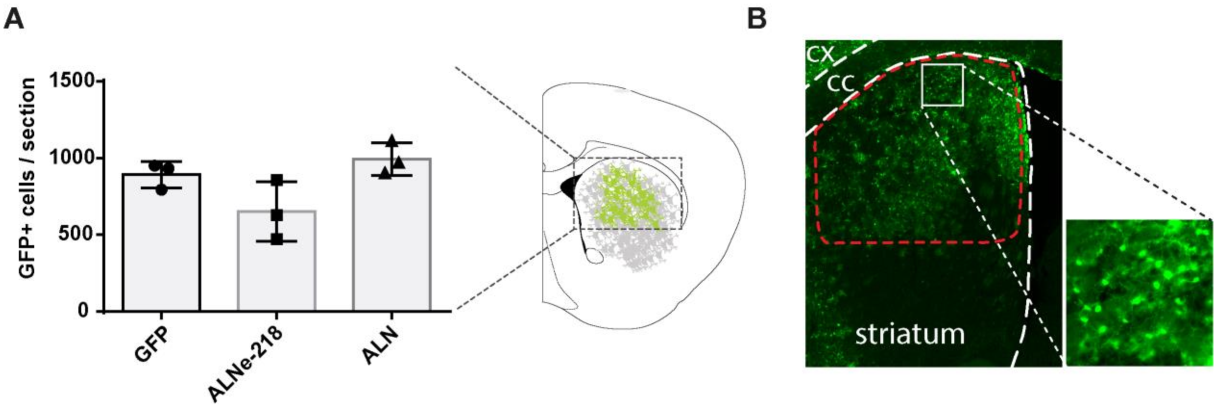


**Appendix Figure S4: Total amount and regional distribution of GFP$^+$ cells *in vivo* in *dCAM x Gfap-Cre* mice injected with FLEx-GFP reporter.**

**A,** GFP$^+$ cells in the ipsilateral dorsal striatum of one slide after five weeks of injection. No significant difference could be observed between the different reprogramming conditions and the GFP control. *GFP* 892.0 ± 85.4, *ALNe-218* 652.7 ± 193.6, *ALN* 993.3 ± 106.6. Error bars represent mean ± SD. **B,** Immunohistochemical staining of GFP positive cells 13 wpi with ALN illustrates the regional distribution of the infected and reprogrammed cells. Quantifications are performed in the dorsal striatum (red dashed line) excluding the subventricular zone. Abbreviations: CX – cortes, CC - corpus callosum.

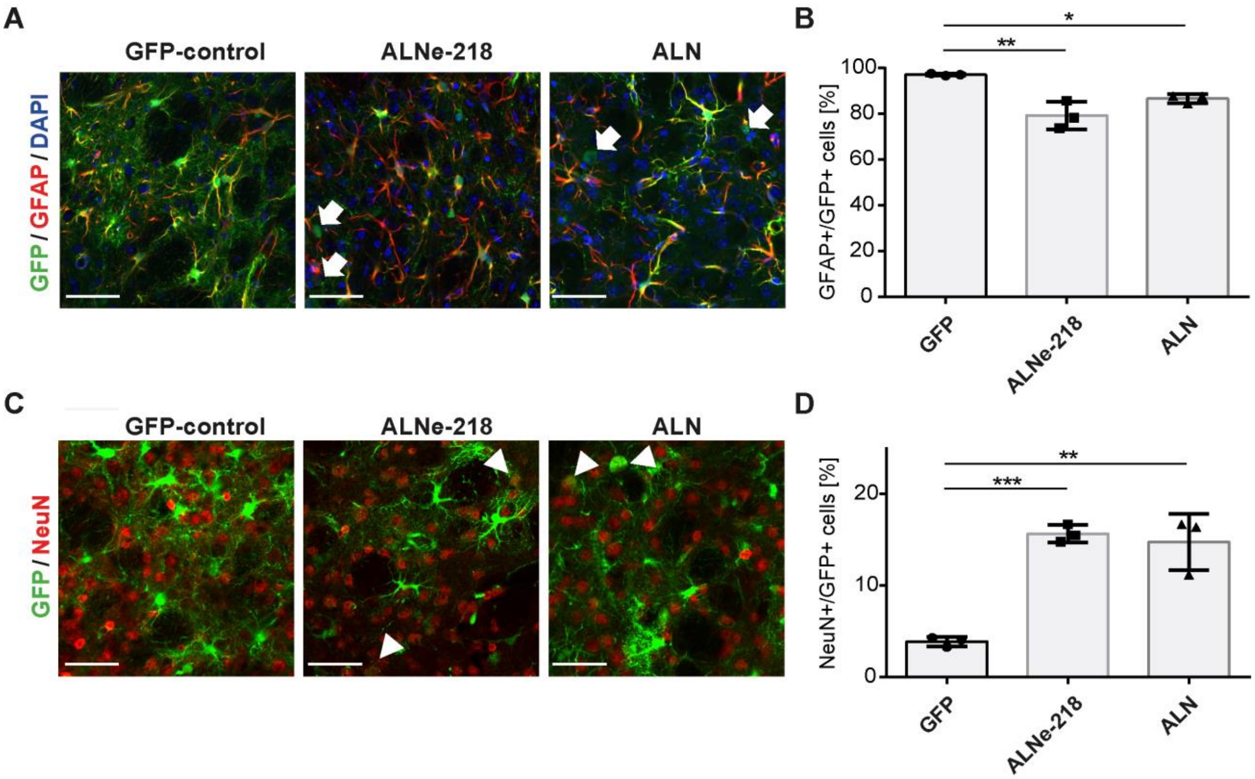


**Appendix Figure S5: *In vivo* reprogramming 5 weeks after AAV injection in dCAM x Gfap-Cre mice.**

**A,** Photomicrographs showing GFP$^+$/Gfap$^+$ double positive cells 5 wpi. Arrows indicating GFP$^+$/Gfap$^-$ cells. **B,** Quantification GFAP$^+$/GFP$^+$ cells. *GFP* 97.13 ± 0.45%, *ALNe-218* 79.33 ± 6.05 %, and *ALN* 86.70 ± 1.90%. *GFP* vs. *ALNe-218* P=0.0025, *GFP* vs. *ALN* P=0.03. Multiple comparison ANOVA F(2,6)=17.78. **C,** Photomicrographs showing GFP$^+$/NeuN$^+$ neurons 5 wpi. Arrow heads indicating GFP$^+$/NeuN$^+$ cells. **D,** Quantification NeuN$^+$/GFP$^+$ cells. *GFP* 3.9 ± 0.53%, *ALNe-218* 15.67 ± 0.96% and *ALN* 14.77 ± 3.09%. *GFP* vs. *ALNe-218* P=0.0007, *GFP* vs. *ALN* P=0.001. Multiple comparison ANOVA F(2,6)=35-85. Scale bar indicates 50 µm. Error bars represent mean ± SD. Tukey's multiple comparisons test * P<0.05, **P<0.01, ***P<0.001, ****P<0.0001.

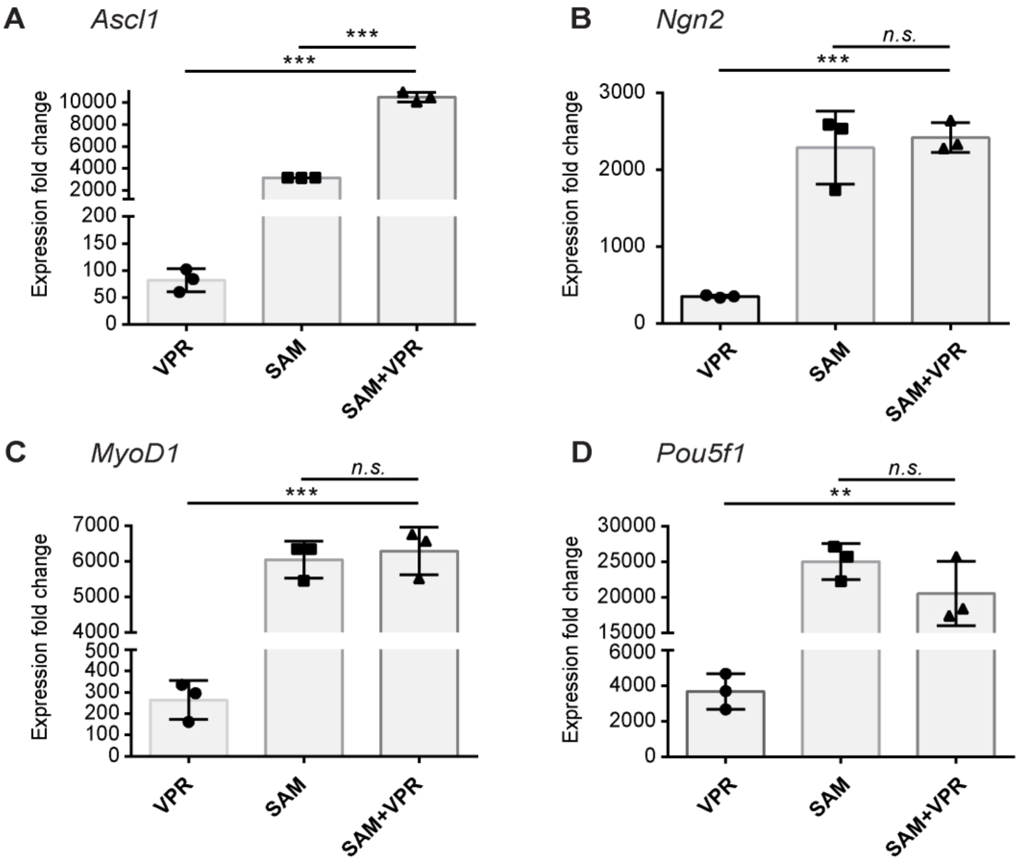


**Appendix Figure S6: Activation of endogenous genes using the dCas9-VPR and the SAM activator system in Neuro2A cells.**

**A,** Activation of *Ascl1.* (VPR 82 ± 21, SAM 3158 ± 10, SAM&VPR 10493 ± 432) **B,** Activation of *Ngn2.* (VPR 355 ± 17, SAM 2290 ± 476, SAM&VPR 2422 ± 195) **C,** Activation of *MyoD1.* (VPR 264 ± 91, SAM 6047 ± 517, SAM&VPR 6285 ± 669) **D,** Activation of *Pou5F1*. (VPR 3682 ± 1003, SAM 25041 ± 2507, SAM&VPR 20534 ± 4521). Each gene was activated by two sgRNAs targeting a region -200bp to 1bp upstream of the transcriptional start side. Activation levels are depicted as fold change between cells transfected with and without sgRNAs. All levels were normalized to β-Actin. n=1, with three technical replicates. Error bars represent mean ± SD between technical replicates.

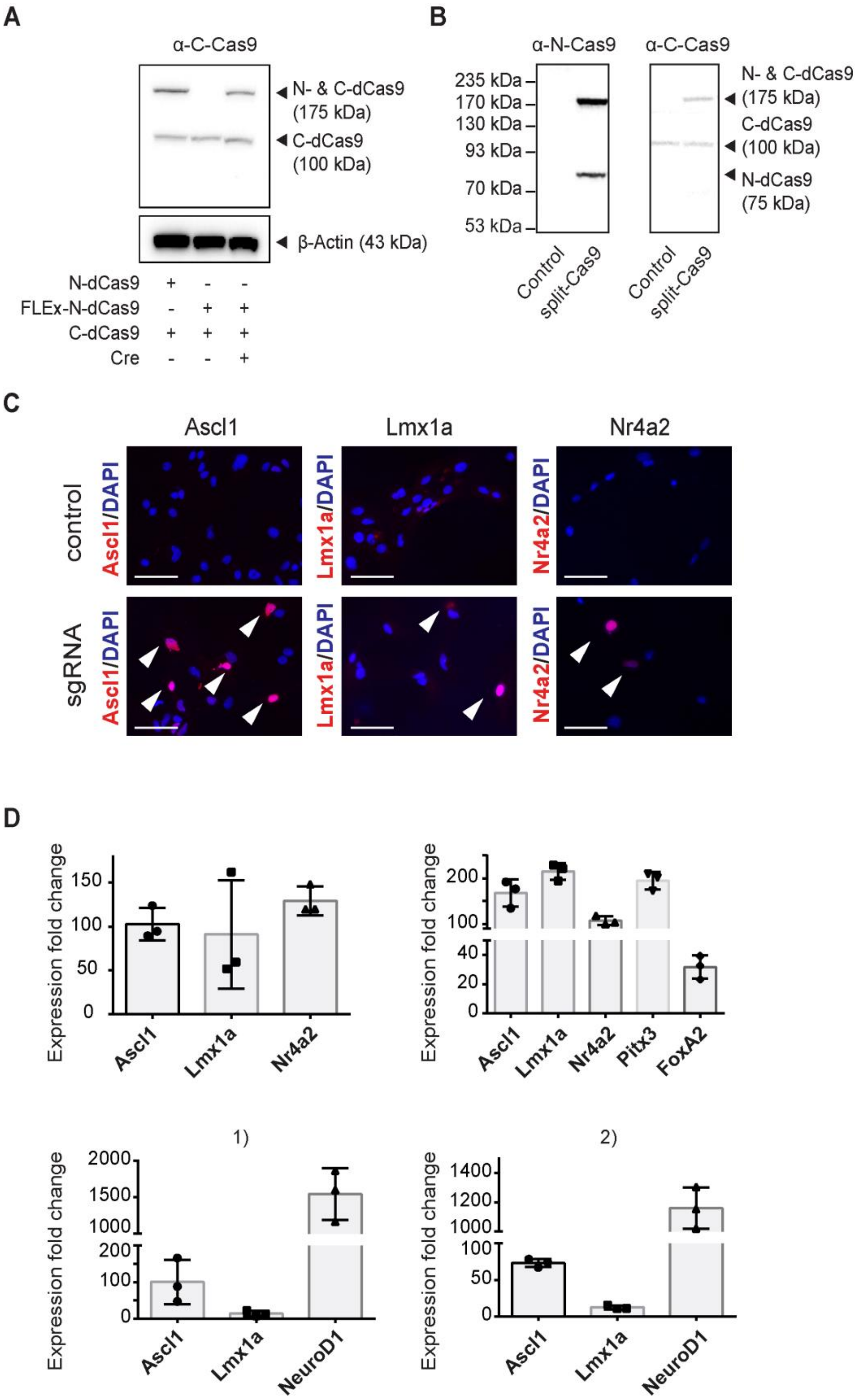

A
α-C-Cas9
N- & C-dCas9
(175 kDa)
C-dCas9
(100 kDa)
β-Actin (43 kDa)
N-dCas9 + - -
FLEx-N-dCas9 - + +
C-dCas9 + + +
Cre - - +
B
α-N-Cas9
α-C-Cas9
235 kDa
170 kDa
130 kDa
93 kDa
70 kDa
53 kDa
N- & C-dCas9
(175 kDa)
C-dCas9
(100 kDa)
N-dCas9
(75 kDa)
Control
split-Cas9
Control
split-Cas9
C
Ascl1
Lmx1a
Nr4a2
control
sgRNA
Ascl1/DAPI
Lmx1a/DAPI
Nr4a2/DAPI
D
Expression fold change
Ascl1
Lmx1a
Nr4a2
Pitx3
FoxA2
1)
2)
NeuroD1

**Appendix Figure S7: Evaluation of AAV-dCAS system *in vitro*.**

**A,** Western blot analysis evaluating the FLEx-N-dCas9 system in Neuro2A cells, using a C-Cas9 antibody. **B,** Western blot analysis evaluating the split-dCas9 system in Neuro2A cells, left blot – N-Cas9 antibody, right blot – C-Cas9 antibody. Correct fusion of the split-dCas9 parts at 175 kDa. **C,** Immunocytochemistry analysis on primary astrocytic cultures. Activation of *Ascl1*, *Lmx1a* and *Nr4a2*. Upper lane – Transfection of dCas9-activators without sgRNAs. Lower lane - Transfection of dCas9-activators with sgRNAs. Red channel staining for the respective protein. Scale bars indicate 10 µm. **D,** RT-qPCR levels. Multiplexed activation of *Ascl1*, *Lmx1a*, *Nr4a2* (*Ascl1* 103 ± 19, *Lmx1a* 91 ± 62, *Nr4a2* 129 ± 16) and *Ascl1, Lmx1a,* and *NeuroD1* (left (1): *Ascl1* 100 ± 61, *Lmx1a* 14 ± 7.5, *NeuroD1* 1542 ± 352, right (2): *Ascl1* 73 ± 6, *Lmx1a* 12 ± 3, *NeuroD1* 1160 ± 142) and *Ascl1*, *Lmx1a*, *Nr4a2, Pitx3, FoxA2* (Ascl1 167 ± 30, Lmx1a 215 ± 18, Nr4a2 107 ± 10, Pitx3 195 ± 19, FoxA2 32 ± 8) in primary astrocytic cultures. Activation levels are depicted as fold change between cells transfected with and without sgRNAs. All levels were normalized to β-Actin. Error bars represent mean ± SD between technical replicates.

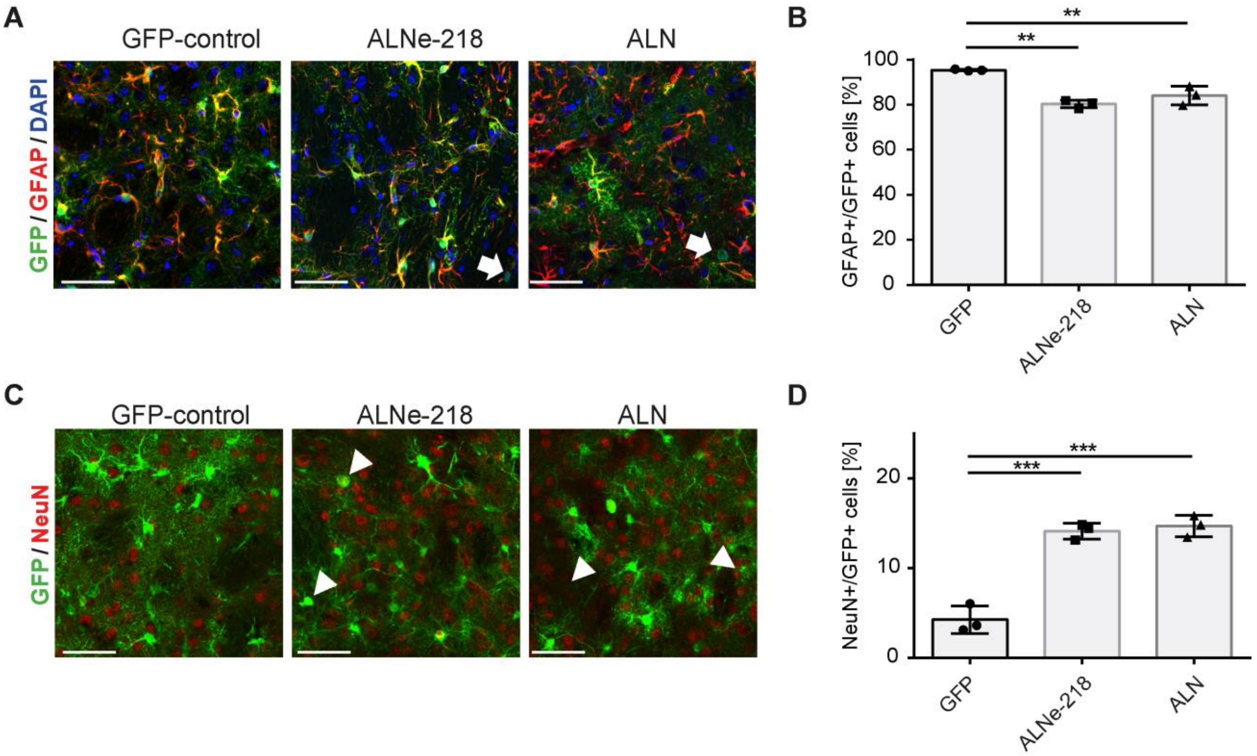


**Appendix Figure S8: *In vivo* reprogramming 5 wpi – AAV-dCAS reprogramming in Gfap-Cre mice.**

**A,** Photomicrographs showing GFP$^+$/GFAP$^+$ cells 5 wpi. Arrows indicating GFP$^+$/GFAP$^-$ cells. **B,** Quantification GFAP$^+$/GFP$^+$ cells. *GFP* 95.37 ± 0.40%, *ALNe-218* 80.33 ± 1.75% and *ALN* 84.10 ± 4.16%. *GFP* vs. *ALNe-218* P=0.001, *GFP* vs. *ALN* P=0.0045. Multiple comparison ANOVA F(2,6)=26.85. **C,** Photomicrographs showing GFP$^+$/NeuN$^+$ neurons 5 wpi. Arrow heads indicating GFP$^+$/NeuN$^+$ cells. **D,** Quantification NeuN$^+$/GFP$^+$ cells. *GFP* 4.23 ± 1.55%, *ALNe-218* 14.10 ± 0.89%, *ALN* 14.67 ± 1.21%. *GFP* vs. *ALNe-218* P=0.0002, *GFP* vs. *ALN* P=0.001. Multiple comparison ANOVA F(2,6)=66.67. Scale bar indicates 50 µm. Error bars represent mean ± SD. Tukey's multiple comparisons test * P<0.05, **P<0.01, ***P<0.001, ****P<0.0001.

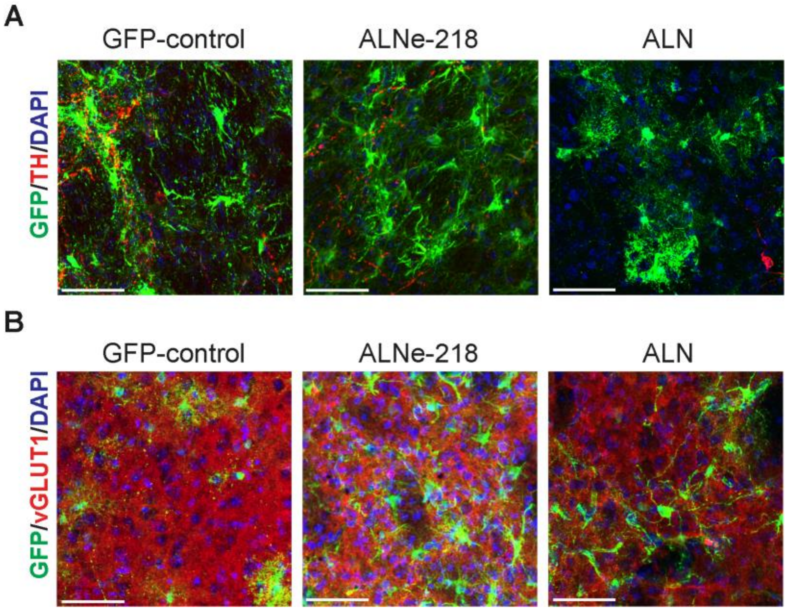


**Appendix Figure S9: Neurotransmitter identities of reprogrammed neurons using AAV-dCAS.**

**A, B,** Confocal images showing co-localization of GFP with specific markers for neurotransmitter subtypes. **A,** Tyrosine hydroxylase – dopaminergic neurons **B,** Vesicular glutamate transporter 1 – glutamatergic neurons. Scale bars indicate 50 µm.

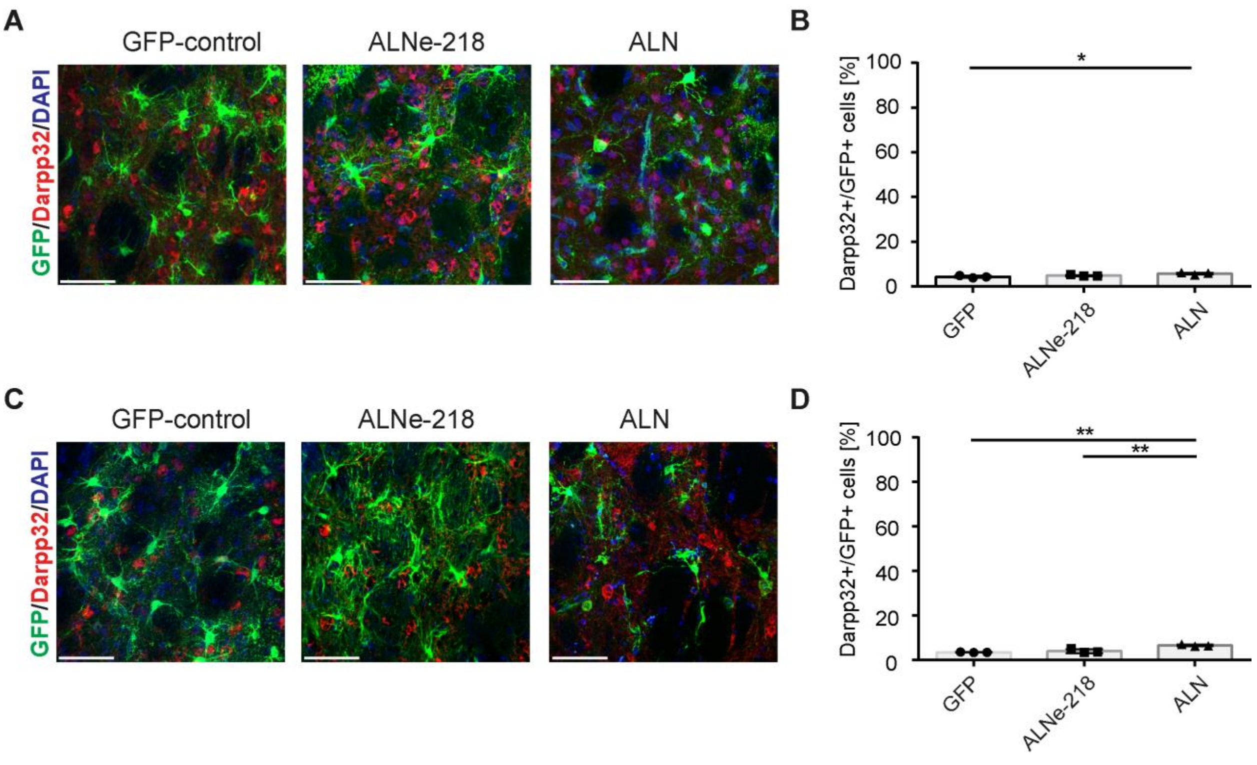


**Appendix Figure S10: Darpp32 staining and quantification 13 wpi.**

**A, B,** Evaluation Darpp32 staining in *dCAM* model. **A,** Confocal images showing co-localization of GFP and Darpp32. **B,** Quantification Darpp32$^{+}$/GFP$^{+}$ cells. *GFP* 4.3 ± 0.5%, *ALNe-218* 4.76 ± 0.38% and *ALN* 5.67 ± 0.49%. *GFP* vs. *ALN* P=0.023. Multiple comparison ANOVA F(2,6)=7.078. **C, D,** Evaluation Darpp32 staining in *AAV-dCAS* model. **C,** Confocal images showing co-localization of GFP and Darpp32. **D,** Quantification Darpp32$^{+}$/GFP$^{+}$ cells. *GFP* 3.4 ± 0.1%, *ALNe-218* 3.97 ± 0.99% and *ALN* 6.43 ± 0.42%. *GFP* vs. *ALN* P=0.0024, *ALN* vs. *ALNe-218* P=0.0067. Multiple comparison ANOVA F(2,6)=20.24. Scale bars indicate 50 µm. Error bars represent mean ± SD.

Tukey's multiple comparisons test * P<0.05, **P<0.01, ***P<0.001.

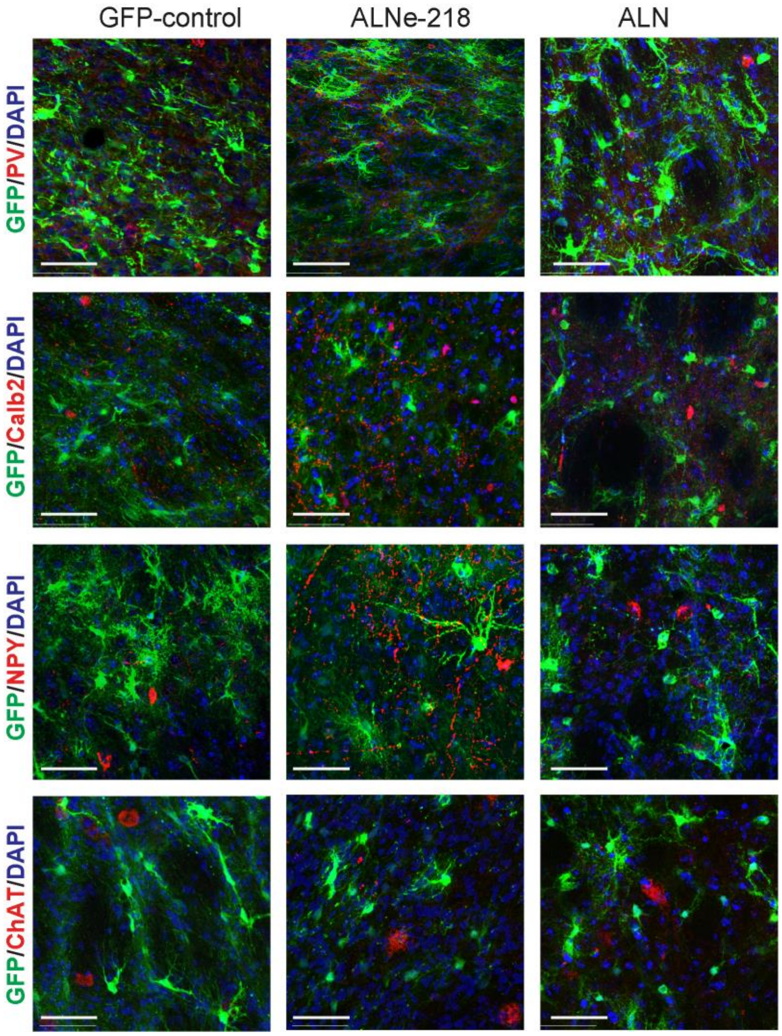


**Appendix Figure S11: Phenotypical characterization of AAV-dCAS reprogrammed neurons.**

Confocal images demonstrating the absence of several interneuron subtype markers in GFP+ cells: Parvalbumin (PV), Calretinin (Calb2), Neuropeptide Y (NPY) and Choline acetyl transferase (ChAT). Scale bars indicate 50 µm.

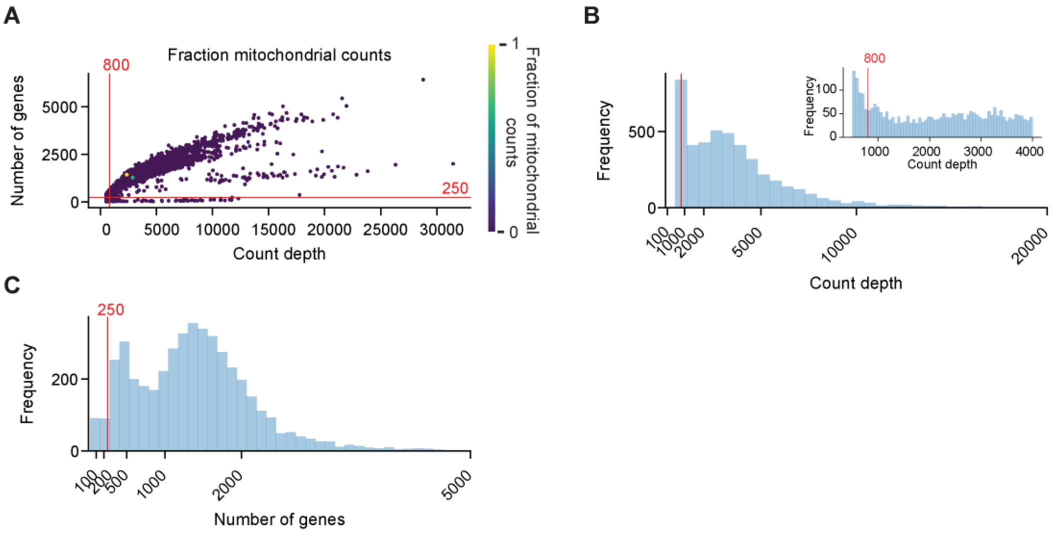


**Appendix Figure S12: Quality control of single cell RNA-seq at 13 wpi of AAVs in dCAM x GFAP-Cre mice striatal tissue.**

**A,** Number of genes (*y*-axis) versus count depth (*x*-axis) per cell. Color highlights fraction of mitochondrial reads. Quality control thresholds of 800 and 250 for number of genes and minimum cell depth are defined, respectively, obtaining 3,899 cells. **B,** Distributions of count depth for all cells. Inset shows count depth distribution from for all cells with fewer than 4000 counts. The count depth threshold of 800 is shown as a red, vertical line. **C,** Distribution of number of genes detected per cell. Red line indicates thresholds as in A.

A

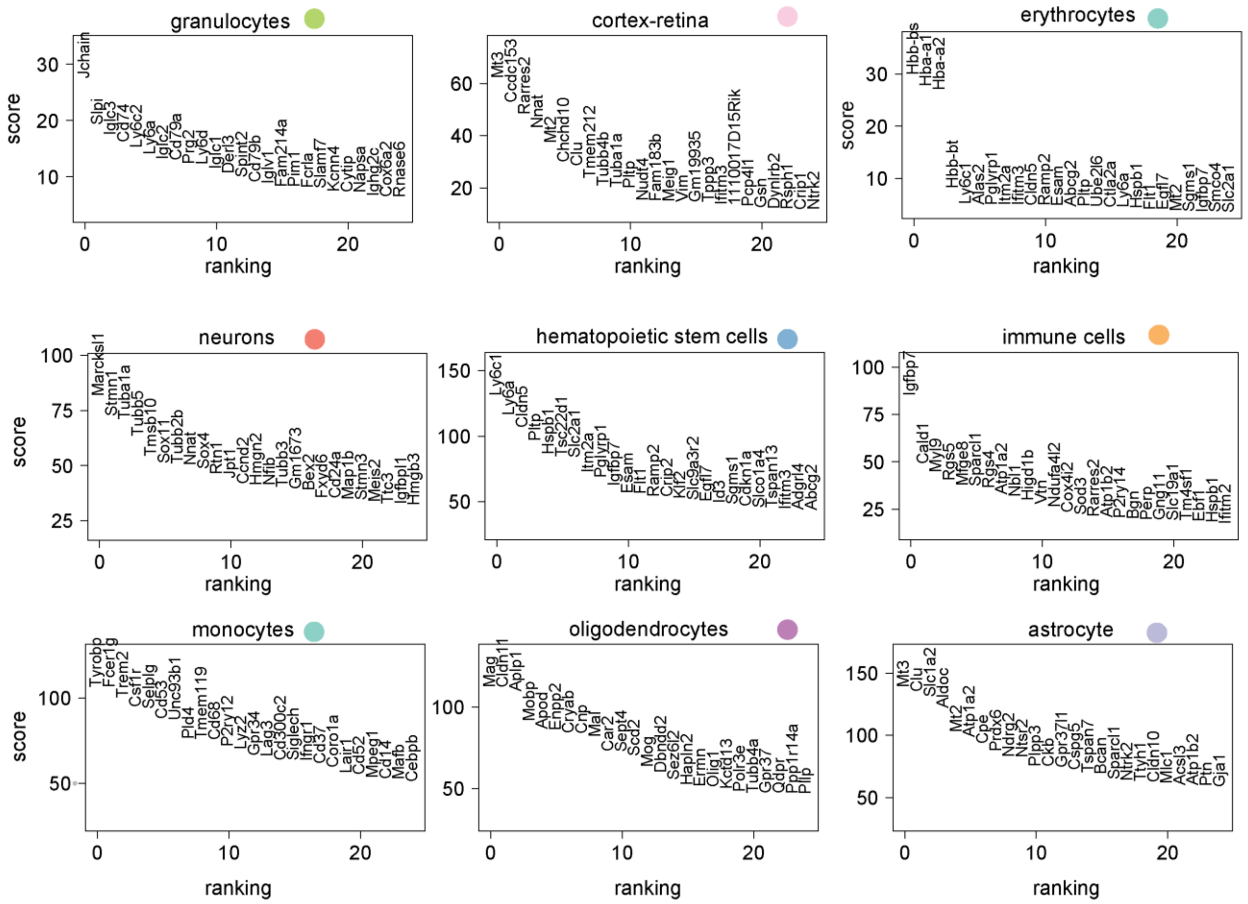
granulocytes
cortex-retina
erythrocytes
neurons
hematopoietic stem cells
immune cells
monocytes
oligodendrocytes
astrocyte
score
ranking

B

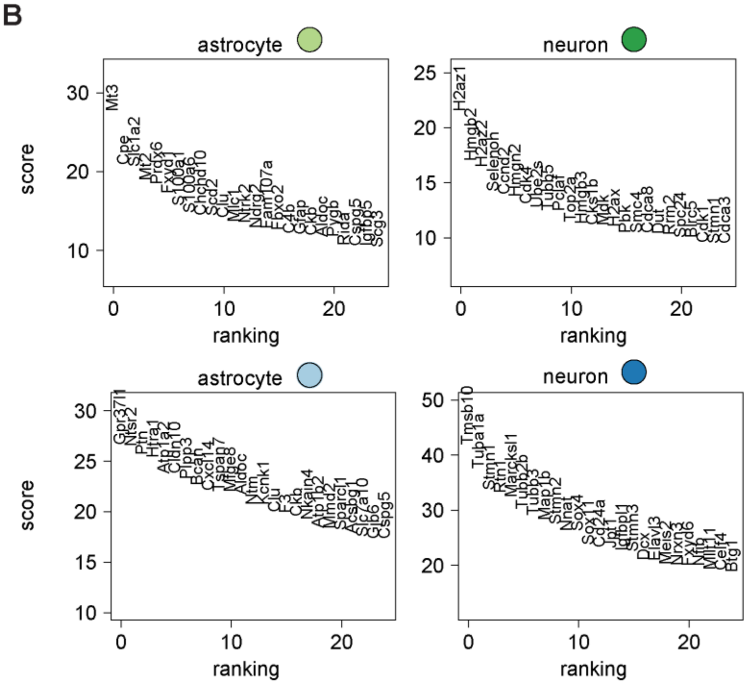
astrocyte
neuron
astrocyte
neuron
score
ranking

**Appendix Figure S13: Rank_genes_groups plots from Scanpy showing the top 25 marker genes using a t-test between log-normalized expression values.**

**A,** Top 25 marker genes ranked by their using *t*-test statistic when comparing normalized cell counts between annotated group against all other groups. **B,** Same as in A but using four astrocytic-neuronal subclusters (n=1,110 cells). Colors as in Figure 4 a, b.

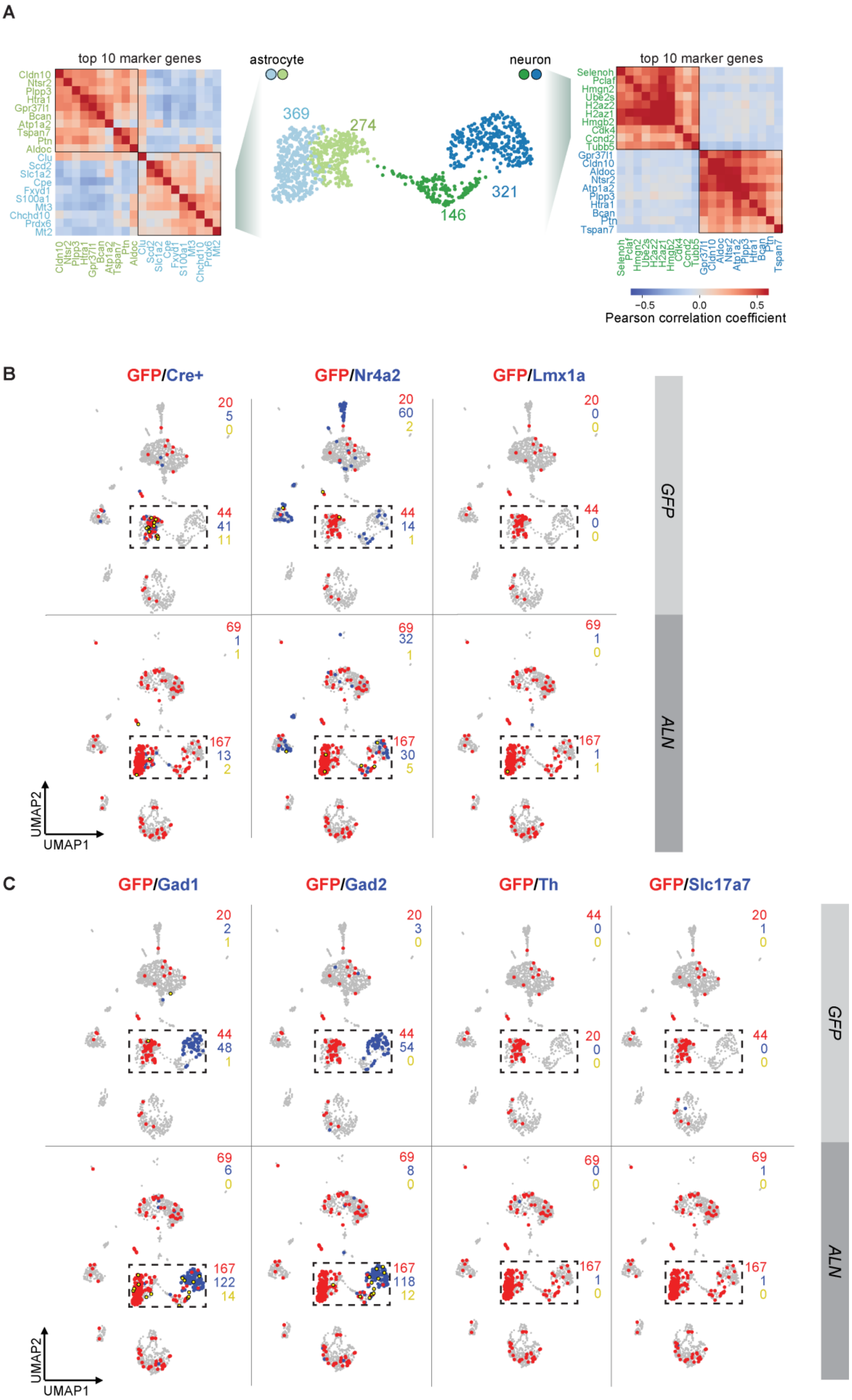
A
top 10 marker genes
astrocyte
neuron
top 10 marker genes
369
274
146
321
Cldn10
Ntsr2
Plpp3
Htra1
Gpr37l1
Bcan
Atp1a2
Tspan7
Ptn
Aldoc
Clu
Scd2
Slc1a2
Cpe
Fxyd1
S100a1
Mt3
Chchd10
Prdx6
Mt2
Selenoh
Pclaf
Hmgn2
Ube2s
H2az2
H2az1
Hmgb2
Cdk4
Ccnd2
Tubb5
−0.5
0.0
0.5
Pearson correlation coefficient
B
GFP/Cre+
GFP/Nr4a2
GFP/Lmx1a
GFP
ALN
UMAP2
UMAP1
C
GFP/Gad1
GFP/Gad2
GFP/Th
GFP/Slc17a7
GFP
ALN
UMAP2
UMAP1

**Appendix Figure S14: scRNA-seq analysis 13 wpi in dCAM x GFAP-Cre mice.**

**A,** Pearson´s correlation coefficient of top-10 marker gene expression levels of cells in astrocytic subgroups (n=643) and in neuronal cell subgroups (n=467). **B,** Counts for GFP$^+$ cells (red), markers *Cre*, *Nr4a2*, and *Lmx1a* (blue) and co-detection of cell with both markers (yellow) in *GFP control* and *ALN* reprogramming. **B,** Same as in B, but showing *Gad1*, *Gad2*, *Th* and *Slc17a7* as markers genes.

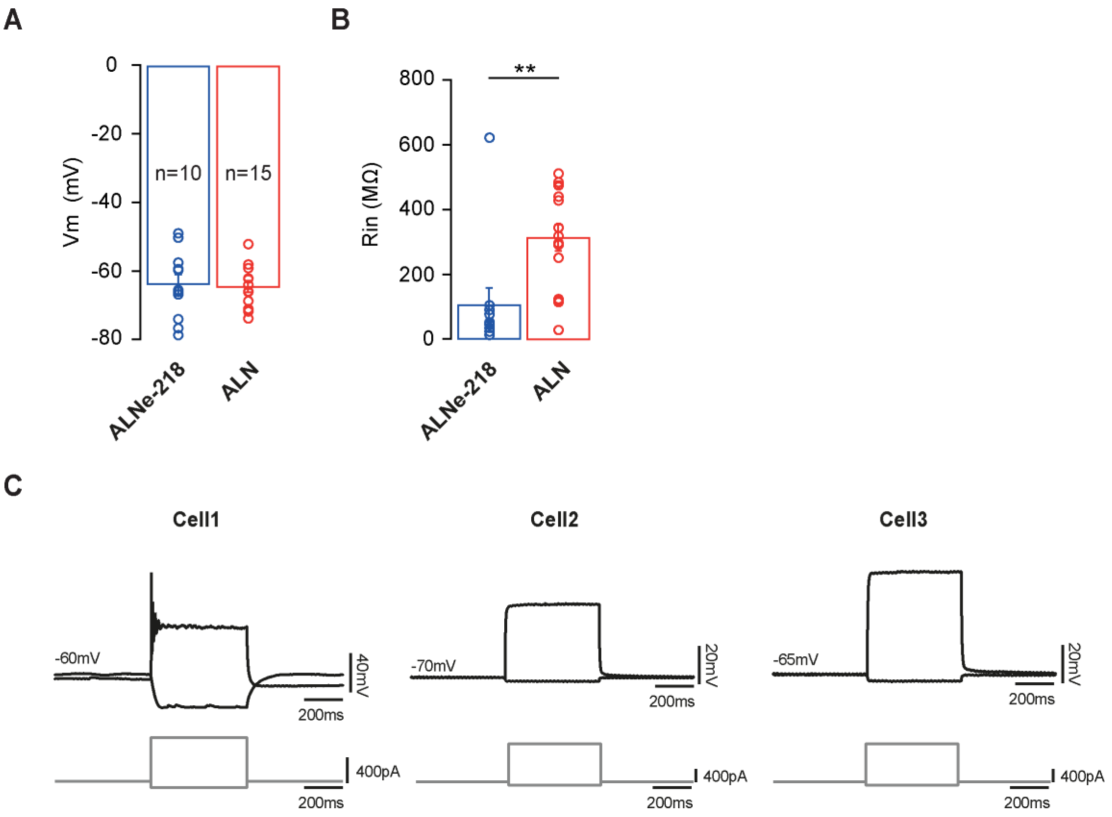


**Appendix Figure S15: Additional electrophysiological measurements 13 and 5 weeks after injection in the AAV-dCAS setting.**

**A,** The resting membrane potential ($V_m$ in mV) is similar between different reprogramming conditions. **B,** Input resistance ($R_{in}$ in mΩ) is significantly different between the different conditions (p=0.002, Kruskall-Wallis test). The input resistance of cells measured in the *ALNe-218* condition are similar to immature neurons/glia-like cells, whereas *ALN* reprogrammed cells exhibit an input resistance within the range of endogenous neurons. Kruskall-Wallis test **P<0.01. Error bars represent mean ± SEM. **C,** Firing pattern of induced neurons 5 weeks after *ALN* injection. Neurons exhibit electrophysiological properties of immature neurons (cell 1 exhibited one action potential) respectively of glial cells (cell 2 and 3).

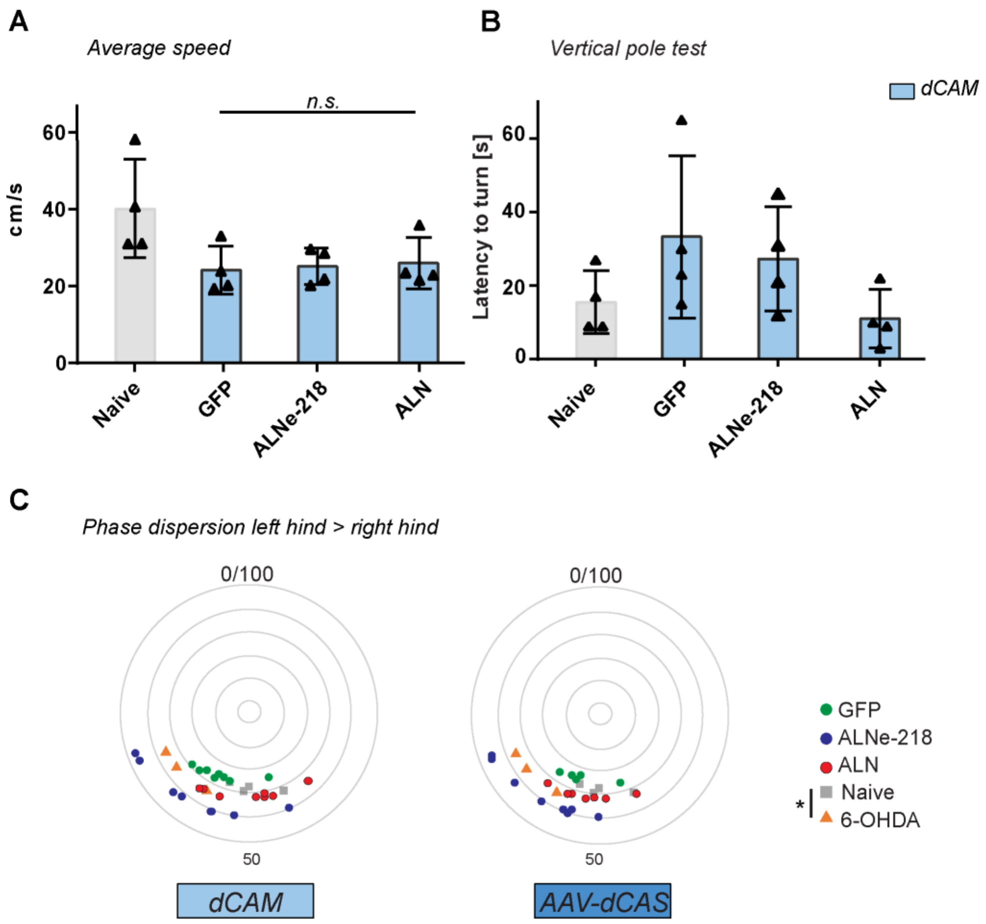


**Appendix Figure S16: Motor behavior analysis.**

**A,** Gait analysis using the CatWalk XT system. Average speed of tread. *dCAM* animals 5 wpi, n=4. Data in cm/s: Naïve 40.30 ± 12.78, *GFP* 24.19 ± 6.25, *ALNe-218* 25.14 ± 4.74, *ALN* 26.03 ± 6.69. **B,** Vertical pole test for *dCAM* animals. Latency time to turn, n=4. Data in s: Naïve 15.50 ± 8.54, *GFP* 33.25 ± 22.04, *ALNe-218* 27.25 ± 14.15, *ALN* 11.00 ± 7.96. *GFP* vs *ALN* P=0.17. **C,** Phase dispersion left hind paw to right hind paw (LH>RH). Controls: Grey square – naïve, orange triangle – 6-OHDA treated animals. Data in %: Naïve 52.42 ± 3.58, 6-OHDA 62.53 ± 4.08. Naïve vs. 6-OHDA P=0.0174. Treatments: Green – *GFP*, blue – *ALNe-218*, red – *ALN*. *dCAM*: *GFP* 54.47 ± 3.66, *ALNe-218* 56.84 ± 7.28, *ALN* 49.82 ± 5.14. *ALN* vs. *ALNe-218* P=0.0549, multiple comparison ANOVA F(2,20)=3.250. dCAS: *GFP* 53.77 ± 3.56, *ALNe-218* 58.04 ± 5.42, *ALN* 52.38 ± 3.84. *ALN* vs. *ALNe-218* P=0.0653, multiple comparison ANOVA F(2,17)=3.193. Error bars represent mean ± SD.

A

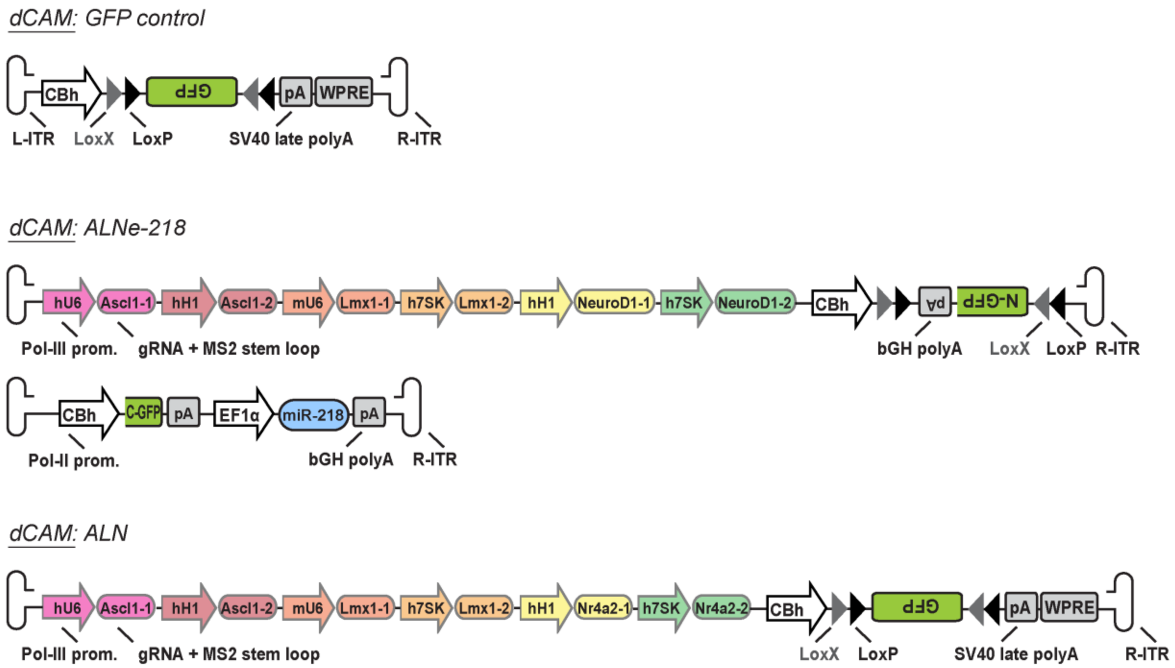
dCAM: GFP control
CBh
GFP
pA
WPRE
L-ITR
LoxX
LoxP
SV40 late polyA
R-ITR
dCAM: ALNe-218
hU6
Ascl1-1
hH1
Ascl1-2
mU6
Lmx1-1
h7SK
Lmx1-2
hH1
NeuroD1-1
h7SK
NeuroD1-2
CBh
pA
N-GFP
Pol-III prom.
gRNA + MS2 stem loop
bGH polyA
LoxX
LoxP
R-ITR
CBh
C-GFP
pA
EF1α
miR-218
pA
Pol-II prom.
bGH polyA
R-ITR
dCAM: ALN
hU6
Ascl1-1
hH1
Ascl1-2
mU6
Lmx1-1
h7SK
Lmx1-2
hH1
Nr4a2-1
h7SK
Nr4a2-2
CBh
GFP
pA
WPRE
Pol-III prom.
gRNA + MS2 stem loop
LoxX
LoxP
SV40 late polyA
R-ITR

B

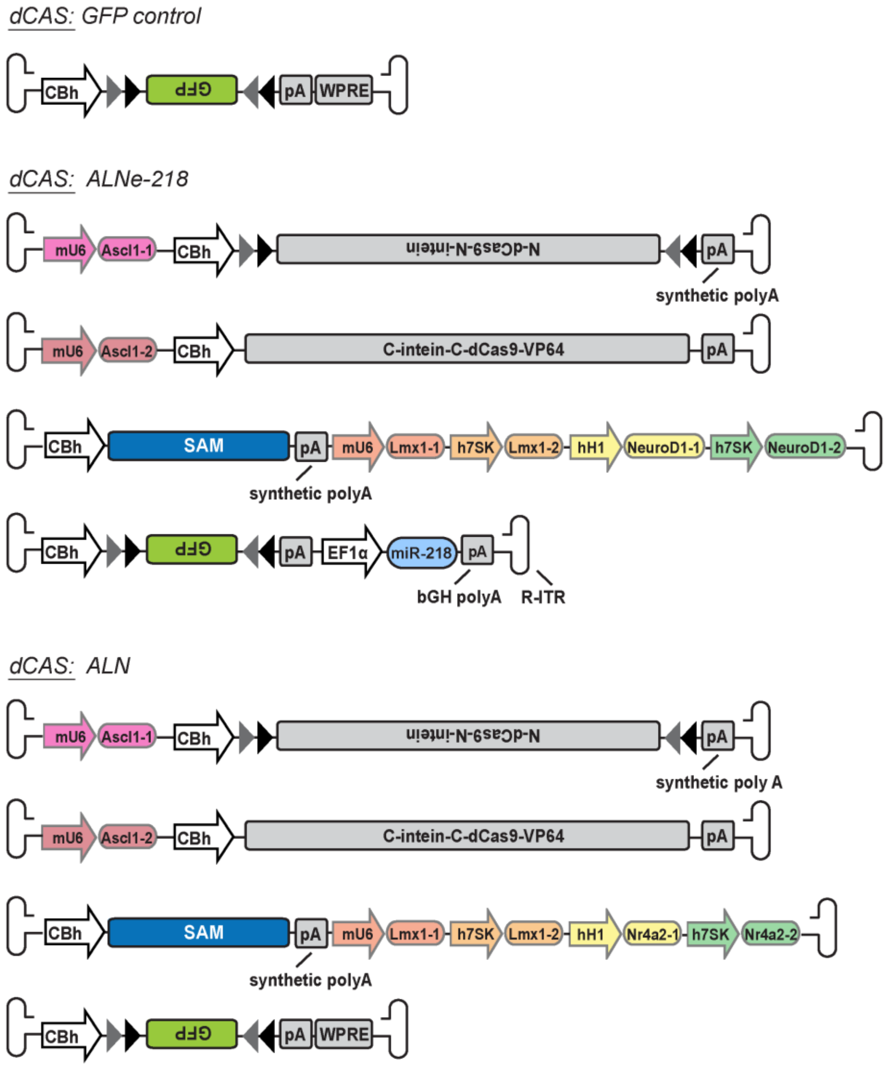
dCAS: GFP control
CBh
GFP
pA
WPRE
dCAS: ALNe-218
mU6
Ascl1-1
CBh
N-dCas9-N-intein
pA
synthetic polyA
mU6
Ascl1-2
CBh
C-intein-C-dCas9-VP64
pA
CBh
SAM
pA
mU6
Lmx1-1
h7SK
Lmx1-2
hH1
NeuroD1-1
h7SK
NeuroD1-2
synthetic polyA
CBh
GFP
pA
EF1α
miR-218
pA
bGH polyA
R-ITR
dCAS: ALN
mU6
Ascl1-1
CBh
N-dCas9-N-intein
pA
synthetic poly A
mU6
Ascl1-2
CBh
C-intein-C-dCas9-VP64
pA
CBh
SAM
pA
mU6
Lmx1-1
h7SK
Lmx1-2
hH1
Nr4a2-1
h7SK
Nr4a2-2
synthetic polyA
CBh
GFP
pA
WPRE

**Appendix Figure S17: AAV combinations.**

A representation of the AAV combinations, which were used for the different approaches and experimental groups with detailed information to promoter and gRNA position and regulatory elements. **A,** Combinations used for the dCas9 activator mouse experiments. **B,** Combinations used for the adeno-associated virus (AAV)-based intein-split-dCas9 activator system (AAV-dCAS).

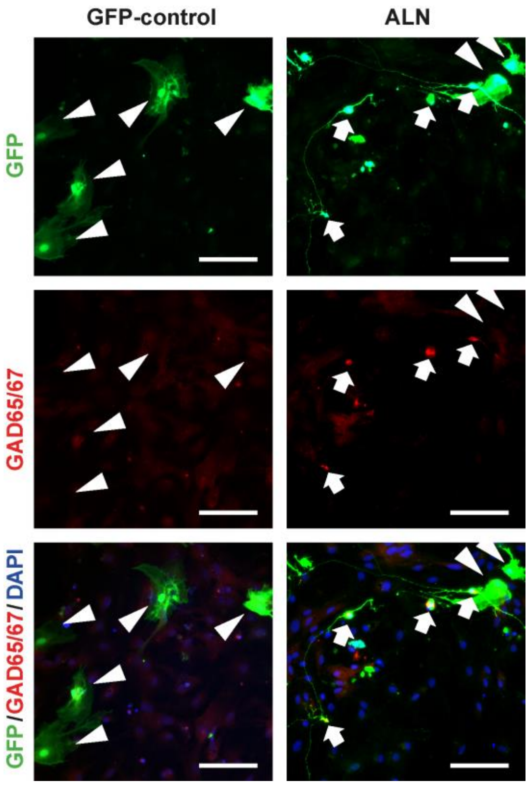


**Appendix Figure S18: dCAS reprogramming of primary astrocytes.**

Reprogramming primary astrocytes by dCAS-mediated induction of Ascl1, Lmx1a and Nr4a2 results in Gad65/67-positive neurons: Immunocytochemical analysis showing GFP+/GAD6567+ double positive cells 3 weeks after transduction of primary astrocytes. Arrows indicate double positive GFP+/GAD6567+ cells, arrowheads indicate GFP+/GAD6567- cells. Primary astrocytes, (after 8 days in culture) were transfected using LTX+ reagent with AAV plasmids in an equimolar ratio (total 500ng). GFP control contains the non-flexed version of the CBh driven GFP control plasmid. ALN reprogrammed cells were transfected with the non-flexed versions of the AAV-dCas9 ALN combination as indicated in Appendix Figure S17. Cells were fixed and 21 days after transfection. Scale bars indicate 100 µm.

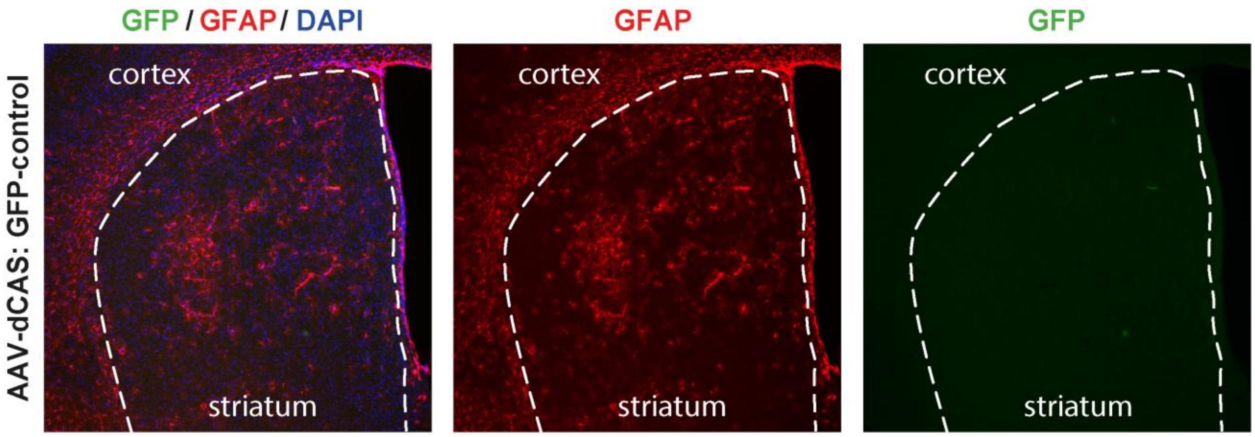


**Appendix Figure S19: AAV-dCAS injection into Gfap-Cre negative mice.**

Immunohistochemical staining for GFAP and GFP in Gfap-Cre negative mice 13 weeks after injection of the AAV-dCAS GFP-control virus. Absence of any GFP expression indicates the functionality of the FLEx (Cre-ON) reporter system.

| gRNA | Gene | Sequence 5´- 3´ |
|---|---|---|
| Ascl1-1 | Ascl1 | GGGAGCCGCTCGCTGCAGCAGCG |
| Ascl1-2 | Ascl1 | GGGGCTGAATGGAGAGTTTGCA |
| Lmx1a-1 | Lmx1a | GGGAGCAAAGGAGTCGCCTTG |
| Lmx1a-2 | Lmx1a | GAATGCATCCAAGAGTGAACC |
| Nr4a2-1 | Nr4a2 | GGCGGTGGGTCATTGTTTCCG |
| Nr4a2-2 | Nr4a2 | GTGCCAGTGACGCCGGCCTGG |
| NeuroD1-1 | NeuroD1 | GGTTCTGGGAGGGGTGAATGA |
| NeuroD1-2 | NeuroD1 | GGCCATATGGCGCATGCCGGGG |
| Neurog2-1 | Ngn2 | ATAAGCTGGGGAGGGAGAGC |
| Neurog2-2 | Ngn2 | AAACAATCAGATCTGCCCCG |
| Pitx3-1 | Pitx3 | ATTCACTTTATGGCAACCCA |
| Pitx3-2 | Pitx3 | GCTAGCCTGGGAGAGCCCAG |
| FoxA2-1 | FoxA2 | GAAAGTAACCTTGAAACACCG |
| FoxA2-2 | FoxA2 | GGGTAGCCAGAAAGAGGACTG |
| MyoD1-1 | MyoD1 | CCAATAGGAGTGTAGTAGGG |
| MyoD1-2 | MyoD1 | GAGAGACTGGCAGCCATACG |
| Pou5f1-1 | Pou5f1 | ATCTGCCTGTGTCTTCCAGA |
| Pou5f1-2 | Pou5f1 | TGTCCGGTGACCCAAGGCAG |

**Appendix Table S1: Sequences of gRNAs used in this study**

| Gene | TaqMan probe (Assay ID) |
|---|---|
| Ascl1/Mash1 | Mm03058063_m1 |
| Lmx1a | Mm00473947_m1 |
| Nr4a2/NR4A2 | Mm00443060_m1 |
| NeuroD1 | Mm01280117_m1 |
| Ngn2 | Mm00437603_g1 |
| Pitx3 | Mm01194166_g1 |
| FoxA2 | Mm00839704_mH |
| MyoD1 | Mm00440387_m1 |
| Pou5f1 | Mm00658129_gH |
| B-Actin | Mm00607939_s1 |

**Appendix Table S2: TaqMan probes used in this study**

**Sequence data:**

```
>dCas9-SAM-P2A-VPR
ctagggcgcgccggattcgacattgattattgactagttattaatagtaatcaattacggggtcattagt
tcatagcccatatatggagttccgcgttacataacttacggtaaatggcccgcctggctgaccgcccaac
gacccccgcccattgacgtcaataatgacgtatgttcccatagtaacgccaatagggactttccattgac
gtcaatgggtggagtatttacggtaaactgcccacttggcagtacatcaagtgtatcatatgccaagtac
gccccctattgacgtcaatgacggtaaatggcccgcctggcattatgcccagtacatgaccttatgggac
tttcctacttggcagtacatctacgtattagtcatcgctattaccatggtcgaggtgagccccacgttct
gcttcactctccccatctcccccccctccccacccccaattttgtatttatttattttttaattattttg
tgcagcgatgggggcgggggggggggggggcgcgcgccaggcggggcggggcggggcgaggggcggggcgg
ggcgaggcggagaggtgcggcggcagccaatcagagcggcgcgctccgaaagtttccttttatggcgagg
cggcggcggcggcggccctataaaaagcgaagcgcgcggcgggcgggagtcgctgcgttgccttcgcccc
gtgccccgctccgcgccgcctcgcgccgcccgccccggctctgactgaccgcgttactcccacaggtgag
cgggcgggacggcccttctcctccgggctgtaattagcgcttggtttaatgacggctcgtttcttttctg
tggctgcgtgaaagccttaaagggctccgggagggccctttgtgcgggggggagcggctcgggggtgcg
tgcgtgtgtgtgtgcgtggggagcgccgcgtgcggcccgcgctgcccggcggctgtgagcgctgcgggcg
cggcgcggggctttgtgcgctccgcgtgtgcgcgaggggagcgcggccgggggcggtgccccgcggtgcg
gggggctgcgaggggaacaaaggctgcgtgcggggtgtgtgcgtgggggggtgagcagggggtgtgggc
gcggcggtcgggctgtaacccccccctgcacccccctccccgagttgctgagcacggcccggcttcgggt
gcggggctccgtacggggcgtggcgcggggctcgccgtgccgggcggggggtggcggcaggtgggggtgc
cgggcggggcggggccgcctcggccggggagggctcgggggaggggcgcggcggcccccggagcgccggc
ggctgtcgaggcgcggcgagccgcagccattgccttttatggtaatcgtgcgagagggcgcagggacttc
ctttgtcccaaatctggcggagccgaaatctgggaggcgccgccgcaccccctctagcgggcgcgggcga
agcggtgcggcgccggcaggaaggaaatgggcggggagggccttcgtgcgtcgccgcgccgccgtcccct
tctccatctccagcctcggggctgccgcagggggacggctgccttcgggggggacggggcagggcggggt
tcggcttctggcgtgtgaccggcggctctagagcctctgctaaccatgttcatgccttcttctttttcct
acagatccttaattaaccgtcttaagcctgtaacaaccggtacagttcgaataacttcgtatagcataca
ttatacgaagttataagcttgcatgcctgcaggtcggccgccacgaccggccggtgccgccaccatcccc
```

tgacccacgcccctgacccctcacaaggagacgaccttccatgaccgagtacaagcccacggtgcgcctc
gccacccgcgacgacgtccccgggccgtacgcaccctcgccgccgcgttcgccgactaccccgccacgc
gccacaccgtcgacccggaccgccacatcgagcgggtcaccgagctgcaagaactcttcctcacgcgcgt
cgggctcgacatcggcaaggtgtgggtcgcggacgacggcgccgcggtggcggtctggaccacgccggag
agcgtcgaagcgggggcggtgttcgccgagatcggcccgcgcatggccgagttgagcggttcccggctgg
ccgcgcagcaacagatggaaggcctcctggcgccgcaccggcccaaggagcccgcgtggttcctggccac
cgtcggcgtctcgcccgaccaccagggcaagggtctgggcagcgccgtcgtgctccccggagtggaggcg
gccgagcgcgccggggtgcccgccttcctggagacctccgcgccccgcaacctccccttctacgagcggc
tcggcttcaccgtcaccgccgacgtcgaggtgcccgaaggaccgcgcacctggtgcatgacccgcaagcc
cggtgcctgacgcccgccccacgacccgcagcgcccgaccgaaaggagcgcacgaccccatggctccgac
cgaagccacccggggcggccccgccgaccccgcacccgcccccgaggcccaccgactctagaggatcata
atcagccataccacatttgtagaggttttacttgctttaaaaaacctcccacacctccccctgaacctga
aacataaaatgaatgcaattgttgttgttaacttgtttattgcagcttataatggttacaaataaagcaa
tagcatcacaaatttcacaaataaagcatttttttcactgcattctagttgtggtttgtccaaactcatc
aatgtatcttatcatgtctggatccataacttcgtatagcatacattatacgaagttataccggccacc
atagtcgcgagtagcttgggccagctaggccttgaccaaagttcctctggaattgcgatcgcgaagttcc
tattctctagaaagtataggaacttcgtgccgccaccatggactacaaggacgacgacgataaggcttca
aactttactcagttcgtgctcgtggacaatggtgggacaggggatgtgacagtggctccttctaatttcg
ctaatggggtggcagagtggatcagctccaactcacggagccaggcctacaaggtgacatgcagcgtcag
gcagtctagtgcccagaagagaaagtataccatcaaggtggaggtccccaaagtggctacccagacagtg
ggcggagtcgaactgcctgtcgccgcttggaggtcctacctgaacatggagctcactatcccaattttcg
ctaccaattctgactgtgaactcatcgtgaaggcaatgcaggggctcctcaaagacggtaatcctatccc
ttccgccatcgccgctaactcaggtatctacagcgctggaggaggtggaagcggaggaggaggaagcgga
ggaggaggtagcggacctaagaaaaagaggaaggtggcggccgctggatccccttcagggcagatcagca
accaggccctggctctggcccctagctccgctccagtgctggcccagactatggtgccctctagtgctat
ggtgcctctggcccagccacctgctccagcccctgtgctgaccccaggaccaccccagtcactgagcgct
ccagtgcccaagtctacacaggccggcgaggggactctgagtgaagctctgctgcacctgcagttcgacg
ctgatgaggacctgggagctctgctggggaacagcaccgatcccggagtgttcacagatctggcctccgt
ggacaactctgagtttcagcagctgctgaatcagggcgtgtccatgtctcatagtacagccgaaccaatg
ctgatggagtaccccgaagccattacccggctggtgaccggcagccagcggcccccgaccccgctccaa
ctcccctgggaaccagcggcctgcctaatggctgtccggagatgaagacttctcaagcatcgctgatat
ggactttagtgccctgctgtcacagatttcctctagtgggcagggaggaggtggaagcggcttcagcgtg
gacaccagtgccctgctggacctgttcagcccctcggtgaccgtgcccgacatgagcctgcctgaccttg
acagcagcctggccagtatccaagagctcctgtctccccaggagcccccaggcctcccgaggcagagaa
cagcagcccggattcagggaagcagctggtgcactacacagcgcagccgctgttcctgctggaccccggc
tccgtggacaccgggagcaacgacctgccggtgctgtttgagctgggagagggctcctacttctccgaag
gggacggcttcgccgaggaccccaccatctccctgctgacaggctcggagcctcccaaagccaaggaccc
cactgtctcccgaagttcctattctctagaaagtataggaacttcgggcagcggcgccaccaacttcagc
ctgctgaaacaggctggcgacgtcgaggaaaatcccgggccatgtacaatgtacccatacgatgttccag
attacgcttcgccgaagaaaaagcgcaaggtcgaagcgtccgacaagaagtacagcatcggcctggccat
cggcaccaactctgtgggctgggccgtgatcaccgacgagtacaaggtgcccagcaagaaattcaaggtg
ctgggcaacaccgaccggcacagcatcaagaagaacctgatcggagccctgctgttcgacagcggcgaaa
cagccgaggccacccggctgaagagaaccgccagaagaagatacaccagacggaagaaccggatctgcta
tctgcaagagatcttcagcaacgagatggccaaggtggacgacagcttcttccacagactggaagagtcc
ttcctggtggaagaggataagaagcacgagcggcaccccatcttcggcaacatcgtggacgaggtggcct
accacgagaagtaccccaccatctaccacctgagaaagaaactggtggacagcaccgacaaggccgacct
gcggctgatctatctggccctggcccacatgatcaagttccggggccacttcctgatcgagggcgacctg
aaccccgacaacagcgacgtggacaagctgttcatccagctggtgcagacctacaaccagctgttcgagg
aaaaccccatcaacgccagcggcgtggacgccaaggccatcctgtctgccagactgagcaagagcagacg
gctggaaaatctgatcgcccagctgcccggcgagaagaagaatggcctgttcggcaacctgattgccctg
agcctgggcctgacccccaacttcaagagcaacttcgacctggccgaggatgccaaactgcagctgagca
aggacacctacgacgacgacctggacaacctgctggcccagatcggcgaccagtacgccgacctgtttct
ggccgccaagaacctgtccgacgccatcctgctgagcgacatcctgagagtgaacaccgagatcaccaag

gccccccctgagcgcctctatgatcaagagatacgacgagcaccaccaggacctgaccctgctgaaagctc
tcgtgcggcagcagctgcctgagaagtacaaagagattttcttcgaccagagcaagaacggctacgccgg
ctacattgacggcggagccagccaggaagagttctacaagttcatcaagcccatcctggaaaagatggac
ggcaccgaggaactgctcgtgaagctgaacagagaggacctgctgcggaagcagcggaccttcgacaacg
gcagcatcccccaccagatccacctgggagagctgcacgccattctgcggcggcaggaagattttaccc
attcctgaaggacaaccgggaaaagatcgagaagatcctgaccttccgcatcccctactacgtgggccct
ctggccaggggaaacagcagattcgcctggatgaccagaaagagcgaggaaaccatcacccccctggaact
tcgaggaagtggtggacaagggcgcttccgcccagagcttcatcgagcggatgaccaacttcgataagaa
cctgcccaacgagaaggtgctgcccaagcacagcctgctgtacgagtacttcaccgtgtataacgagctg
accaaagtgaaatacgtgaccgagggaatgagaaagcccgccttcctgagcggcgagcagaaaaaggcca
tcgtggacctgctgttcaagaccaaccggaaagtgaccgtgaagcagctgaaagaggactacttcaagaa
aatcgagtgcttcgactccgtggaaatctccggcgtggaagatcggttcaacgcctccctgggcacatac
cacgatctgctgaaaattatcaaggacaaggacttcctggacaatgaggaaaacgaggacattctggaag
atatcgtgctgaccctgacactgtttgaggacagagagatgatcgaggaacggctgaaaacctatgccca
cctgttcgacgacaaagtgatgaagcagctgaagcggcggagatacaccggctggggcaggctgagccgg
aagctgatcaacggcatccgggacaagcagtccggcaagacaatcctggatttcctgaagtccgacggct
tcgccaacagaaacttcatgcagctgatccacgacgacagcctgacctttaaagaggacatccagaaagc
ccaggtgtccggccagggcgatagcctgcacgagcacattgccaatctggccggcagccccgccattaag
aagggcatcctgcagacagtgaaggtggtggacgagctcgtgaaagtgatgggccggcacaagcccgaga
acatcgtgatcgaaatggccagagagaaccagaccacccagaagggacagaagaacagccgcgagagaat
gaagcggatcgaagagggcatcaaagagctgggcagccagatcctgaaagaacaccccgtggaaaacacc
cagctgcagaacgagaagctgtacctgtactacctgcagaatgggcgggatatgtacgtggaccaggaac
tggacatcaaccggctgtccgactacgatgtggacgccatcgtgcctcagagctttctgaaggacgactc
catcgacaacaaggtgctgaccagaagcgacaagaaccggggcaagagcgacaacgtgccctccgaagag
gtcgtgaagaagatgaagaactactggcggcagctgctgaacgccaagctgattacccagagaaagttcg
acaatctgaccaaggccgagagaggcggcctgagcgaactggataaggccggcttcatcaagagacagct
ggtggaaacccggcagatcacaaagcacgtggcacagatcctggactcccggatgaacactaagtacgac
gagaatgacaagctgatccgggaagtgaaagtgatcaccctgaagtccaagctggtgtccgatttccgga
aggatttccagttttacaaagtgcgcgagatcaacaactaccaccacgcccacgacgcctacctgaacgc
cgtcgtgggaaccgccctgatcaaaaagtaccctaagctggaaagcgagttcgtgtacggcgactacaag
gtgtacgacgtgcggaagatgatcgccaagagcgagcaggaaatcggcaaggctaccgccaagtacttct
tctacagcaacatcatgaactttttcaagaccgagattaccctggccaacggcgagatccggaagcggcc
tctgatcgagacaaacggcgaaaccggggagatcgtgtgggataagggccgggattttgccaccgtgcgg
aaagtgctgagcatgccccaagtgaatatcgtgaaaaagaccgaggtgcagacaggcggcttcagcaaag
agtctatcctgcccaagaggaacagcgataagctgatcgccagaaagaaggactgggaccctaagaagta
cggcggcttcgacagccccaccgtggcctattctgtgctggtggtggccaaagtggaaaagggcaagtcc
aagaaactgaagagtgtgaaagagctgctggggatcaccatcatggaaagaagcagcttcgagaagaatc
ccatcgactttctggaagccaagggctacaaagaagtgaaaaaggacctgatcatcaagctgcctaagta
ctccctgttcgagctggaaaacggccggaagagaatgctggcctctgccggcgaactgcagaagggaaac
gaactggccctgccctccaaatatgtgaacttcctgtacctggccagccactatgagaagctgaagggct
cccccgaggataatgagcagaaacagctgtttgtggaacagcacaagcactacctggacgagatcatcga
gcagatcagcgagttctccaagagagtgatcctggccgacgctaatctggacaaagtgctgtccgcctac
aacaagcaccgggataagcccatcagagagcaggccgagaatatcatccacctgtttaccctgaccaatc
tgggagcccctgccgccttcaagtactttgacaccaccatcgaccggaagaggtacaccagcaccaaaga
ggtgctggacgccaccctgatccaccagagcatcaccggcctgtacgagacacggatcgacctgtctcag
ctgggaggcgacagccccaagaagaagagaaaggtggaggccagcgggccggccggatccggacggctg
acgcattggacgattttgatctggatatgctgggaagtgacgccctcgatgattttgaccttgacatgct
tggttcggatgcccttgatgactttgacctcgacatgctcggcagtgacgcccttgatgatttcgacctg
gacatgctgattaactctagaagttccggatctccgaaaaagaaacgcaaagttggtagccagtacctgc
ccgacaccgacgaccggcaccggatcgaggaaaagcggaagcggacctacgagacattcaagagcatcat
gaagaagtccccccttcagcggccccaccgaccctagacctccacctagaagaatcgccgtgcccagcaga
tccagcgccagcgtgccaaaacctgccccccagccttaccccttcaccagcagcctgagcaccatcaact
acgacgagttccctaccatggtgttccccagcggccagatctctcaggcctctgctctggctccagcccc

tcctcaggtgctgcctcaggctcctgctcctgcaccagctccagccatggtgtctgcactggctcaggca
ccagcacccgtgcctgtgctggctcctggacctccacaggctgtggctccaccagcccctaaacctacac
aggccggcgagggcacactgtctgaagctctgctgcagctgcagttcgacgacgaggatctgggagccct
gctgggaaacagcaccgatcctgccgtgttcaccgacctggccagcgtggacaacagcgagttccagcag
ctgctgaaccagggcatccctgtggcccctcacaccaccgagcccatgctgatggaataccccgaggcca
tcacccggctcgtgacaggcgctcagaggcctcctgatccagctcctgcccctctgggagcaccaggcct
gcctaatggactgctgtctggcgacgaggacttcagctctatcgccgatatggatttctcagccttgctg
ggctctggcagcggcagccgggattccagggaagggatgtttttgccgaagcctgaggccggctccgcta
ttagtgacgtgtttgagggccgcgaggtgtgccagccaaaacgaatccggccatttcatcctccaggaag
tccatgggccaaccgcccactccccgccagcctcgcaccaacaccaaccggtccagtacatgagccagtc
gggtcactgaccccggcaccagtccctcagccactggatccagcgcccgcagtgactcccgaggccagtc
acctgttggaggatcccgatgaagagacgagccaggctgtcaaagcccttcgggagatggccgatactgt
gattccccagaaggaagaggctgcaatctgtggccaaatggacctttcccatccgcccccaaggggccat
ctggatgagctgacaaccacacttgagtccatgaccgaggatctgaacctggactcacccctgaccccgg
aattgaacgagattctggataccttcctgaacgacgagtgcctcttgcatgccatgcatatcagcacagg
actgtccatcttcgacacatctctgttttgaacgcgtaaatgattgcagatccactagttctagagctcg
ctgatcagcctcgactgtgccttctagttgccagccatctgttgtttgcccctcccccgtgccttccttg
accctggaaggtgccactcccactgtcctttcctaataaaatgaggaaattgcatcgcattgtctgagta
ggtgtcattctattctggggggtggggtggggcaggacagcaagggggaggattgggaagacaatagcag
gcatgctggggatgcggtgggctctatggcttctgaggcggaaagaaccagctggggctcgaggctagc

>AAV-ALN-flexGFP
cctgcaggcagctgcgcgctcgctcgctcactgaggccgcccgggcaaagcccgggcgtcgggcgacctt
tggtcgcccggcctcagtgagcgagcgagcgcgcagagagggagtggccaactccatcactaggggttcc
tgcggccgcacgcgtagagggcctatttcccatgattccttcatatttgcatatacgatacaaggctgtt
agagagataattggaattaatttgactgtaaacacaaagatattagtacaaaatacgtgacgtagaaagt
aataatttcttgggtagtttgcagttttaaaattatgttttaaaatggactatcatatgcttaccgtaac
ttgaaagtatttcgatttcttggctttatatatcttgtggaaaggacgaaacaccggggagccgctcgct
gcagcagcggttttagagctaggccaacatgaggatcacccatgtctgcagggcctagcaagttaaaata
aggctagtccgttatcaacttggccaacatgaggatcacccatgtctgcagggccaagtggcaccgagtc
ggtgctttttttcggtgcttttttttgaacgctgacgtcatcaacccgctccaaggaatcgcgggcccag
tgtcactaggcgggaacacccagcgcgcgtgcgccctggcaggaagatggctgtgagggacaggggagtg
gcgccctgcaatatttgcatgtcgctatgtgttctgggaaatcaccataaacgtgaaatgtctttggatt
tgggaatcttataagttctgtatgagaccactctttcccaggggggctgaatggagagtttgcagttttag
agctaggccaacatgaggatcacccatgtctgcagggcctagcaagttaaaataaggctagtccgttatc
aacttggccaacatgaggatcacccatgtctgcagggccaagtggcaccgagtcggtgctttttttcggt
gcttttttttgatccgacgccgccatctctaggcccgcgccggcccctcgcacagacttgtgggagaag
ctcggctactcccctgccccggttaatttgcatataatatttcctagtaactatagaggcttaatgtgcg
ataaaagacagataatctgttctttttaatactagctacattttacatgataggcttggatttctataag
agatacaaatactaaattattattttaaaaaacagcacaaaaggaaactcaccctaactgtaaagtaatt
gtgtgttttgagactataaatatcccttggagaaaagccttgtttggggggagcaaaggagtcgccttggt
tttagagctaggccaacatgaggatcacccatgtctgcagggcctagcaagttaaaataaggctagtccg
ttatcaacttggccaacatgaggatcacccatgtctgcagggccaagtggcaccgagtcggtgcttttttt
tcggtgcttttttttctgcagtatttagcatgccccacccatctgcaaggcattctggatagtgtcaaaa
cagccggaaatcaagtccgtttatctcaaactttagcattttgggaataaatgatatttgctatgctggt
taaattagatttagttaaatttcctgctgaagctctagtacgataagcaacttgacctaagtgtaaagt
tgagacttccttcaggtttatatagcttgtgcgccgcttgggtacctcggaatgcatccaagagtgaacc
gttttagagctaggccaacatgaggatcacccatgtctgcagggcctagcaagttaaaataaggctagtc
cgttatcaacttggccaacatgaggatcacccatgtctgcagggccaagtggcaccgagtcggtgctttt
tttgaacgctgacgtcatcaacccgctccaaggaatcgcgggcccagtgtcactaggcgggaacacccag
cgcgcgtgcgccctggcaggaagatggctgtgagggacaggggagtggcgccctgcaatatttgcatgtc
gctatgtgttctgggaaatcaccataaacgtgaaatgtctttggatttgggaatcttataagttctgtat
gagaccactctttcccaggggcggtgggtcattgtttccggttttagagctaggccaacatgaggatcac

ccatgtctgcagggcctagcaagttaaaataaggctagtccgttatcaacttggccaacatgaggatcac
ccatgtctgcagggccaagtggcaccgagtcggtgctttttttcggtgcttttttttctgcagtatttag
catgccccacccatctgcaaggcattctggatagtgtcaaaacagccggaaatcaagtccgtttatctca
aactttagcattttgggaataaatgatatttgctatgctggttaaattagattttagttaaatttcctgc
tgaagctctagtacgataagcaacttgacctaagtgtaaagttgagacttccttcaggtttatatagctt
gtgcgccgcttgggtacctcggtgccagtgacgccggcctgggttttagagctaggccaacatgaggatc
acccatgtctgcagggcctagcaagttaaaataaggctagtccgttatcaacttggccaacatgaggatc
acccatgtctgcagggccaagtggcaccgagtcggtgctttttttgagctgtactgagacgcacgtgcgc
cattgacgtcaataatgacgtatgttcccatagtaacgccaatagggactttccattgacgtcaatgggt
ggagtatttacggtaaactgcccacttggcagtacatcaagtgtatcatatgccaagtacgccccctatt
gacgtcaatgacggtaaatggcccgcctggcattatgcccagtacatgaccttatgggactttcctactt
ggcagtacatctacgtattagtcatcgctattaccatggtcgaggtgagccccacgttctgcttcactct
ccccatctcccccccctccccacccccaattttgtatttatttattttttaattattttgtgcagcgatg
ggggcggggggggggggggggcgcgcgccaggcggggcggggcggggcgaggggcggggcggggcgaggc
ggagaggtgcggcggcagccaatcagagcggcgcgctccgaaagtttccttttatggcgaggcggcggcg
gcggcggccctataaaaagcgaagcgcgcggcgggcgggagtcgctgcgacgctgccttcgccccgtgcc
ccgctccgccgccgcctcgcgccgcccgccccggctctgactgaccgcgttactcccacaggtgagcggg
cgggacggcccttctcctccgggctgtaattagcgcttggtttaatgacggcttgtttcttttctgtggc
tgcgtgaaagccttgaggggctccgggagggccctttgtgcggggggagcggctcggggctgtccgcggg
gggacggctgccttcgggggggacggggcagggcggggttcggcttctggcgtgtgaccggcggctctag
agcctctgctaaccatgttcatgccttcttctttttcctacagctcctgggcaacgtgctggttattgtg
ctgtctcatcattttggcaaagaattggatccccataacttcgtataaagtatcctatacgaagttatat
caaaataggaagaccaatgcttcaccatcgacccgaattgccaagcatcaccatcgacccataacttcgt
ataatgtatgctatacgaagttatactagggatccccctcgagttttacttgtacagctcgtccatgcc
gagagtgatcccggcggcggtcacgaactccagcaggaccatgtgatcgcgcttctcgttggggtctttg
ctcagggcggactgggtgctcaggtagtggttgtcgggcagcagcacggggccgtcgccgatgggggtgt
tctgctggtagtggtcggcgagctgcacgctgccgtcctcgatgttgtggcggatcttgaagttcacctt
gatgccgttcttctgcttgtcggccatgatatagacgttgtggctgttgtagttgtactccagcttgtgc
cccaggatgttgccgtcctccttgaagtcgatgcccttcagctcgatgcggttcaccagggtgtcgccct
cgaacttcacctcggcgcgggtcttgtagttgccgtcgtccttgaagaagatggtgcgctcctggacgta
gccttcgggcatggcggacttgaagaagtcgtgctgcttcatgtggtcggggtagcggctgaagcactgc
acgccgtaggtcagggtggtcacgagggtgggccagggcacgggcagcttgccggtggtgcagatgaact
tcagggtcagcttgccgtaggtggcatcgccctcgccctcgccggacacgctgaacttgtggccgtttac
gtcgccgtccagctcgaccaggatgggcaccaccccggtgaacagctcctcgcccttgctcaccatggtg
gcggtaccgaattctagtataacttcgtataggatactttatacgaagttatcattggattcttcctat
tttgatccaagcatcaccatcgaccctctagtccagatctcaccatcgacccataacttcgtatagcata
cattatacgaagttatgtccctcgaagaggttcgaattcgataataaaggaaatttattttcattgcaat
agtgtgttggttttttgtgtgcgagcggccgcaggaacccctagtgatggagttggccactccctctctg
cgcgctcgctcgctcactgaggccgggcgaccaaaggtcgcccgacgcccgggctttgcccgggcggcct
cagtgagcgagcgagcgcgcagctgcctgcagg

>AAV-N-dCas9$^{aa1-573}$-N-intein
ctgcgcgctcgctcgctcactgaggccgcccgggcaaagcccgggcgtcgggcgacctttggtcgcccgg
cctcagtgagcgagcgagcgcgcagagagggagtggccaactccatcactaggggttcctgcggccgcaa
ggtcgggcaggaagagggcctatttcccatgattccttcatatttgcatatacgatacaaggctgttaga
gagataattggaattaatttgactgtaaacacaaagatattagtacaaaatacgtgacgtagaaagtaat
aatttcttgggtagtttgcagttttaaaattatgttttaaaatggactatcatatgcttaccgtaacttg
aaagtatttcgatttcttggctttatatatcttgtggaaaggacgaaatacctcgggagccgctcgctgc
agcagcggttttagagctaggccaacatgaggatcacccatgtctgcagggcctagcaagttaaaataag
gctagtccgttatcaacttggccaacatgaggatcacccatgtctgcagggccaagtggcaccgagtcgg
tgcttttttttgagcgacctgtacccgttacataacttacggtaaatggcccgcctggctgaccgcccaa
cgacccccgcccattgacgtcaatagtaacgccaatagggactttccattgacgtcaatgggtggagtat
ttacggtaaactgcccacttggcagtacatcaagtgtatcatatgccaagtacgccccctattgacgtca

atgacggtaaatggcccgcctggcattgtgcccagtacatgaccttatgggactttcctacttggcagta
catctacgtattagtcatcgctattaccatggtcgaggtgagccccacgttctgcttcactctccccatc
tcccccccctccccacccccaattttgtatttatttattttttaattattttgtgcagcgatgggggcgg
gggggggggggggcgcgcgccaggcggggcggggcggggcgaggggcggggcggggcgaggcggagagg
tgcggcggcagccaatcagagcggcgcgctccgaaagtttccttttatggcgaggcggcggcggcggcgg
ccctataaaaagcgaagcgcgcggcgggcgggagtcgctgcgacgctgccttcgccccgtgccccgctcc
gccgccgcctcgcgccgcccgccccggctctgactgaccgcgttactcccacaggtgagcgggcgggacg
gcccttctcctccgggctgtaattagctgagcaagaggtaagggtttaagggatggttggttggtggggt
attaatgtttaattacctggagcacctgcctgaaatcactttttttcaggttggatccttaattaaataa
cttcgtatagcatacattatacgaagttatagtcataacttcgtatagtatacattatacgaagttatgt
tgggcaggttgtccacccgcatcaggtcaagttcccgctcaaaaatctcgtcaatgggcagcatctgccc
gtccacggtcatgaacttgtggtctttggtggctctaatcaggctgccgtcctccagacaatactcaaac
acctcctgctcgcccctgtcgtgccactgagccacgggctgggtgtagatgttgccgttgttgtccacgc
tatacacggtgcattcaatccgcttctccacaatcttgccgatgggcagcagcccatactccacggtcag
gatttcggtctcgtaggacaggcactcgatttcttgaagtagtcctctttcagctgcttcacggtcact
ttccggttggtcttgaacagcaggtccacgatggcctttttctgctcgccgctcaggaaggcgggctttc
tcattccctcggtcacgtatttcactttggtcagctcgttatacacggtgaagtactcgtacagcaggct
gtgcttgggcagcaccttctcgttgggcaggttcttatcgaagttggtcatccgctcgatgaagctctgg
gcggaagcgcccttgtccaccacttcctcgaagttccaggggtgatggtttcctcgctctttctggtca
tccaggcgaatctgctgtttcccctggccagagggcccacgtagtaggggatgcggaaggtcaggatctt
ctcgatcttttcccggttgtccttcaggaatgggtaaaaatcttcctgccgccgcagaatggcgtgcagc
tctcccaggtggatctggtgggggatgctgccgttgtcgaaggtccgctgcttccgcagcaggtcctctc
tgttcagcttcacgagcagttcctcggtgccgtccatcttttccaggatgggcttgatgaacttgtagaa
ctcttcctggctggctccgccgtcaatgtagccggcgtagccgttcttgctctggtcgaagaaaatctct
ttgtacttctcaggcagctgctgccgcacgagagctttcagcagggtcaggtcctggtggtgctcgtcgt
atctcttgatcatagaggcgctcagggggccttggtgatctcggtgttcactctcaggatgtcgctcag
caggatggcgtcggacaggttcttggcggccagaaacaggtcggcgtactggtcgccgatctgggccagc
aggttgtccaggtcgtcgtcgtaggtgtccttgctcagctgcagtttggcatcctcggccaggtcgaagt
tgctcttgaagttgggggtcaggcccaggctcagggcaatcaggttgccgaacaggccattcttcttctc
gccgggcagctgggcgatcagattttccagccgtctgctcttgctcagtctggcagacaggatggccttg
gcgtccacgccgctggcgttgatggggtttcctcgaacagctggttgtaggtctgcaccagctggatga
acagcttgtccacgtcgctgttgtcggggttcaggtcgccctcgatcaggaagtggccccggaacttgat
catgtgggccagggccagatagatcagccgcaggtcggccttgtcggtgctgtccaccagtttctttctc
aggtggtagatggtggggtacttctcgtggtaggccacctcgtccacgatgttgccgaagatggggtgcc
gctcgtgcttcttatcctcttccaccaggaaggactcttccagtctgtggaagaagctgtcgtccacctt
ggccatctcgttgctgaagatctcttgcagatagcagatccggttcttccgtctggtgtatcttcttctg
gcggttctcttcagccgggtggcctcggctgtttcgccgctgtcgaacagcagggctccgatcaggttct
tcttgatgctgtgccggtcggtgttgcccagcaccttgaatttcttgctgggcaccttgtactcgtcggt
gatcacggcccagcccacagagttggtgccgatggccaggccgatgctgtacttcttgtcggctgctggg
actccgtggatacctttatcgtcgtcatccttgtagtcagcggccgccaccttcctctttttcttaggtc
ccatggtggcggcataacttcgtataatgtatgctatacgaagttatagtcataacttcgtataatgtat
actatacgaagttattgaacgcgtaataaaggaaatttattttcattgcaatagtgtgttggttttttgt
gtgcggccgcaggaacccctagtgatggagttggccactccctctctgcgcgctcgctcgctcactgagg
ccgggcgaccaaaggtcgcccgacgcccgggctttgcccgggcggcctcagtgagcgagcgagcgcgcag

>AAV-C-dCas9$^{aa574-1368}$-VP64-C-intein
ctgcgcgctcgctcgctcactgaggccgcccgggcaaagcccgggcgtcgggcgacctttggtcgcccgg
cctcagtgagcgagcgagcgcgcagagagggagtggccaactccatcactaggggttcctgcggccgcaa
ggtcgggcaggaagagggcctatttcccatgattccttcatatttgcatatacgatacaaggctgttaga
gagataattggaattaatttgactgtaaacacaaagatattagtacaaaatacgtgacgtagaaagtaat
aatttcttgggtagtttgcagttttaaaattatgttttaaaatggactatcatatgcttaccgtaacttg
aaagtatttcgatttcttggctttatatatcttgtggaaaggacgaaatacctcggggctgaatggagag
tttgcagttttagagctaggccaacatgaggatcacccatgtctgcagggcctagcaagttaaaataagg

ctagtccgttatcaacttggccaacatgaggatcacccatgtctgcagggccaagtggcaccgagtcggt
gcttttttttgagcgacctgtacccgttacataacttacggtaaatggcccgcctggctgaccgcccaac
gacccccgcccattgacgtcaatagtaacgccaatagggactttccattgacgtcaatgggtggagtatt
tacggtaaactgcccacttggcagtacatcaagtgtatcatatgccaagtacgccccctattgacgtcaa
tgacggtaaatggcccgcctggcattgtgcccagtacatgaccttatgggactttcctacttggcagtac
atctacgtattagtcatcgctattaccatggtcgaggtgagccccacgttctgcttcactctccccatct
cccccccctccccacccccaattttgtatttatttattttttaattattttgtgcagcgatgggggcggg
ggggggggggggcgcgcgccaggcggggcggggcggggcgaggggcggggcggggcgaggcggagaggt
gcggcggcagccaatcagagcggcgcgctccgaaagtttccttttatggcgaggcggcggcggcggcggc
cctataaaaagcgaagcgcgcggcgggcgggagtcgctgcgacgctgccttcgccccgtgccccgctccg
ccgccgcctcgcgccgcccgccccggctctgactgaccgcgttactcccacaggtgagcgggcgggacgg
cccttctcctccgggctgtaattagctgagcaagaggtaagggtttaagggatggttggttggtggggta
ttaatgtttaattacctggagcacctgcctgaaatcactttttttcaggttggatccttaattaataata
cgactcactataggggccgccaccatgataaagattgccaccagaaagtatctgggcaagcagaacgtgt
atgacatcggcgtggagagagaccacaacttcgccctgaagaacggcttcatcgccagcaactgcttcga
ctccgtggaaatctccggcgtggaagatcggttcaacgcctccctgggcacataccacgatctgctgaaa
attatcaaggacaaggacttcctggacaatgaggaaaacgaggacattctggaagatatcgtgctgaccc
tgacactgtttgaggacagagagatgatcgaggaacggctgaaaacctatgcccacctgttcgacgacaa
agtgatgaagcagctgaagcggcggagatacaccggctggggcaggctgagccggaagctgatcaacggc
atccgggacaagcagtccggcaagacaatcctggatttcctgaagtccgacggcttcgccaacagaaact
tcatgcagctgatccacgacgacagcctgacctttaaagaggacatccagaaagcccaggtgtccggcca
gggcgatagcctgcacgagcacattgccaatctggccggcagccccgccattaagaagggcatcctgcag
acagtgaaggtggtggacgagctcgtgaaagtgatgggccggcacaagcccgagaacatcgtgatcgaaa
tggccagagagaaccagaccacccagaagggacagaagaacagccgcgagagaatgaagcggatcgaaga
gggcatcaaagagctgggcagccagatcctgaaagaacaccccgtggaaaacacccagctgcagaacgag
aagctgtacctgtactacctgcagaatgggcgggatatgtacgtggaccaggaactggacatcaaccggc
tgtccgactacgatgtggaccatatcgtgcctcagagctttctgaaggacgactccatcgacaacaaggt
gctgaccagaagcgacaaggcccggggcaagagcgacaacgtgccctccgaagaggtcgtgaagaagatg
aagaactactggcggcagctgctgaacgccaagctgattacccagagaaagttcgacaatctgaccaagg
ccgagagaggcggcctgagcgaactggataaggccggcttcatcaagagacagctggtggaaacccggca
gatcacaaagcacgtggcacagatcctggactcccggatgaacactaagtacgacgagaatgacaagctg
atccgggaagtgaaagtgatcaccctgaagtccaagctggtgtccgatttccggaaggatttccagtttt
acaaagtgcgcgagatcaacaactaccaccacgcccacgacgcctacctgaacgccgtcgtgggaaccgc
cctgatcaaaaagtaccctaagctggaaagcgagttcgtgtacggcgactacaaggtgtacgacgtgcgg
aagatgatcgccaagagcgagcaggaaatcggcaaggctaccgccaagtacttcttctacagcaacatca
tgaactttttcaagaccgagattaccctggccaacggcgagatccggaagcggcctctgatcgagacaaa
cggcgaaaccggggagatcgtgtgggataagggccgggattttgccaccgtgcggaaagtgctgagcatg
ccccaagtgaatatcgtgaaaaagaccgaggtgcagacaggcggcttcagcaaagagtctatcctgccca
agaggaacagcgataagctgatcgccagaaagaaggactgggaccctaagaagtacggcggcttcgacag
ccccaccgtggcctattctgtgctggtggtggccaaagtggaaaagggcaagtccaagaaactgaagagt
gtgaaagagctgctggggatcaccatcatggaaagaagcagcttcgagaagaatcccatcgactttctgg
aagccaagggctacaaagaagtgaaaaaggacctgatcatcaagctgcctaagtactccctgttcgagct
ggaaaacggccggaagagaatgctggcctctgccggcgaactgcagaagggaaacgaactggccctgccc
tccaaatatgtgaacttcctgtacctggccagccactatgagaagctgaagggctcccccgaggataatg
agcagaaacagctgtttgtggaacagcacaagcactacctggacgagatcatcgagcagatcagcgagtt
ctccaagagagtgatcctggccgacgctaatctggacaaagtgctgtccgcctacaacaagcaccgggat
aagcccatcagagagcaggccgagaatatcatccacctgtttaccctgaccaatctgggagcccctgccg
ccttcaagtactttgacaccaccatcgaccggaagaggtacaccagcaccaaagaggtgctggacgccac
cctgatccaccagagcatcaccggcctgtacgagacacggatcgacctgtctcagctgggaggcgacgcc
tatccctatgacgtgcccgattatgccagcctgggcagcggctcacccaagaagaagaggaaggtggctg
caggaggcggtggaagcgggcgcgccgacgcattggacgattttgatctggatatgctgggaagtgacgc
cctcgatgattttgaccttgacatgcttggttcggatgcccttgatgactttgacctcgacatgctcggc
agtgacgcccttgatgatttcgacctggacatgctgattaactctagaagttccggatctccgaaaaaga

aacgcaaagtttgaacgcgtaataaaggaaatttattttcattgcaatagtgtgttggtttttgtgtgc
ggccgcaggaacccctagtgatggagttggccactccctctctgcgcgctcgctcgctcactgaggccgg
gcgaccaaaggtcgcccgacgcccgggctttgcccgggcggcctcagtgagcgagcgagcgcgcag

>AAV-Lmx1a-Nr4a2-SAM
ctgcgcgctcgctcgctcactgaggccgcccgggcaaagcccgggcgtcgggcgacctttggtcgcccgg
cctcagtgagcgagcgagcgcgcagagagggagtggccaactccatcactaggggttcctgcggccgccg
ttacataacttacggtaaatggcccgcctggctgaccgcccaacgacccccgcccattgacgtcaatagt
aacgccaatagggactttccattgacgtcaatgggtggagtatttacggtaaactgcccacttggcagta
catcaagtgtatcatatgccaagtacgccccctattgacgtcaatgacggtaaatggcccgcctggcatt
gtgcccagtacatgaccttatgggactttcctacttggcagtacatctacgtattagtcatcgctattac
catggtcgaggtgagccccacgttctgcttcactctccccatctcccccccctccccacccccaattttg
tatttatttattttttaattattttgtgcagcgatgggggcgggggggggggggggcgcgcgccaggcg
gggcggggcggggcgaggggcggggcggggcgaggcggagaggtgcggcggcagccaatcagagcggcgc
gctccgaaagtttccttttatggcgaggcggcggcggcggcggccctataaaaagcgaagcgcgcggcgg
gcgggagtcgctgcgacgctgccttcgccccgtgccccgctccgccgccgcctcgcgccgcccgccccgg
ctctgactgaccgcgttactcccacaggtgagcgggcgggacggcccttctcctccgggctgtaattagc
tgagcaagaggtaagggtttaagggatggttggttggtggggtattaatgtttaattacctggagcacct
gcctgaaatcactttttttcaggttggagcgatcgcgccgccaccatggcttcaaactttactcagttcg
tgctcgtggacaatggtgggacaggggatgtgacagtggctccttctaatttcgctaatggggtggcaga
gtggatcagctccaactcacggagccaggcctacaaggtgacatgcagcgtcaggcagtctagtgcccag
aagagaaagtataccatcaaggtggaggtccccaaagtggctacccagacagtgggcggagtcgaactgc
ctgtcgccgcttggaggtcctacctgaacatggagctcactatcccaattttcgctaccaattctgactg
tgaactcatcgtgaaggcaatgcaggggctcctcaaagacggtaatcctatcccttccgccatcgccgct
aactcaggtatctacagcgctggaggaggtggaagcggaggaggaggaagcggaggaggaggtagcggac
ctaagaaaaagaggaaggtggcgaccgctggatccccttcagggcagatcagcaaccaggccctggctct
ggcccctagctccgctccagtgctggcccagactatggtgccctctagtgctatggtgcctctggcccag
ccacctgctccagcccctgtgctgaccccaggaccaccccagtcactgagcgctccagtgcccaagtcta
cacaggccggcgaggggactctgagtgaagctctgctgcacctgcagttcgacgctgatgaggacctggg
agctctgctggggaacagcaccgatcccggagtgttcacagatctggcctccgtggacaactctgagttt
cagcagctgctgaatcagggcgtgtccatgtctcatagtacagccgaaccaatgctgatggagtaccccg
aagccattacccggctggtgaccggcagccagcggcccccgaccccgctccaactcccctgggaaccag
cggcctgcctaatgggctgtccggagatgaagacttctcaagcatcgctgatatggactttagtgccctg
ctgtcacagatttcctctagtgggcagggaggaggtggaagcggcttcagcgtggacaccagtgccctgc
tggacctgttcagcccctcggtgaccgtgcccgacatgagcctgcctgaccttgacagcagcctggccag
tatccaagagctcctgtctccccaggagcccccaggcctcccgaggcagagaacagcagcccggattca
gggaagcagctggtgcactacacagcgcagccgctgttcctgctggaccccggctccgtggacaccggga
gcaacgacctgccggtgctgtttgagctgggagagggctcctacttctccgaaggggacggcttcgccga
ggaccccaccatctccctgctgacaggctcggagcctcccaaagccaaggaccccactgtctcctgtaca
tccgggtttgccaacgaactaggcccgcgacttatgggtaaataaaataaaggaaatttattttcattgc
aatagtgtgttggtttttgtgtggtcgacgagggcctatttcccatgattccttcatatttgcatatac
gatacaaggctgttagagagataattggaattaatttgactgtaaacacaaagatattagtacaaaatac
gtgacgtagaaagtaataatttcttgggtagtttgcagttttaaaattatgttttaaaatggactatcat
atgcttaccgtaacttgaaagtatttcgatttcttggctttatatatcttgtggaaaggacgaaacaccg
gagcaaaggagtcgccttggttttagagctaggccaacatgaggatcacccatgtctgcagggcctagca
agttaaaataaggctagtccgttatcaacttggccaacatgaggatcacccatgtctgcagggccaagtg
gcaccgagtcggtgctttttttcggtgcttttttttgaacgctgacgtcatcaacccgctccaaggaatc
gcgggcccagtgtcactaggcgggaacacccagcgcgcgtgcgccctggcaggaagatggctgtgaggga
caggggagtggcgccctgcaatatttgcatgtcgctatgtgttctgggaaatcaccataaacgtgaaatg
tctttggatttgggaatcttataagttctgtatgagaccactctttcccagaatgcatccaagagtgaac
cgttttagagctaggccaacatgaggatcacccatgtctgcagggcctagcaagttaaaataaggctagt
ccgttatcaacttggccaacatgaggatcacccatgtctgcagggccaagtggcaccgagtcggtgcttt
ttttcggtgcttttttttgatccgacgccgccatctctaggcccgcgccggcccctcgcacagacttgt

gggagaagctcggctactcccctgccccggttaatttgcatataatatttcctagtaactatagaggctt
aatgtgcgataaaagacagataatctgttctttttaatactagctacattttacatgataggcttggatt
tctataagagatacaaatactaaattattattttaaaaaacagcacaaaaggaaactcaccctaactgta
aagtaattgtgtgttttgagactataaatatcccttggagaaaagccttgtttggcggtgggtcattgtt
tccggttttagagctaggccaacatgaggatcacccatgtctgcagggcctagcaagttaaaataaggct
agtccgttatcaacttggccaacatgaggatcacccatgtctgcagggccaagtggcaccgagtcggtgc
tttttttcggtgctttttttctgcagtatttagcatgccccacccatctgcaaggcattctggatagtg
tcaaaacagccggaaatcaagtccgtttatctcaaactttagcattttgggaataaatgatatttgctat
gctggttaaattagattttagttaaatttcctgctgaagctctagtacgataagcaacttgacctaagtg
taaagttgagacttccttcaggtttatatagcttgtgcgccgcttgggtacctcggtgccagtgacgccg
gcctgggttttagagctaggccaacatgaggatcacccatgtctgcagggcctagcaagttaaaataagg
ctagtccgttatcaacttggccaacatgaggatcacccatgtctgcagggccaagtggcaccgagtcggt
gctttttttgagctgtactgagacgttaattaacgtgcagcggccgcaggaacccctagtgatggagttg
gccactccctctctgcgcgctcgctcgctcactgaggccgggcgaccaaaggtcgcccgacgcccgggct
ttgcccgggcggcctcagtgagcgagcgagcgcgcag

>AAV-flexGFP
gcgcgctcgctcgctcactgaggccgcccgggcaaagcccgggcgtcgggcgacctttggtcgcccggcc
tcagtgagcgagcgagcgcgcagagagggagtggccaactccatcactaggggttccttgtagttaatga
ttaacccgccatgctacttatctacgtagccatgctctaggaagatcgtaccattgacgtcaataatgac
gtatgttcccatagtaacgccaatagggactttccattgacgtcaatgggtggagtatttacggtaaact
gcccacttggcagtacatcaagtgtatcatatgccaagtacgccccctattgacgtcaatgacggtaaat
ggcccgcctggcattatgcccagtacatgaccttatgggactttcctacttggcagtacatctacgtatt
agtcatcgctattaccatggtcgaggtgagccccacgttctgcttcactctccccatctcccccccctcc
ccacccccaattttgtatttatttattttttaattattttgtgcagcgatgggggcggggggggggggg
ggcgcgcgccaggcggggcggggcggggcgaggggcggggcggggcgaggcggagaggtgcggcggcagc
caatcagagcggcgcgctccgaaagtttccttttatggcgaggcggcggcggcggcggccctataaaaag
cgaagcgcgcggcgggcgggagtcgctgcgacgctgccttcgccccgtgccccgctccgccgccgcctcg
cgccgcccgccccggctctgactgaccgcgttactcccacaggtgagcgggcgggacggcccttctcctc
cgggctgtaattagcgcttggtttaatgacggcttgtttcttttctgtggctgcgtgaaagccttgaggg
gctccgggagggccctttgtgcggggggagcggctcggggctgtccgcggggggacggctgccttcgggg
gggacggggcagggcggggttcggcttctggcgtgtgaccggcggctctagagcctctgctaaccatgtt
catgccttcttctttttcctacagctcctgggcaacgtgctggttattgtgctgtctcatcattttggca
aagaattggatccccataacttcgtataaagtatcctatacgaagttatatcaaaataggaagaccaatg
cttcaccatcgacccgaattgccaagcatcaccatcgacccataacttcgtataatgtatgctatacgaa
gttatactagggatccccccctcgagttttacttgtacagctcgtccatgccgagagtgatcccggcggcg
gtcacgaactccagcaggaccatgtgatcgcgcttctcgttggggtctttgctcagggcggactgggtgc
tcaggtagtggttgtcgggcagcagcacggggccgtcgccgatgggggtgttctgctggtagtggtcggc
gagctgcacgctgccgtcctcgatgttgtggcggatcttgaagttcaccttgatgccgttcttctgcttg
tcggccatgatatagacgttgtggctgttgtagttgtactccagcttgtgccccaggatgttgccgtcct
ccttgaagtcgatgcccttcagctcgatgcggttcaccagggtgtcgccctcgaacttcacctcggcgcg
ggtcttgtagttgccgtcgtccttgaagaagatggtgcgctcctggacgtagccttcgggcatggcggac
ttgaagaagtcgtgctgcttcatgtggtcggggtagcggctgaagcactgcacgccgtaggtcagggtgg
tcacgagggtgggccagggcacgggcagcttgccggtggtgcagatgaacttcagggtcagcttgccgta
ggtggcatcgccctcgccctcgccggacacgctgaacttgtggccgtttacgtcgccgtccagctcgacc
aggatgggcaccaccccggtgaacagctcctcgcccttgctcaccatggtggcggtaccgaattctagta
taacttcgtataggatactttatacgaagttatcattgggattcttcctattttgatccaagcatcacca
tcgaccctctagtccagatctcaccatcgacccataacttcgtatagcatacattatacgaagttatgtc
cctcgaagaggttcgaattcgatatcaagcttatcgataatcaacctctggattacaaaatttgtgaaag
attgactggtattcttaactatgttgctccttttacgctatgtggatacgctgctttaatgcctttgtat
catgctattgcttcccgtatggctttcattttctcctccttgtataaatcctggttgctgtctctttatg
aggagttgtggcccgttgtcaggcaacgtggcgtggtgtgcactgtgtttgctgacgcaacccccactgg
ttggggcattgccaccacctgtcagctcctttccgggactttcgctttccccctccctattgccacggcg

gaactcatcgccgcctgccttgcccgctgctggacaggggctcggctgttgggcactgacaattccgtgg
tgttgtcggggaaatcatcgtcctttccttggctgctcgcctgtgttgccacctggattctgcgcgggac
gtccttctgctacgtcccttcggccctcaatccagcggaccttccttcccgcggcctgctgccggctctg
cggcctcttccgcgtcttcgccttcgccctcagacgagtcggatctccctttgggccgcctccccgcatc
gataccgtcgacccgggcggccgcttcgagcagacatgataagatacattgatgagtttggacaaaccac
aactagaatgcagtgaaaaaaatgctttatttgtgaaatttgtgatgctattgctttatttgtaaccatt
ataagctgcaataaacaagttaacaacaacaattgcattcattttatgtttcaggttcagggggagatgt
gggaggtttttaaagcaagtaaaacctctacaaatgtggtaaaatcgataaggatcttcctagagcatg
gctacgtagataagtagcatggcgggttaatcattaactacaaggaacccctagtgatggagttggccac
tccctctctgcgcgctcgctcgctcactgaggccgggcgaccaaaggtcgcccgacgcccgggctttgcc
cgggcggcctcagtgagcgagcgagcgcgcagctg
