# Supplementary material for: Parkinson's disease motor symptoms rescue by CRISPRa‐reprogramming astrocytes into GABAergic neurons
Source: EMBO Mol Med. 2022 Apr 4;14(5):e14797. doi: 10.15252/emmm.202114797 (PMC9081909; doi:10.15252/emmm.202114797)

Appendix Figure S1a

WB: α-P2A-Ab

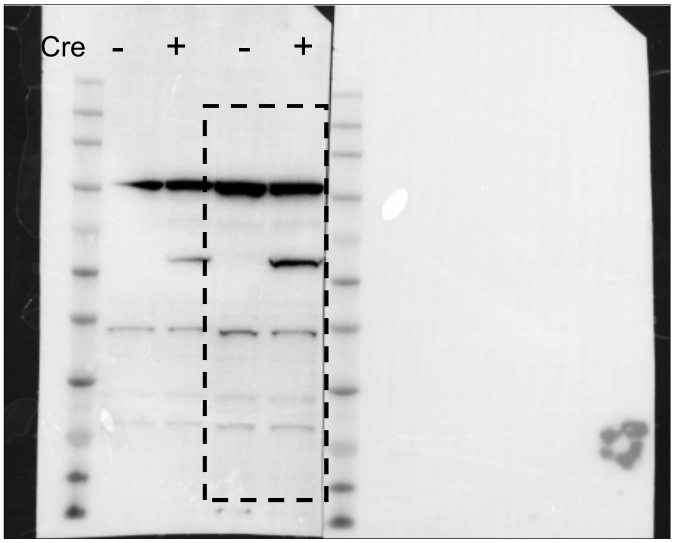

WB: α-N-Cas9-Ab

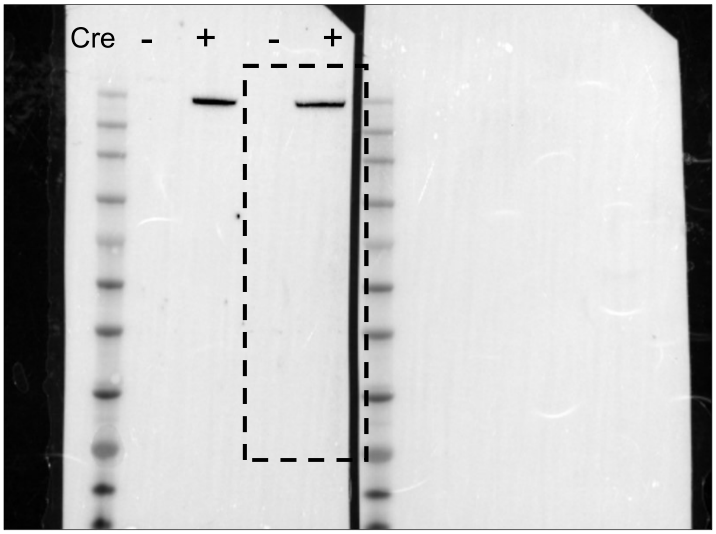

Appendix Figure S1c

Southern Blot

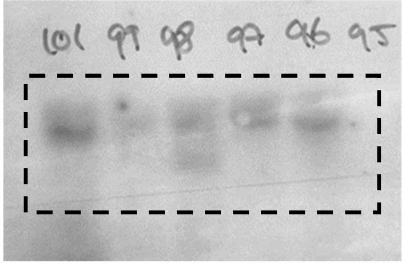

PCR Genotyping

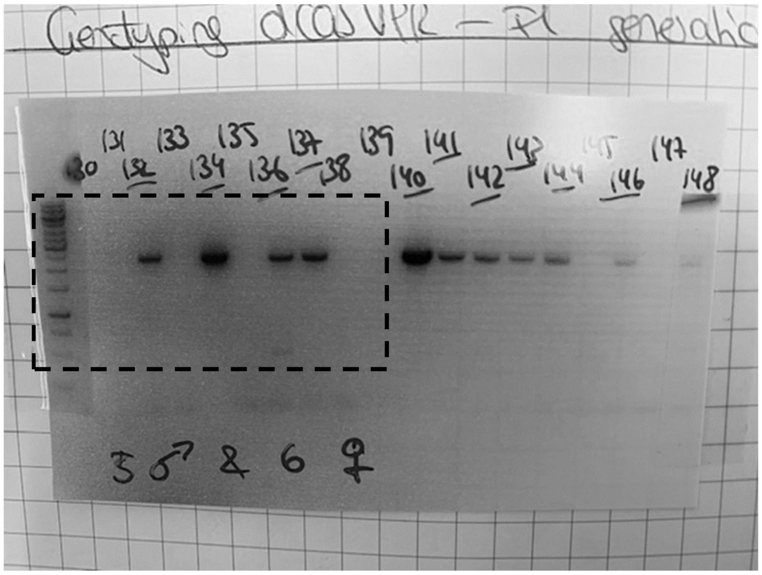

Appendix Figure S1c

WB: α-ACTB

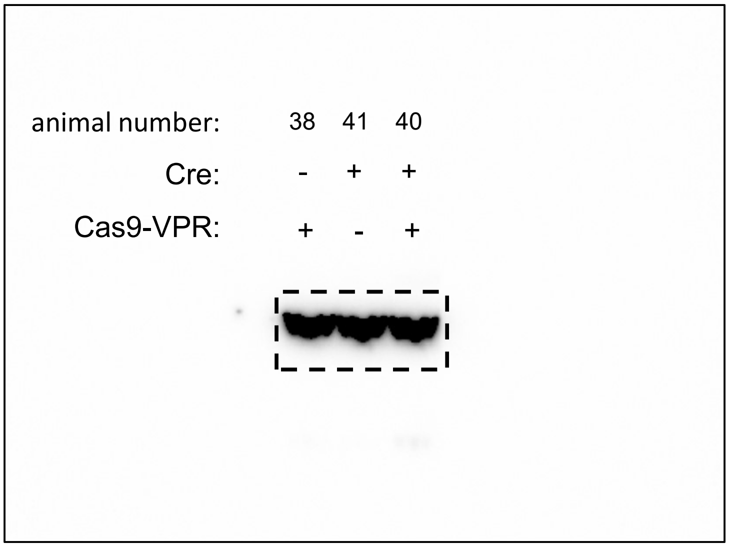

WB: α-N-Cas9-Ab

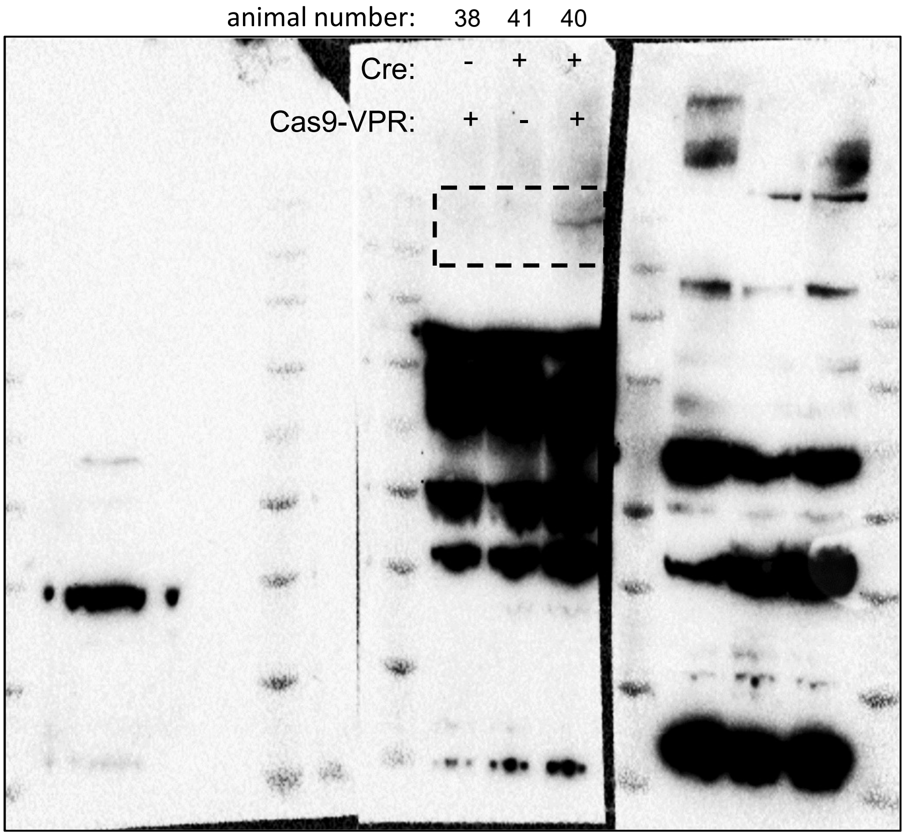

Supplement: Supplementary file 2 — Source Data for Appendix [file EMMM-14-e14797-s005.zip › emmm202114797-sup-0007-SDataEV/emmm202114797-sup-0007-SDataAppendixFigS1.pdf]
