# Supplementary figures and images for: Parkinson's disease motor symptoms rescue by CRISPRa‐reprogramming astrocytes into GABAergic neurons
Source: EMBO Mol Med. 2022 Apr 4;14(5):e14797. doi: 10.15252/emmm.202114797 (PMC9081909; doi:10.15252/emmm.202114797)

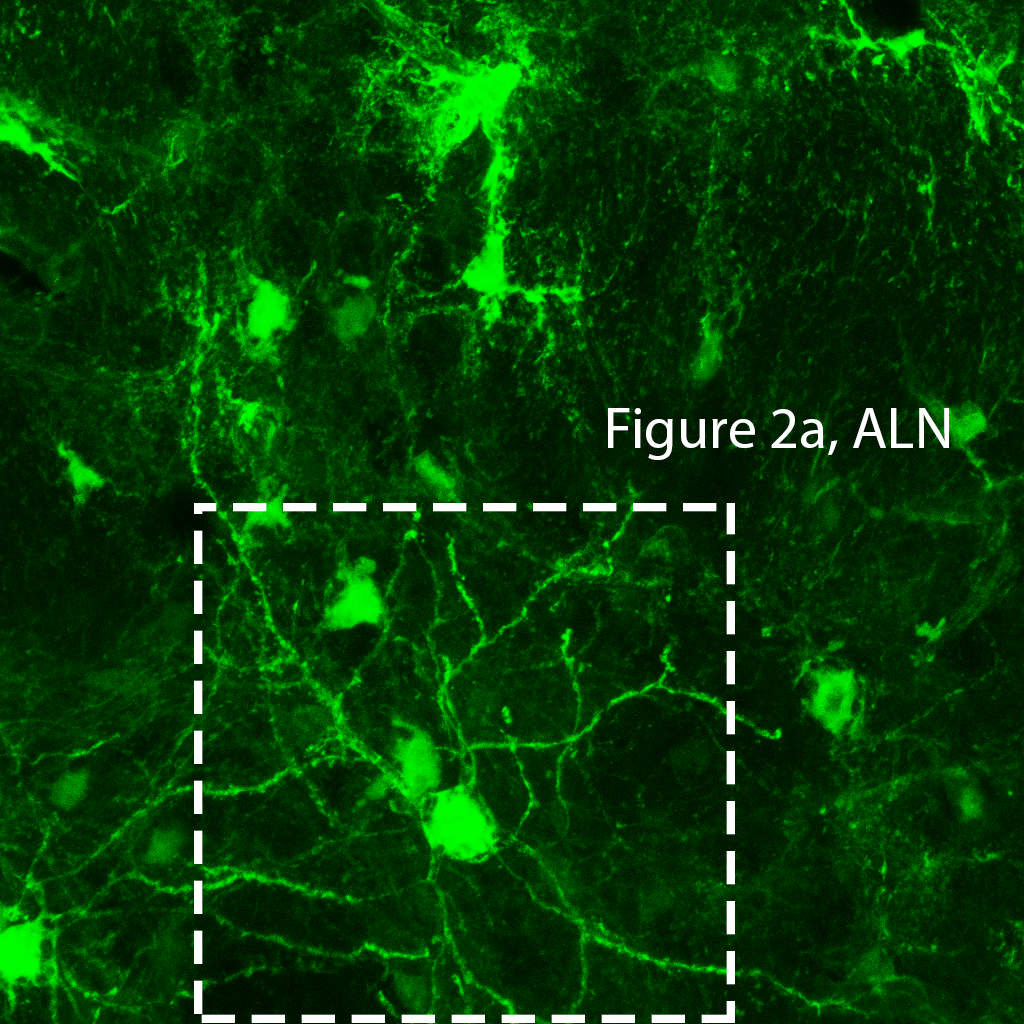

Supplement: Supplementary file 4 — Source Data for Figure 2 [file EMMM-14-e14797-s002.zip › Figure2a_ALN.tif]

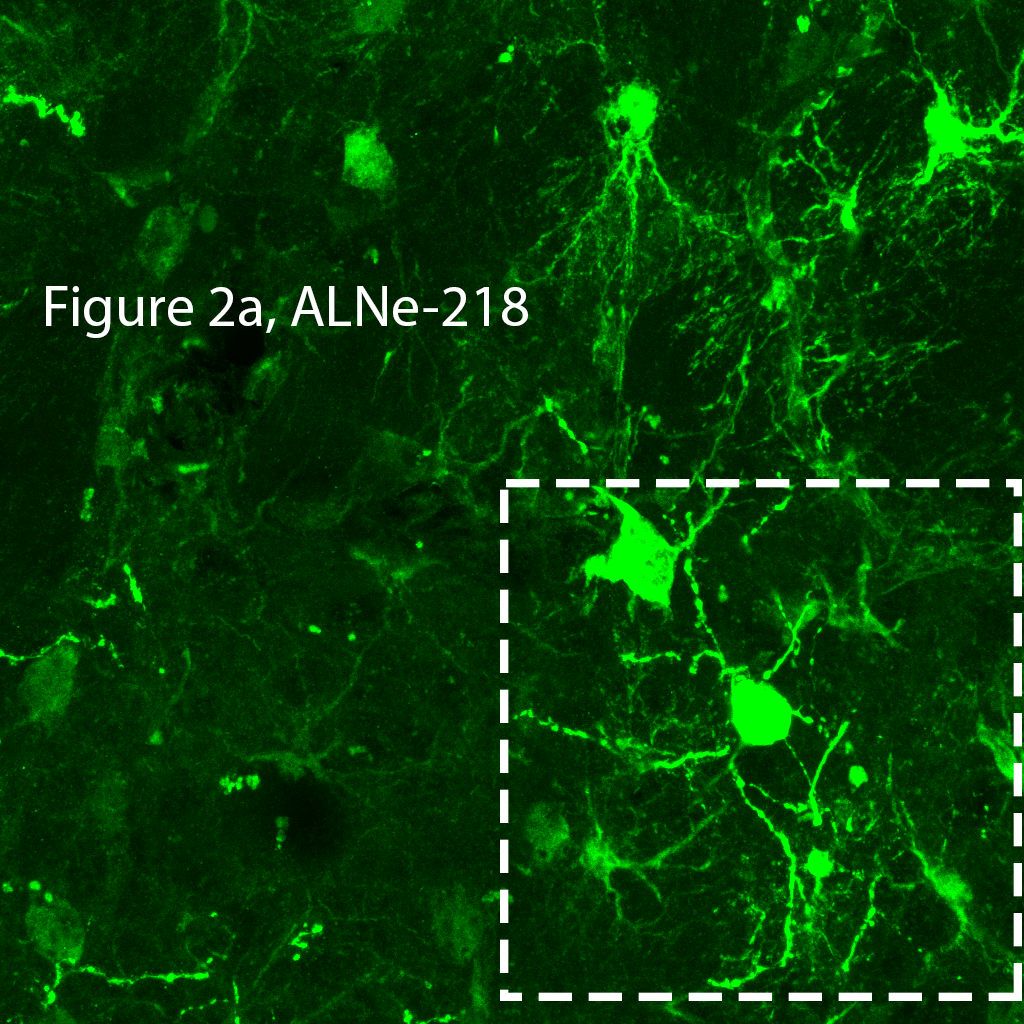

Supplement: Supplementary file 4 — Source Data for Figure 2 [file EMMM-14-e14797-s002.zip › Figure2a_ALNe-218.tif]

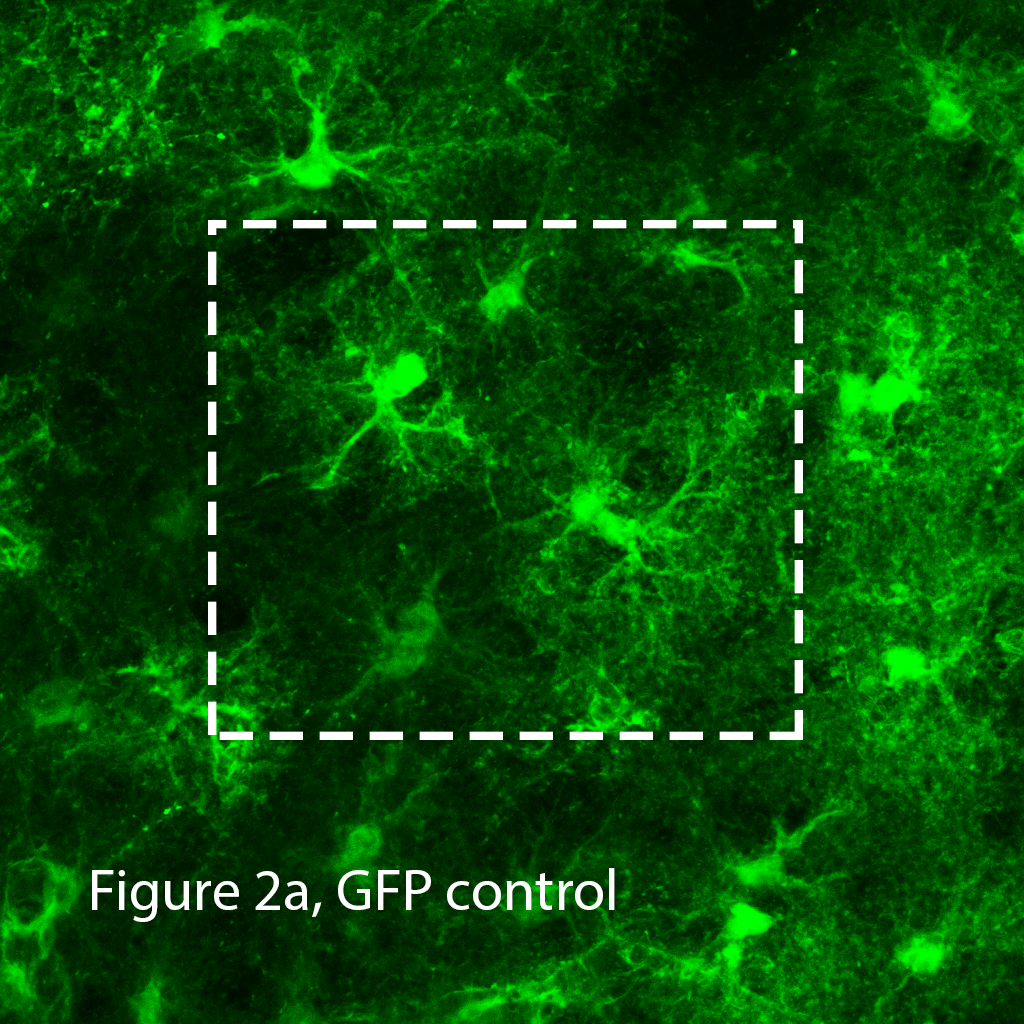

Supplement: Supplementary file 4 — Source Data for Figure 2 [file EMMM-14-e14797-s002.zip › Figure2a_GFPcontrol.tif]

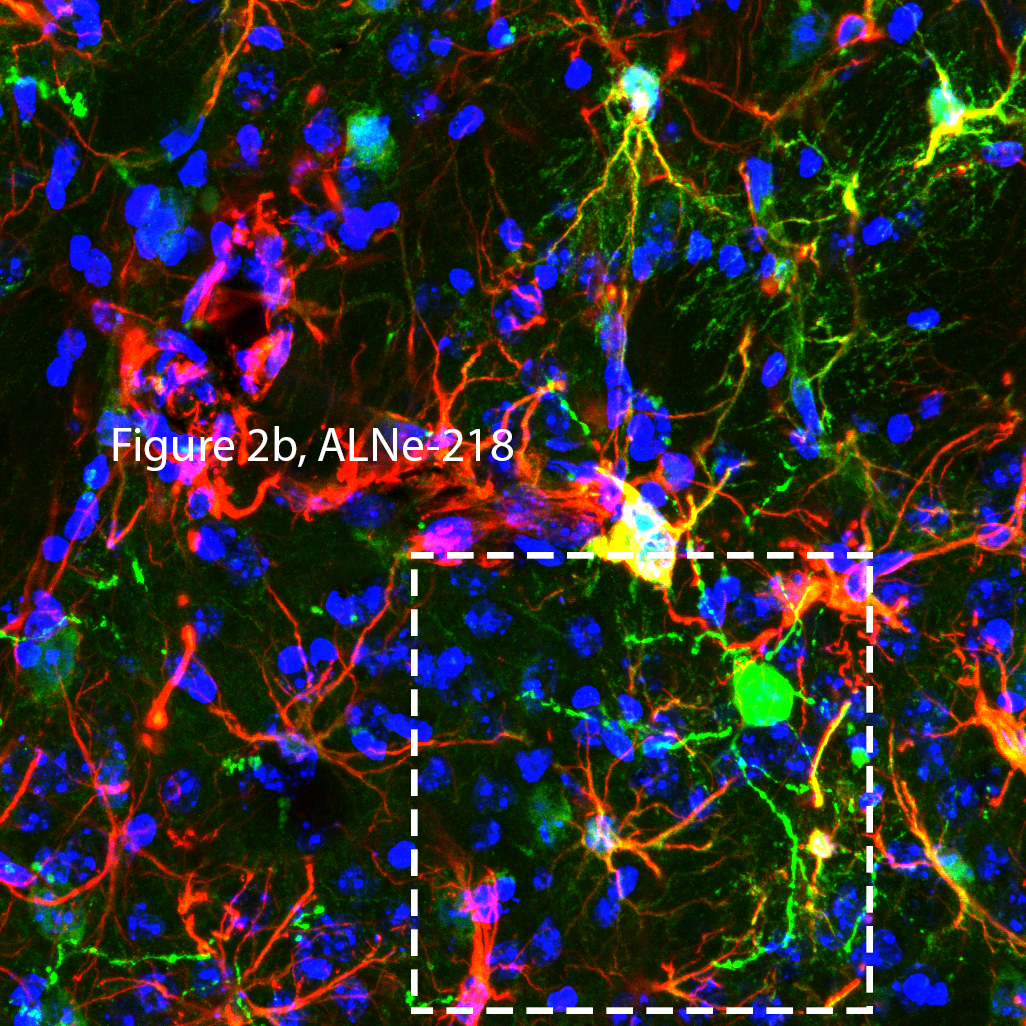

Supplement: Supplementary file 4 — Source Data for Figure 2 [file EMMM-14-e14797-s002.zip › Figure2b_ALNe-218_composite.tif]

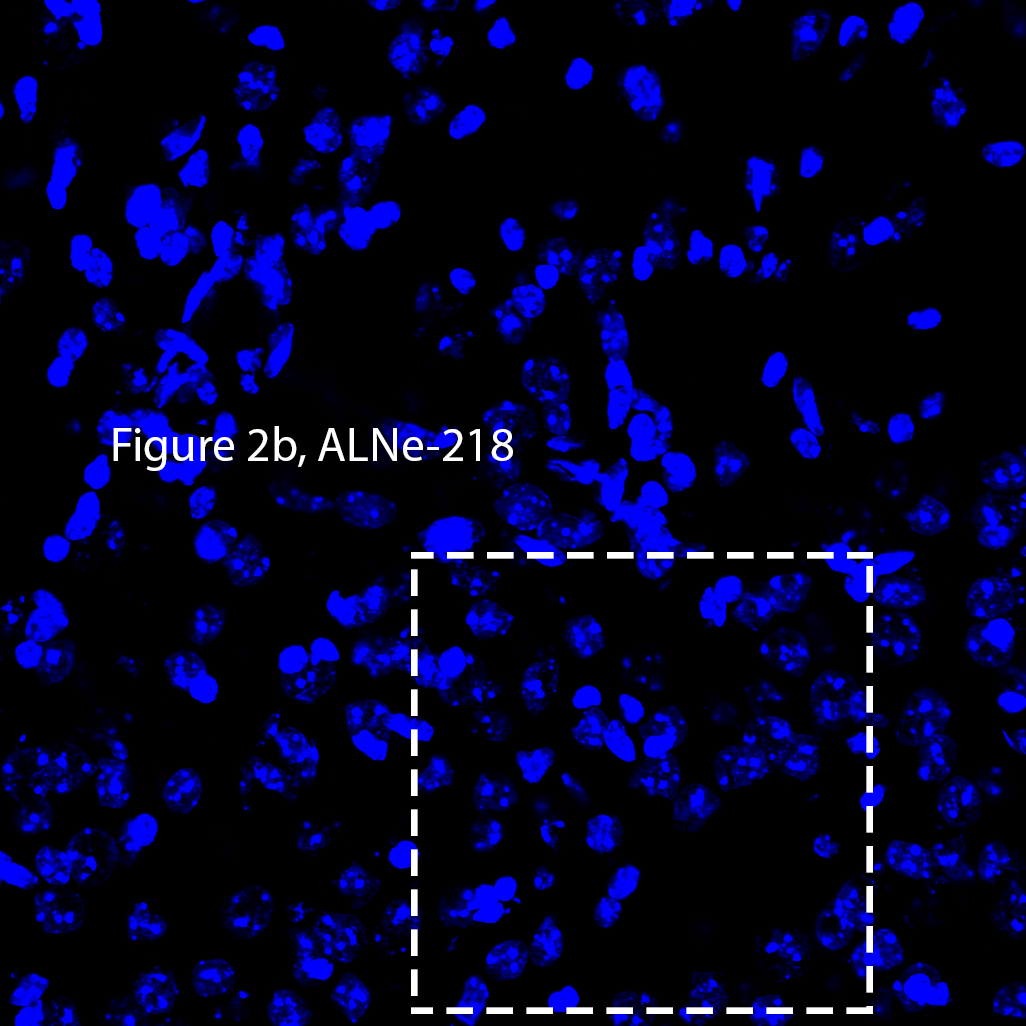

Supplement: Supplementary file 4 — Source Data for Figure 2 [file EMMM-14-e14797-s002.zip › Figure2b_ALNe-218_DAPI.tif]

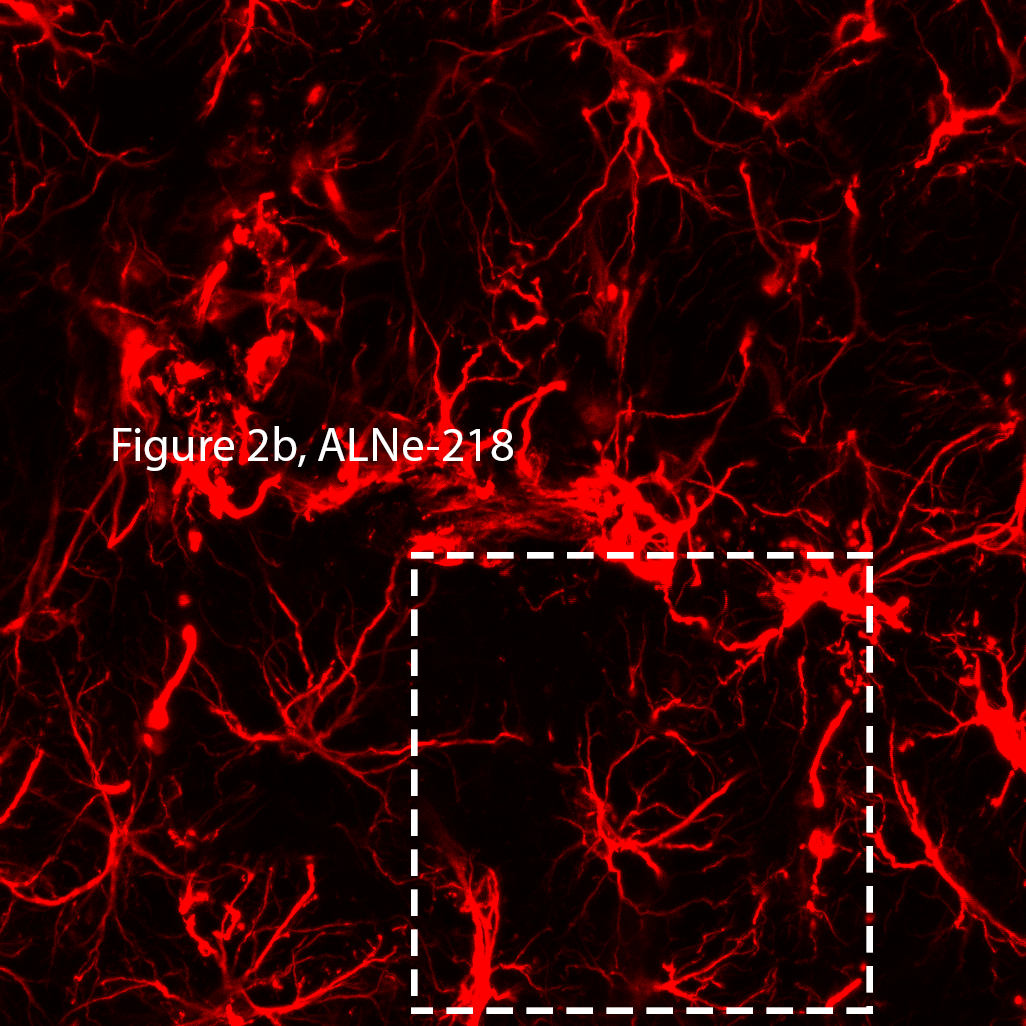

Supplement: Supplementary file 4 — Source Data for Figure 2 [file EMMM-14-e14797-s002.zip › Figure2b_ALNe-218_GFAP.tif]

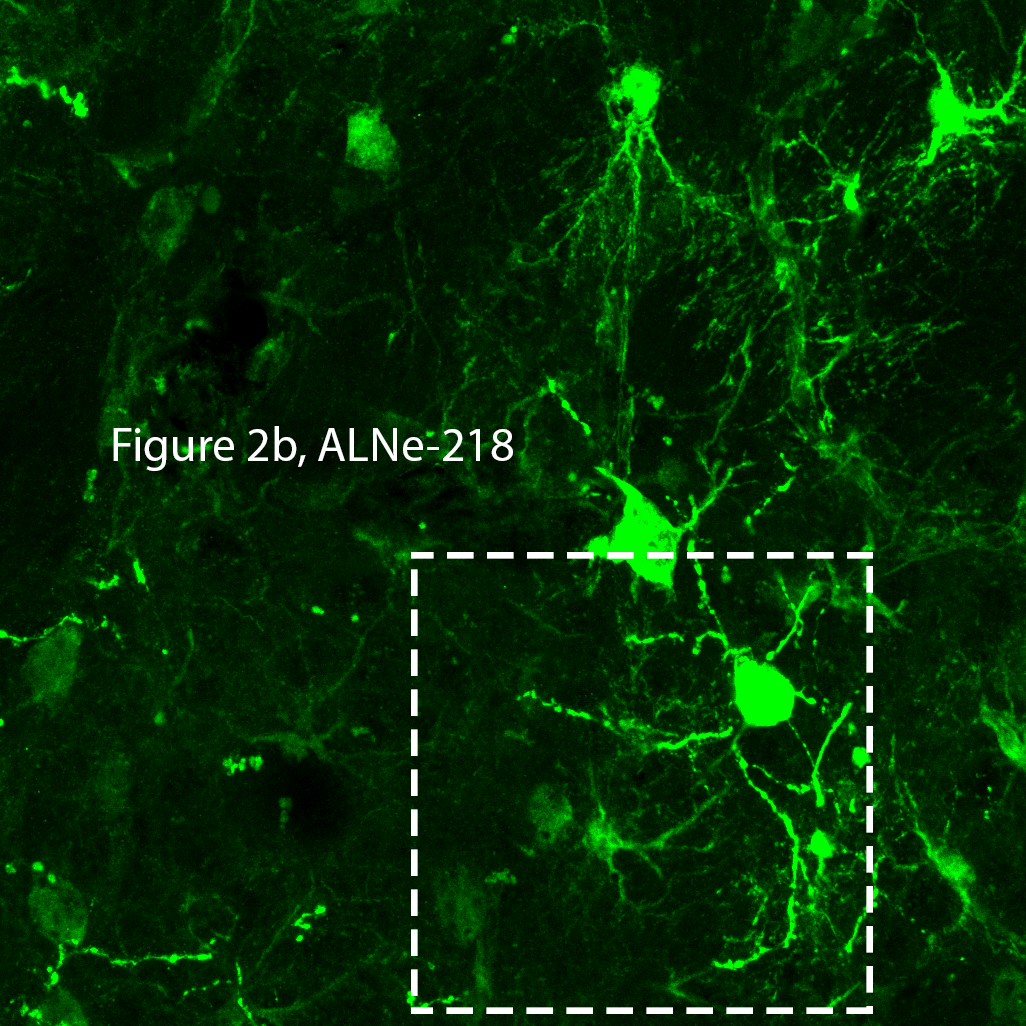

Supplement: Supplementary file 4 — Source Data for Figure 2 [file EMMM-14-e14797-s002.zip › Figure2b_ALNe-218_GFP.tif]

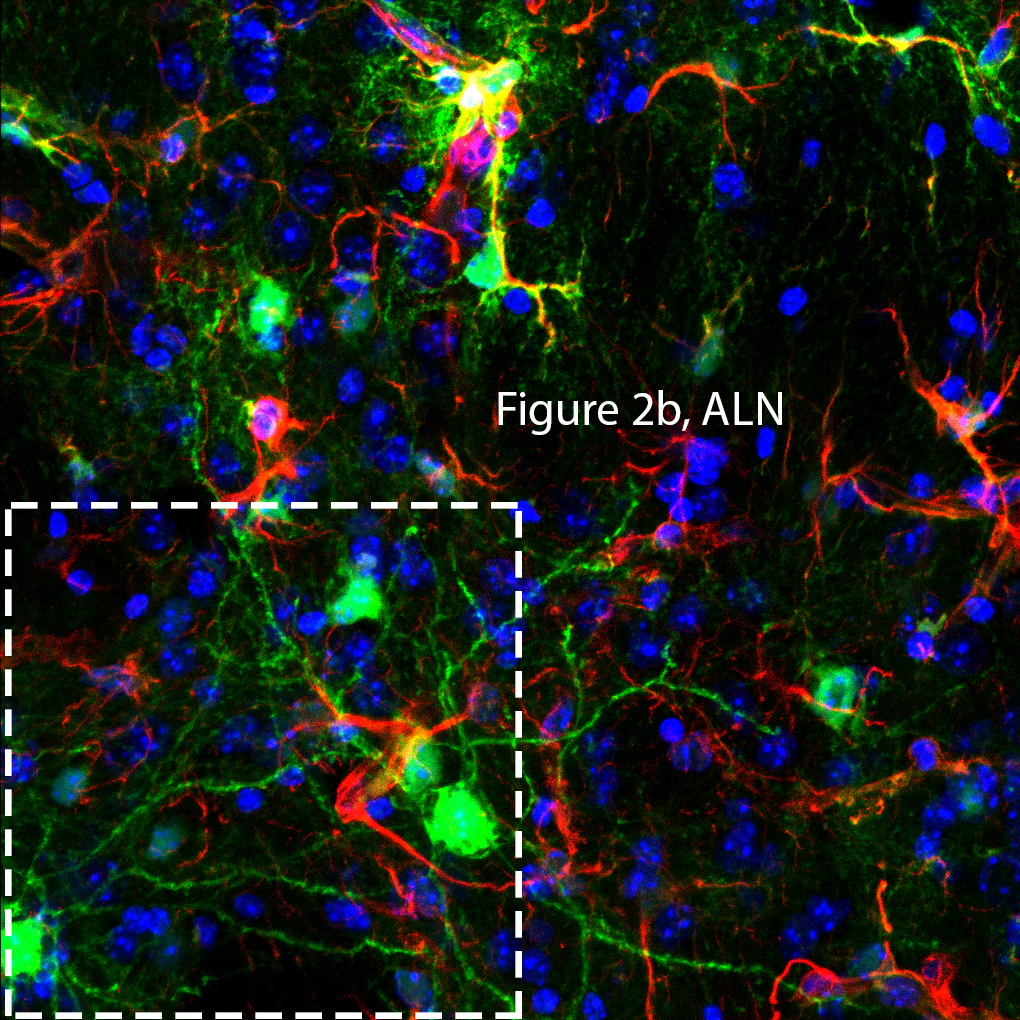

Supplement: Supplementary file 4 — Source Data for Figure 2 [file EMMM-14-e14797-s002.zip › Figure2b_ALN_composite.tif]

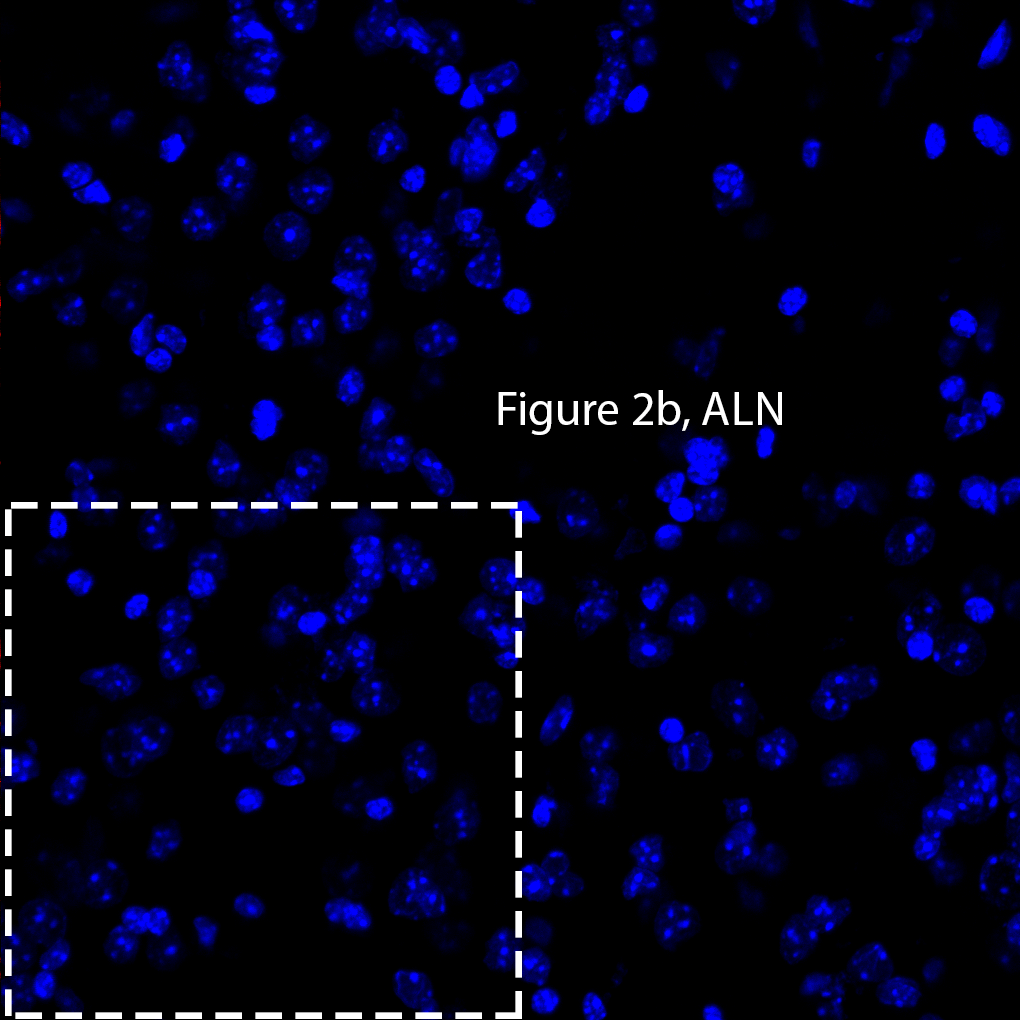

Supplement: Supplementary file 4 — Source Data for Figure 2 [file EMMM-14-e14797-s002.zip › Figure2b_ALN_DAPI.tif]

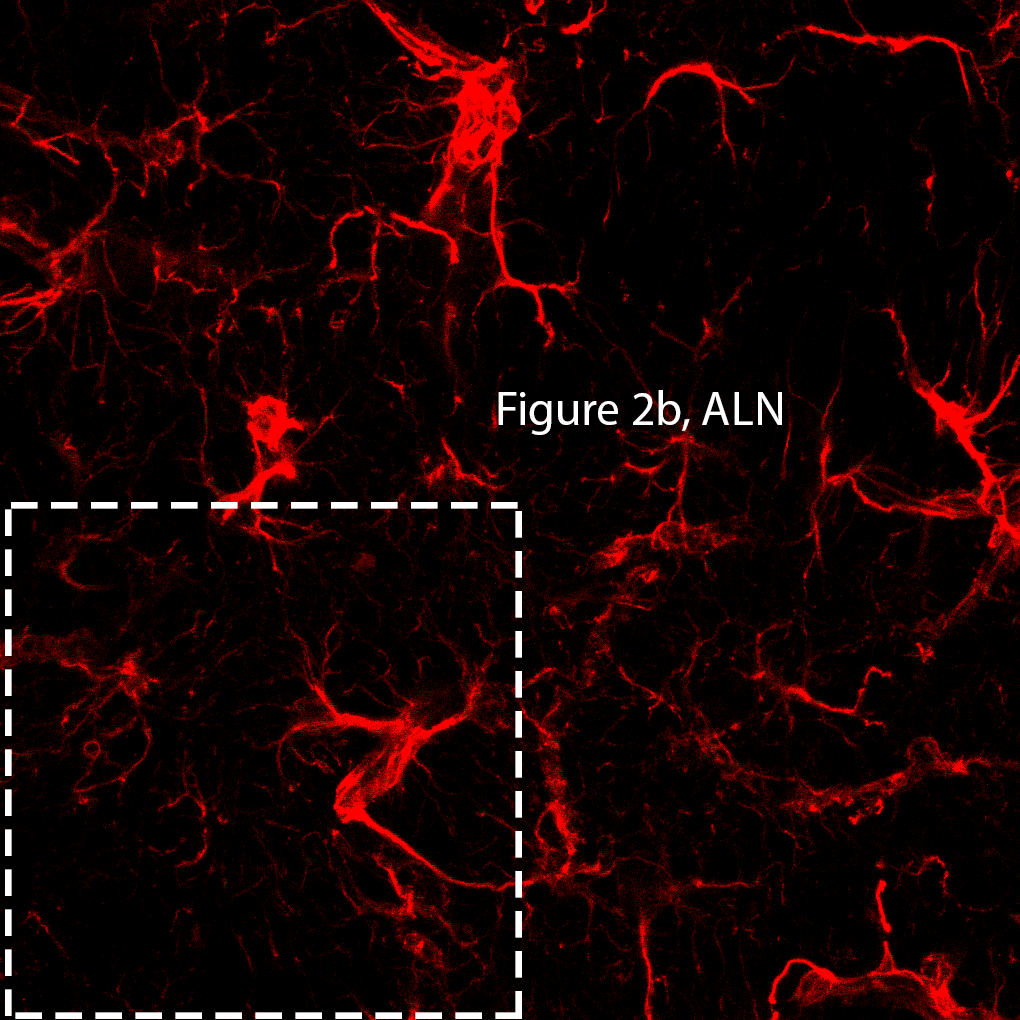

Supplement: Supplementary file 4 — Source Data for Figure 2 [file EMMM-14-e14797-s002.zip › Figure2b_ALN_GFAP.tif]

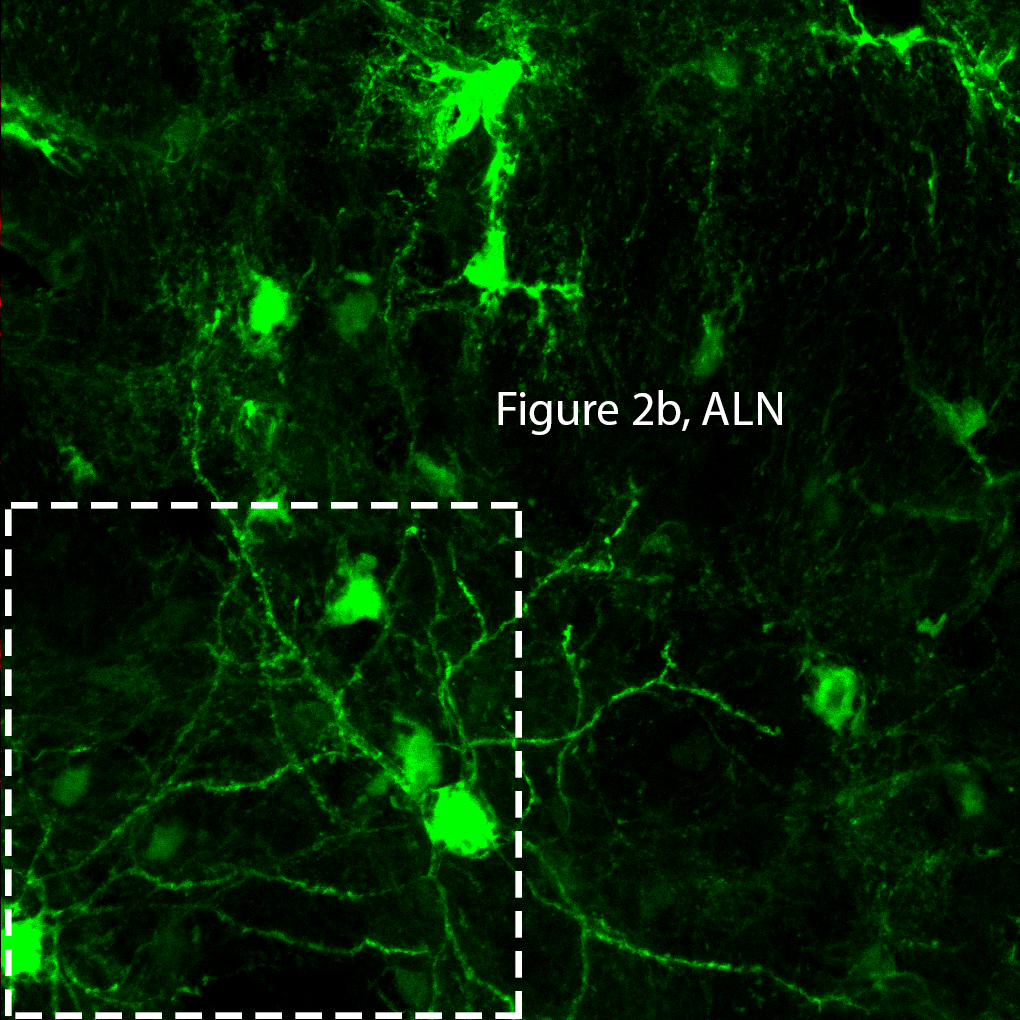

Supplement: Supplementary file 4 — Source Data for Figure 2 [file EMMM-14-e14797-s002.zip › Figure2b_ALN_GFP.tif]

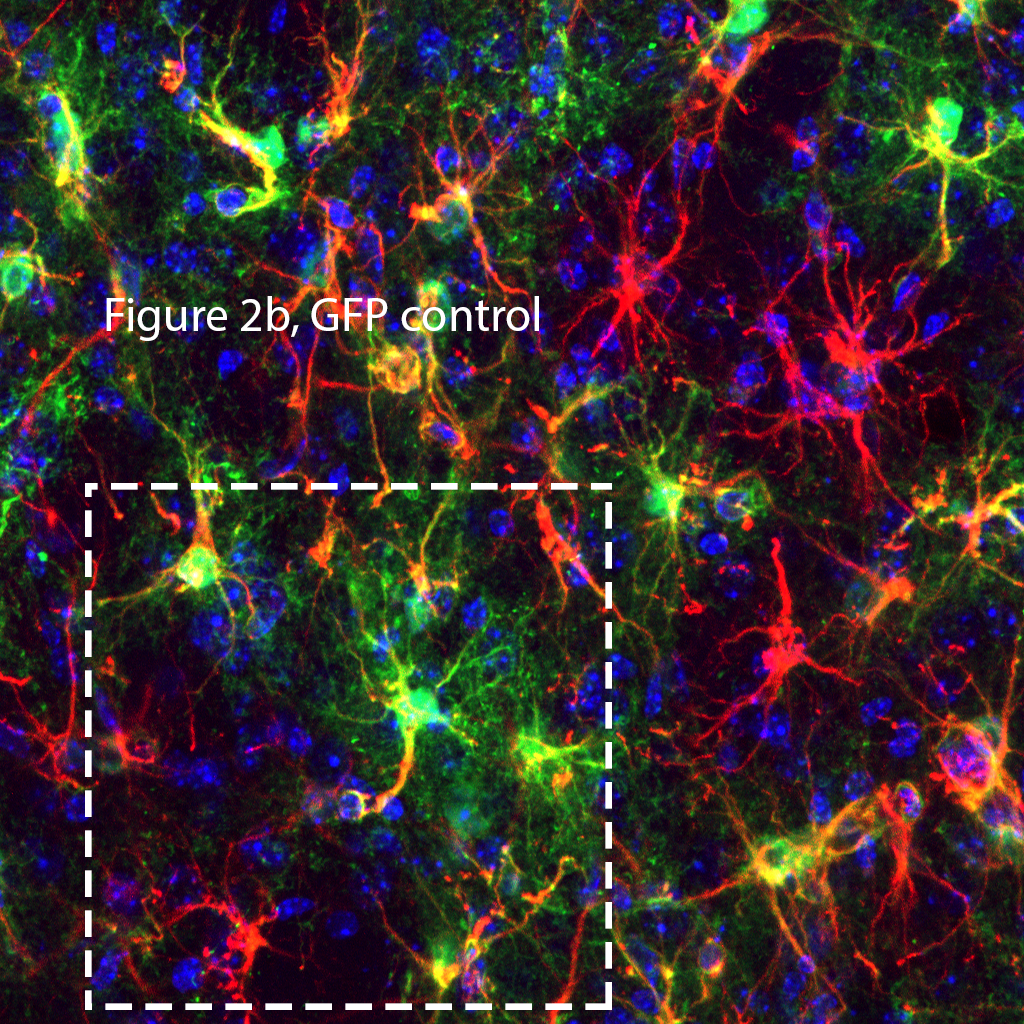

Supplement: Supplementary file 4 — Source Data for Figure 2 [file EMMM-14-e14797-s002.zip › Figure2b_GFPcontrol_composite.tif]

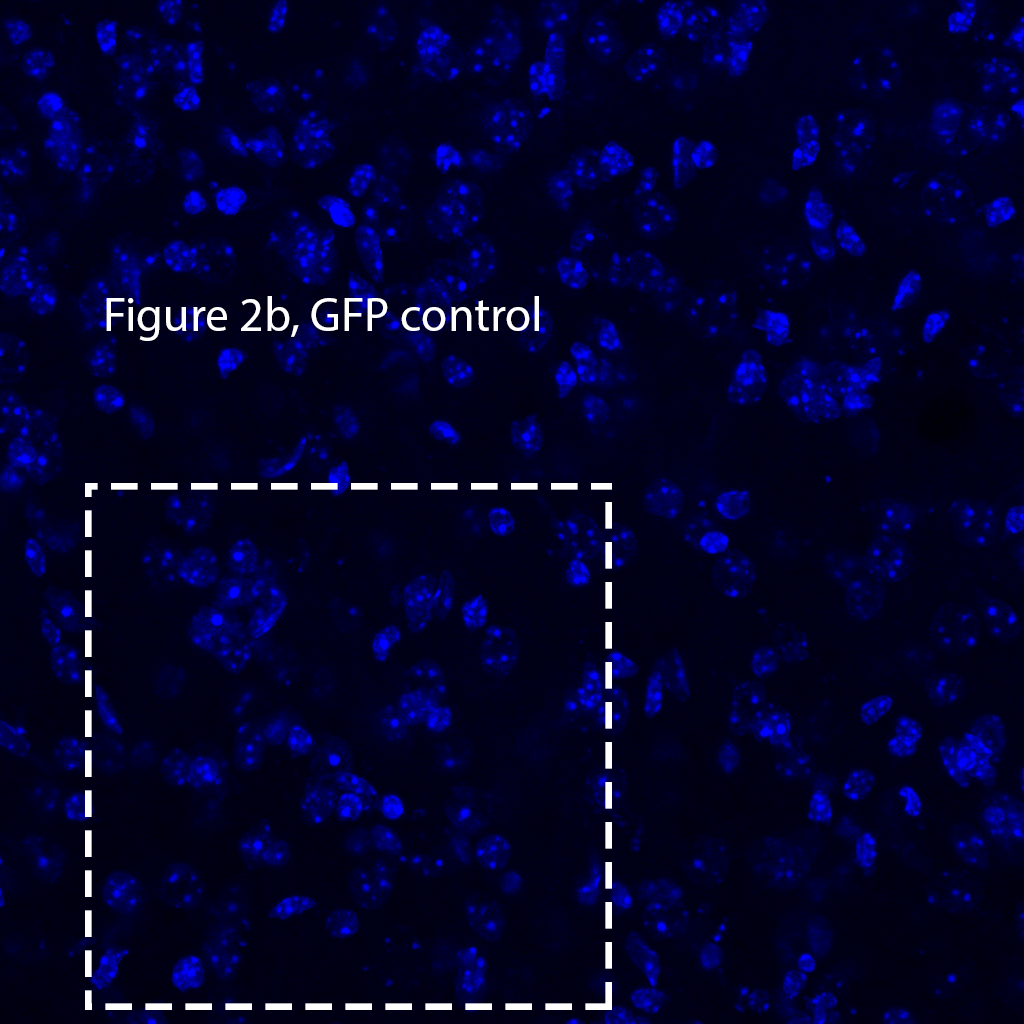

Supplement: Supplementary file 4 — Source Data for Figure 2 [file EMMM-14-e14797-s002.zip › Figure2b_GFPcontrol_DAPI.tif]

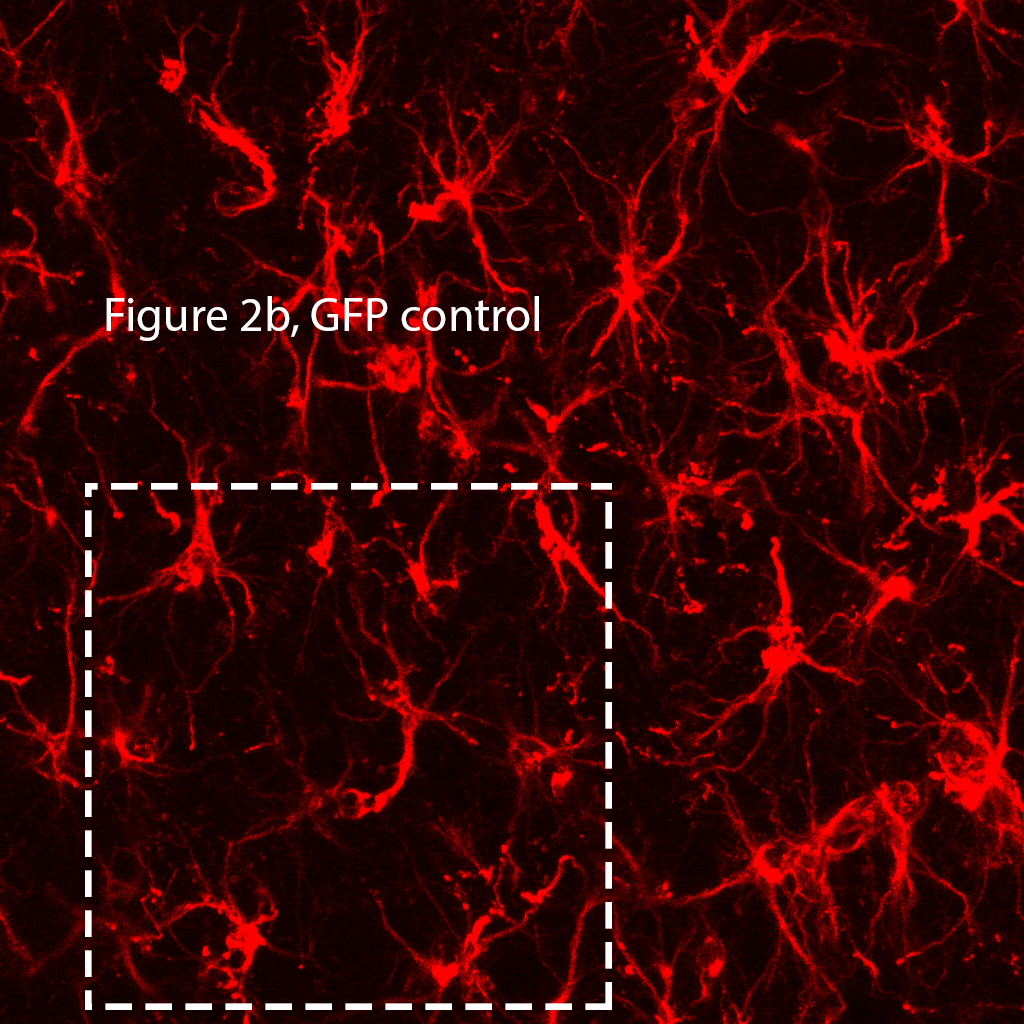

Supplement: Supplementary file 4 — Source Data for Figure 2 [file EMMM-14-e14797-s002.zip › Figure2b_GFPcontrol_GFAP.tif]

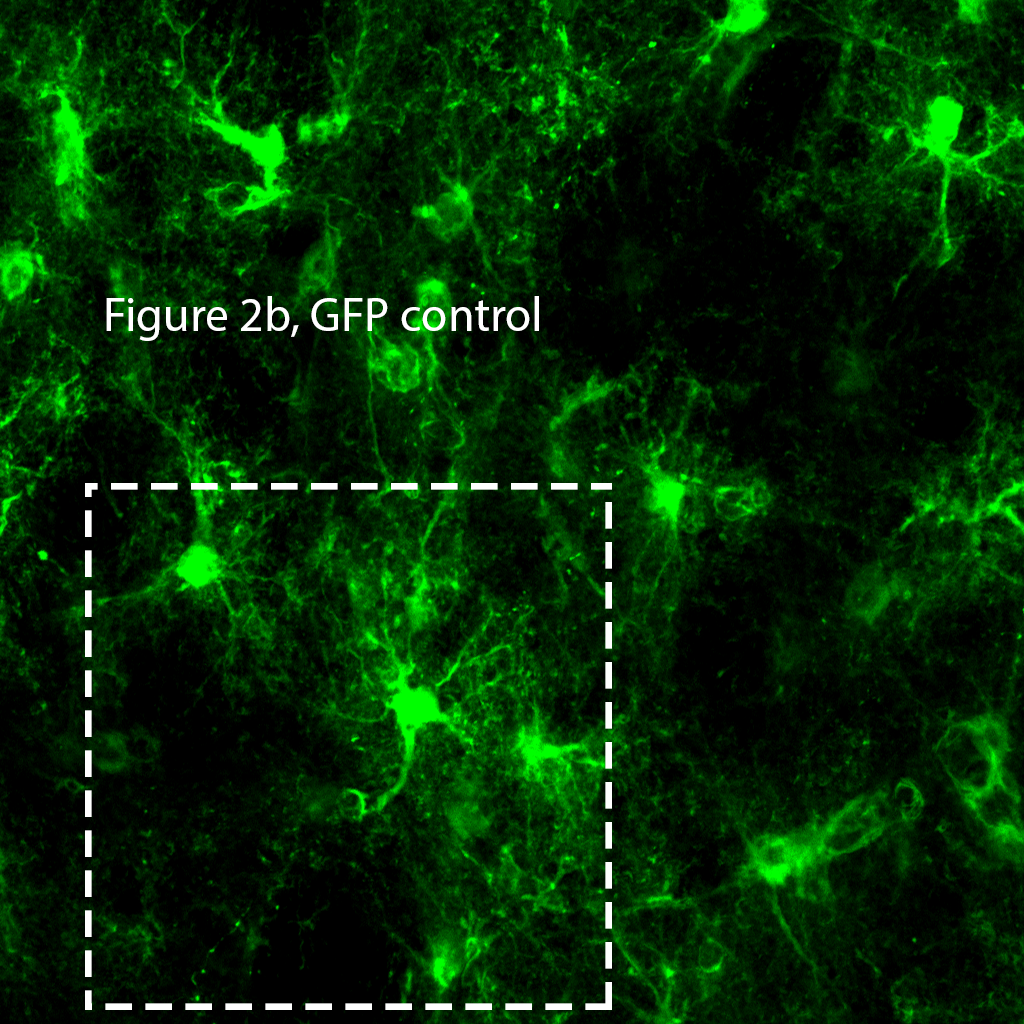

Supplement: Supplementary file 4 — Source Data for Figure 2 [file EMMM-14-e14797-s002.zip › Figure2b_GFPcontrol_GFP.tif]

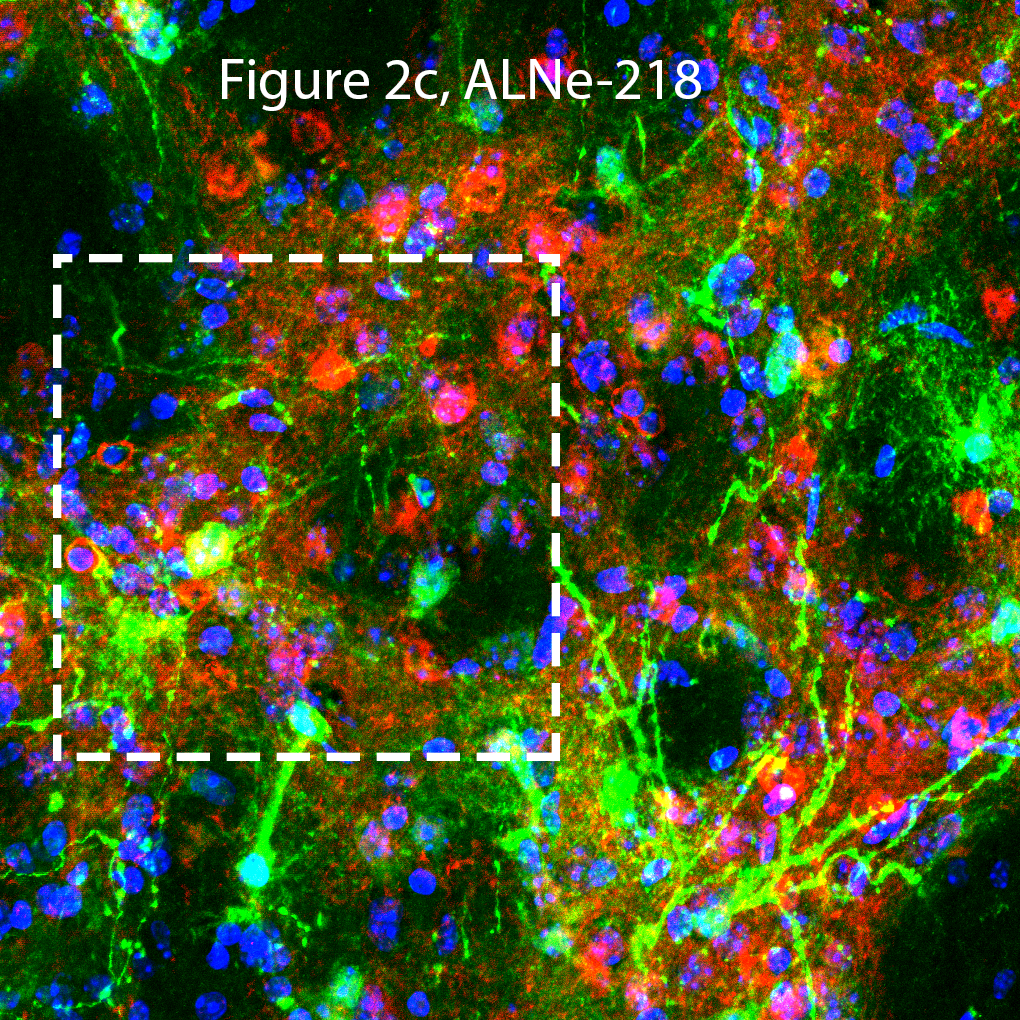

Supplement: Supplementary file 4 — Source Data for Figure 2 [file EMMM-14-e14797-s002.zip › Figure2c_ALNe-218_composite.tif]

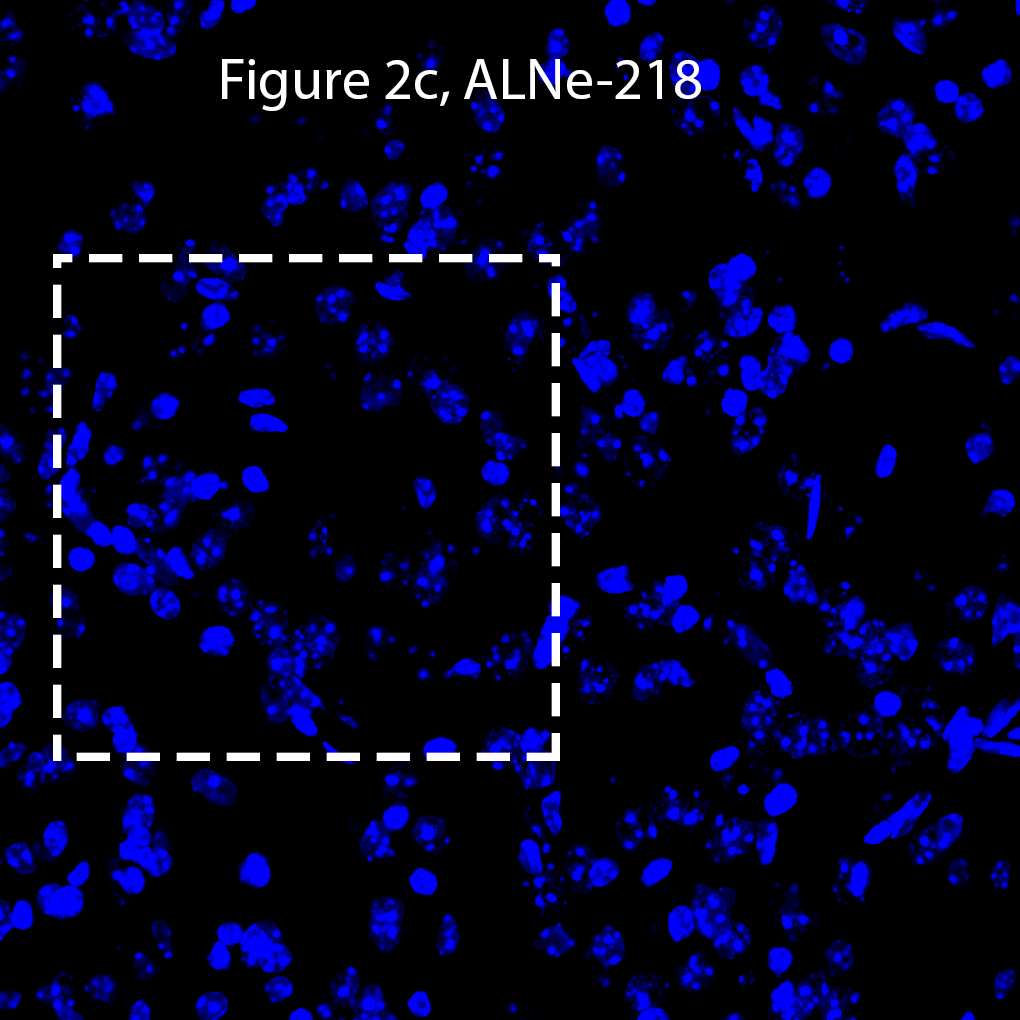

Supplement: Supplementary file 4 — Source Data for Figure 2 [file EMMM-14-e14797-s002.zip › Figure2c_ALNe-218_DAPI.tif]

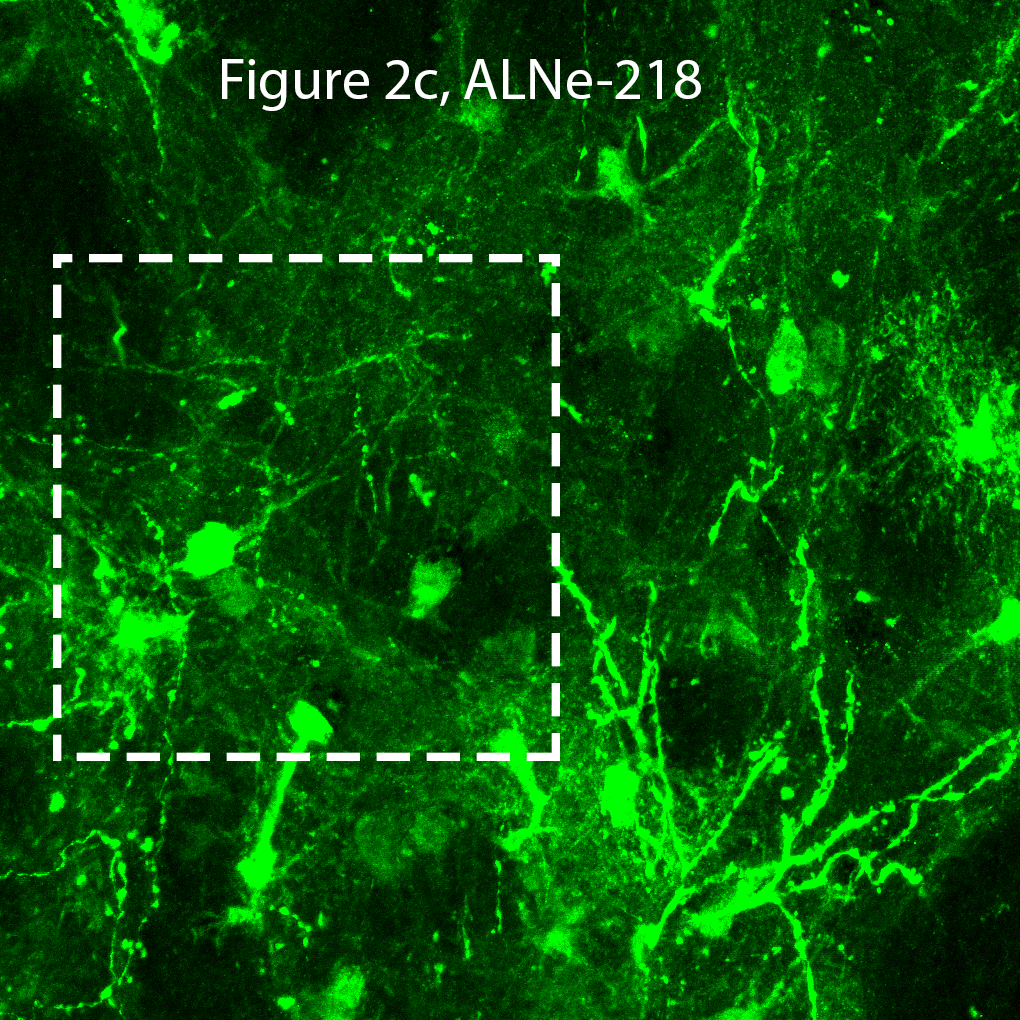

Supplement: Supplementary file 4 — Source Data for Figure 2 [file EMMM-14-e14797-s002.zip › Figure2c_ALNe-218_GFP.tif]

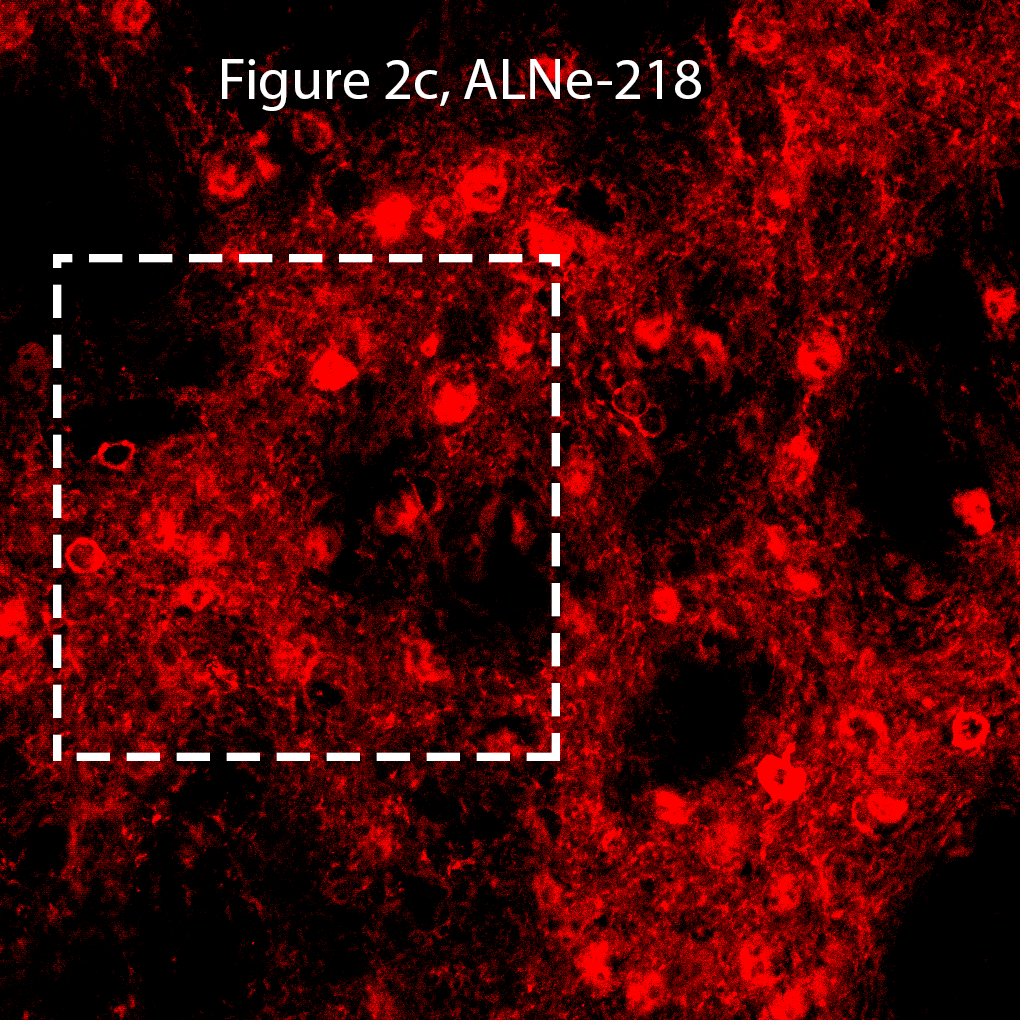

Supplement: Supplementary file 4 — Source Data for Figure 2 [file EMMM-14-e14797-s002.zip › Figure2c_ALNe-218_NeuN.tif]

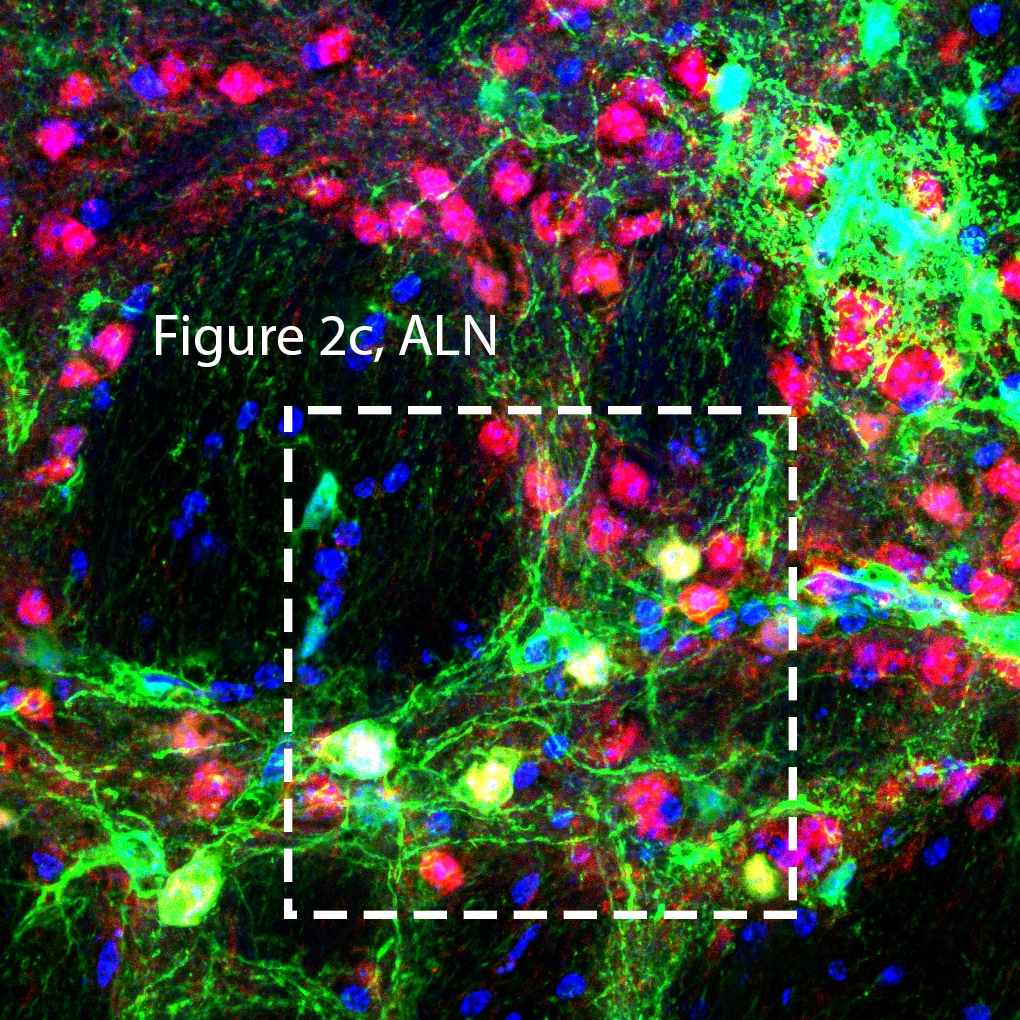

Supplement: Supplementary file 4 — Source Data for Figure 2 [file EMMM-14-e14797-s002.zip › Figure2c_ALN_composite.tif]

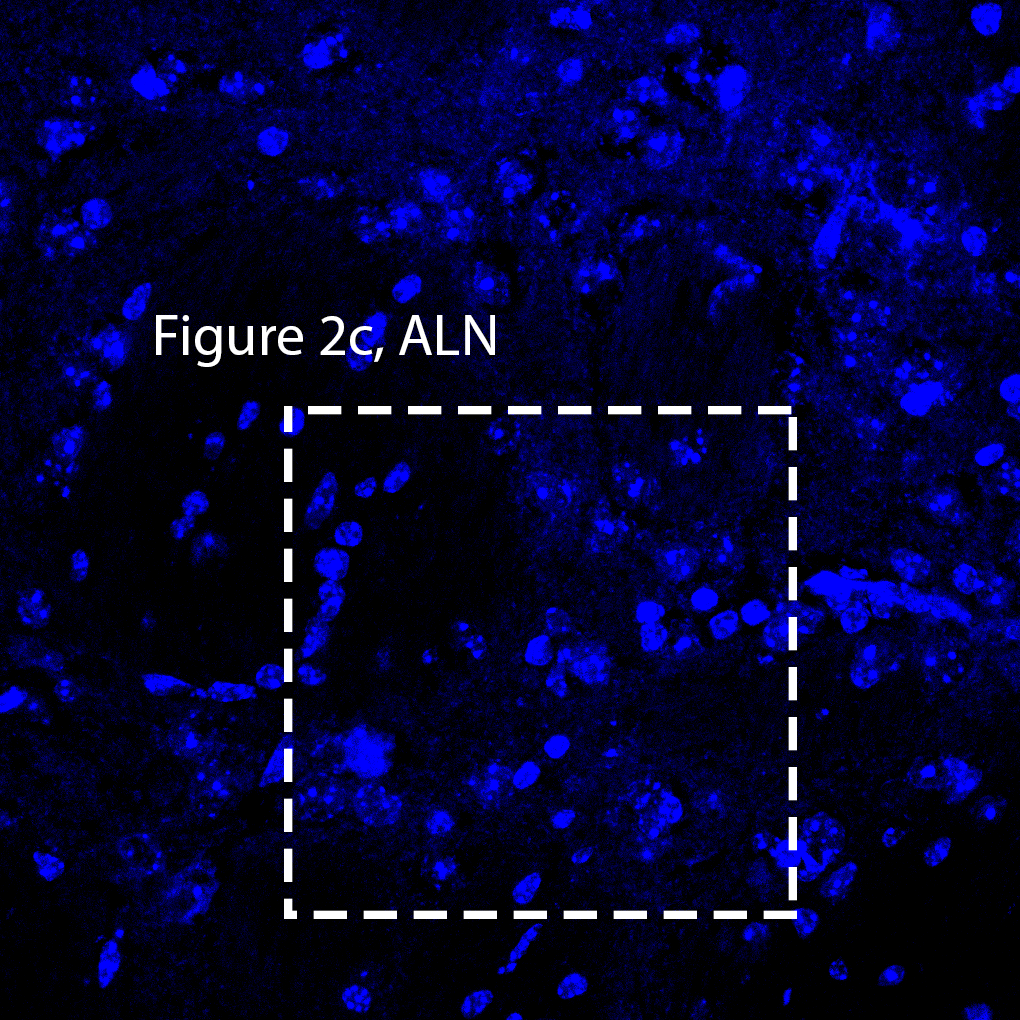

Supplement: Supplementary file 4 — Source Data for Figure 2 [file EMMM-14-e14797-s002.zip › Figure2c_ALN_DAPI.tif]

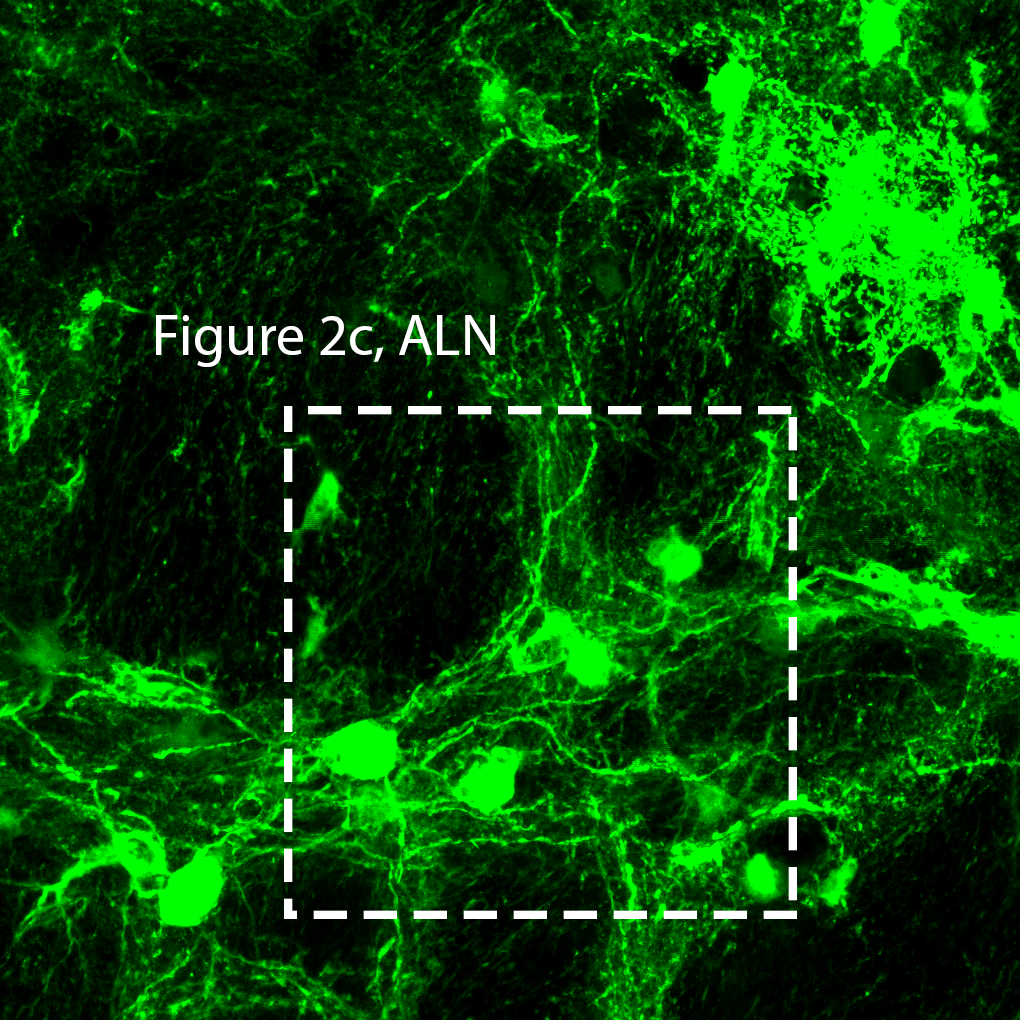

Supplement: Supplementary file 4 — Source Data for Figure 2 [file EMMM-14-e14797-s002.zip › Figure2c_ALN_GFP.tif]

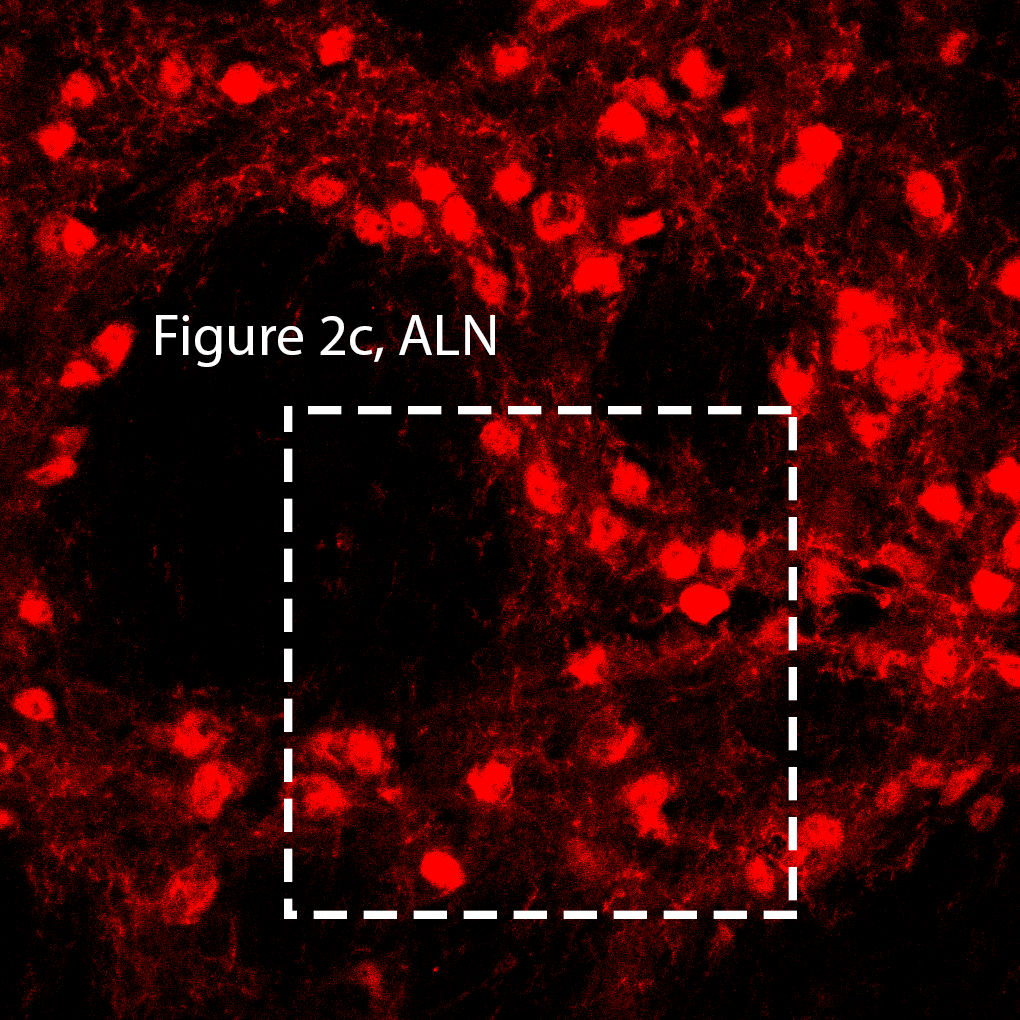

Supplement: Supplementary file 4 — Source Data for Figure 2 [file EMMM-14-e14797-s002.zip › Figure2c_ALN_NeuN.tif]

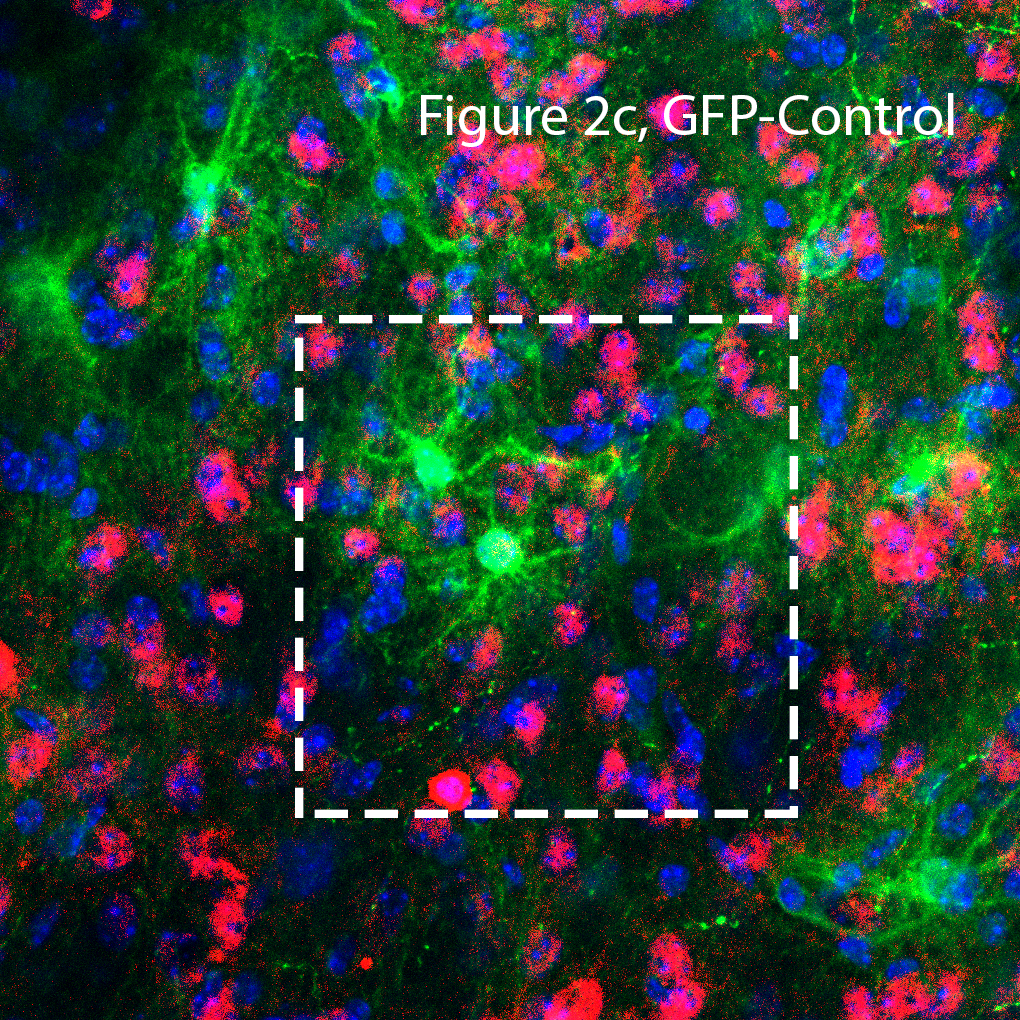

Supplement: Supplementary file 4 — Source Data for Figure 2 [file EMMM-14-e14797-s002.zip › Figure2c_GFPcontrol_composite.tif]

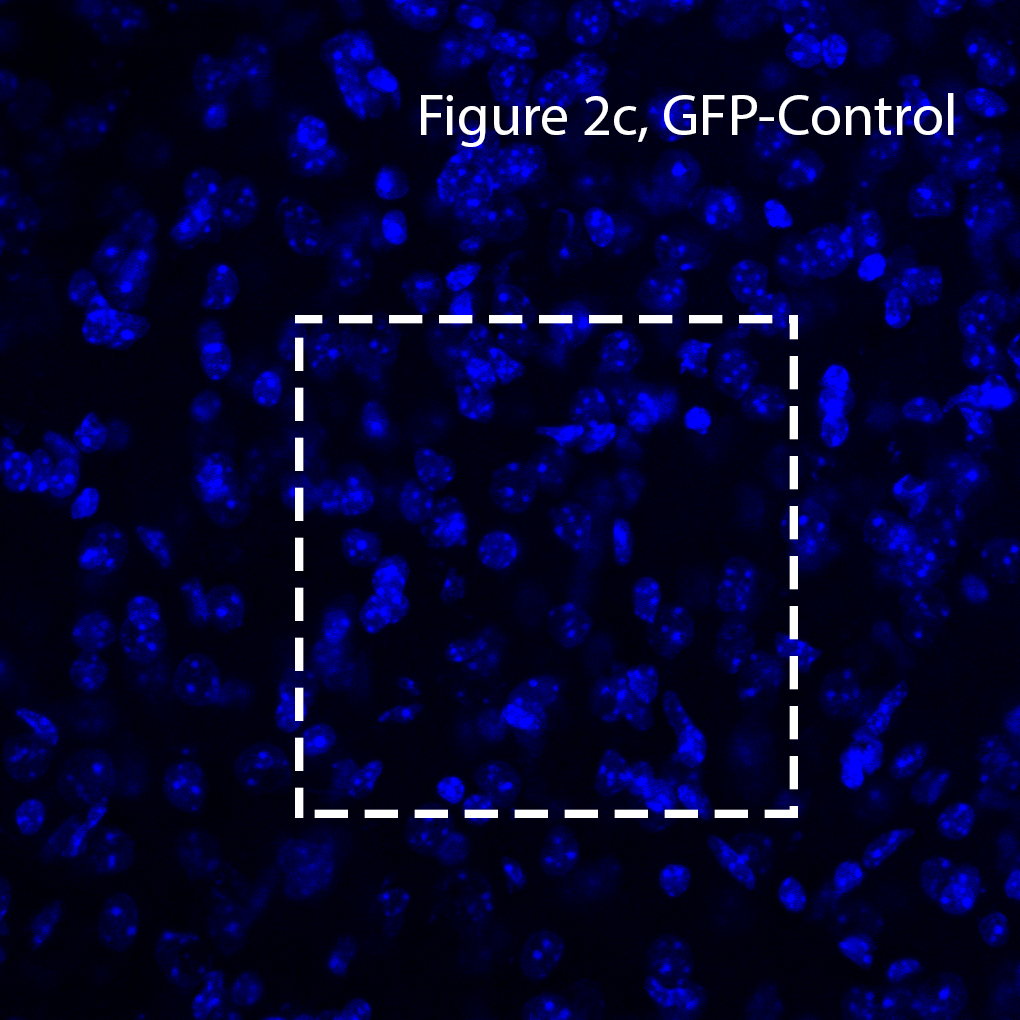

Supplement: Supplementary file 4 — Source Data for Figure 2 [file EMMM-14-e14797-s002.zip › Figure2c_GFPcontrol_DAPI.tif]

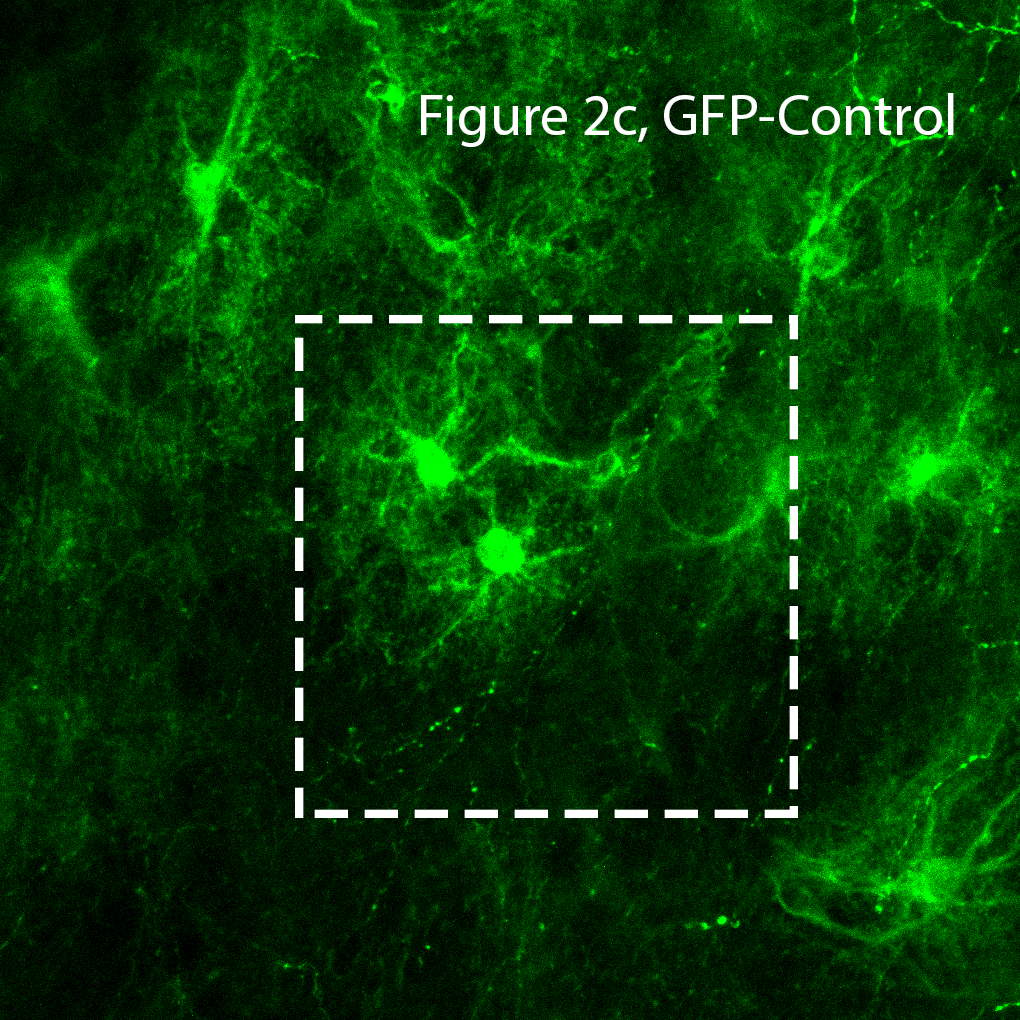

Supplement: Supplementary file 4 — Source Data for Figure 2 [file EMMM-14-e14797-s002.zip › Figure2c_GFPcontrol_GFP.tif]

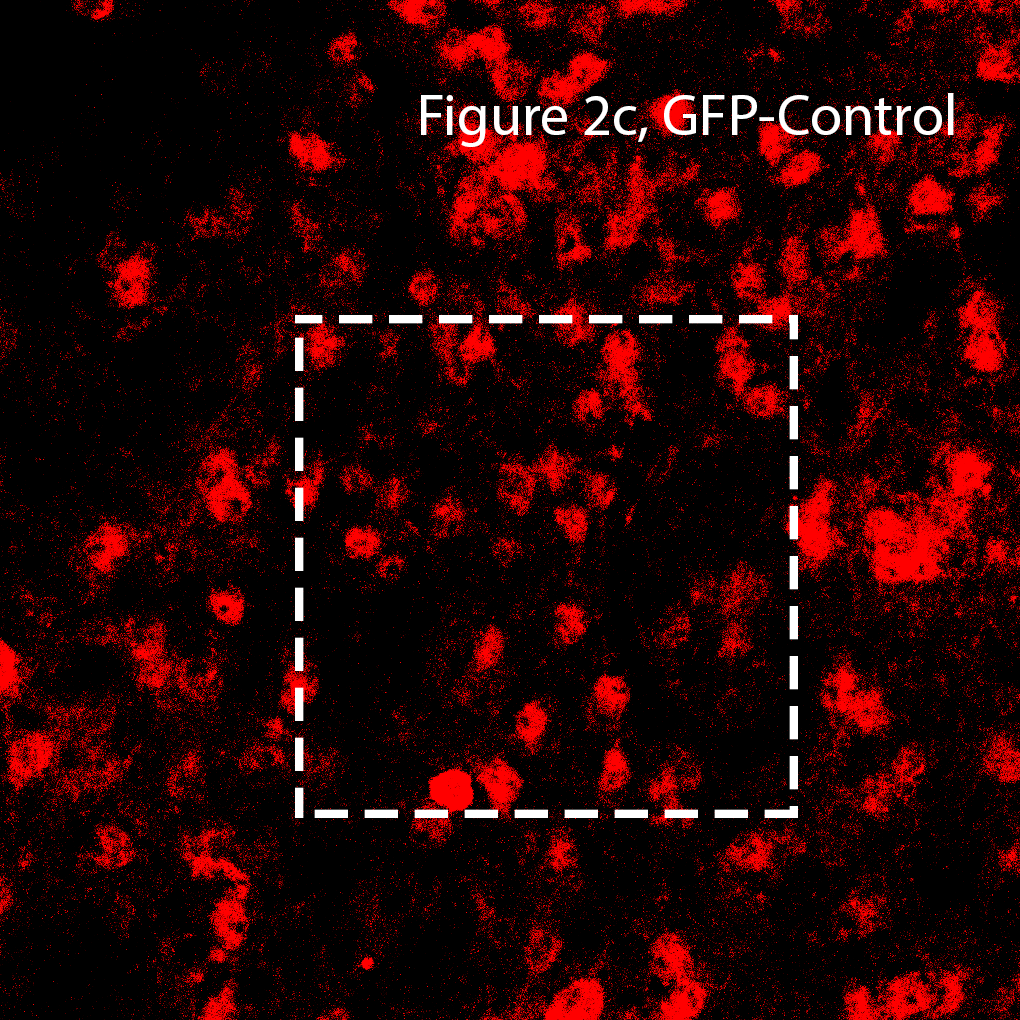

Supplement: Supplementary file 4 — Source Data for Figure 2 [file EMMM-14-e14797-s002.zip › Figure2c_GFPcontrol_NeuN.tif]

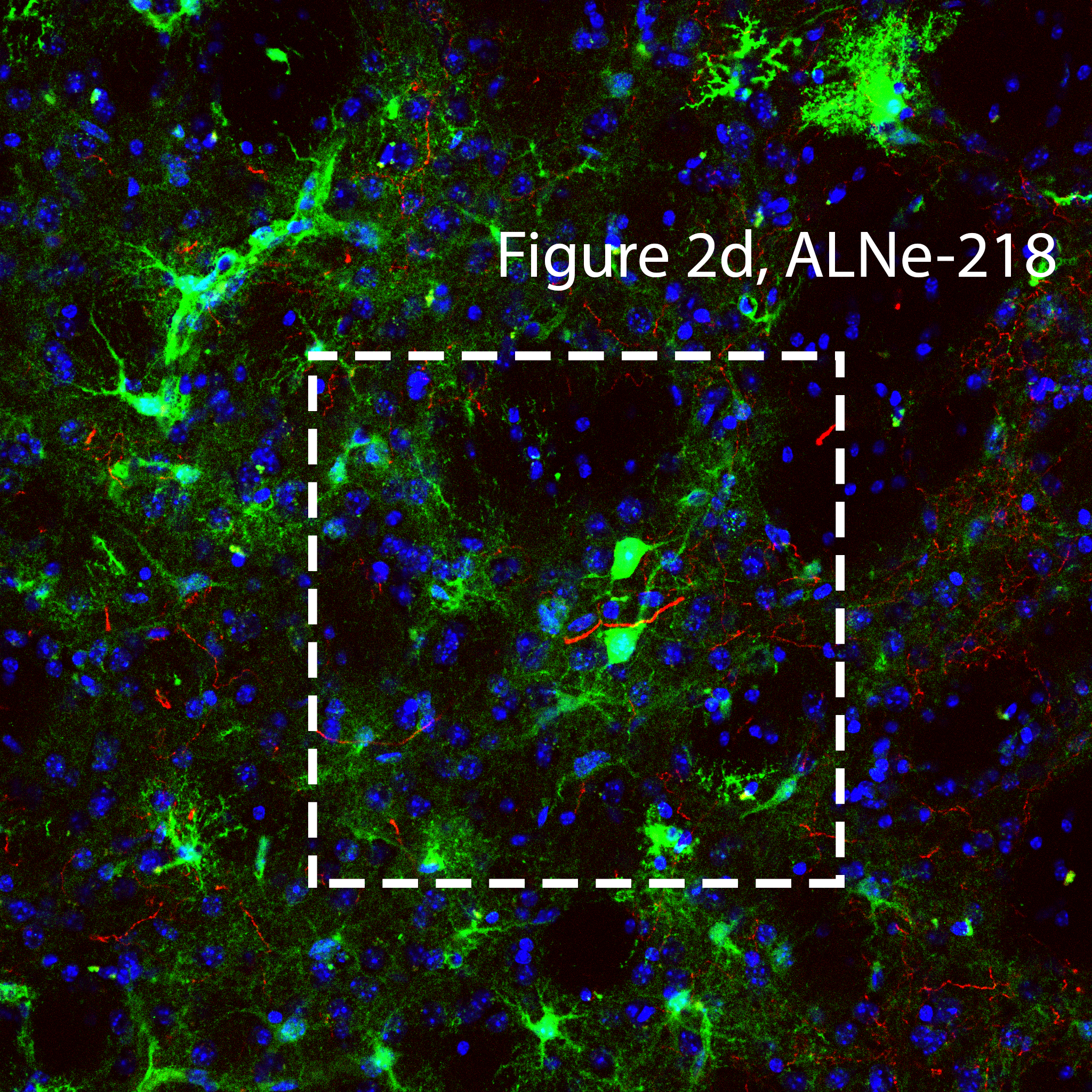

Supplement: Supplementary file 4 — Source Data for Figure 2 [file EMMM-14-e14797-s002.zip › Figure2d_ALNe-218_composite.tif]

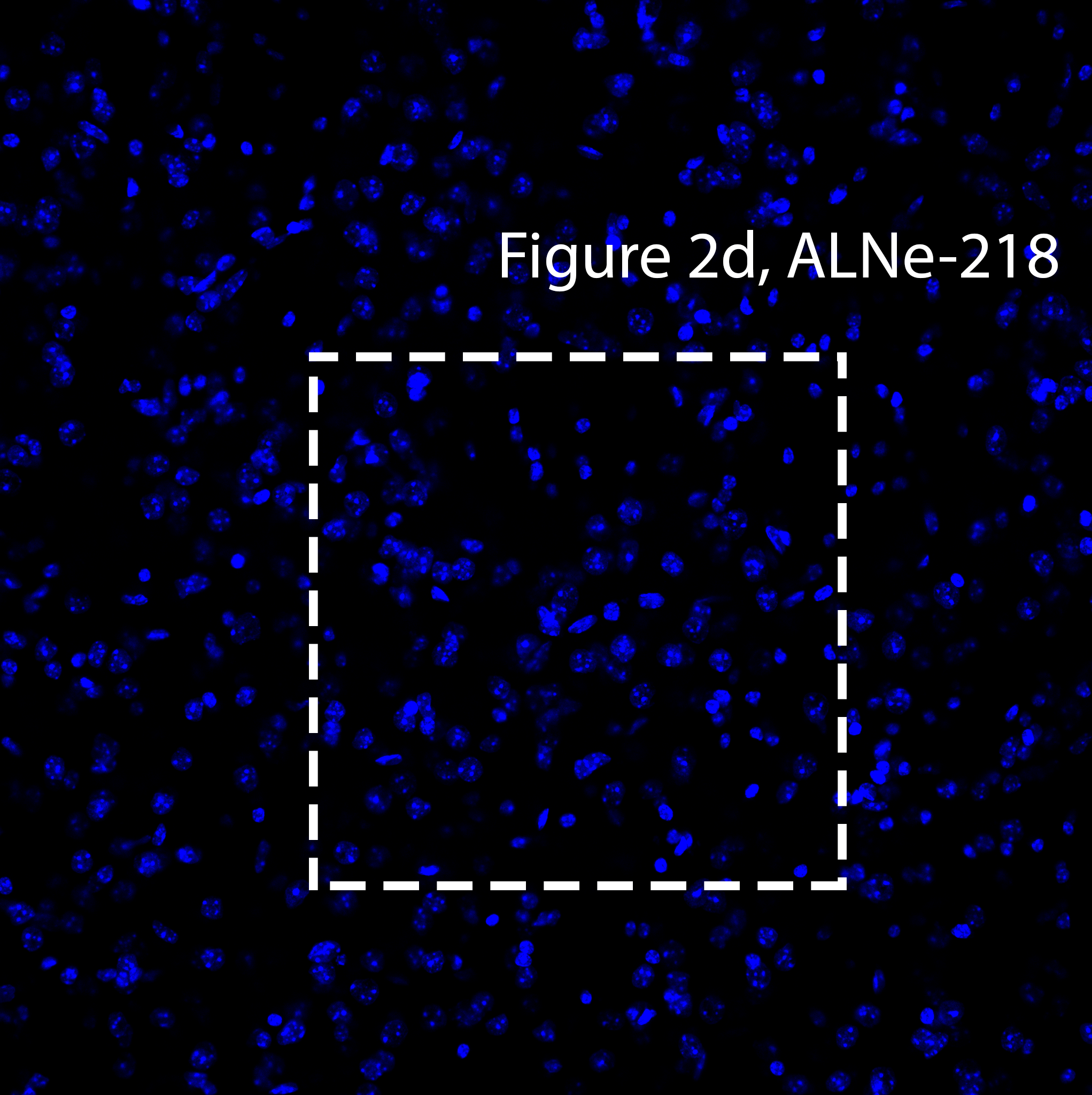

Supplement: Supplementary file 4 — Source Data for Figure 2 [file EMMM-14-e14797-s002.zip › Figure2d_ALNe-218_DAPI.tif]

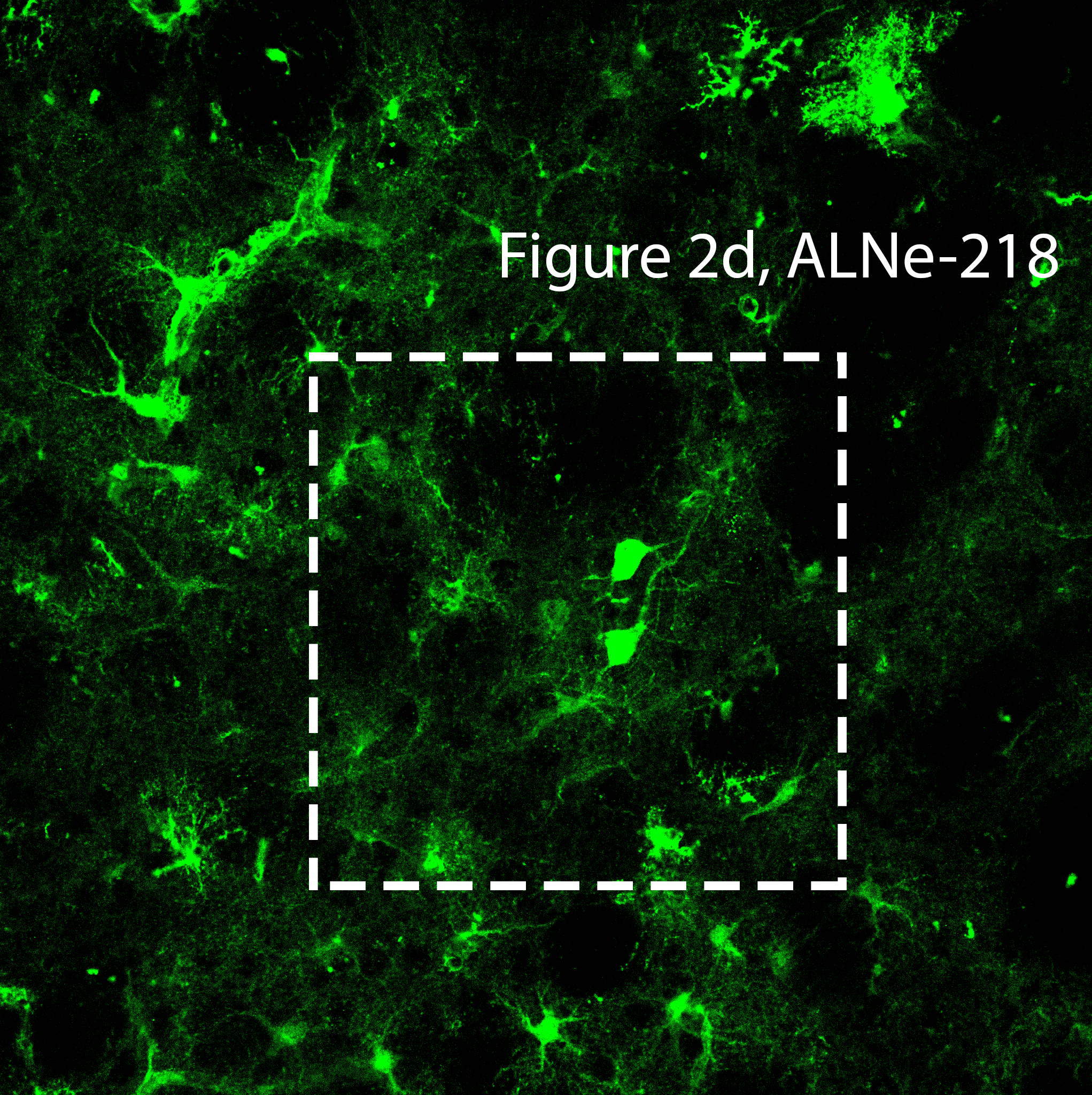

Supplement: Supplementary file 4 — Source Data for Figure 2 [file EMMM-14-e14797-s002.zip › Figure2d_ALNe-218_GFP.tif]

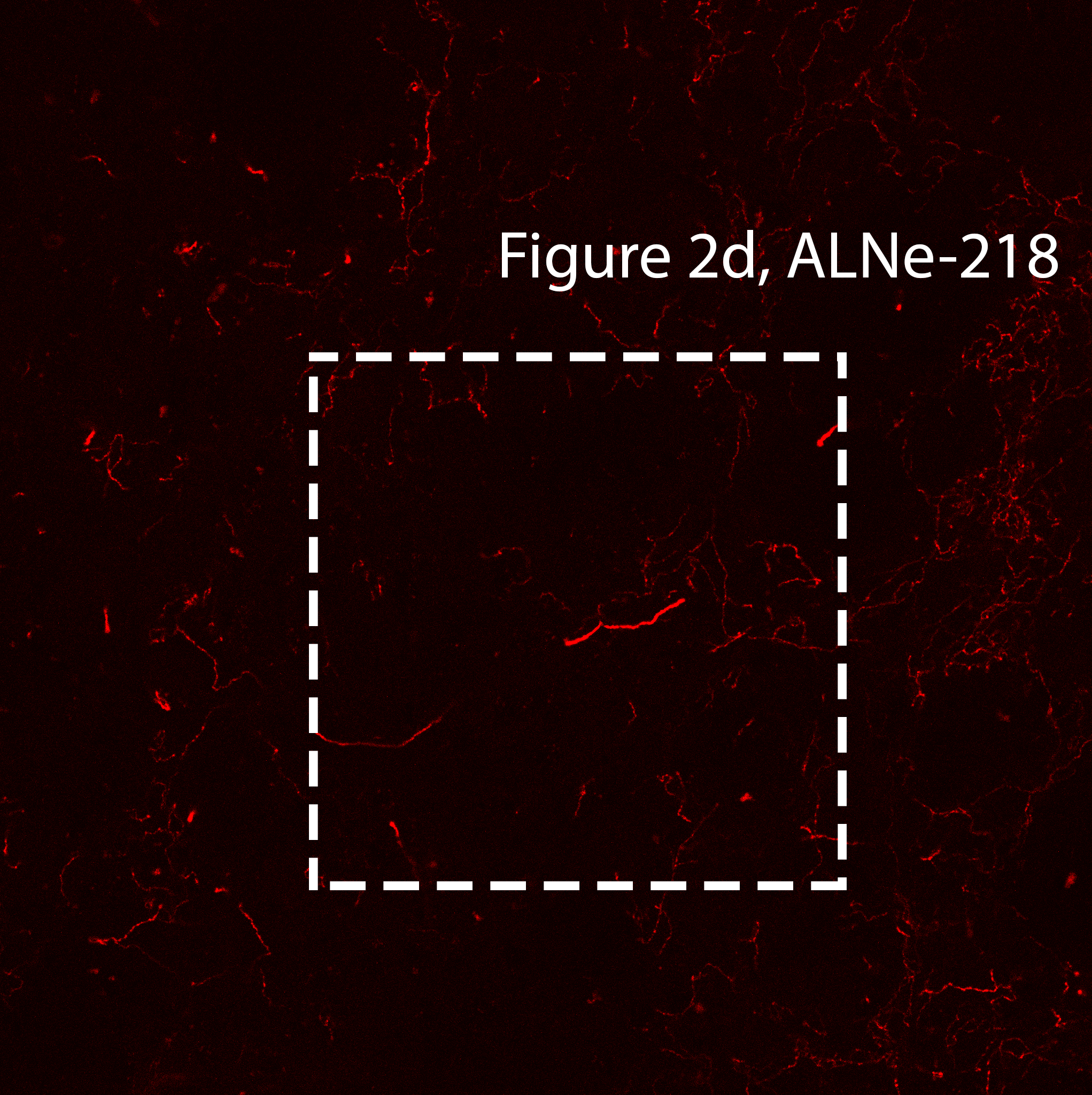

Supplement: Supplementary file 4 — Source Data for Figure 2 [file EMMM-14-e14797-s002.zip › Figure2d_ALNe-218_TH.tif]

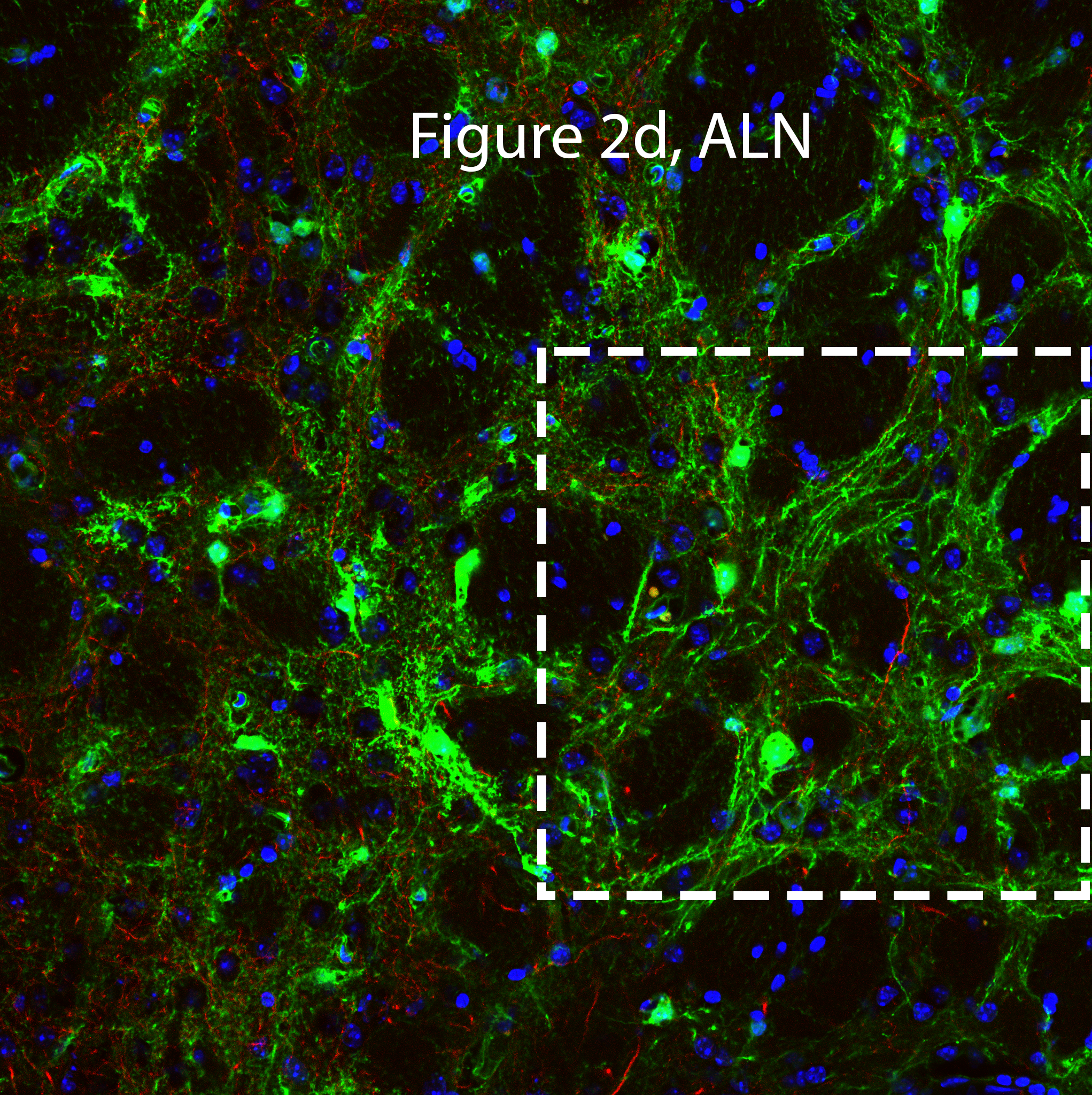

Supplement: Supplementary file 4 — Source Data for Figure 2 [file EMMM-14-e14797-s002.zip › Figure2d_ALN_composite.tif]

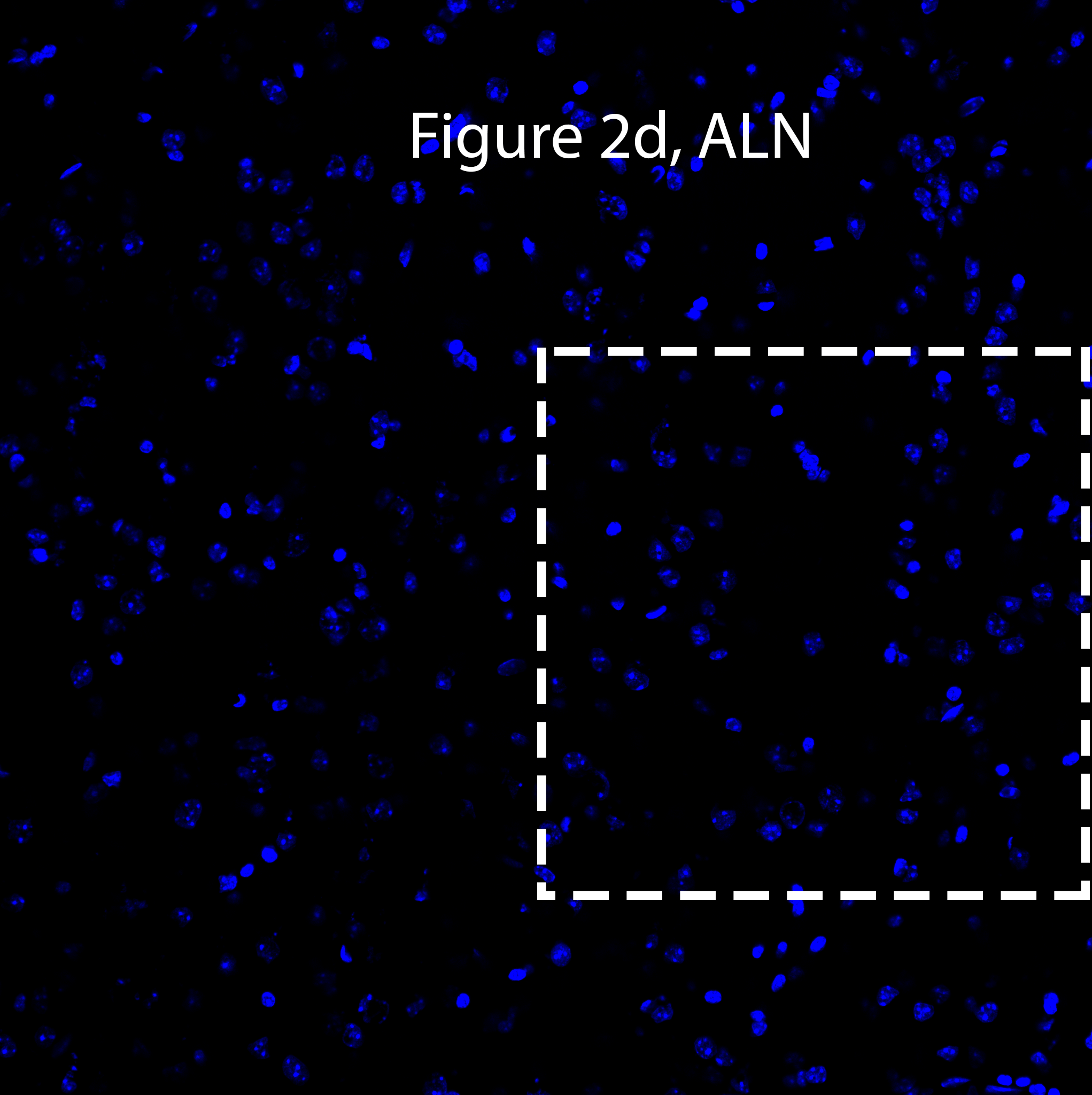

Supplement: Supplementary file 4 — Source Data for Figure 2 [file EMMM-14-e14797-s002.zip › Figure2d_ALN_DAPI.tif]

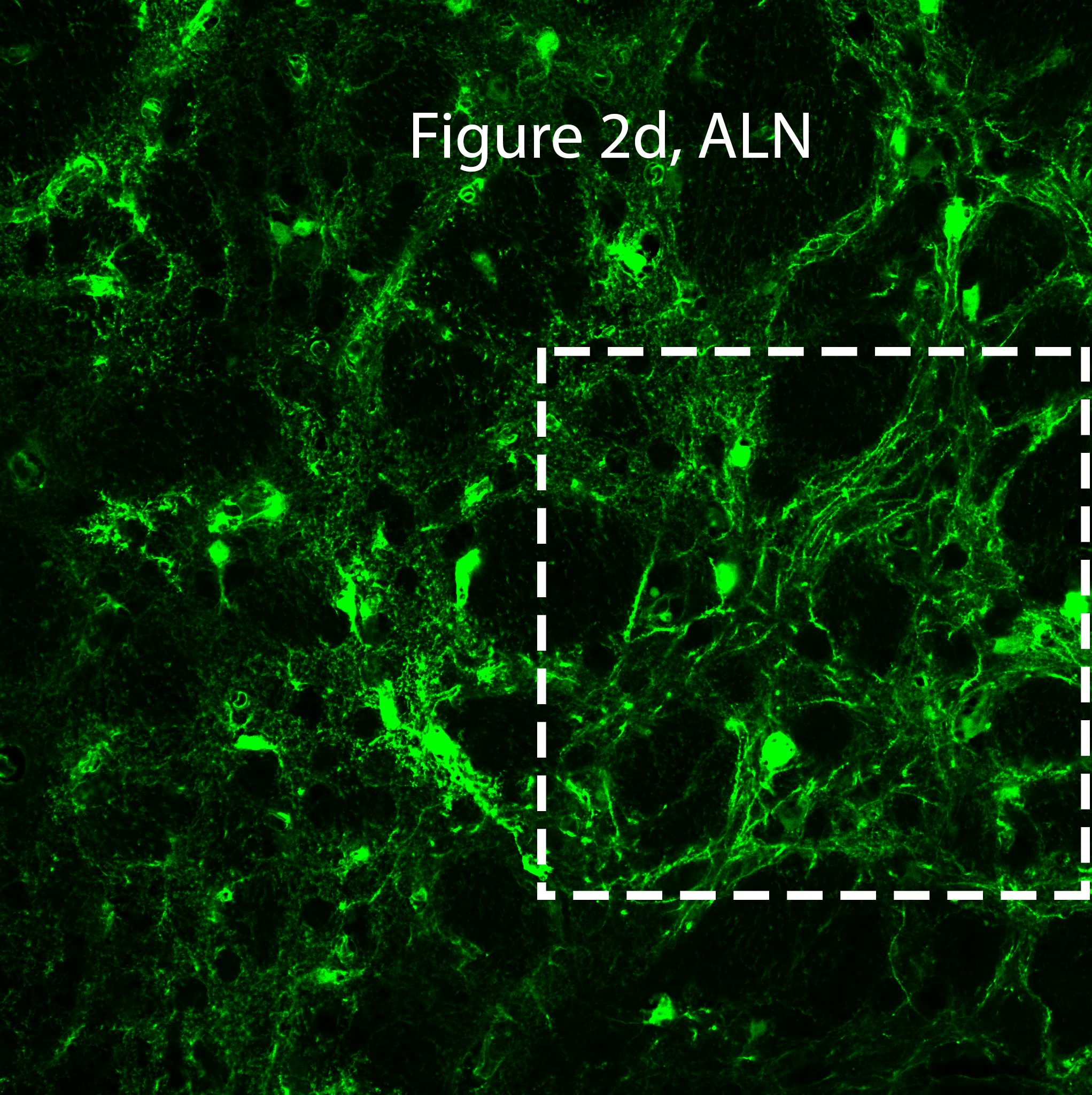

Supplement: Supplementary file 4 — Source Data for Figure 2 [file EMMM-14-e14797-s002.zip › Figure2d_ALN_GFP.tif]

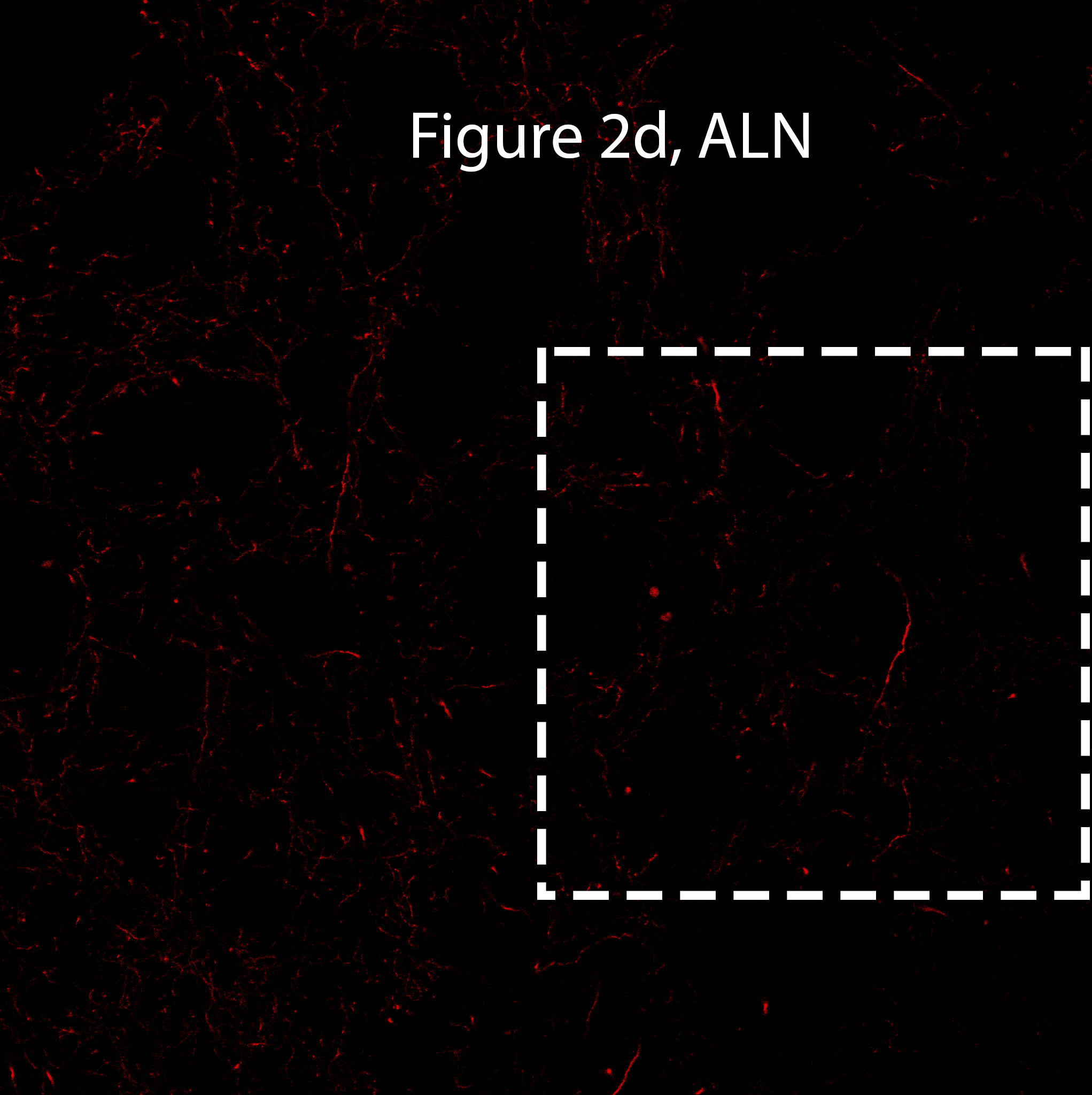

Supplement: Supplementary file 4 — Source Data for Figure 2 [file EMMM-14-e14797-s002.zip › Figure2d_ALN_TH.tif]

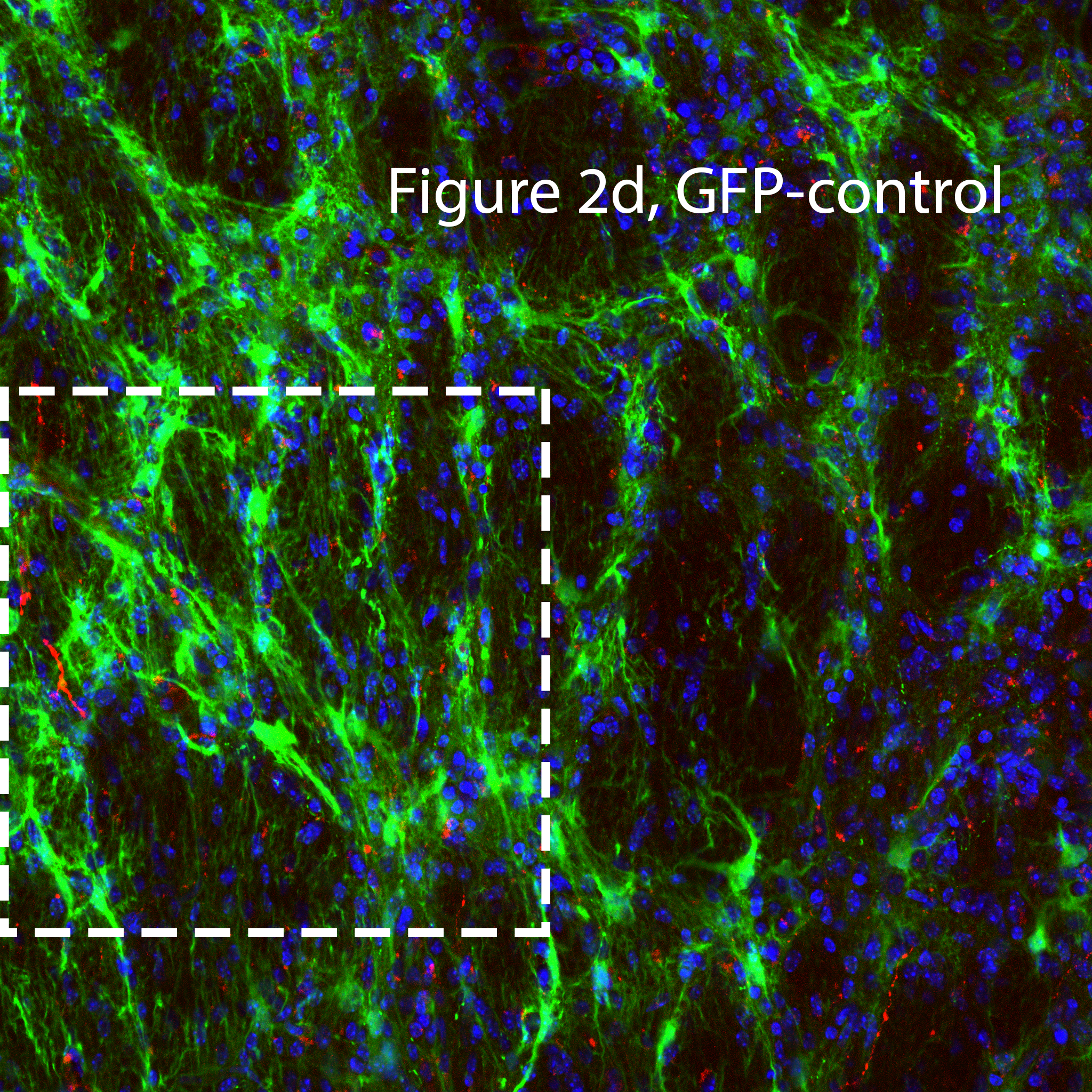

Supplement: Supplementary file 4 — Source Data for Figure 2 [file EMMM-14-e14797-s002.zip › Figure2d_GFPcontrol_composite.tif]

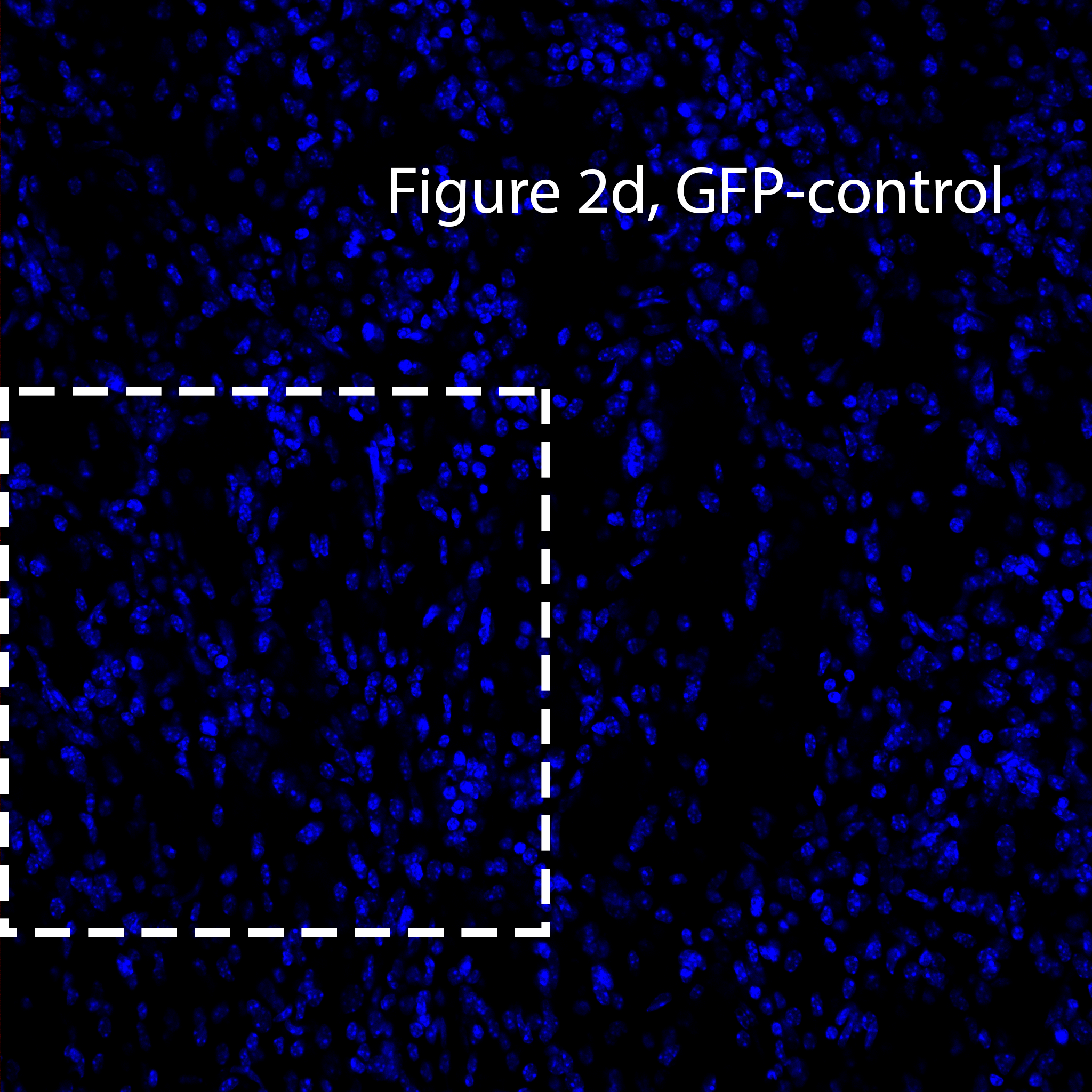

Supplement: Supplementary file 4 — Source Data for Figure 2 [file EMMM-14-e14797-s002.zip › Figure2d_GFPcontrol_DAPI.tif]

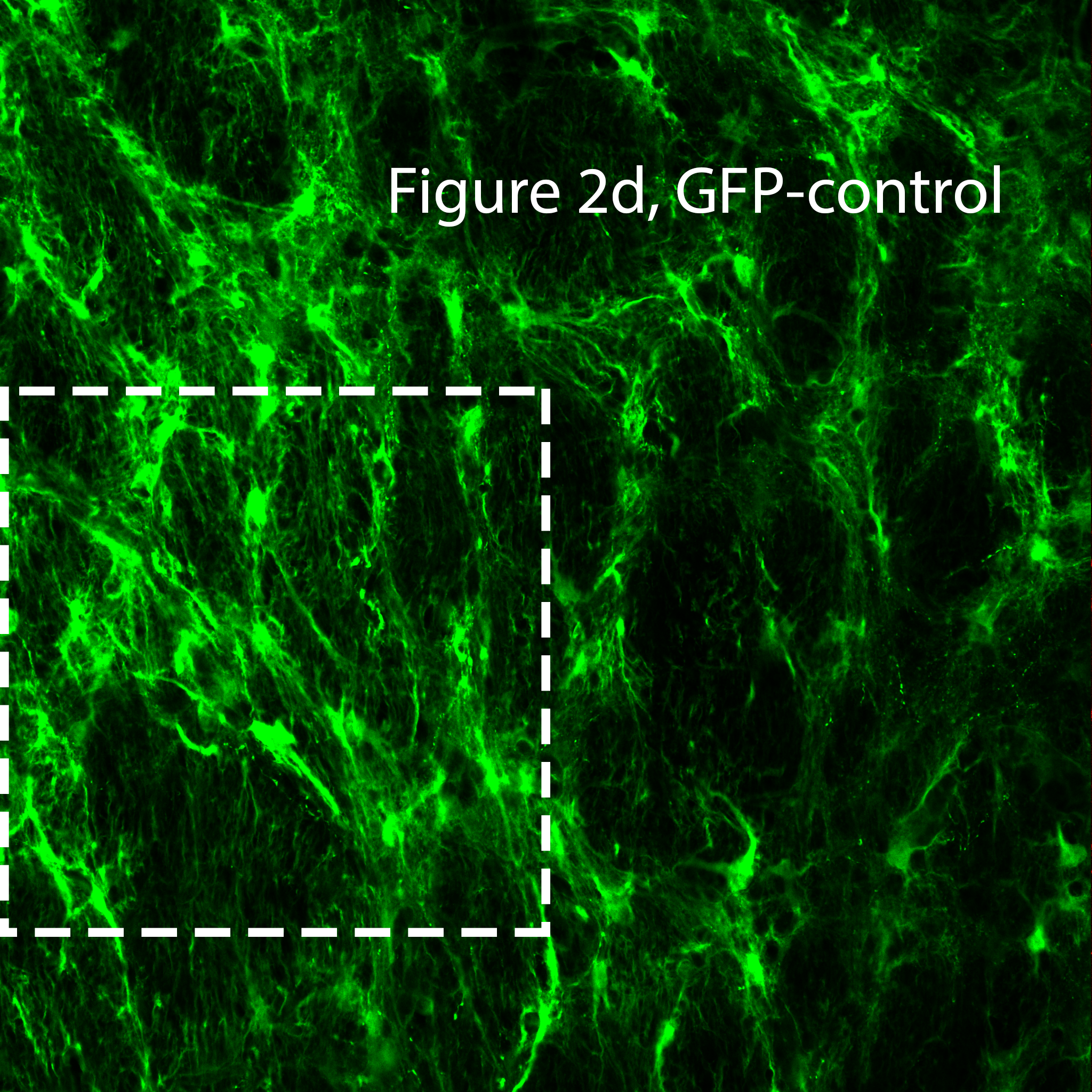

Supplement: Supplementary file 4 — Source Data for Figure 2 [file EMMM-14-e14797-s002.zip › Figure2d_GFPcontrol_GFP.tif]

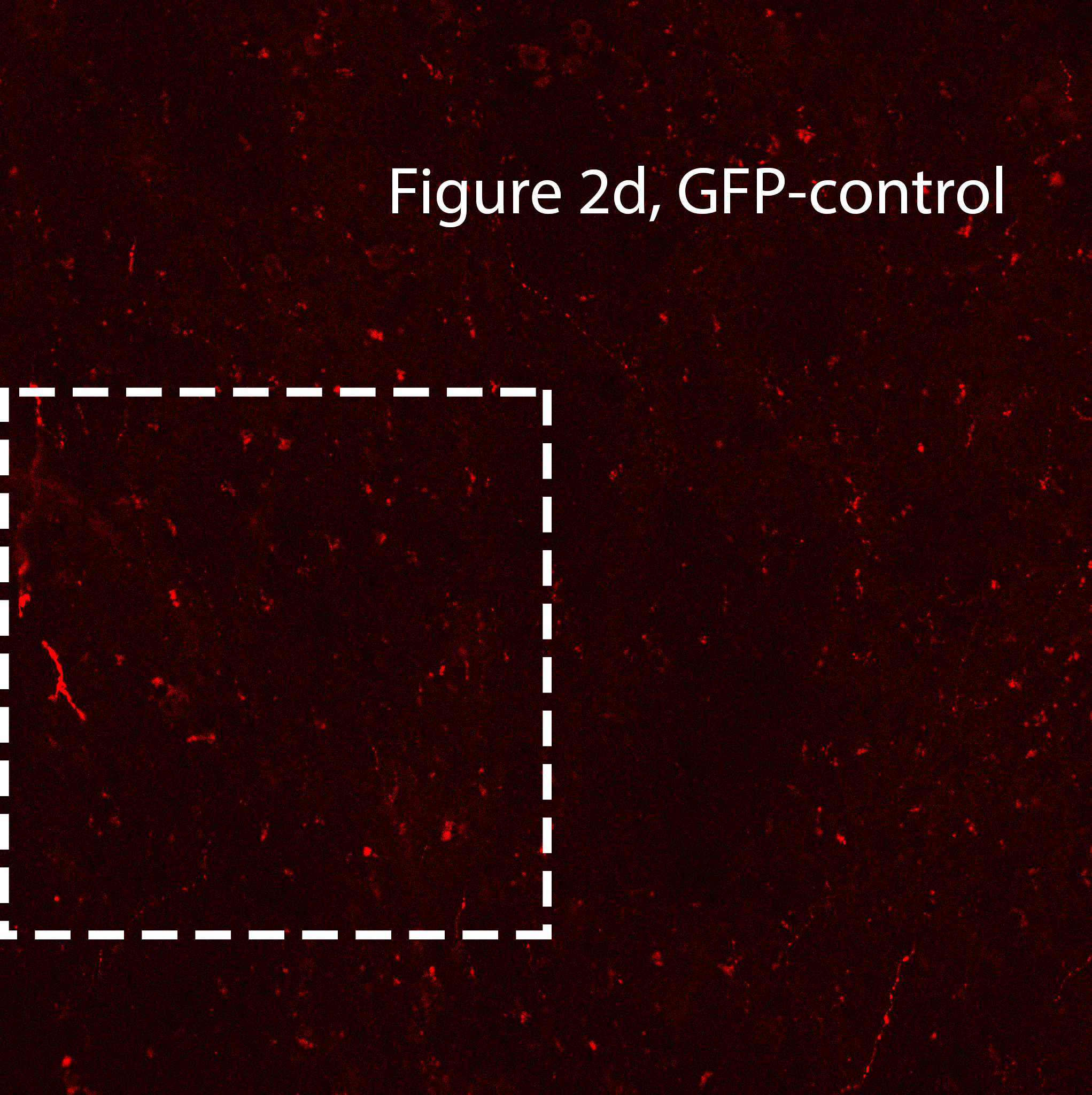

Supplement: Supplementary file 4 — Source Data for Figure 2 [file EMMM-14-e14797-s002.zip › Figure2d_GFPcontrol_TH.tif]

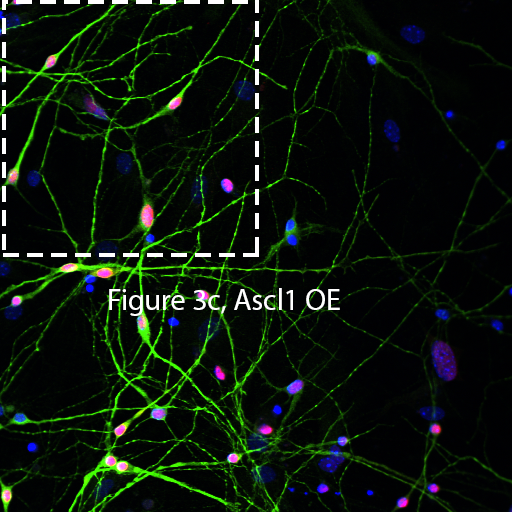

Supplement: Supplementary file 5 — Source Data for Figure 3 [file EMMM-14-e14797-s004.zip › Figure_3c_Ascl1OE_composition.tif]

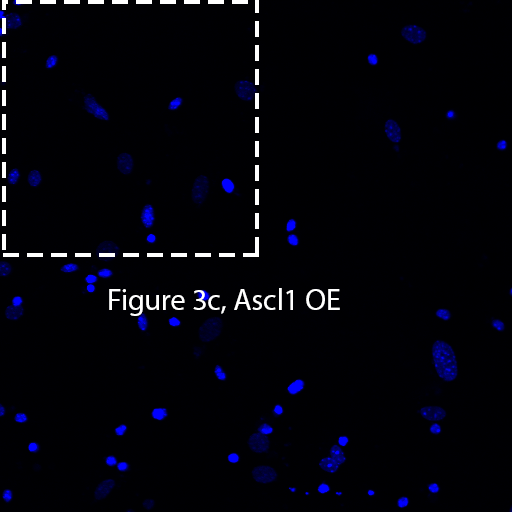

Supplement: Supplementary file 5 — Source Data for Figure 3 [file EMMM-14-e14797-s004.zip › Figure_3c_Ascl1OE_DAPI.tif]

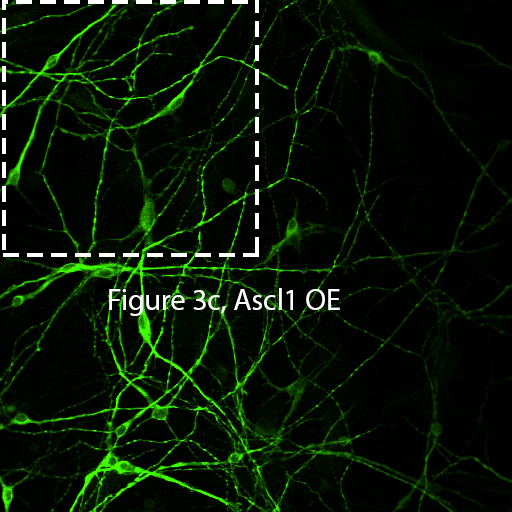

Supplement: Supplementary file 5 — Source Data for Figure 3 [file EMMM-14-e14797-s004.zip › Figure_3c_Ascl1OE_MAP2.tif]

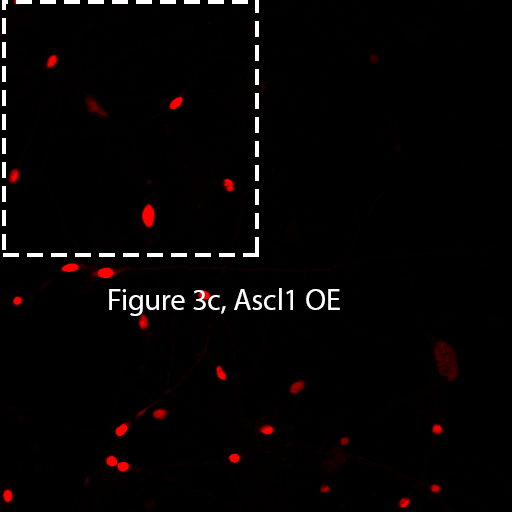

Supplement: Supplementary file 5 — Source Data for Figure 3 [file EMMM-14-e14797-s004.zip › Figure_3c_Ascl1OE_NCas9.tif]

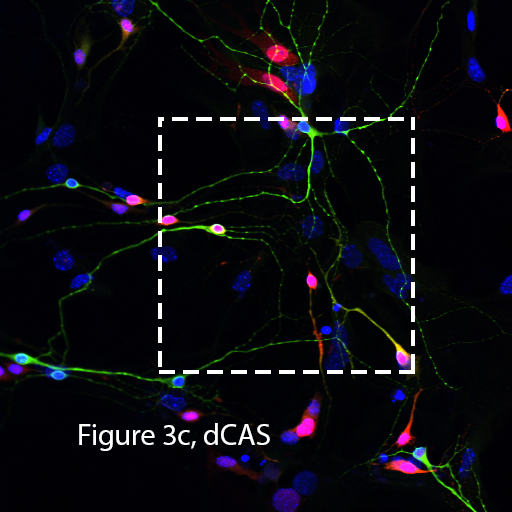

Supplement: Supplementary file 5 — Source Data for Figure 3 [file EMMM-14-e14797-s004.zip › Figure_3c_dCAS_composition.tif]

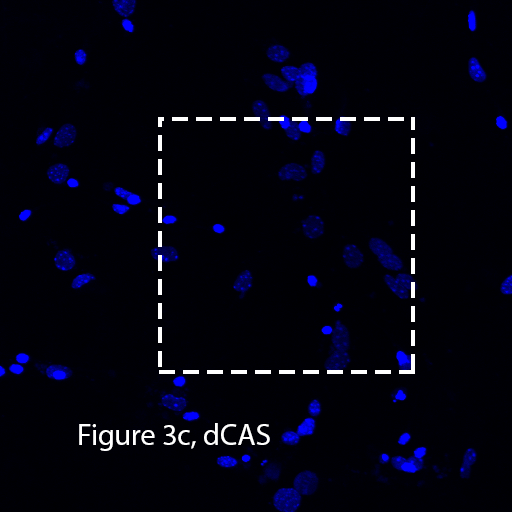

Supplement: Supplementary file 5 — Source Data for Figure 3 [file EMMM-14-e14797-s004.zip › Figure_3c_dCAS_DAPI.tif]

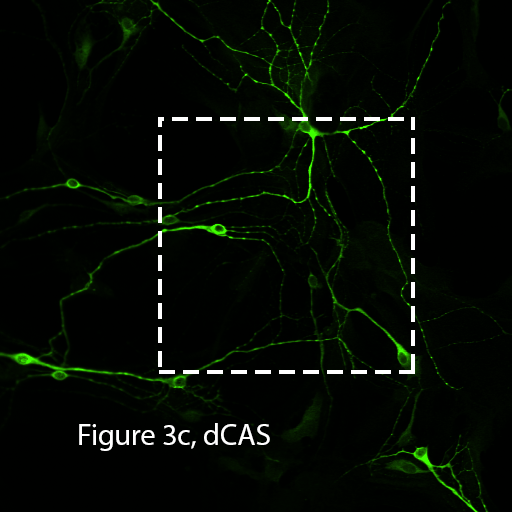

Supplement: Supplementary file 5 — Source Data for Figure 3 [file EMMM-14-e14797-s004.zip › Figure_3c_dCAS_MAP2.tif]

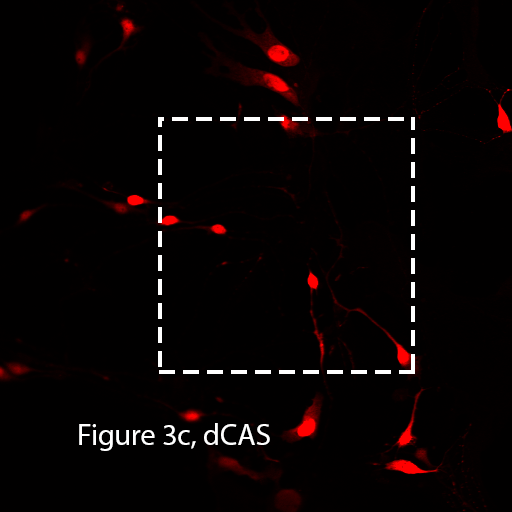

Supplement: Supplementary file 5 — Source Data for Figure 3 [file EMMM-14-e14797-s004.zip › Figure_3c_dCAS_NCas9.tif]

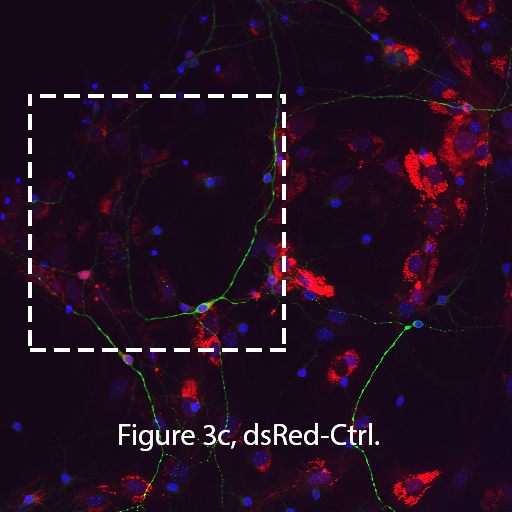

Supplement: Supplementary file 5 — Source Data for Figure 3 [file EMMM-14-e14797-s004.zip › Figure_3c_dsRed-Ctrl_composition.tif]

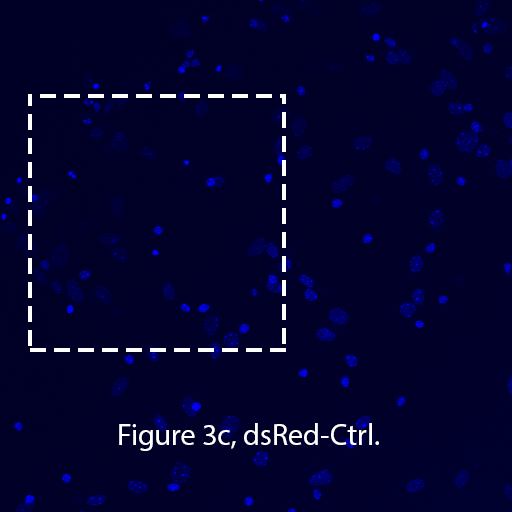

Supplement: Supplementary file 5 — Source Data for Figure 3 [file EMMM-14-e14797-s004.zip › Figure_3c_dsRed-Ctrl_DAPI.tif]

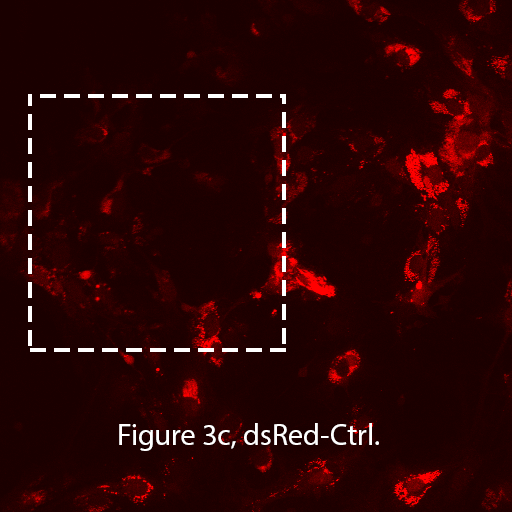

Supplement: Supplementary file 5 — Source Data for Figure 3 [file EMMM-14-e14797-s004.zip › Figure_3c_dsRed-Ctrl_dsRed.tif]

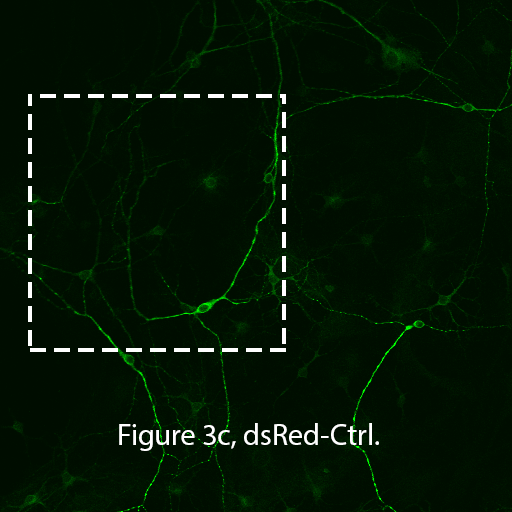

Supplement: Supplementary file 5 — Source Data for Figure 3 [file EMMM-14-e14797-s004.zip › Figure_3c_dsRed-Ctrl_MAP2.tif]

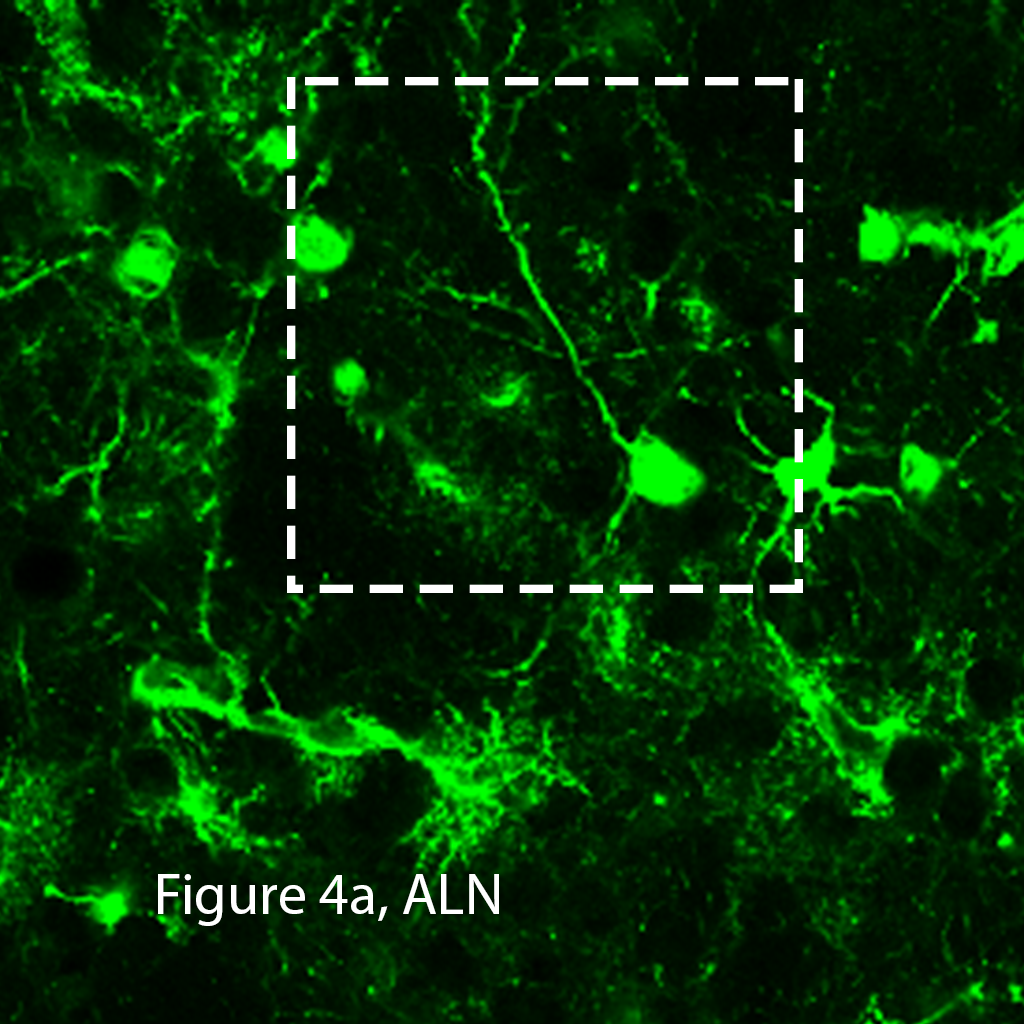

Supplement: Supplementary file 6 — Source Data for Figure 4 [file EMMM-14-e14797-s008.zip › Figure4a_ALN.tif]

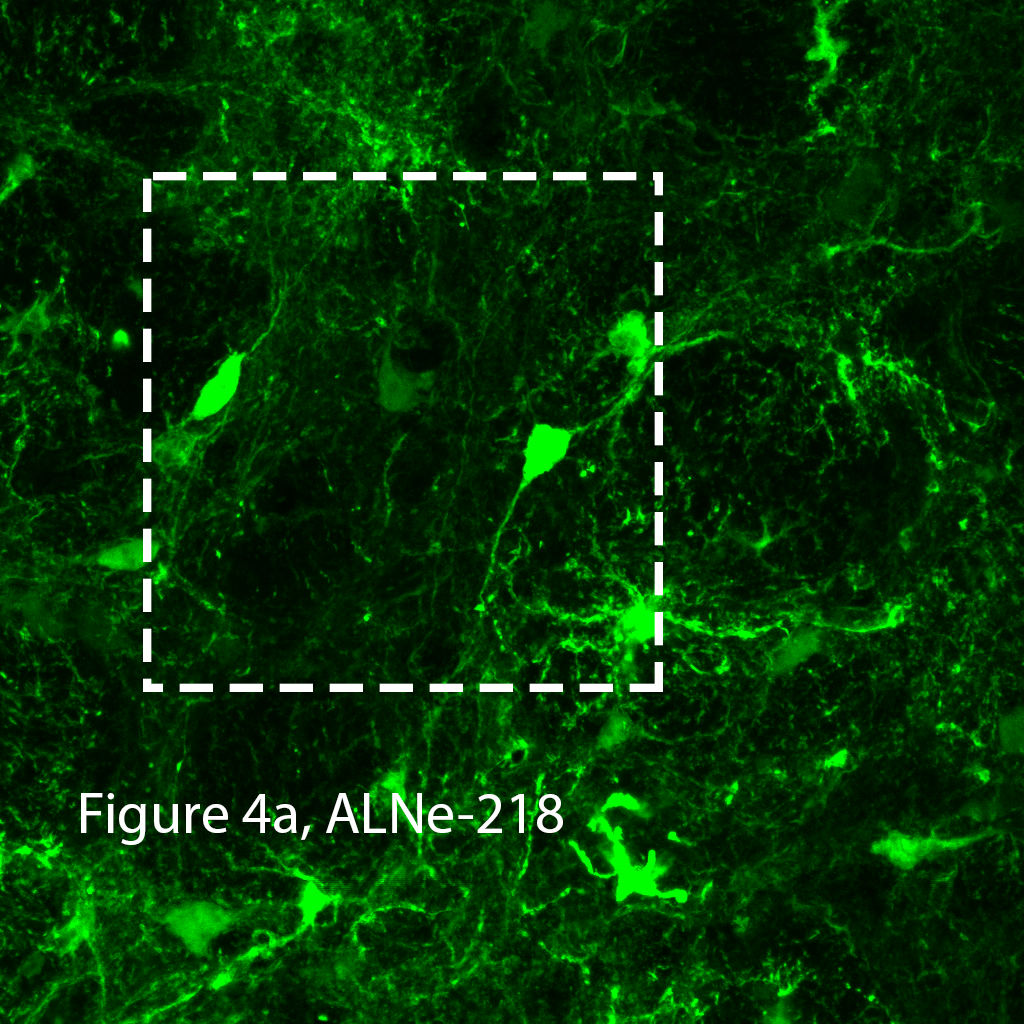

Supplement: Supplementary file 6 — Source Data for Figure 4 [file EMMM-14-e14797-s008.zip › Figure4a_ALNe-218.tif]

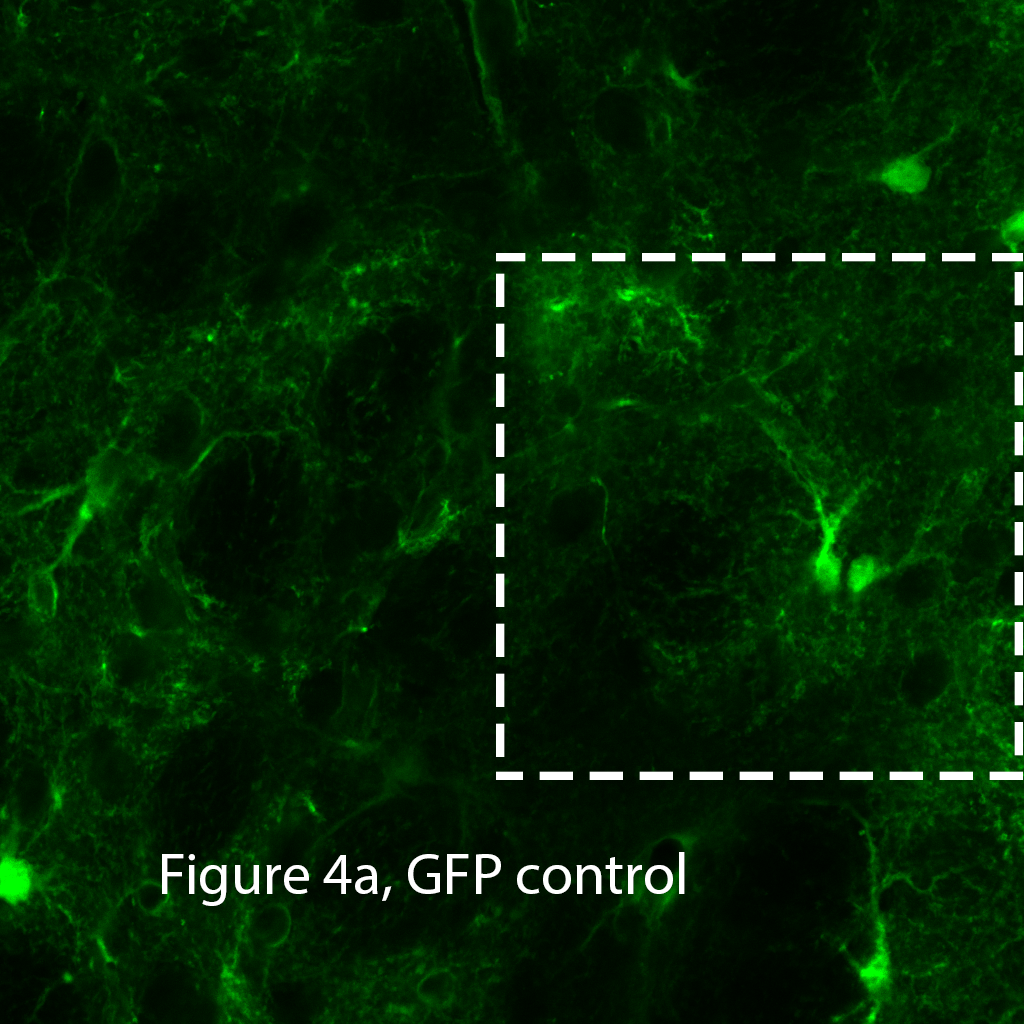

Supplement: Supplementary file 6 — Source Data for Figure 4 [file EMMM-14-e14797-s008.zip › Figure4a_GFPcontrol.tif]

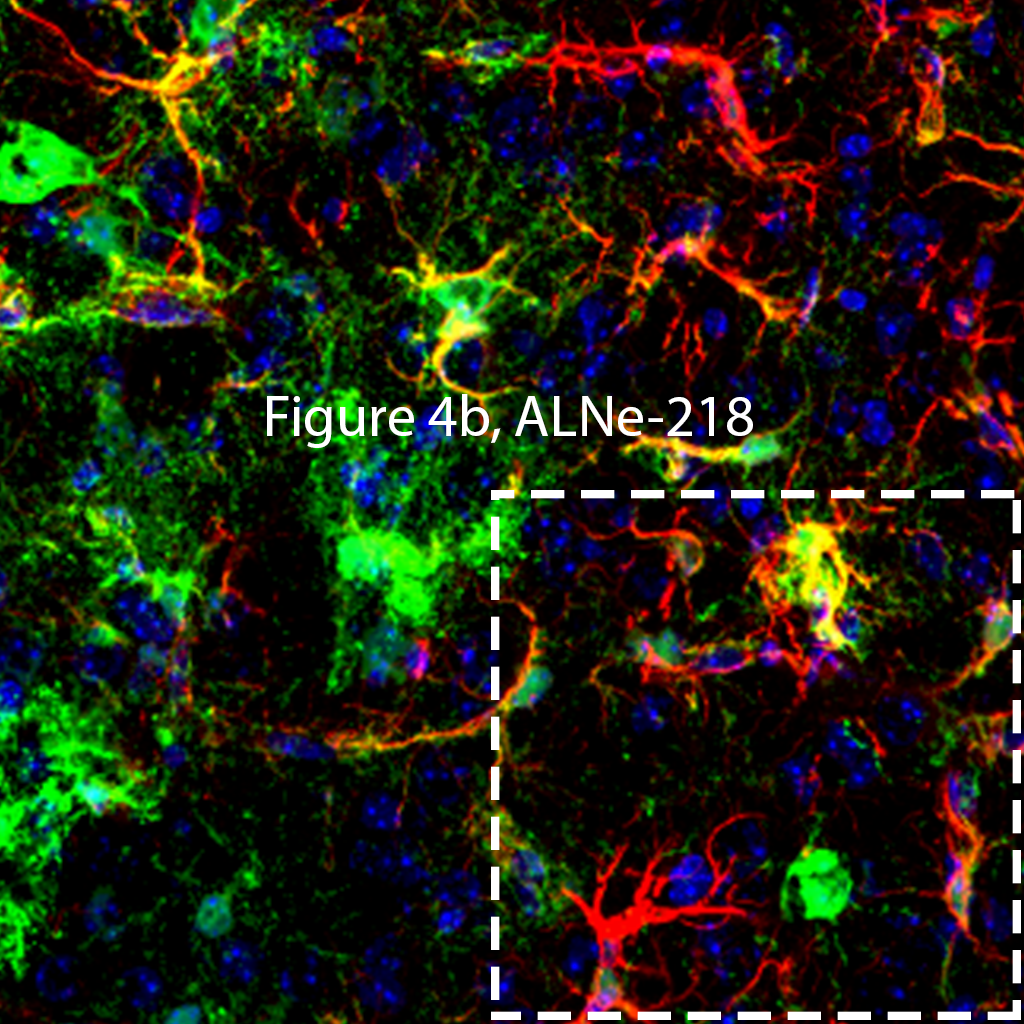

Supplement: Supplementary file 6 — Source Data for Figure 4 [file EMMM-14-e14797-s008.zip › Figure4b_ALNe-218_composite.tif]

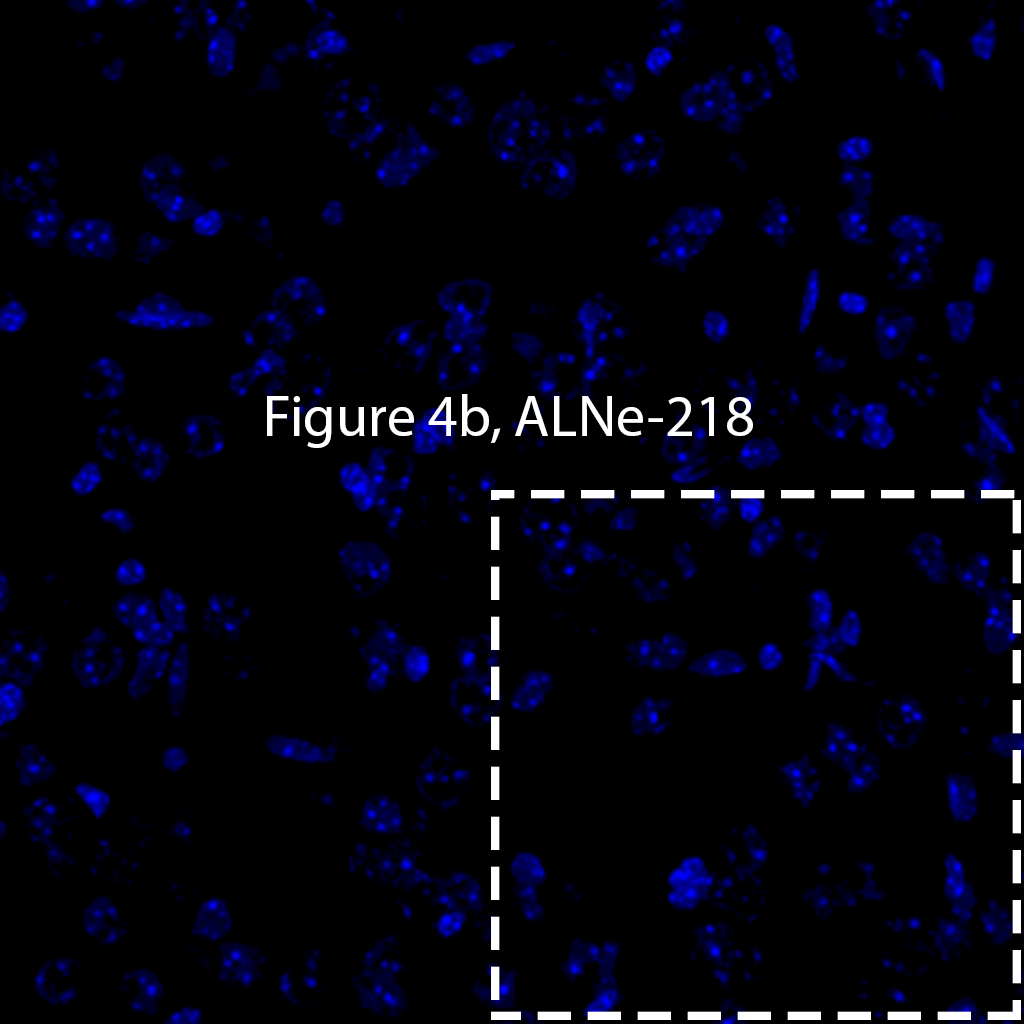

Supplement: Supplementary file 6 — Source Data for Figure 4 [file EMMM-14-e14797-s008.zip › Figure4b_ALNe-218_DAPI.tif]

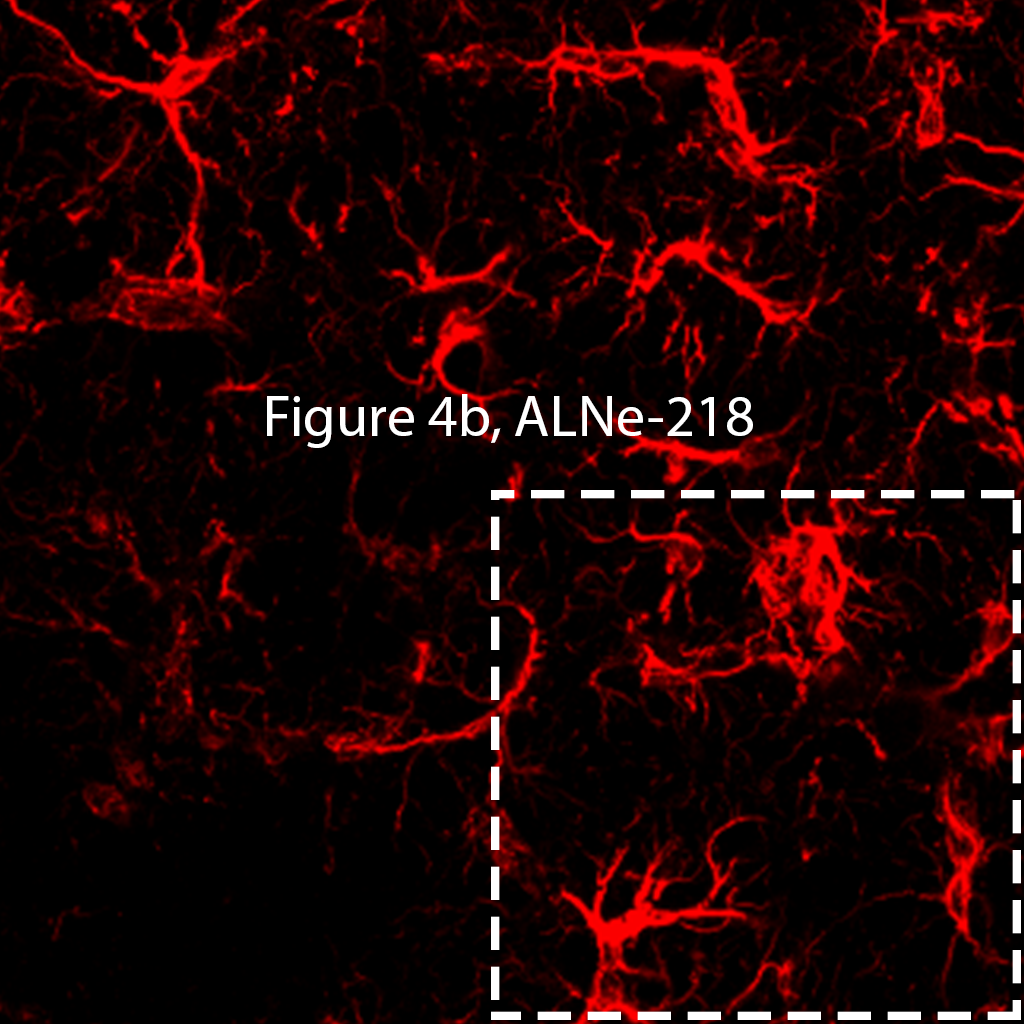

Supplement: Supplementary file 6 — Source Data for Figure 4 [file EMMM-14-e14797-s008.zip › Figure4b_ALNe-218_GFAP.tif]

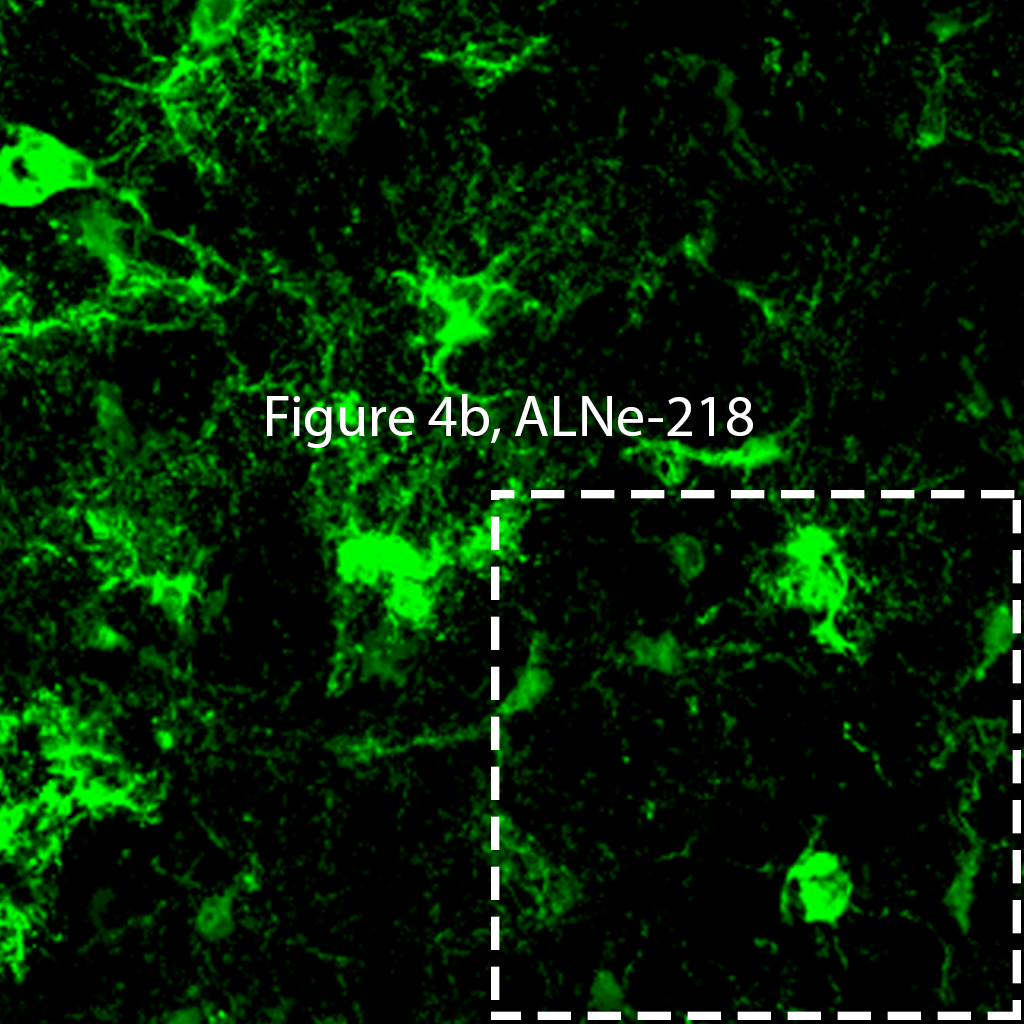

Supplement: Supplementary file 6 — Source Data for Figure 4 [file EMMM-14-e14797-s008.zip › Figure4b_ALNe-218_GFP.tif]

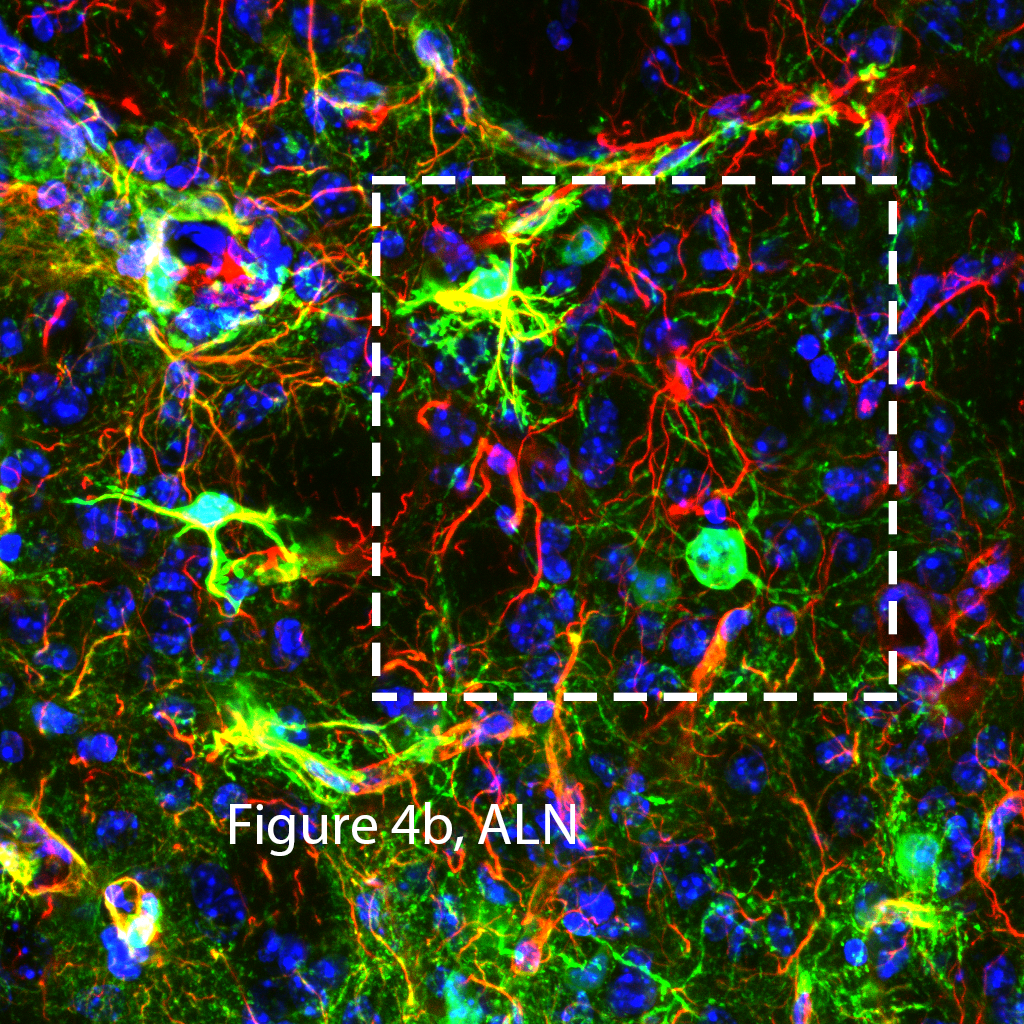

Supplement: Supplementary file 6 — Source Data for Figure 4 [file EMMM-14-e14797-s008.zip › Figure4b_ALN_composite.tif]

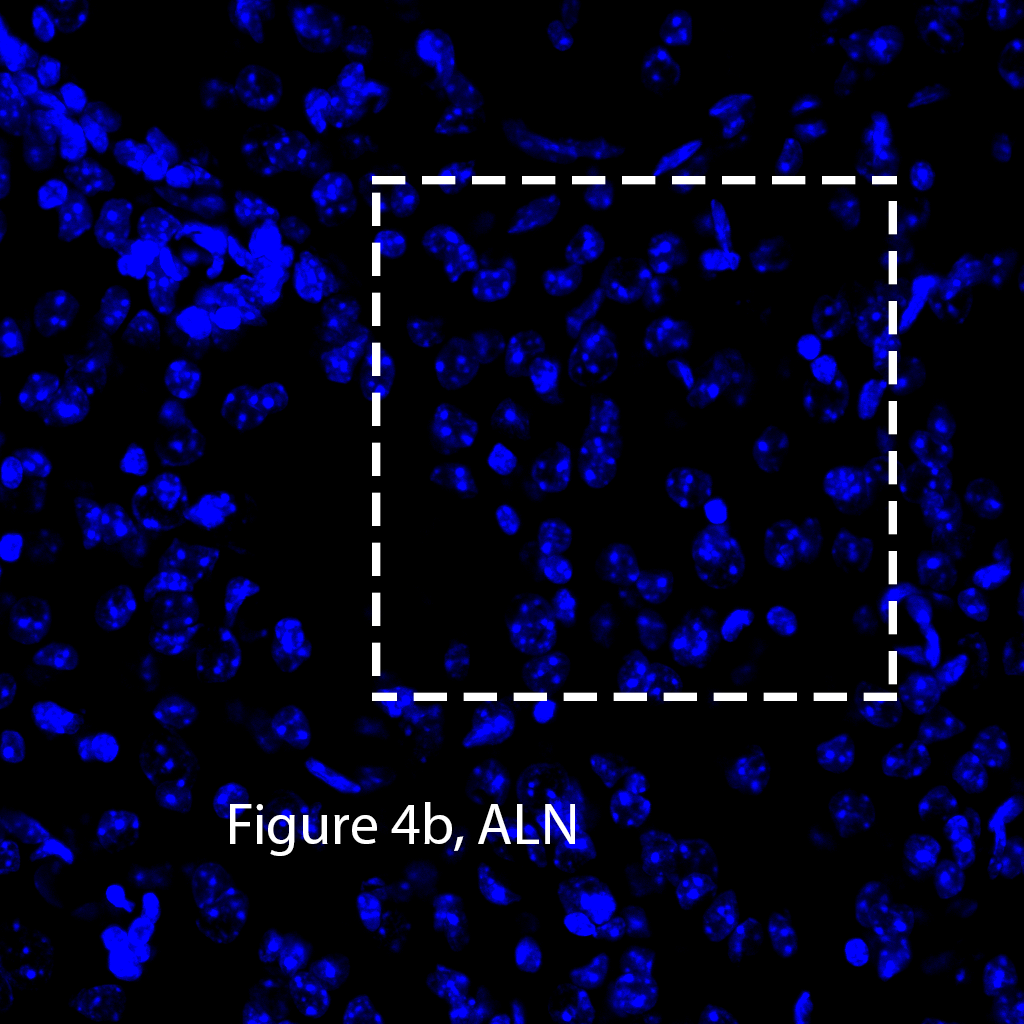

Supplement: Supplementary file 6 — Source Data for Figure 4 [file EMMM-14-e14797-s008.zip › Figure4b_ALN_DAPI.tif]

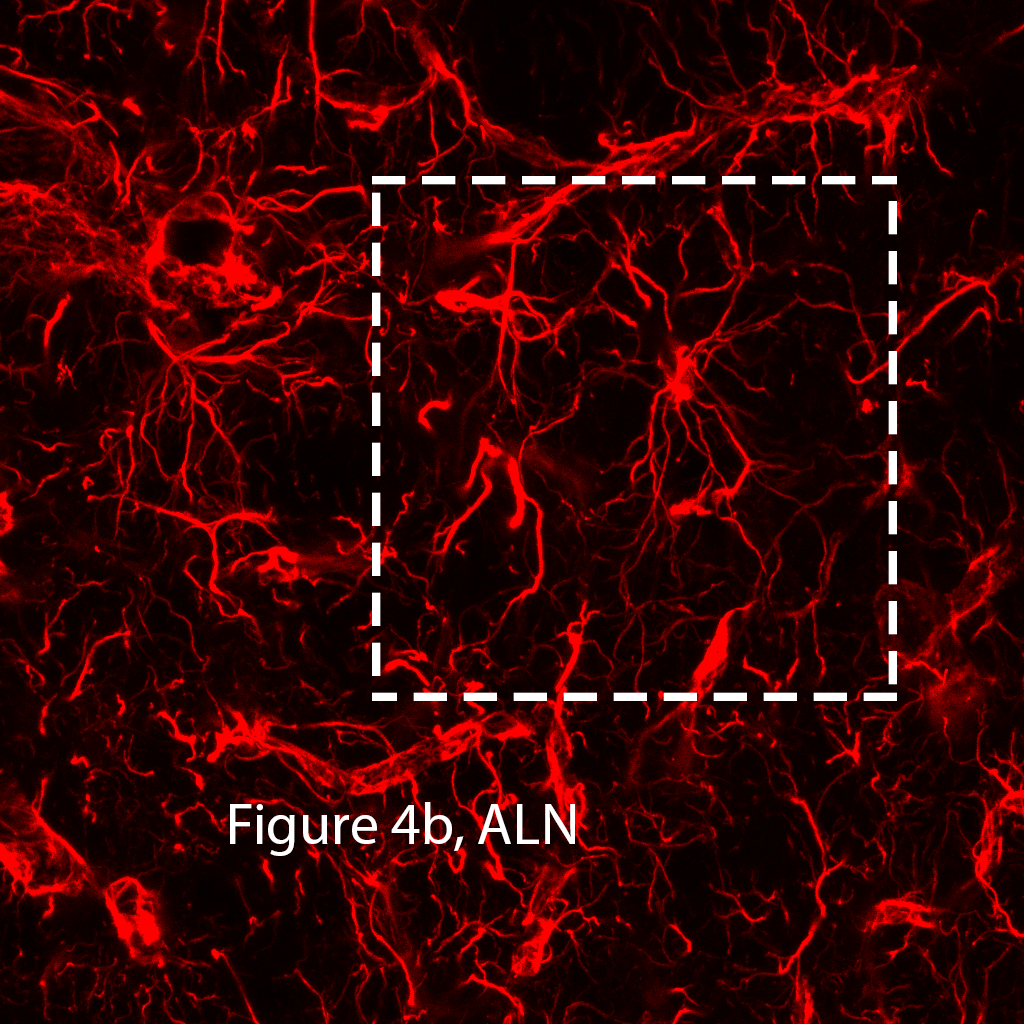

Supplement: Supplementary file 6 — Source Data for Figure 4 [file EMMM-14-e14797-s008.zip › Figure4b_ALN_GFAP.tif]

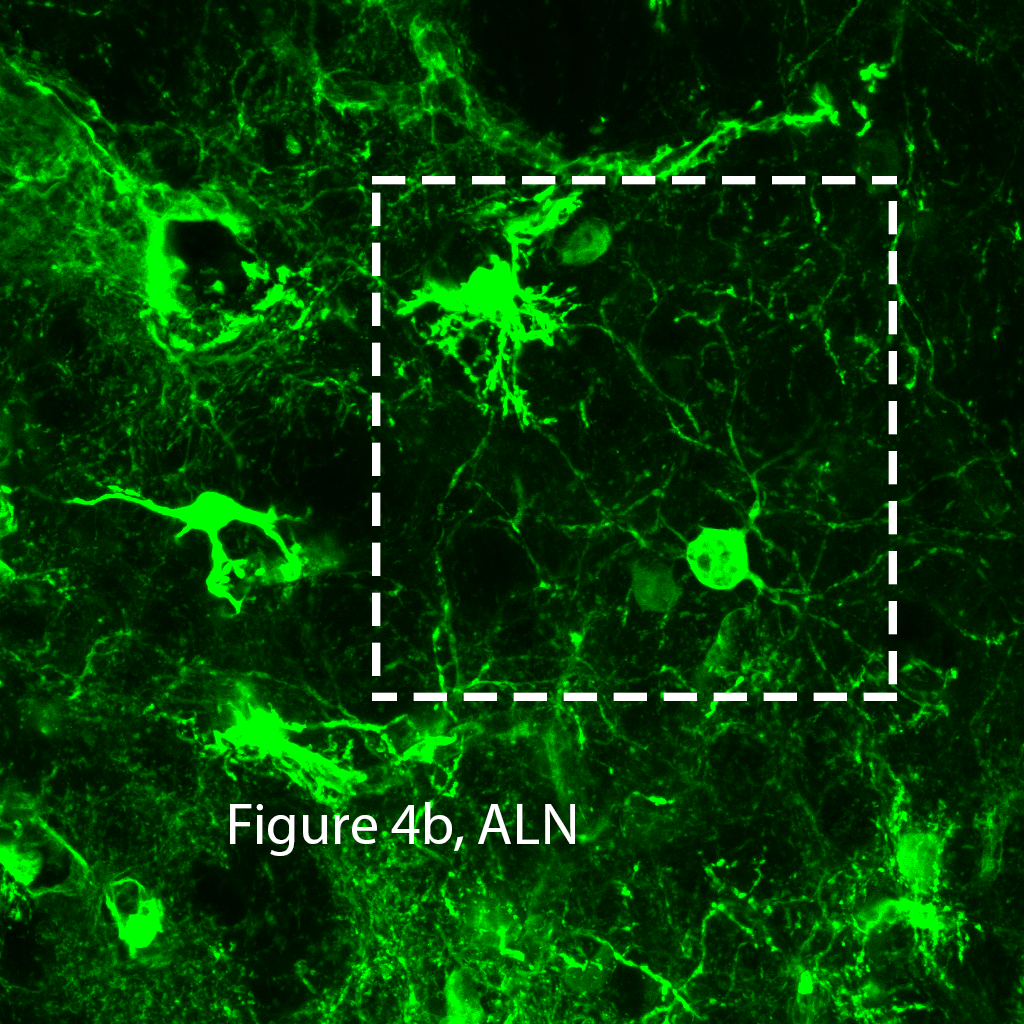

Supplement: Supplementary file 6 — Source Data for Figure 4 [file EMMM-14-e14797-s008.zip › Figure4b_ALN_GFP.tif]

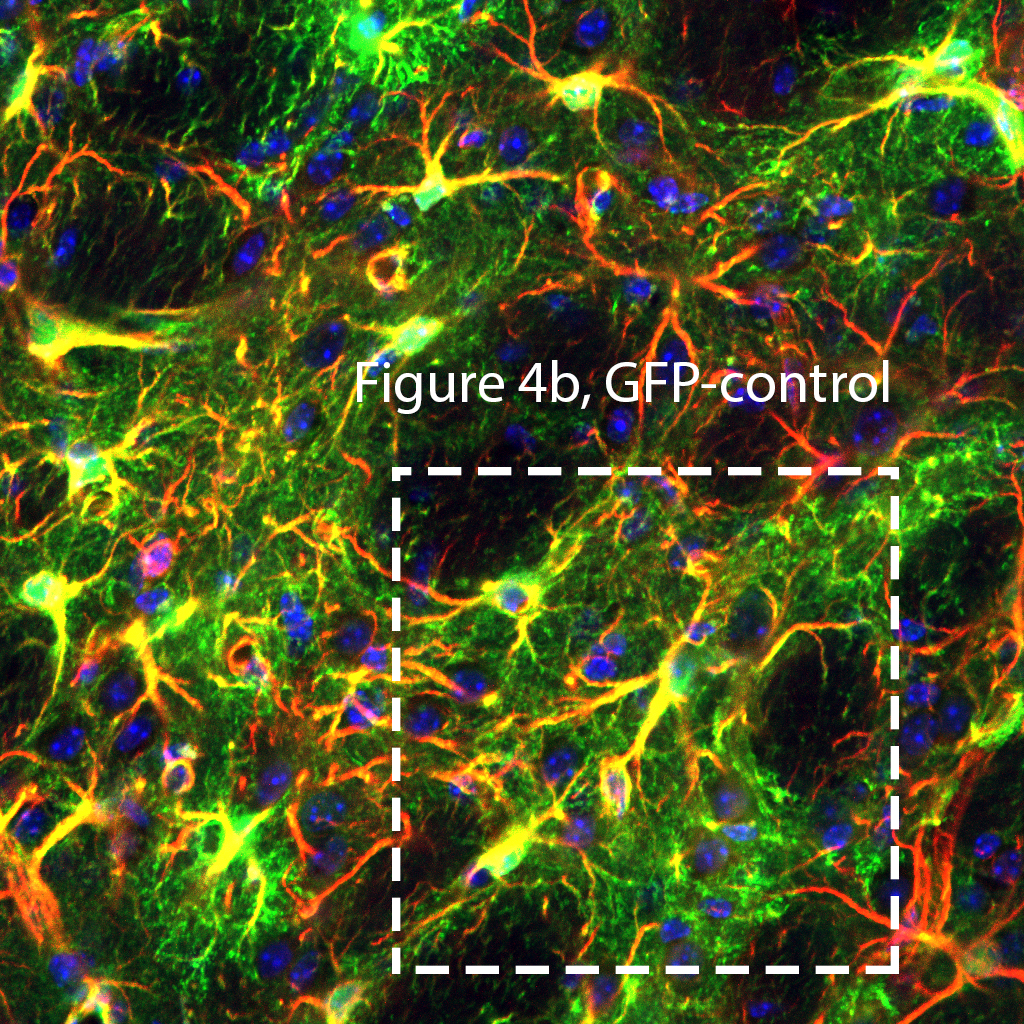

Supplement: Supplementary file 6 — Source Data for Figure 4 [file EMMM-14-e14797-s008.zip › Figure4b_GFPcontrol_composite.tif]

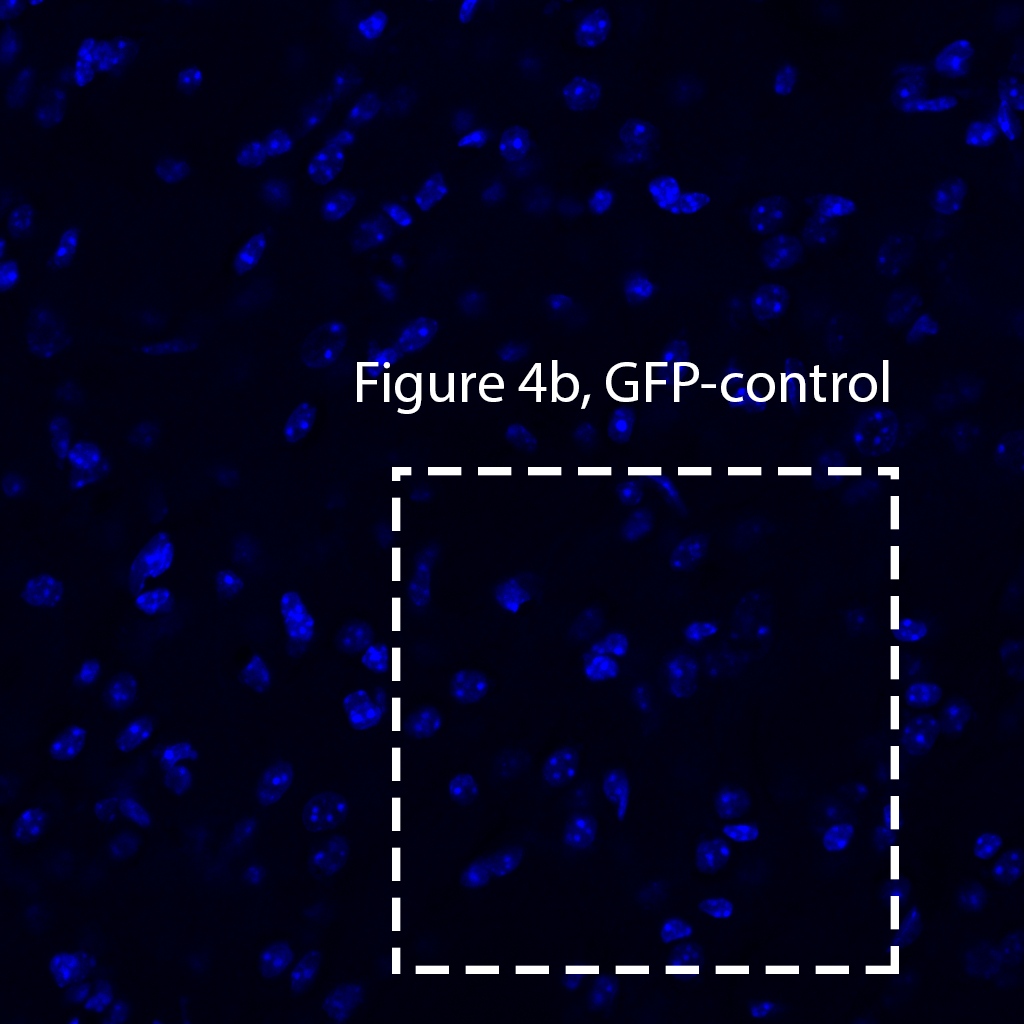

Supplement: Supplementary file 6 — Source Data for Figure 4 [file EMMM-14-e14797-s008.zip › Figure4b_GFPcontrol_DAPI.tif]

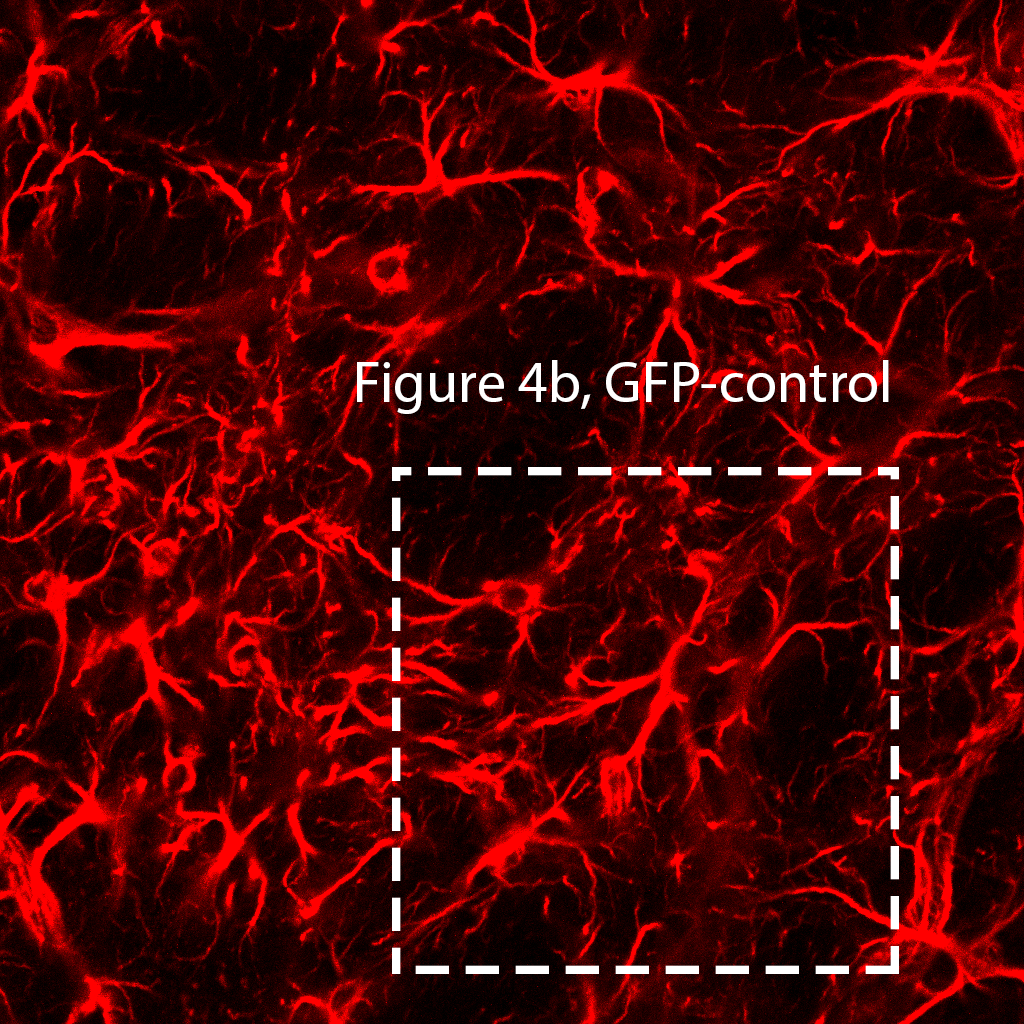

Supplement: Supplementary file 6 — Source Data for Figure 4 [file EMMM-14-e14797-s008.zip › Figure4b_GFPcontrol_GFAP.tif]

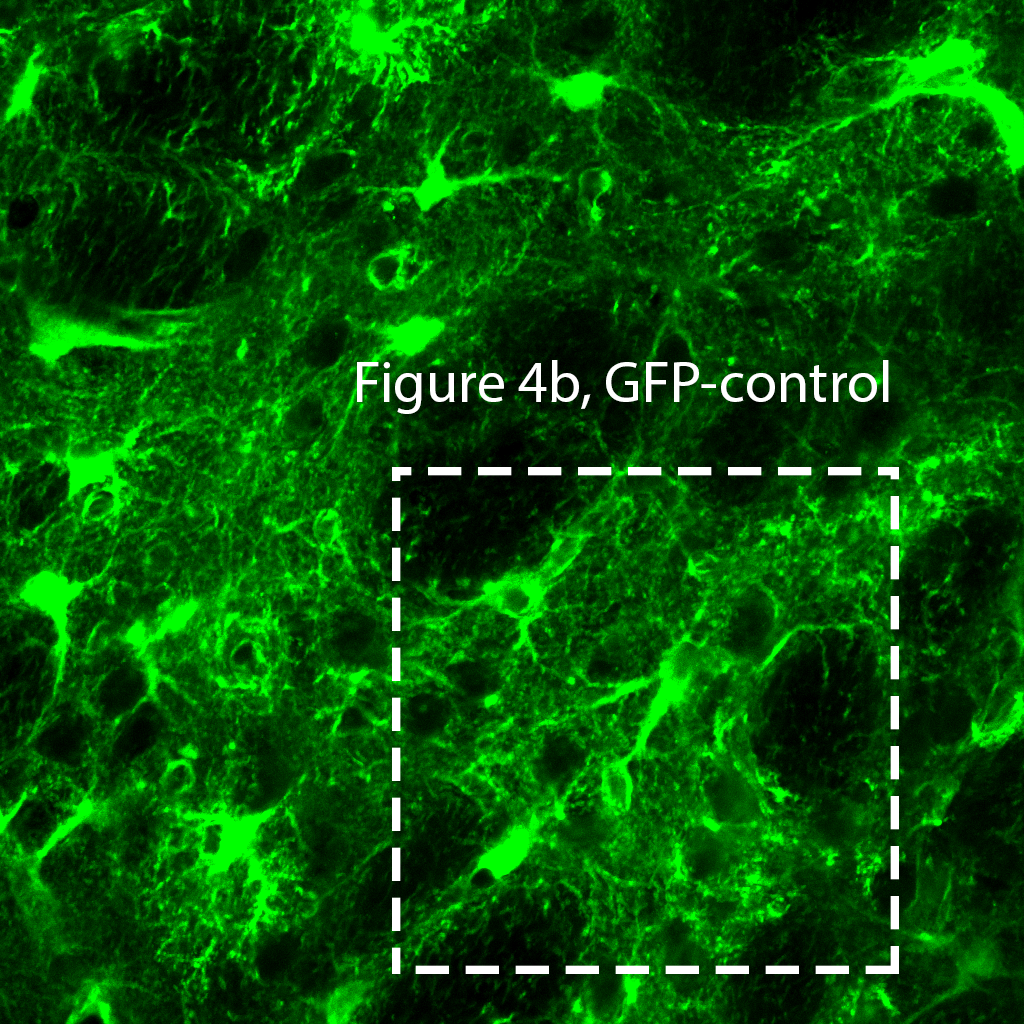

Supplement: Supplementary file 6 — Source Data for Figure 4 [file EMMM-14-e14797-s008.zip › Figure4b_GFPcontrol_GFP.tif]

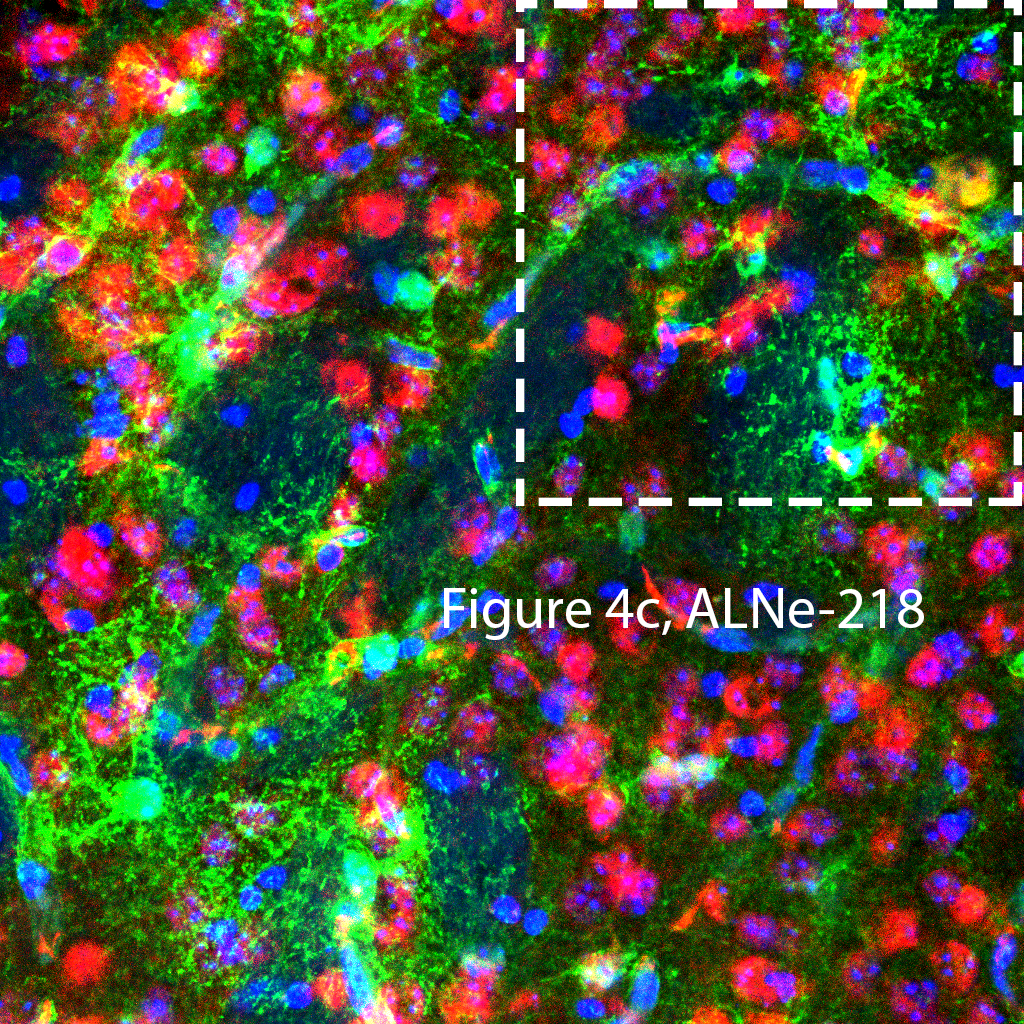

Supplement: Supplementary file 6 — Source Data for Figure 4 [file EMMM-14-e14797-s008.zip › Figure4c_ALNe-218_composite.tif]

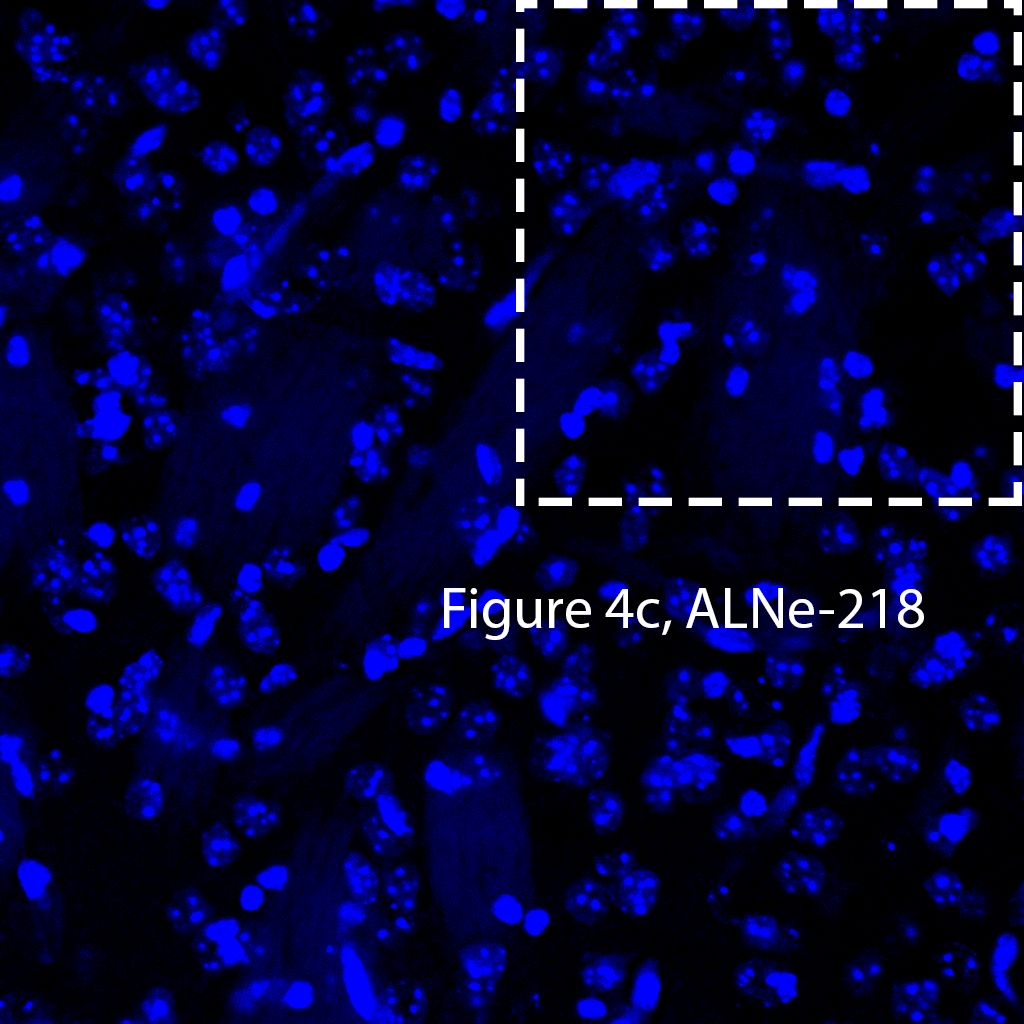

Supplement: Supplementary file 6 — Source Data for Figure 4 [file EMMM-14-e14797-s008.zip › Figure4c_ALNe-218_DAPI.tif]

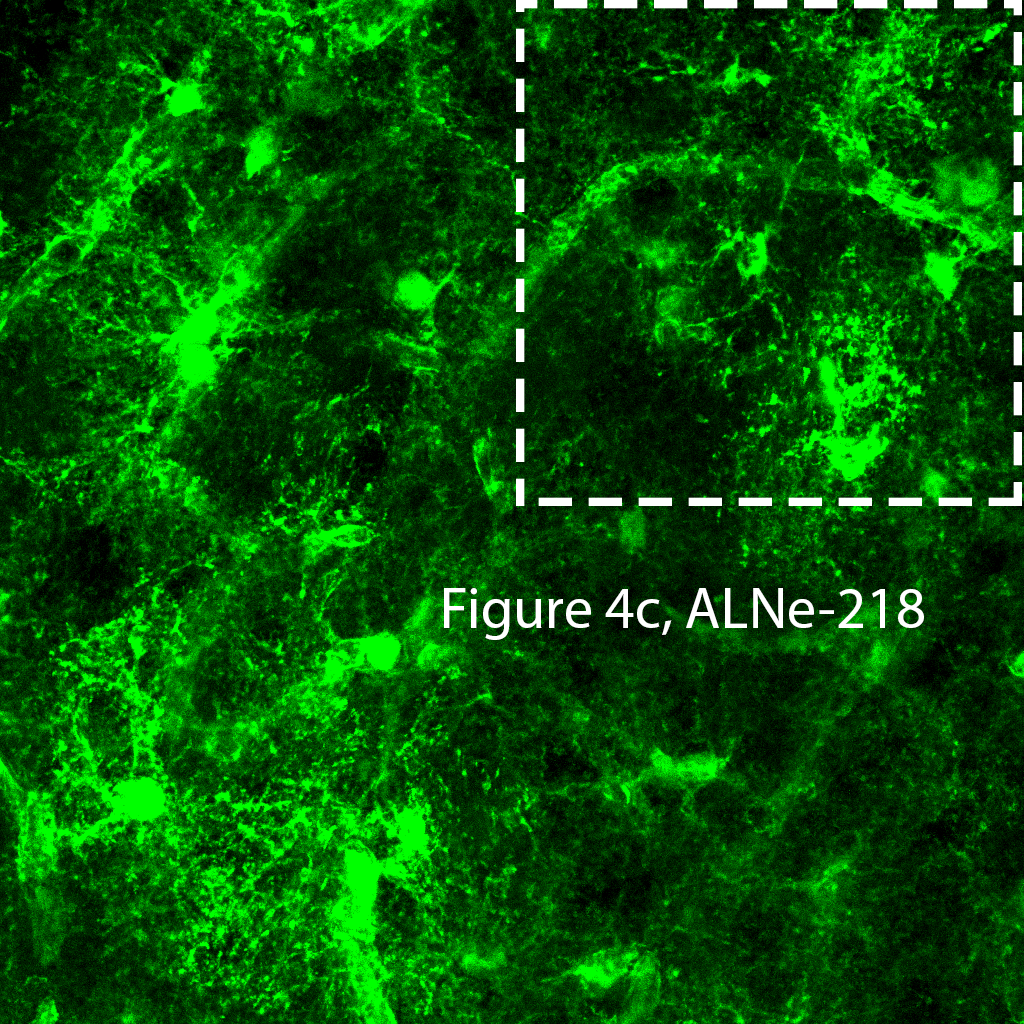

Supplement: Supplementary file 6 — Source Data for Figure 4 [file EMMM-14-e14797-s008.zip › Figure4c_ALNe-218_GFP.tif]

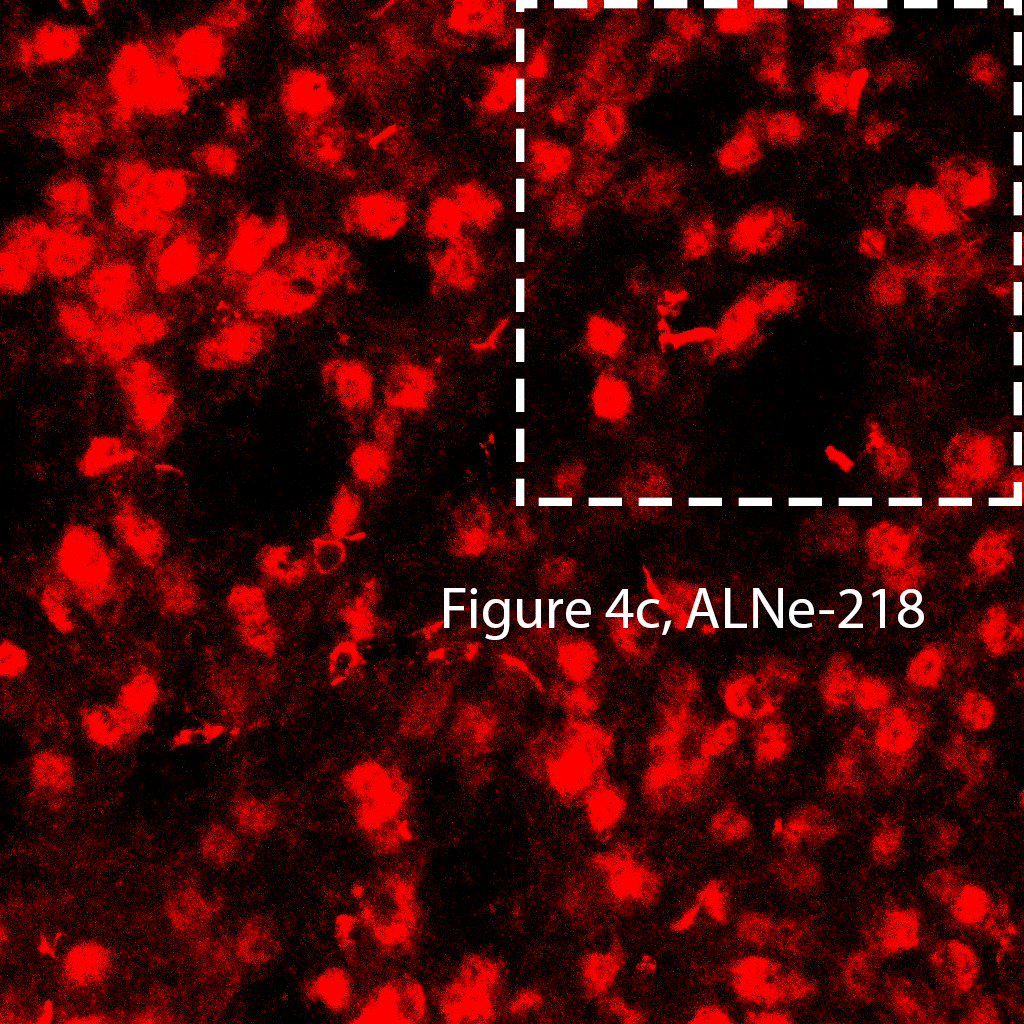

Supplement: Supplementary file 6 — Source Data for Figure 4 [file EMMM-14-e14797-s008.zip › Figure4c_ALNe-218_NeuN.tif]

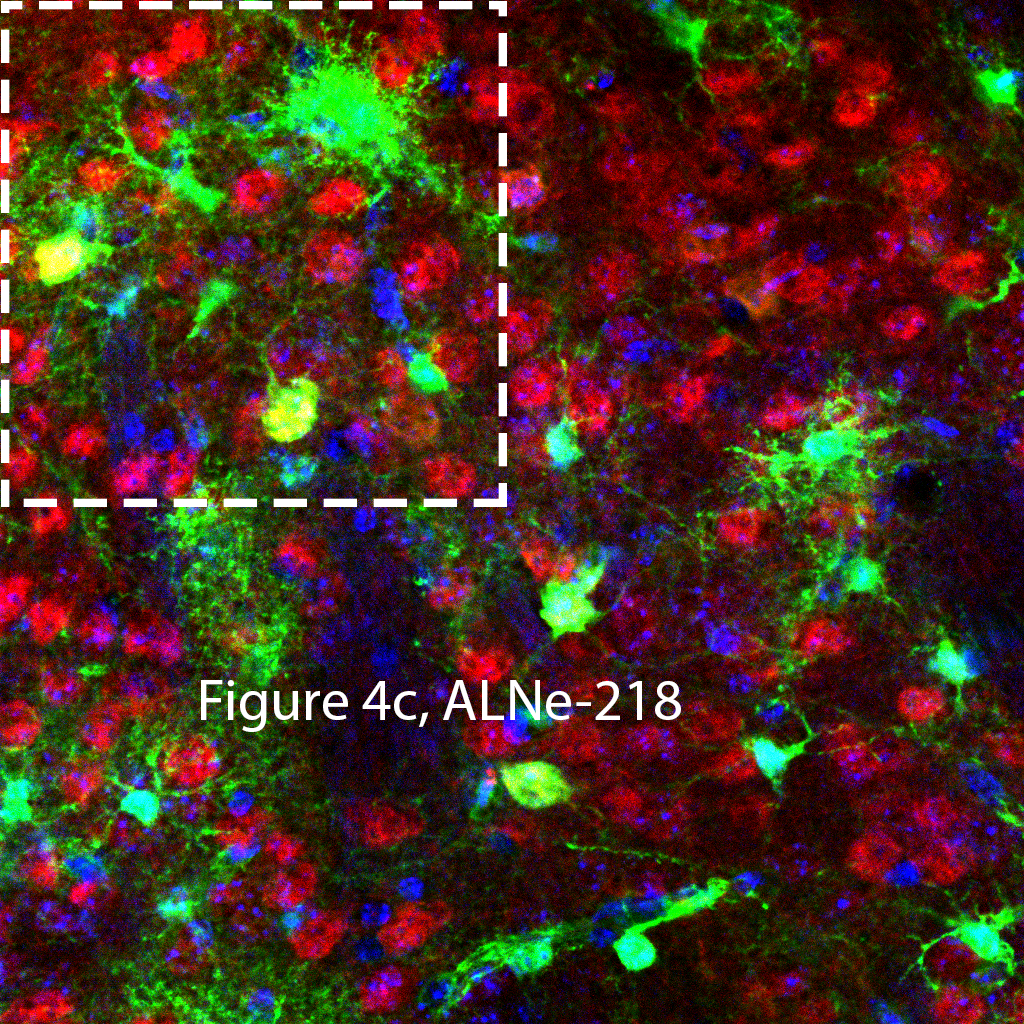

Supplement: Supplementary file 6 — Source Data for Figure 4 [file EMMM-14-e14797-s008.zip › Figure4c_ALN_composite.tif]

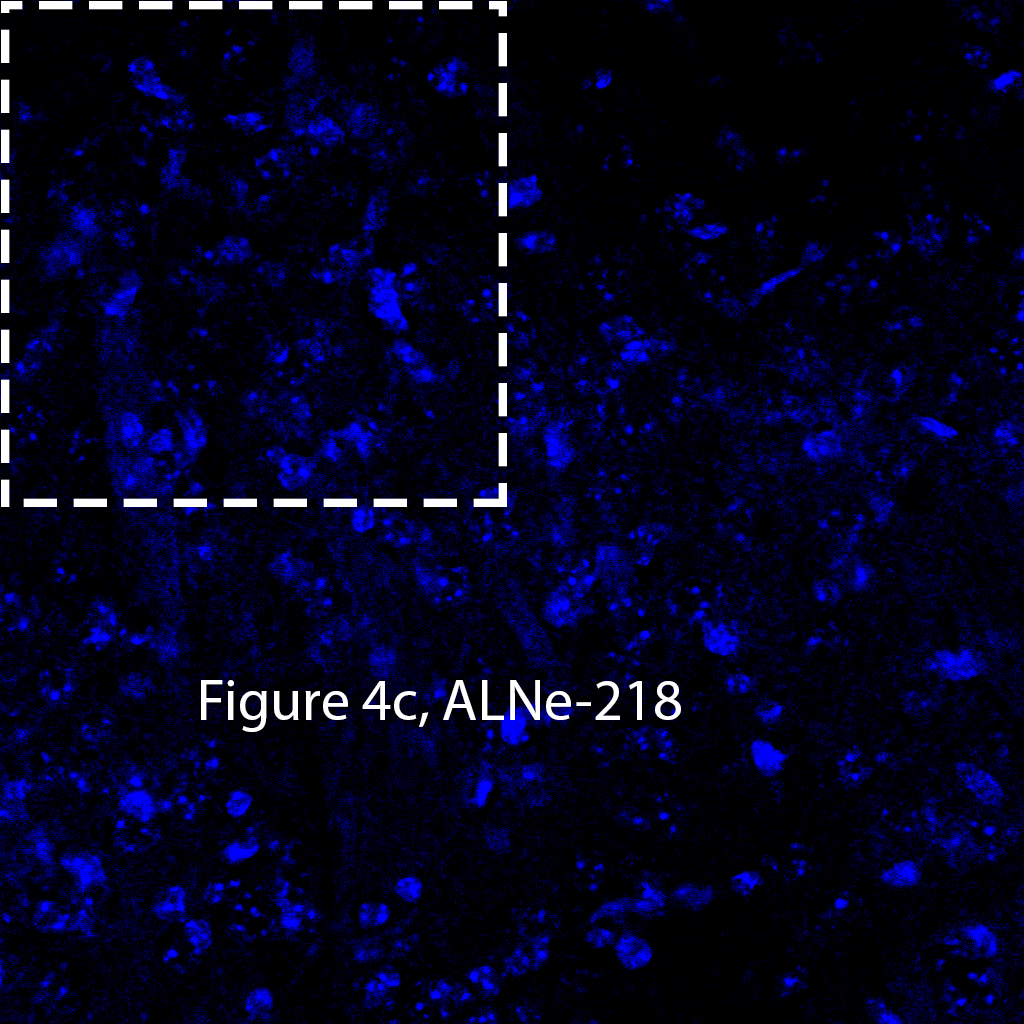

Supplement: Supplementary file 6 — Source Data for Figure 4 [file EMMM-14-e14797-s008.zip › Figure4c_ALN_DAPI.tif]

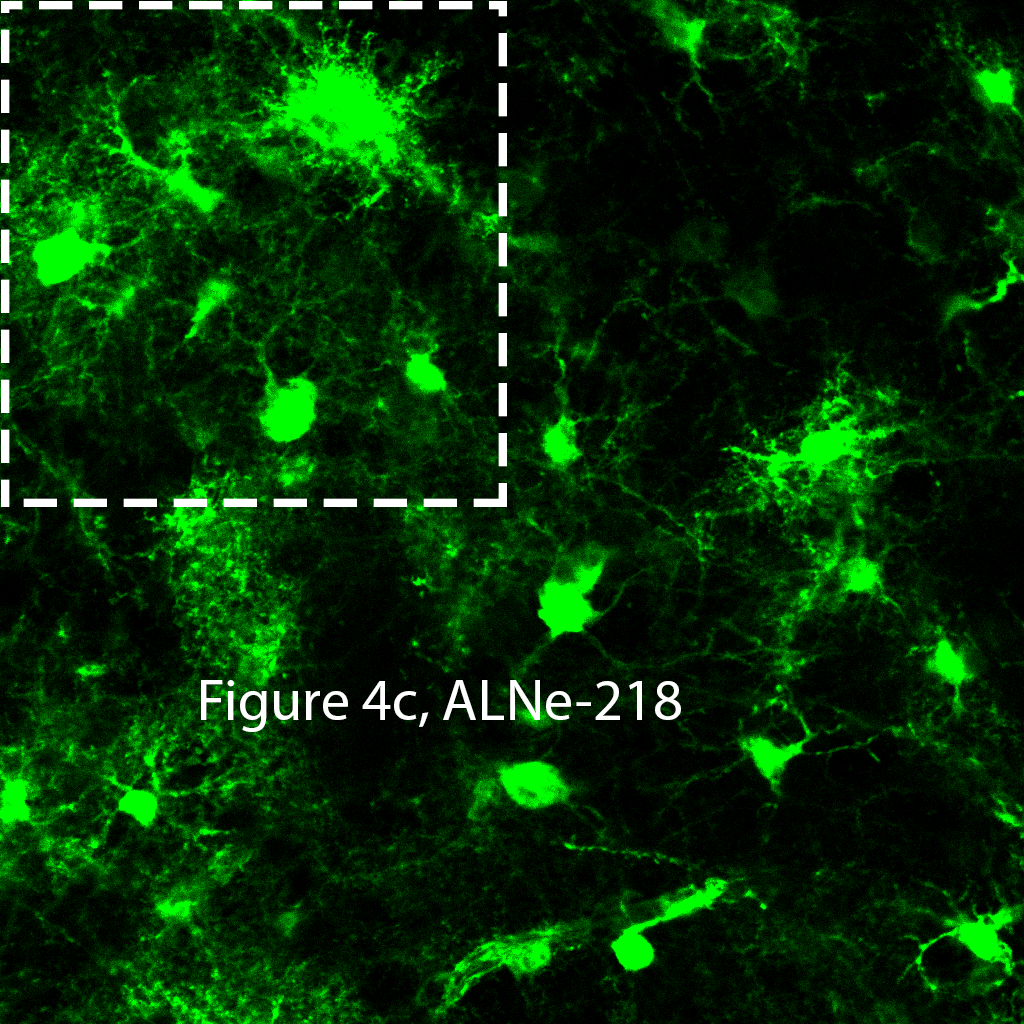

Supplement: Supplementary file 6 — Source Data for Figure 4 [file EMMM-14-e14797-s008.zip › Figure4c_ALN_GFP.tif]

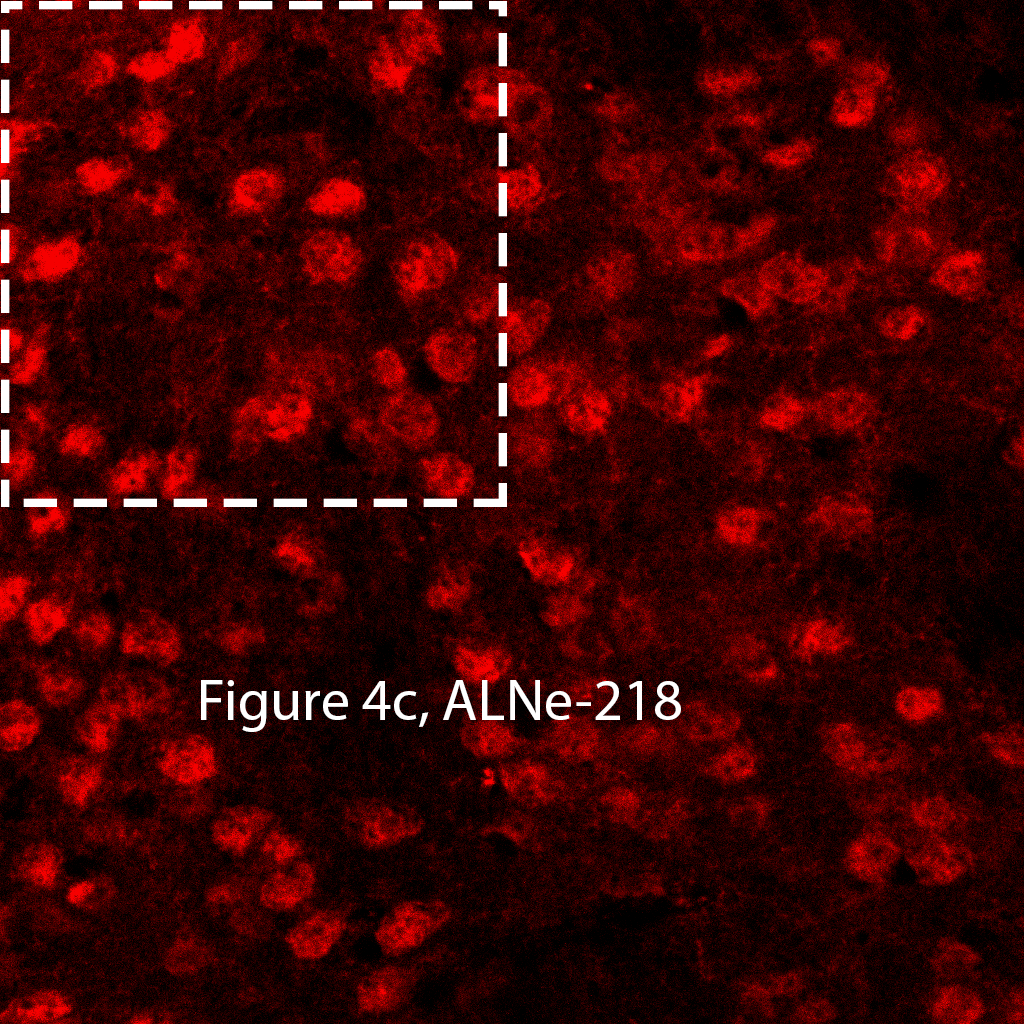

Supplement: Supplementary file 6 — Source Data for Figure 4 [file EMMM-14-e14797-s008.zip › Figure4c_ALN_NeuN.tif]

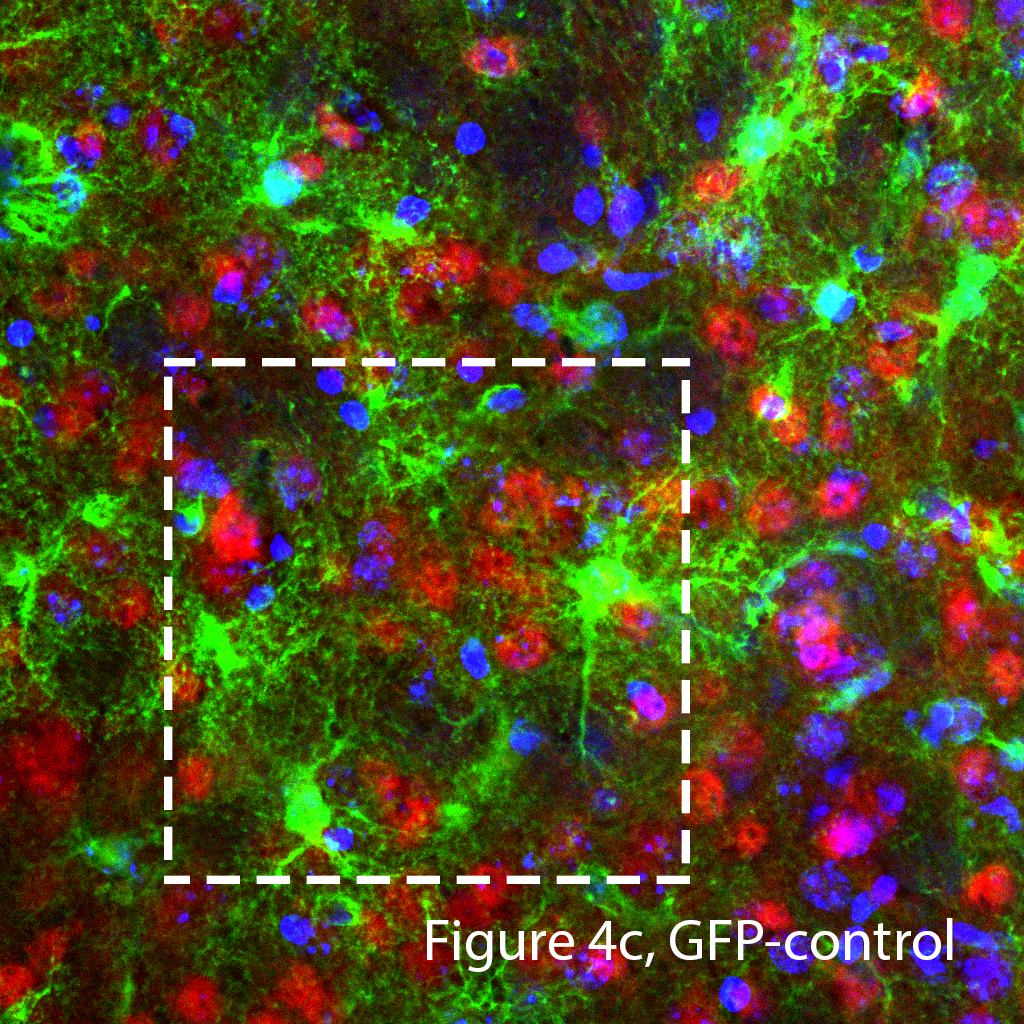

Supplement: Supplementary file 6 — Source Data for Figure 4 [file EMMM-14-e14797-s008.zip › Figure4c_GFPcontrol_composite.tif]

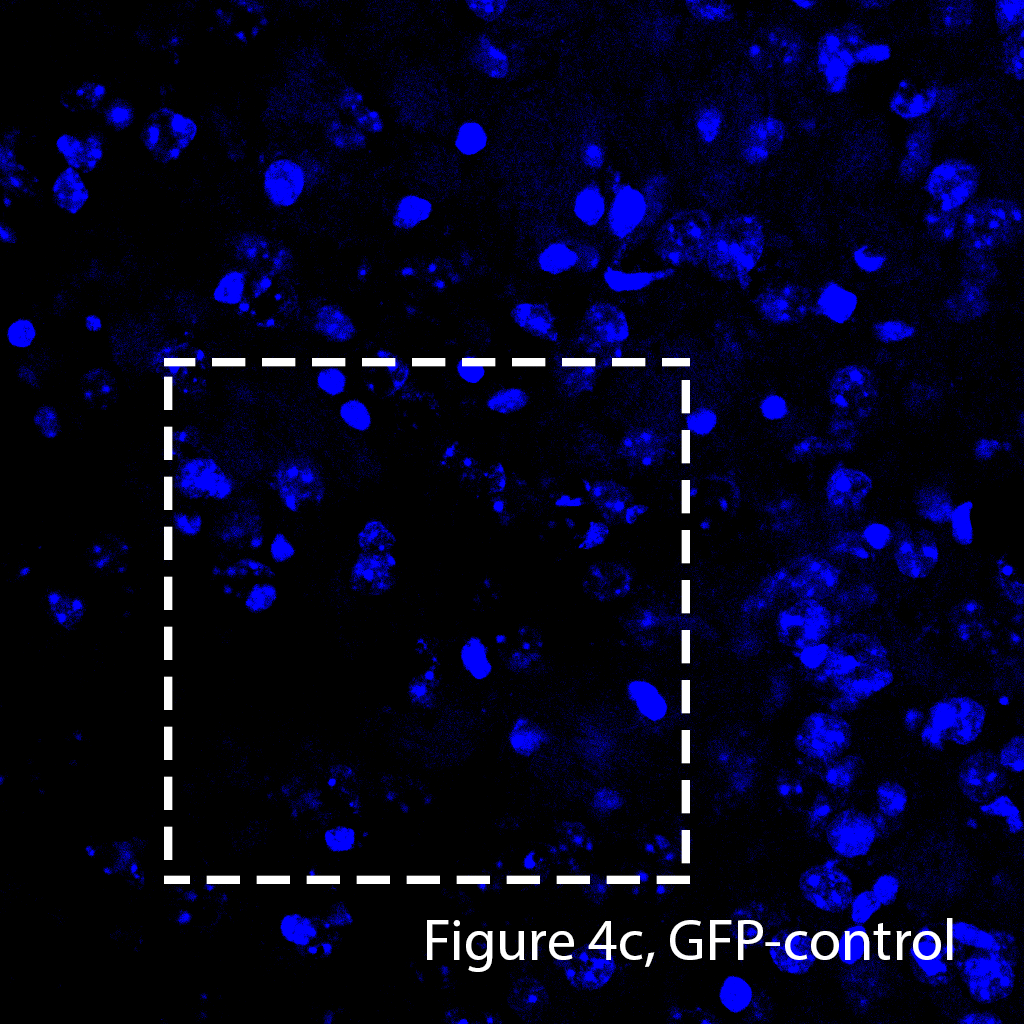

Supplement: Supplementary file 6 — Source Data for Figure 4 [file EMMM-14-e14797-s008.zip › Figure4c_GFPcontrol_DAPI.tif]

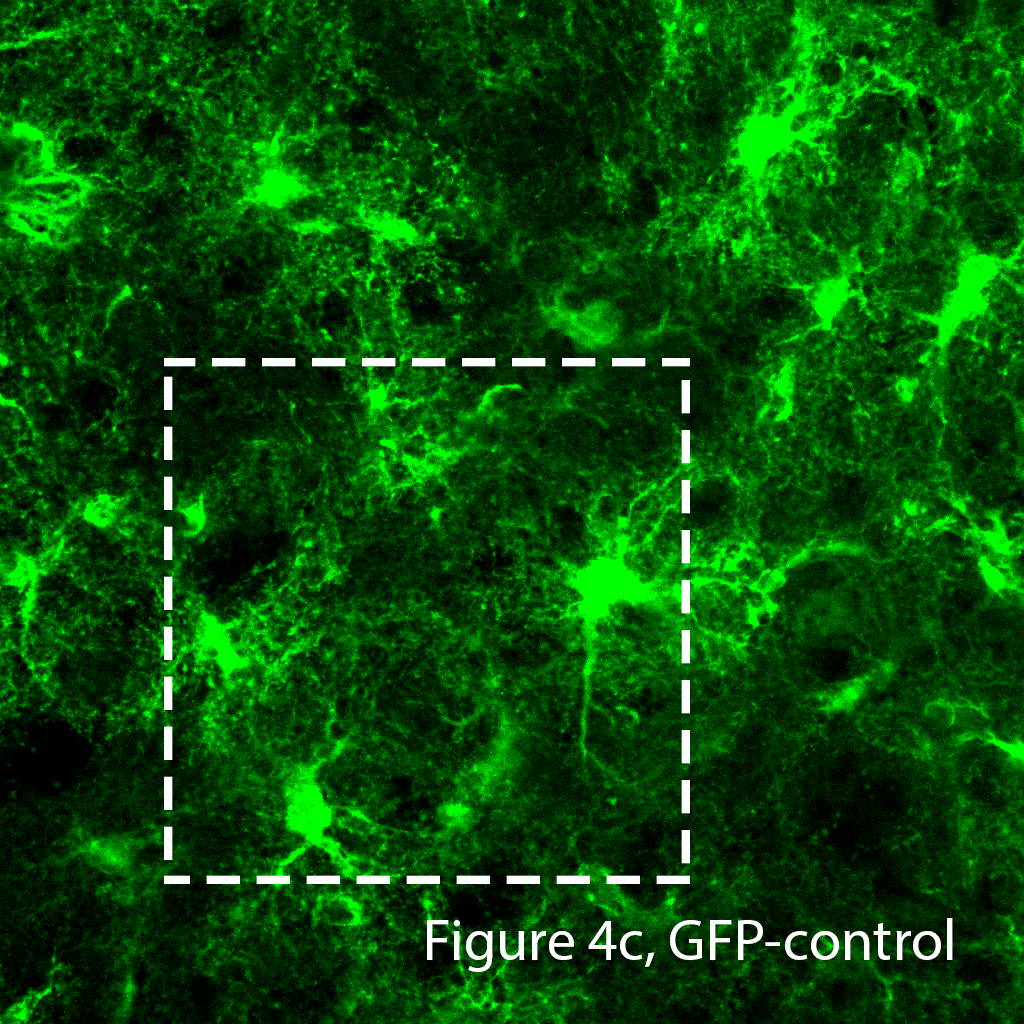

Supplement: Supplementary file 6 — Source Data for Figure 4 [file EMMM-14-e14797-s008.zip › Figure4c_GFPcontrol_GFP.tif]

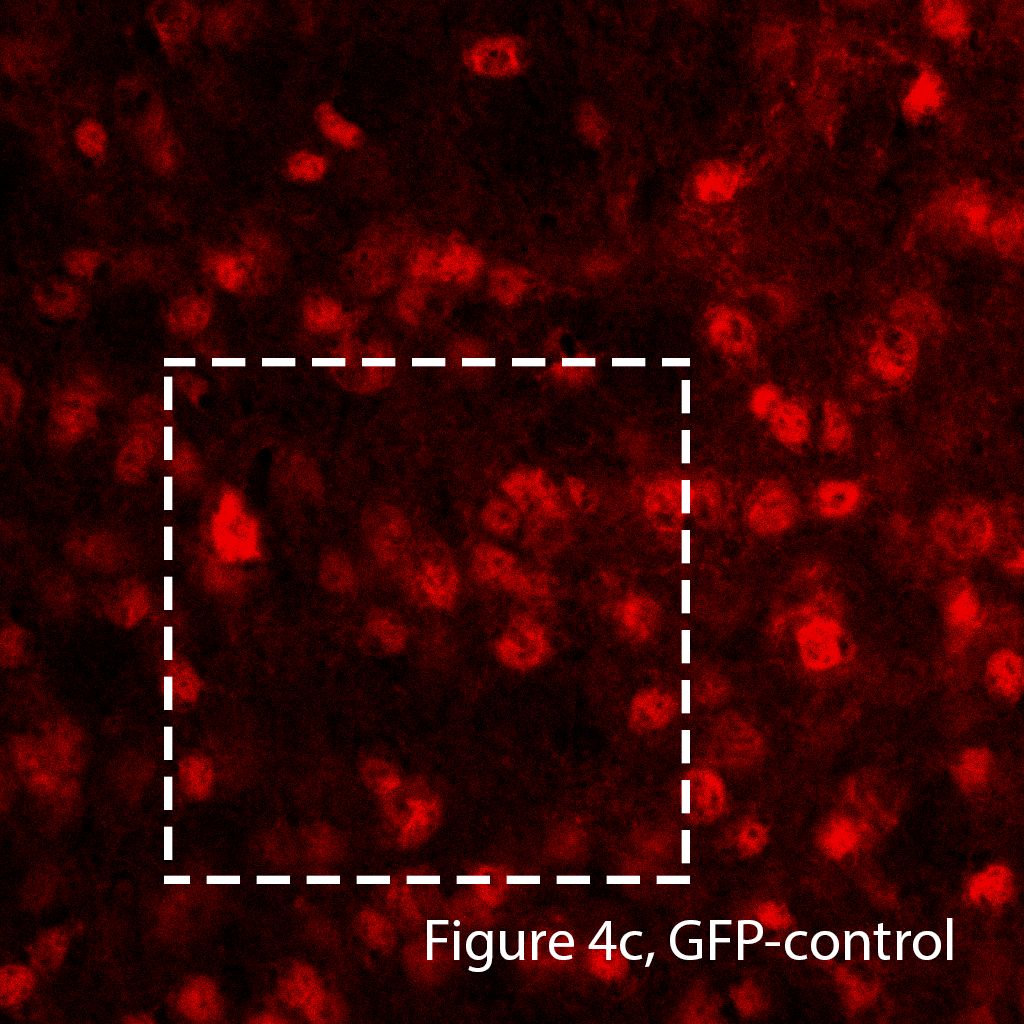

Supplement: Supplementary file 6 — Source Data for Figure 4 [file EMMM-14-e14797-s008.zip › Figure4c_GFPcontrol_NeuN.tif]

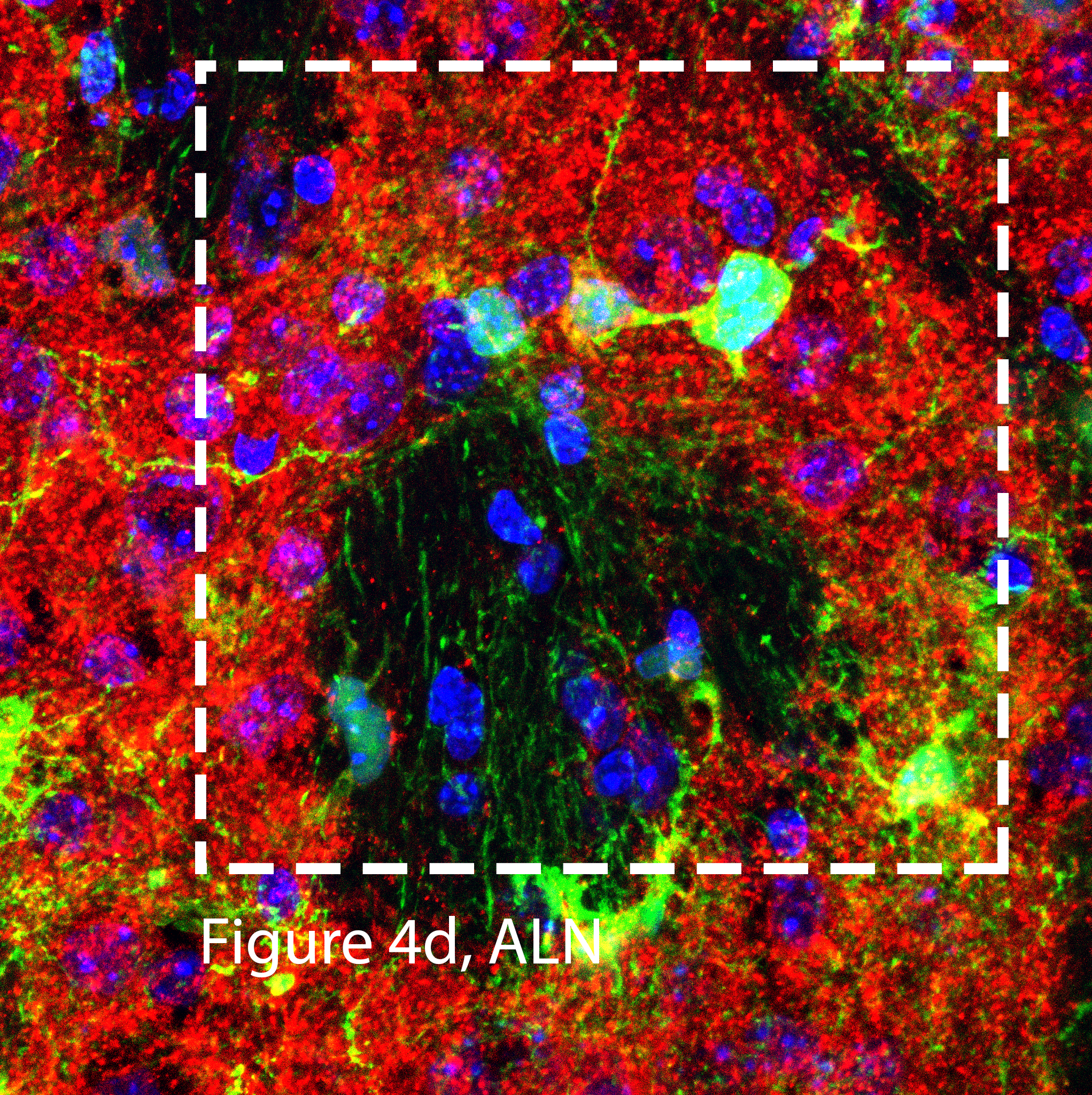

Supplement: Supplementary file 6 — Source Data for Figure 4 [file EMMM-14-e14797-s008.zip › Figure4d_ALN_composite.tif]

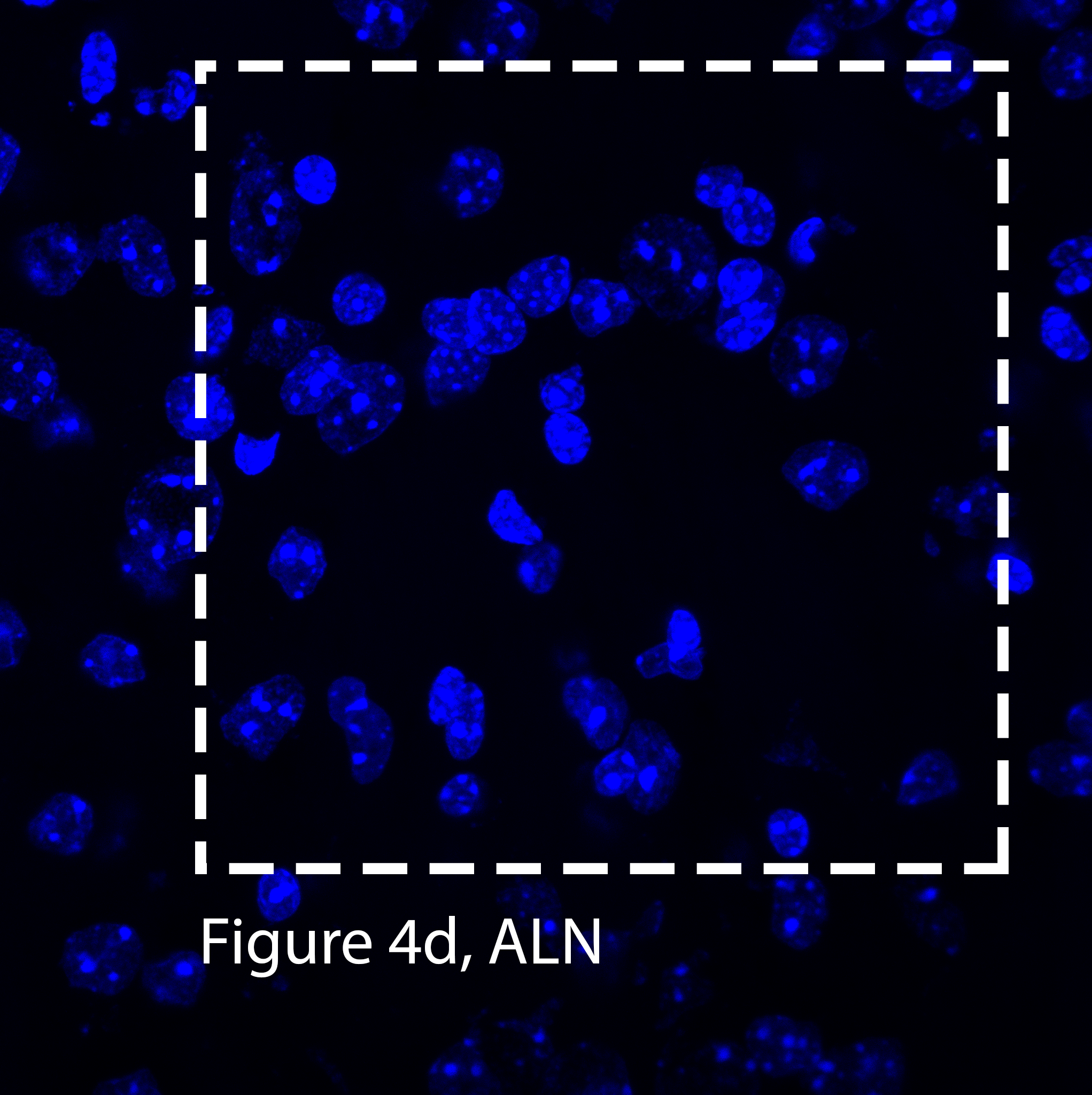

Supplement: Supplementary file 6 — Source Data for Figure 4 [file EMMM-14-e14797-s008.zip › Figure4d_ALN_DAPI.tif]

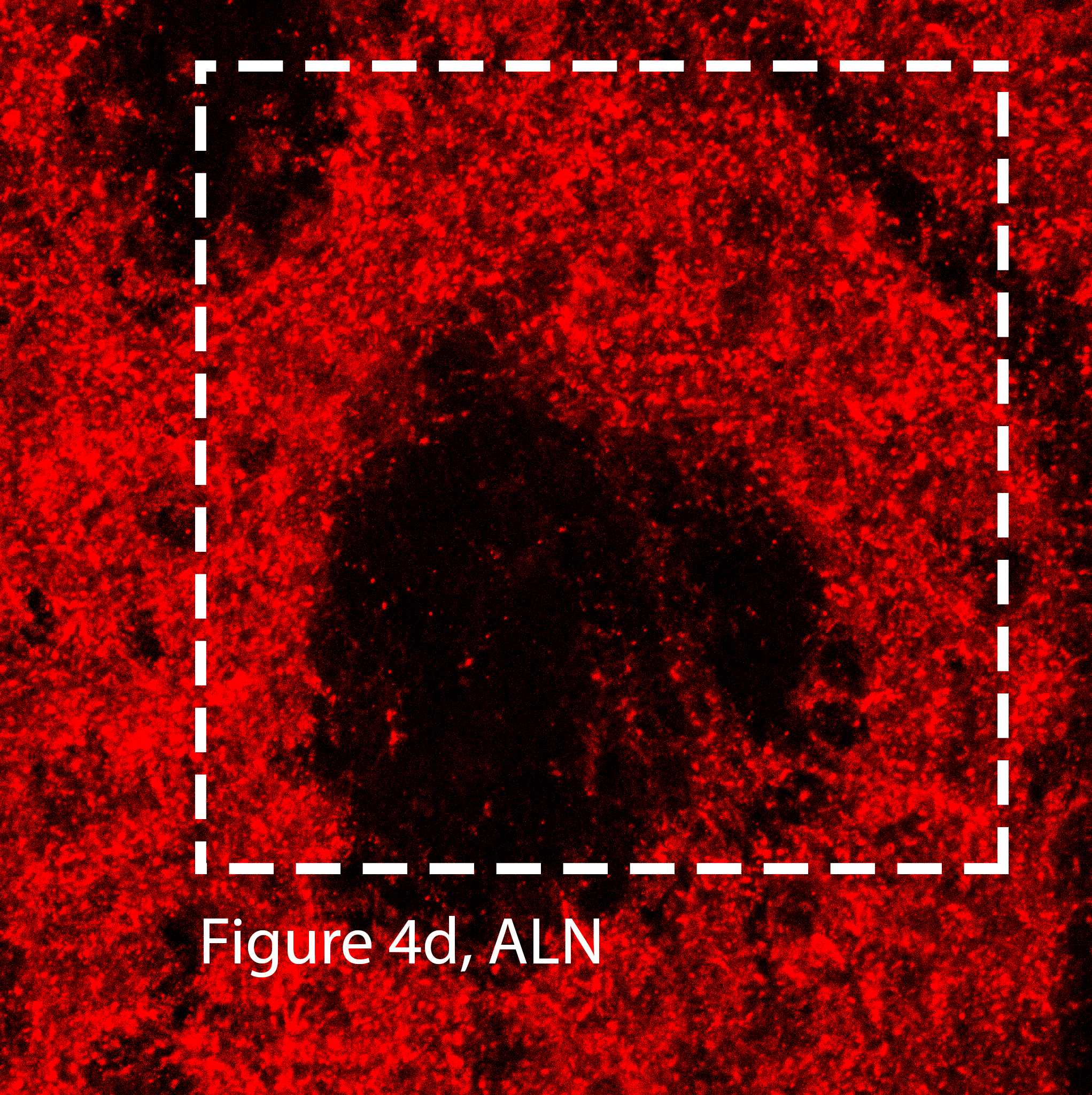

Supplement: Supplementary file 6 — Source Data for Figure 4 [file EMMM-14-e14797-s008.zip › Figure4d_ALN_GAD6567.tif]

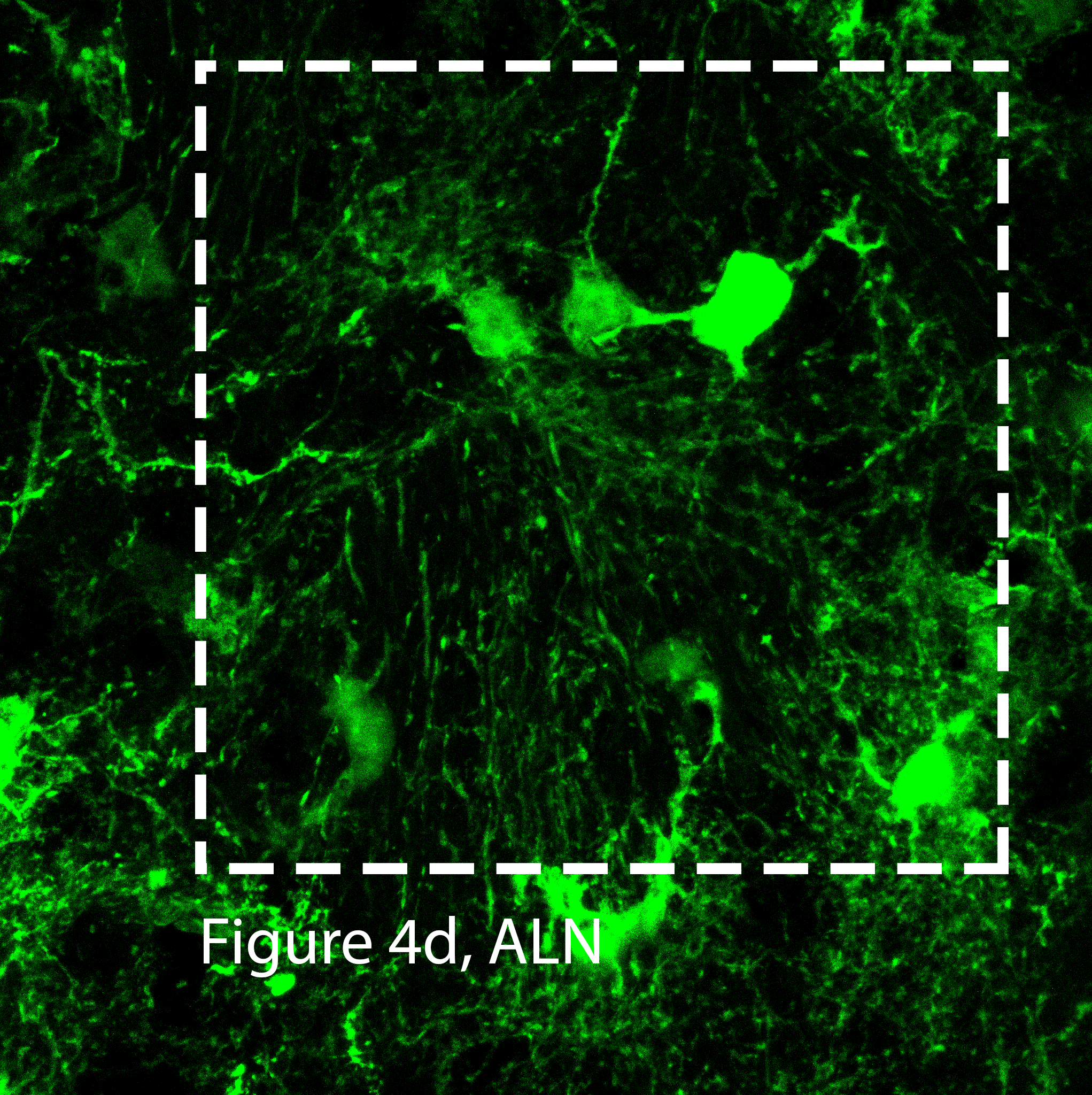

Supplement: Supplementary file 6 — Source Data for Figure 4 [file EMMM-14-e14797-s008.zip › Figure4d_ALN_GFP.tif]
