# Supplementary material for: Parkinson's disease motor symptoms rescue by CRISPRa‐reprogramming astrocytes into GABAergic neurons
Source: EMBO Mol Med. 2022 Apr 4;14(5):e14797. doi: 10.15252/emmm.202114797 (PMC9081909; doi:10.15252/emmm.202114797)
Supplement: Supplementary file 8 [file EMMM-14-e14797-s003.pdf]

EMBO Molecular Medicine

# Parkinson's disease motor symptoms rescue by CRISPRa-reprogramming astrocytes into GABAergic neurons

Jessica Giehrl-Schwab, Florian Giesert, Benedict Rauser, Chu Lan Lao, Sina Hembach, Sandrine Lefort, Ignacio Ibarra Del Río, Christina Koupourtidou, Malte Luecken, Dong-Jiunn Truong, Judith Fischer-Sternjak, Giacomo Masserdotti, Nilima Prakash, Jovica Ninkovic, Sabine Hölter, Daniela Vogt-Weisenhorn, Fabian Theis, Magdalena Götz, and Wolfgang Wurst
**DOI: 10.15252/emmm.202114797**

*Corresponding author(s): Wolfgang Wurst (wurst@helmholtz-muenchen.de)*

## Review Timeline:

| | |
|---|---|
| Submission Date: | 2nd Jul 21 |
| Editorial Decision: | 26th Jul 21 |
| Revision Received: | 11th Jan 22 |
| Editorial Decision: | 4th Feb 22 |
| Revision Received: | 8th Mar 22 |
| Accepted: | 10th Mar 22 |

*Editor: Jingyi Hou*

## Transaction Report:

(Note: With the exception of the correction of typographical or spelling errors that could be a source of ambiguity, letters and reports are not edited. Depending on transfer agreements, referee reports obtained elsewhere may or may not be included in this compilation. Referee reports are anonymous unless the Referee chooses to sign their reports.)

26th Jul 2021

Dear Prof. Wurst,

Thank you for submitting your work to EMBO Molecular Medicine. We have now heard back from the three referees who evaluated your manuscript. As you will see from the reports below, the referees acknowledge the potential interest and relevance of the study. However, they also raise a series of concerns about your work, which should be convincingly addressed in a major revision of the present manuscript.

Without reiterating all the points raised in the reviews below, some of the more substantial issues are the following:

1. Additional details and information requested by the Referees must be provided.
2. The exact numbers and quantifications (including the conversion efficiency) are required at several places.
3. The therapeutic implications need to be better discussed in light of Referees #1 and #3's concerns.
4. In particular, Referees #1 and #2 are concerned about the claim on the functional integration of astrocyte-derived neurons into the existing neural circuitry. During our pre-decision cross-commenting process (in which the referees are given a chance to make additional comments, including on each other's reports), Referee #3 suggested that the authors can "perform an optogenetics experiment where ChR2 is expressed in the new neurons. Photo stimulation of the new neurons followed by imaging or electrophysiology will be able to detect synaptic transmission to downstream cells in the striatum." Referees #1 and #2 agreed that the suggested optogenetic strategy would add significant value to the study. Therefore, we would encourage you to address this point in light of Referee #3's suggestion to enhance the conclusiveness of the study. However, this is not mandatory for publication, and if you decide not to address the raised concern experimentally, the claim in this regard needs to be toned down.

We would welcome the submission of a revised version within three months for further consideration. Please note that EMBO Molecular Medicine strongly supports a single round of revision. As acceptance or rejection of the manuscript will depend on another round of review, your responses should be as complete as possible.

EMBO Molecular Medicine has a "scooping protection" policy, whereby similar findings that are published by others during review or revision are not a criterion for rejection. Should you decide to submit a revised version, I do ask that you get in touch after three months if you have not completed it to update us on the status.

We are aware that many laboratories cannot function at full efficiency during the current COVID-19/SARS-CoV-2 pandemic and have therefore extended our "scooping protection policy" to cover the period required for a full revision to address the experimental issues. Please let me know should you need additional time, and also if you see a paper with related content published elsewhere.

Please read below for important editorial formatting and consult our author's guidelines for proper formatting of your revised article for EMBO Molecular Medicine.

I look forward to receiving your revised manuscript. Use this link to login to the manuscript system and submit your revision:
https://embomolmed.msubmit.net/cgi-bin/main.plex

Kind regards,
Jingyi

Jingyi Hou
Editor
EMBO Molecular Medicine

*****

When submitting your revised manuscript, please carefully review the instructions that follow below. We perform an initial quality control of all revised manuscripts before re-review; failure to include requested items will delay the evaluation of your revision.

We require:

1) A .docx formatted version of the manuscript text (including legends for main figures, EV figures and tables). Please make sure that the changes are highlighted to be clearly visible.

2) Individual production quality figure files as .eps, .tif, .jpg (one file per figure). For guidance, download the 'Figure Guide PDF' (https://www.embopress.org/page/journal/17574684/authorguide#figureformat).

3) A .docx formatted letter INCLUDING the reviewers' reports and your detailed point-by-point responses to their comments. As part of the EMBO Press transparent editorial process, the point-by-point response is part of the Review Process File (RPF), which will be published alongside your paper.

4) A complete author checklist, which you can download from our author guidelines (https://www.embopress.org/page/journal/17574684/authorguide#submissionofrevisions). Please insert information in the checklist that is also reflected in the manuscript. The completed author checklist will also be part of the RPF.

5) Please note that all corresponding authors are required to supply an ORCID ID for their name upon submission of a revised manuscript.

6) It is mandatory to include a 'Data Availability' section after the Materials and Methods. Before submitting your revision, primary datasets produced in this study need to be deposited in an appropriate public database, and the accession numbers and database listed under 'Data Availability'. Please remember to provide a reviewer password if the datasets are not yet public (see https://www.embopress.org/page/journal/17574684/authorguide#dataavailability).

In case you have no data that requires deposition in a public database, please state so in this section. Note that the Data Availability Section is restricted to new primary data that are part of this study.

7) For data quantification: please specify the name of the statistical test used to generate error bars and P values, the number (n) of independent experiments (specify technical or biological replicates) underlying each data point and the test used to calculate p-values in each figure legend. The figure legends should contain a basic description of n, P and the test applied. Graphs must include a description of the bars and the error bars (s.d., s.e.m.).

8) We would also encourage you to include the source data for figure panels that show essential data. Numerical data should be provided as individual .xls or .csv files (including a tab describing the data). For blots or microscopy, uncropped images should be submitted (using a zip archive if multiple images need to be supplied for one panel). Additional information on source data and instruction on how to label the files are available at

.

9) Our journal encourages inclusion of *data citations in the reference list* to directly cite datasets that were re-used and obtained from public databases. Data citations in the article text are distinct from normal bibliographical citations and should directly link to the database records from which the data can be accessed. In the main text, data citations are formatted as follows: "Data ref: Smith et al, 2001" or "Data ref: NCBI Sequence Read Archive PRJNA342805, 2017". In the Reference list, data citations must be labeled with "[DATASET]". A data reference must provide the database name, accession number/identifiers and a resolvable link to the landing page from which the data can be accessed at the end of the reference. Further instructions are available at .

10) We replaced Supplementary Information with Expanded View (EV) Figures and Tables that are collapsible/expandable online. A maximum of 5 EV Figures can be typeset. EV Figures should be cited as 'Figure EV1, Figure EV2" etc... in the text and their respective legends should be included in the main text after the legends of regular figures.

- For the figures that you do NOT wish to display as Expanded View figures, they should be bundled together with their legends in a single PDF file called *Appendix*, which should start with a short Table of Content. Appendix figures should be referred to in the main text as: "Appendix Figure S1, Appendix Figure S2" etc.

- Additional Tables/Datasets should be labeled and referred to as Table EV1, Dataset EV1, etc. Legends have to be provided in a separate tab in case of .xls files. Alternatively, the legend can be supplied as a separate text file (README) and zipped together with the Table/Dataset file.

See detailed instructions here:

.

11) The paper explained: EMBO Molecular Medicine articles are accompanied by a summary of the articles to emphasize the major findings in the paper and their medical implications for the non-specialist reader. Please provide a draft summary of your

article highlighting

- the medical issue you are addressing,

- the results obtained and

- their clinical impact.

This may be edited to ensure that readers understand the significance and context of the research. Please refer to any of our published articles for an example.

12) For more information: There is space at the end of each article to list relevant web links for further consultation by our readers. Could you identify some relevant ones and provide such information as well? Some examples are patient associations, relevant databases, OMIM/proteins/genes links, author's websites, etc...

13) Author contributions: the contribution of every author must be detailed in a separate section (before the acknowledgments).

14) A Conflict of Interest statement should be provided in the main text.

15) Every published paper now includes a 'Synopsis' to further enhance discoverability. Synopses are displayed on the journal webpage and are freely accessible to all readers. They include a short stand first (maximum of 300 characters, including space) as well as 2-5 one-sentences bullet points that summarizes the paper. Please write the bullet points to summarize the key NEW findings. They should be designed to be complementary to the abstract - i.e. not repeat the same text. We encourage inclusion of key acronyms and quantitative information (maximum of 30 words / bullet point). Please use the passive voice. Please attach these in a separate file or send them by email, we will incorporate them accordingly.

Please also suggest a striking image or visual abstract to illustrate your article as a PNG file 550 px wide x 300-600 px high.

EMBO Molecular Medicine has a "scooping protection" policy, whereby similar findings that are published by others during review or revision are not a criterion for rejection. Should you decide to submit a revised version, I do ask that you get in touch after three months if you have not completed it, to update us on the status.

***** Reviewer's comments *****

Referee #1 (Remarks for Author):

In this manuscript, Giehrl-Schwab and colleagues report the development of two genetic tools to reprogram astrocytes into neurons in vivo by driving the overexpression of two combinations of transcription factors via a dead Cas9 (dCas9) coupled to transcriptional activators and a set of five sgRNAs. First, the authors pack this molecular tool in a knock-in mouse, then they utilize an adeno-associated viral (AAV)-method to enable cell reprogramming independent from genetic modification of the recipient. The authors validate their systems in a 6-OHDA (6-hydroxydopamine)-induced model of Parkinson disease (PD), showing that both strategies succeed at reprogramming astrocytes into GABAergic neurons and that this conversion mitigates voluntary motor behavior aspects in the animal model used.
This work presents a therapeutic strategy to reprogram cells into cellular types that have been compromised by PD. Although the approach described here is promising, several theoretical and technical issues should be solved before recommending it for publication.

1. The authors do not justify why AAV2/5 was chosen for the study. Although this choice is supported by works reporting the high tropism of AAV2/5 for astrocytes, the authors do not motivate it. The works by Ortinski, 2010, Nat. Neurosci, and Xie at al., 2010, Neuroscience, on this subject should be cited.

2. The authors develop AAV-dCAS "to enable therapeutic applications independent of a genetically modified recipient". Nevertheless, experiments with the AAV-dCAS system were performed in the transgenic Gfap-Cre mouse line, which enables the reconstitution of a functional dCas9 by inversion of N-dCas9aa1-573-N-intein via Cre-Lox recombination. The requirement of a transgenic mouse line for the system to be operational defeats the purpose stated by the authors. Can the system be engineered to ensure astrocyte-specific targeting independently of the genetic background of the recipient? How?

3. The statement "the CRISPRa platform allows multiplexed activation of many endogenous genes solely 300 by introducing specific sgRNAs..." fails to mention that the strategy relies on simultaneous introduction of a dCAS activator with the sgRNAs.

4. A surprising finding is that neither transcription factor combination nor reprogramming tool resulted in the conversion of glial cells into dopaminergic neurons, a result in contrast with the initial expectations of the study. In this light, I suggest to include Supplementary Figure 9a among the main figures.

5. The efficiency of dCAM and AAV-dCas platforms in converting GFP+GFAP cells into GFP+Neu+ appears to be similar (Figure 2b and 2c compared to Figure 4b and 4c). This is unexpected since the successful reconstitution of AAV-dCas requires the simultaneous transduction by four AAV particles followed by intein-mediated protein splicing of dCas9. It would have been interesting to compare the average expression of Cas9 and gRNAs in dCAM and AAV-dCas9. In light of the amplification undergone by AAV genomes, is the presence of the AAV carrying SAM required to drive the activation of the targeted TF, or can it be circumvented by multiplication of the intein-Cas9 and GFP harboring viral genomes?

6. "Although the author claim that "a comprehensive IHC analysis 190 was performed including both transcription factor combinations (ALNe-218 and ALN) and both 191 reprogramming tools (dCAM and AAV-dCAS)", the figures used to support this claim (Supplementary Figure 9 and Supplementary Figure 10) are limited to AAV-dCas9 only. Furthermore, Figure 4D refers solely to AAV-dCas ALN.

7. Has any comparison between the transcriptomic profile of GFP+neurons (presumably reprogrammed astrocytes) and the transcriptional outputs of GFP- neurons and at GFP- astrocytes been made? Characterization of the calcium channels equipment of reprogrammed astrocytes could strengthen and validate the observations of electrophysiological recordings.

8. What is the percentage of astrocytes expressing a functional dual Cas9 transactivator that have not been transduced by rAAV2/5? What is the percentage of failed Cre-mediated recombination of the GFP marker harbored by the plasmid? How do you justify the discrepancy in the percentage of GFP+ cells between the two astrocytic subclusters? Does it correlate to the level of expression of GFAP?

9. The electrophysiological data presented are suggestive of neural integration of reprogrammed astrocytes, but stronger evidence is needed to conclude that the physical connections observed correspond to integration of reprogrammed cells into functional integration in the neural circuitry of the striatum. Although likely impractical, electrophysiological recordings of behaving mice would have been powerful in this respect.

10. Given the failure to reprogram astrocytes into dopaminergic neurons despite the activation of transcription factors associated with this neuronal cell type, what speculations would the authors make on the combination of activated genes required to succeed at reprogramming astrocytes into dopaminergic neurons? If reprogramming is dependent on the local environment, could the combination of genes successful at converting astrocytes in GABAergic neurons in the murine striatum fail when employed in humans? What would the therapeutic implications be?

Referee #2 (Comments on Novelty/Model System for Author):

See my review

Referee #2 (Remarks for Author):

This work tested two strategies to induce neuronal conversion from astrocytes by inducing a pool of endogenous neuronal-specific transcription factors. One utilizes the CRISPRa (dCas9-VP64) from knock-in mice in the Rosa26 locus and the other uses a split dCas9-Interin delivered by AAV, the latter of which would be more amendable for future therapeutic applications. Two sets of TF combinations were tested: ALN for Ascl1, Lmx1a, and Nr4a2/Nurr1 and ALNe-218 for Ascl1, Lxm1a, NeuroD1, and miR-218. Both were originally designed to induce dopaminergic neurons (DA) in striatum.

By single cell sequencing, most GFP+ cells were found to be astrocytes with control treatment, but neurons after reprogramming. The LAN combination appears more efficient than ALNe-218 in neuronal conversion and LNA-converted neurons are also more mature compared to those converted wtih ALNe-218. Unexpectedly, most converted neurons are GABAergic, instead of originally intended for DA neurons Dopaminergic, and puzzlingly, those GABAergic neurons appear Darpp32 negative, the main population of neurons in striatum. ALN treatment appear to show a degree of behavioral benefits in a 6-OHDA model, none of which is directly related to dopamine-dependent motor symptoms. This manuscript reports many interesting findings. However, many issues need to be addressed before publication:

Main concerns:

1. It is not well explained for the rationale to use the split Interin system, which does not seem necessary. Is it used to ensure

neuronal conversion from astrocytes infected by multiple AAVs? It is also curious how many GFP+ cells are also positive for dCas9-VP64? This result is important for the estimation of conversion efficiency.

2. What would be the neuronal subtypes if converted with ALN or ALN2-218 in vitro? This would help reconcile with the in vivo results.

3. There are ~1000 GFP+ cells per section. Based on the data in Fig. 2b and 2c, the percentage of GFP+/GFAP+ astrocytes is 50% with ALN and the percentage of GFP+/NeuN+ neurons is 25%. As such GFP+ positive cells are rather high, it appears that the number of infected cells is too high with just 1 ul of combined viruses. What is the percentage of newly converted neurons compared to endogenous neurons in the same brain section?

4. It appears incorrect to claim that the larger input resistance "indicating that ALN reprogrammed cells acquire a higher amount of ion channels". Low input resistance of astrocytes is mediated by multiple ion channels and gap junctions and the increase in input resistance after neuronal conversion likely reflects the switch from astrocyte-enriched ion channels to neuronal-specific ion channels. Thus, the detected changes may result from switched channels, not increased amount of the existing channels. Those channels should be further characterized by using specific channel blockers.

4. Fig. 4d lacks quantification, although stated in the text that "the majority colocalized with the marker of GABAergic neurons". Somewhat surprisingly, little were Darpp32+ MSNs, which is the major population of neurons in striatum. In fact, the data indicate that the converted neurons do not seem to belong to any well-known major subtypes of GABAergic neurons. This seems incompatible to the postulated regional specificity.

5. The scRNA-seq experiments revealed that a small fraction of GFP+ neurons also express Ascl1. As this TF is normally expressed in neuronal progenitors, does this result mean because the genes activated by AAV have returned to their grand state at the time of analysis?

6. From the scRNA-seq data, do all (if not, which fraction) astrocytes show elevated GFAP indicative of their state of reactive gliosis?

7. Is spontaneous action potential sufficient to indicate the integration within the existing neuronal network?

8. Most converted neurons are GABAergic, not DAergic. Moreover, ALNe-218 was previously shown to be more efficient than ALN in inducing DA neurons in striatum. The opposite was observed here. Need some discussion.

Other points:

1. FRT recombination sites are diagrammed in Fig. 1a, but not described in the text.

2. Fig. S1b: missing qPCR analysis of miR-218.

3. The legend in Fig. S2 does not match with the data (missing legend for b; current legend for b appears to be c).

4. Fig. S3 is a duplicate of main Fig. 3. The missing of the real data in Fig. S3 makes it impossible to verify various statements made in the text.

5. The legend of Fig. 6 indicates the panel a is for ALN and b for ALNe-218, while the data displayed are opposite.

6. The numbers in Fig. 5c are confusing, difficult to match the numbers stated in the text. For example, it was stated that 11 out of 17 astrocytes were GFP+ and Ascl1+ in the astrocyte cluster. However, in the second row, right, it only labeled 66 (blue)/17 (green) in the middle. It would be helpful to indicate the numbers separately in the astrocyte and neuron clusters.

7. For the benefits of future readers, it would be helpful to describe how the expression of Ascl1 and Mytl1 suggests a transition from astrocytes to neurons.

8. The information of lentiviruses used to deliver dCAS system in vitro is missing. Which vector and promoter were employed? Are they Cre dependent? Why there is expression of dsRed (Fig. 3c, left panel) in the lenti-based dCAS system?

Referee #3 (Remarks for Author):

Giehrl-Schwab and colleagues report the application of CRISPR-mediated gene activation to reprogram striatal astrocytes into GABAergic interneurons in vivo. Using either transgenic mice or split AAV systems to express dCas9 activators, the authors show that they can encode multiple sgRNAs into AAV vectors and achieve activation of multiple genes in astrocytes and drive

the transdifferentiation of neurons. The extent of reprogramming and resulting behavioral rescue seem modest but the proof of concept is useful for researchers who are working in this area. The transgenic mouse will also likely to be a useful tool. I support publication of this manuscript pending the authors addressing the below comments.

Figure 1a.
- What is the polyA signal used? What is SAM-P2A and does it carry a stop codon?
- For B6.Cg-Tg(Gfap-cre) mouse, is this a knockin? Or is it a transgenic mouse with a GFAP promoter fragment? How big is the promoter fragment?
- For AAV (rAAV2/5): is there a polyA signal for GFP? Is there WPRE?

Figure 1b.
- Which promoter is used to drive which guide RNA?
- If there are six promoters, are these data from two different vectors each carrying three guide RNAs? What are encoded by the other three pol3 promoters?

Figure 2b.
- Why is the GFP control % only around 90%? Is there 10% of non-GFAP-specific expression of Are?

Figure 2c.
- Similar to question for 2b, why is there GFP fluorescence in NeuN+ cells in the control condition?
- Given the leakiness of targeting to GFAP+ cells, I suggest the authors conduct an in vitro experiment with cultured astrocyte cells and provide the efficiency of neuronal conversion in that setting.

Figure 4:
- What is the co-transduction efficiency for the same cell when using multiple vectors?
- Can the authors provide larger field of view images to give a sense of how widely reprogrammed cells are distributed?

Figure 7:
- How many new neurons are generated in the brains of the mice undergoing these behavioral tests?

Overall comments:
- The behavioral rescue seems modest. Can the authors comment on how they envision this strategy, or derivatives, being applied at a high enough efficiency in a therapeutic context in patients?

**Point by point answers:**

**Editor**

The exact numbers and quantifications (including the conversion efficiency) are required at several places.

We added additional quantifications and further data addressing the respective questions to the manuscript as well as in the response letter (for details please refer to the specific answers to the referee's questions below).

The therapeutic implications need to be better discussed in light of Referees #1 and #3's concerns.

The discussion part of the manuscript has modified in this regard (for details please refer to the specific answers to the referee's questions below)

(*see line 331ff*)

In particular, Referees #1 and #2 are concerned about the claim on the functional integration of astrocyte-derived neurons into the existing neural circuitry. During our pre-decision cross-commenting process (in which the referees are given a chance to make additional comments, including on each other's reports), Referee #3 suggested that the authors can "perform an optogenetics experiment where ChR2 is expressed in the new neurons. Photo stimulation of the new neurons followed by imaging or electrophysiology will be able to detect synaptic transmission to downstream cells in the striatum." Referees #1 and #2 agreed that the suggested optogenetic strategy would add significant value to the study. Therefore, we would encourage you to address this point in light of Referee #3's suggestion to enhance the conclusiveness of the study. However, this is not mandatory for publication, and if you decide not to address the raised concern experimentally, the claim in this regard needs to be toned down.

This crucial point is well taken. To characterize the functional integration of astrocyte-derived neurons is of highest interest and will provide important insights into the circuitries involved. However, to set up and conduct the corresponding experiments would require the implementation of a new animal protocol and will realistically take at least one to two years, which is far beyond the time frame of this revision. Nevertheless, we will definitely go down this road in the future and started to initiate the required administrative work. For the present manuscript, however, we modified the respective paragraphs in the electrophysiological part (*see line 258f, 270f*) as well as in the discussion (*see line 384ff*) and attenuated our interpretation of the data.

**Point by point answers:**

**Referee #1**

1. The authors do not justify why AAV2/5 was chosen for the study. Although this choice is supported by works reporting the high tropism of AAV2/5 for astrocytes, the authors do not motivate it. The works by Ortinski, 2010, Nat. Neurosci, and Xie at al., 2010, Neuroscience, on this subject should be cited.

Thank you for this comment. This missing important detail is crucial to understand that we are achieving this focused expression of our tools in glial cells with a two-fold control of cell type specificity: On the one side, the tropism of the AAV2/5 is already quite specific for the glial lineage, on the other side, using the GFAP-Cre mouse line ensures that the dCas9 system is almost solely active in astrocytes. We have added an additional sentence and citation in the results part to justify our choice: "For this, as well as for all other experiments, the rAAV2/5 serotype has been selected due to its known tropism for astrocytes (Ortinski et al., 2010; Xie et al., 2010). The AAV FLEx-GFP (GFP-control) has been used for the control group" (*see line 124ff*)

2. The authors develop AAV-dCAS "to enable therapeutic applications independent of a genetically modified recipient". Nevertheless, experiments with the AAV-dCAS system were performed in the transgenic Gfap-Cre mouse line, which enables the reconstitution of a functional dCas9 by inversion of N-dCas9aa1-573-N-intein via Cre-Lox recombination. The requirement of a transgenic mouse line for the system to be operational defeats the purpose stated by the authors. Can the system be engineered to ensure astrocyte-specific targeting independently of the genetic background of the recipient? How?

We apologize that especially this aspect was not elaborated comprehensively enough. Certainly, we aim to develop a potentially universal applicable AAV-based tool suitable for therapeutic intervention. To ensure cell type specific expression of the dCAS system, tissue- or cell-type specific promoters have to be used. To further support the applicability of the system as a therapeutic strategy we performed a prove of concept *in vitro* experiment using an astrocyte specific promoter driving the expression of the intein-split dCas9 (see *Response Figure 1*). Comparing the reprogramming efficacy after 16 days in culture exhibited a similar conversion rate using the astrocyte-specific promoter gfaABC1D - a fragment of the endogenous murine GFAP promoter (Lee et al., 2008) when comparing it with the ubiquitous promoter Tet-O (Tet inducible). We have chosen Tet-O for comparison since it is in our hands one of the strongest promoter for primary astrocytic cultures. Interestingly, as we have shown previously already for active Cas9 (Truong et al., 2015), also the efficacy of the dCAS system split into a N- and C-terminal part and reconstituted upon intein-mediated trans splicing is indistinguishable from the full length form in regard to reprogramming ability. Direct overexpression of Ascl1, nevertheless, is in this relatively short timeframe of 16 days of reprogramming even more efficient. Altogether, this suggests, that the system can be operational independent of the genetic background by using tissue-specific promoters. Furthermore, since dCAS is based on two AAVs encoding a split dCas9-V64, it would be possible to express the two protein parts using two different cell type-specific promoters, which would allow a further specification of the target cell type. Based on this we expanded the corresponding part of the discussion (*see line 334ff*).

**FIGURES FOR REFEREE REPORT NOT SHOWN.**

3. The statement "the CRISPRa platform allows multiplexed activation of many endogenous genes solely by introducing specific sgRNAs..." fails to mention that the strategy relies on simultaneous introduction of a dCAS activator with the sgRNAs.

We have rephrased the paragraph as follows: “In contrast, the CRISPRa platform allows multiplexed activation of many endogenous genes by introducing additional sgRNAs to the dCAM or dCAS system. Every gene, regardless of its size or complexity, can be activated with a fixed cargo size comprising of a polymerase III promoter and the respective gRNA. Furthermore, by this the endogenous transcriptional machinery can be co-opted to execute complex genetic splicing patterns” (*see line 329ff*).

4. A surprising finding is that neither transcription factor combination nor reprogramming tool resulted in the conversion of glial cells into dopaminergic neurons, a result in contrast with the initial expectations of the study. In this light, I suggest to include Supplementary Figure 9a among the main figures.

We fully agree - this result was initially very surprising for us and should be better highlighted. For this, we added GFP/TH double immunohistochemistry results to the dCas9 activator mouse analysis as a *new Figure 2d* and the corresponding description to the results part. Like this, we can raise this critical issue early in the manuscript and complement the dCAM analysis with this previously not shown data point. According to this, since now the absence of dopaminergic neurons in the dCAS setting is just a confirmation of the findings in the dCAM mouse, we would keep the corresponding data point in the supplement. The corresponding results part has been modified as well (*see line 148ff*).

5. The efficiency of dCAM and AAV-dCas platforms in converting GFP+GFAP cells into GFP+Neu+ appears to be similar (Figure 2b and 2c compared to Figure 4b and 4c). This is unexpected since the successful reconstitution of AAV-dCas requires the simultaneous transduction by four AAV particles followed by intein-mediated protein splicing of dCas9. It would have been interesting to compare the average expression of Cas9 and gRNAs in dCAM and AAV-dCas9. In light of the amplification undergone by AAV genomes, is the presence of the AAV carrying SAM required to drive the activation of the targeted TF, or can it be circumvented by multiplication of the intein-Cas9 and GFP harboring viral genomes?

We thank the reviewer for bringing these interesting points to discussion. Indeed, the two systems differ in the availability of different components. In the dCAM setting, dCas9-VPR and SAM are expressed in all GFAP-Cre positive cells throughout the organism. In the AAV-dCAS system, only GFAP-Cre positive cells of the target region infected with the two corresponding viruses express full length dCas9. Therefore, to determine the expression levels of Cas9 is without a doubt interesting, but will not be able to explain the similar reprogramming efficacy of the two platforms. The delivery of the gRNAs on the other hand is relatively similar between the two systems. In case of dCAM, all gRNAs are delivered on a single AAV vector, in case of the AAV-dCAS system, the Ascl1 gRNAs are encoded on the split Cas9 vectors while all other gRNAs are delivered on a single AAV vector as well (for a better overview please refer to the new *supplementary figure 17*). Based on this, one possible explanation for the observed similarities is, that the gRNA expression is indeed the limiting factor. As the referee suggested, it would be interesting to evaluate the actual level of expression. Unfortunately, in our single cell transcriptome analysis we were not able to detect the corresponding gRNAs since the protocol is focused on polyadenylated mRNA. Moreover, the restrictions of our animal protocol in regard to animal numbers did not allow us to produce an additional cohort for this purpose during the time of this revision. Nonetheless, the mode of gRNA delivery by relative similar AAV vectors and stereotactic injection is comparable between the two systems and may explain the relative similar rate of reprogramming observed.

Regarding the reviewers question on the necessity to utilize the SAM-expressing AAV vector, one has to emphasize, that in the AAV-dCAS system we utilize a dCas9 fused to VP64 and not to the longer VPR activation complex. As described in the results section *AAV based split-dCas9-activator system (AAV-dCAS) for endogenous gene activation* and in Supplementary Figure 6, we decided to focus on this combination since the SAM activator system alone is sufficient to provide robust gene induction. Nevertheless, as the reviewer correctly pointed out, even in the absence of the SAM expressing AAV vector, an induction of gene expression by dCas9-VP64 is possible. However, in previous experiments we compared the dCas9-VP64 to the SAM, VPR and the SAM split-dCas9-VPR system not only in regard to gene activation but also to its ultimate reprogramming potential. In these experiments, we have seen that dCas9-VP64 alone can reprogram primary astrocytes but with a very low efficacy (see *Response Figure 2*). Based on these results, we think that the SAM system can hardly be circumvented by multiplication of intein-Cas9-VP64.

To emphasis this important difference between dCAM and the AAV-dCAS approach, we added one additional sentence to the respective part in the discussion section of the manuscript (*see line 329ff*).

**FIGURES FOR REFEREE REPORT NOT SHOWN.**

6. "Although the author claim that "a comprehensive IHC analysis was performed including both transcription factor combinations (ALNe-218 and ALN) and both reprogramming tools (dCAM and AAV-dCAS)", the figures used to support this claim (Supplementary Figure 9 and Supplementary Figure 10) are limited to AAV-dCas9 only. Furthermore, Figure 4D refers solely to AAV-dCas ALN.

We have complemented the manuscript in this regard at specific passages: first of all, we added GFP/TH double immunohistochemistry results of dCAM model as a new *Figure 2d* which complements the AAV-dCAS data of *Supplementary Figure 9a*. The new Figure 4e now shows a quantification of co-localisation with the GABAergic marker GAD6567 for both models. Furthermore, *Supplementary Figure 10 a, b* is showing results for the dCAM model while *Supplementary Figure 10 c,d* is showing the same analysis for the AAV-dCAS model.

7. Has any comparison between the transcriptomic profile of GFP+neurons (presumably reprogrammed astrocytes) and the transcriptional outputs of GFP- neurons and at GFP- astrocytes been made? Characterization

of the calcium channels equipment of reprogrammed astrocytes could strengthen and validate the observations of electrophysiological recordings.

This point is well taken. As the reviewer suggested we aimed to obtain a quantitative transcriptomic profile between the induced neurons. However, the low number of GFP+ neurons do not allow significant statements. The low numbers obtained are based on technical limitations doing single cell transcriptomics of adult neuronal tissue. The general low rate of recovered neurons has also been observed by others (Tiklová et al., 2020) and is most probably caused by the complex morphology and integration of neurons in the adult rodent brain. If restricting the analysis to GFP+ cells in the neuronal cluster we end up with 21 cells in total, which is not enough to obtain enough RNA reads – especially of low expressed markers - to make quantitative statements. Indeed, to characterize the channel expressed by induced neurons is an interesting point which was partially also raised by referee #2. Please also refer to the answer of question number 4 of referee number 2.

8. What is the percentage of astrocytes expressing a functional dual Cas9 transactivator that have not been transduced by rAAV2/5? What is the percentage of failed Cre-mediated recombination of the GFP marker harbored by the plasmid? How do you justify the discrepancy in the percentage of GFP+ cells between the two astrocytic subclusters? Does it correlate to the level of expression of GFAP?

Since both in the dCAM as well as in the AAV-dCAS system, the respective gRNAs are transmitted by AAVs, no astrocyte expresses a fully functional dCas9 activator without being transduced by rAAV2/5. Nevertheless, as the reviewers question indicates, in the dCAM x GFAP-cre mouse, every GFAP+ astrocyte expresses the SAM and dCas9-VP64. Given the fact, that efficient binding of at least two dCas9-VPR in close proximity to the transcriptional start site of a gene is necessary to induce robust expression, it remains elusive, if SAM and dCas9-VP64 without appropriate gRNA have a noticeable effect.

When analyzing the raw date of the single cell transcriptome experiment, we could also detect GFP reads matching to unrecombined flexed reporter. However, the vast majority, around 80%, of all GFP positive cells express the correctly recombined GFP (see *Response Figure 3*). For the further analysis, we included only reads mapping to the correctly recombined GFP reporter. The overall high amount of GFP positive cells in the GFP control as well as in the reprogramming conditions confirmed as well, that the Cre-mediated recombination is efficient.

As we stated in the manuscript, cluster-specific up-regulation of marker genes allowed unsupervised separation of neurons and astrocytes into four subclusters. The two astrocytic subclusters differ, as the reviewer correctly pointed out, in the amount of GFP-positiv cells. This is exactly why we decided to show the single-cell data not with unseparated astrocytic and neuronal clusters but with the subclustering. This subclustering indicates indeed that there might be astrocytes with differences in regard to their reprogramming potential. The underlying molecular markers can be found in *Figure 5b* and *Supplementary Figure 13b*. One of the differences might be, as the reviewer suggests, the level of GFAP expression; nevertheless, we did not find a significant difference between the GFAP expression between the two subclusters.

**FIGURES FOR REFEREE REPORT NOT SHOWN.**

9. The electrophysiological data presented are suggestive of neural integration of reprogrammed astrocytes, but stronger evidence is needed to conclude that the physical connections observed correspond to integration of reprogrammed cells into functional integration in the neural circuitry of the striatum. Although likely impractical, electrophysiological recordings of behaving mice would have been powerful in this respect.

We agree that an *in vivo* functional study of the integration of reprogrammed astrocytes into the neural circuitry would be a valuable information. However, this approach would be very challenging. We see that the electrophysiological data shown here is relevant since we demonstrated that reprogrammed astrocytes exhibit hallmark neuronal features for electrical signal integration and propagation, i.e., presence of presynaptic currents and firing response to current injection, which show the activity of ionotropic receptors and functional voltage-gated sodium channels, respectively. These are important evidence supporting that reprogrammed astrocytes are able to be integrated into the neural circuitry. In a follow up study we will perform electrophysiological recordings on behaving mice.

10. Given the failure to reprogram astrocytes into dopaminergic neurons despite the activation of transcription factors associated with this neuronal cell type, what speculations would the authors make on the combination of activated genes required to succeed at reprogramming astrocytes into dopaminergic neurons? If reprogramming is dependent on the local environment, could the combination of genes successful at converting astrocytes in GABAergic neurons in the murine striatum fail when employed in humans? What would the therapeutic implications be?

As the referee correctly pointed out, one of the initial aim of the study – to generate dopaminergic neurons - did fail. Nevertheless, we achieved a significant amelioration of the toxin-induced behavioral alterations. The observation, that the factor combinations ALN and ALNe218 are capable to reprogram astrocytes into dopaminergic neurons *in vitro* is undisputed. We could confirm this using the ALN combination in a classical overexpression set-up (Theodorou et al., 2015). Surprisingly, when utilizing the CRISPRa system, both in vitro (see *Response Figure 4*) as well as in vivo, we observe the generation of GABAergic neurons instead. The most obvious difference, is of course the utilized genetic tool. Although we get reliable and robust induction of the target genes, the total level of expression is significant lower compared to overexpression using heterologous promoters. In the beginning of the project we speculated, that these more endogenous levels of expression might be beneficial for the reprogramming process. Our data clearly demonstrates that these levels are sufficient to induce cellular reprogramming, however, it seems not to be sufficient to reprogram striatal astrocytes into an ectopic neuronal fate, e.g. midbrain DA neuron in the striatum. Nevertheless, this "gentle" reprogramming approach leads to functional GABAergic neurons and amelioration of phenotypes and is therefore a further indication that the GABAergic system is, besides or in complementation to the dopaminergic system, an important target for further therapeutic interventions. We expanded the discussion in this regard in the revised version of the manuscript (*see line 359ff*).

Indeed, since reprogramming seems to be highly dependent on the local environment, the translation to the human system could be challenging. Nevertheless, we are convinced that our main finding, the modulation of the striatal circuits by introducing novel GABAergic neurons, is valid in the human system as well. Moreover, the work from Lozovaya and colleagues indicates an even stronger influence of the impairment of the GABAergic component in PD pathogenesis in primates compared to rodents (Lozovaya et al., 2018). Still, we are well aware of the challenges to replace lost neurons by reprogramming astrocytes into specific neuronal subtypes – the route towards a therapeutic implication is still very long. For the moment, we are convinced that our system is an extension of the genetic tools for targeting various model organisms (like pig or non-human primates) and eventually for therapeutic application in patients. We modified the introduction on this, in order to tone down the therapeutic aspect (*see line 71ff*).

**Referee #2**

*Main concerns:*

1. It is not well explained for the rationale to use the split Interin system, which does not seem necessary. Is it used to ensure neuronal conversion from astrocytes infected by multiple AAVs? It is also curious how many GFP+ cells are also positive for dCas9-VP64? This result is important for the estimation of conversion efficiency.

We thank the referee for pointing out this shortcoming. It is true, that under certain conditions, the packaging limit of the AAV can be circumvented to fit a CRISPRa system onto a single AAV vector. Nevertheless, we are convinced that the modifications e.g. using small Cas9 orthologues from *Campylobacter jejuni* using more complex PAMs (5'-NNNNACAC-3' and compact promoters are detrimental in regard to activation efficacy. Furthermore, as the reviewer correctly indicated, the split-intein system ensures, that active Cas9 protein is only present in cells infected by multiple AAVs. The AAV-dCAS system presented here, represent a flexible system which can be modified in many aspects. Besides the feature of multiplexing, it allows the application of complex, cell-type specific promoters, which will most likely be necessary for clinical applications. On top of that, the split design would allow the combination of two distinct promoters for each of the Cas9 peptides respectively. Like this it will be possible to achieve a maximum of tissue specific expression.

To give the reader a better insight into our rationale to use the split-intein system, we expanded the corresponding paragraphs in the discussion part of the manuscript as follows: "Using the intein-split approach to circumvent the limitations of the AAV packaging limit, provides a higher degree of flexibility compared to approaches using compacted system e.g. based on engineered, short Cas9 versions or smaller Cas orthologs with complex PAMs. By contrast, the AAV-dCAS system is utilizing the well-characterized spCas9 and would allow the implementation of more complex, cell-type specific promoters. Due to this, the AAV-dCAS system is an independent and highly versatile tool which can be readily adapted for clinical application."

Regarding the questions of co-infection, we would courteously ask the referee to refer to our answer to referee #3, question figure 4.

2. What would be the neuronal subtypes if converted with ALN or ALN2-218 in vitro? This would help reconcile with the in vivo results.

We appreciate this reviewer comment. Following the suggestion, we performed in vitro reprogramming of primary murine astrocytes with different experimental settings: GFP control, the dCAS combination ALN, with gRNAs for *Ascl1*, *Lmx1a* and *Nurr1* In the ALN condition, we observed GFP positive neurons indicating successful reprogramming of astrocytes. For an estimation of the reprogramming efficiencies between the CRISPRa and overexpression, please refer to our response on question number 5 of reviewer 1 and *Response Figure 2*. Three weeks after transduction, the cultures were analysed by IHC. No GFP+/TH+ cell could be detected in any of the experimental groups in contrast to our previously performed *in vitro* reprogramming experiments using cDNA co-expression of the ALN factors (Theodorou et al., 2015). Interestingly, virtually all induced neurons of the reprogramming conditions dCAS ALN show GABAergic identity (see *Response Figure 4*). These *in vitro* results support our observations in the dCAM as well as AAV-dCAS setting *in vivo*. The discrepancy might be explained by the different levels of overexpression in the respective experimental settings. CRISPRa activation of the endogenous genes results in lower, but presumably more physiological levels of expression. On the other hand, cDNA-based overexpression, depending on the promoter, can induce much higher levels of expression. Moreover, based on the different technical mechanism, the kinetics of gene induction via CRISPRa is presumably different from the fast induction using cDNA approaches. All of this might contribute to the differences observed in the neuronal identity of the induced neurons between these strategies.

**FIGURES FOR REFEREE REPORT NOT SHOWN.**

3. There are ~1000 GFP+ cells per section. Based on the data in Fig. 2b and 2c, the percentage of GFP+/GFAP+ astrocytes is 50% with ALN and the percentage of GFP+/NeuN+ neurons is 25%. As such GFP+ positive cells are rather high, it appears that the number of infected cells is too high with just 1 ul of combined viruses. What is the percentage of newly converted neurons compared to endogenous neurons in the same brain section?

The reviewer is right that the total number of infected cells or GFP positive cells is rather high. To ensure an efficient co-infection we used high-titer AAV-virus with a physical titer of $3x10^{14}$ up to $5x10^{15}$ gc/ml (genome copies per milliliter). For a given injection, were we used 0.25µl of each AAV this converts to a total amount of viral genomes from about $1x10^{11}$ up to $1x10^{12}$. Cortical injections using similar viral titers and amounts resulted in comparable numbers of infected cells (Mattugini et al., 2019). We included this more detailed specification to the *Material and Method* section to allow the reader a better assessment of the viral load.

4. It appears incorrect to claim that the larger input resistance "indicating that ALN reprogrammed cells acquire a higher amount of ion channels". Low input resistance of astrocytes is mediated by multiple ion channels and gap junctions and the increase in input resistance after neuronal conversion likely reflects the switch from astrocyte-enriched ion channels to neuronal-specific ion channels. Thus, the detected changes may result from switched

channels, not increased amount of the existing channels. Those channels should be further characterized by using specific channel blockers.

We thank the reviewer for identifying this important point and agree that “larger input resistance” does not mean “higher amount of ion channels” but their open/close state. We see that characterizing the ion channels influencing the input resistance change in reprogrammed astrocytes would be interesting. However, our goal was to demonstrate that these cells are able to be integrated into the neural circuitry. For this, we showed that reprogrammed astrocytes receive synaptic inputs (presence of presynaptic currents) and trigger action potentials (functional activity of voltage-gated sodium channels), thus capable of sense synaptic activity and propagate electrical signals. As the reviewer pointed out, the membrane potential and the input resistance alone do not add significant further information; we moved this data to the Supplement Figure 15.

Furthermore, we have included here a complete panel of genes considered receptors, and their related expression levels detected in all cell clusters, and only GFP+ cells. In the heatmap below (see *Response Figure 5*), we can visually compare ion channels that are, based on expression associated to only astrocytes, and only to neurons (e.g. Cacng4, Cacnb3). In addition, it would be great to quantify the channel expression between GFP-positive cells of the ALN and control condition, however, the low amount of cells, picked up by our scRNA-seq experiment, does not allow a meaningful conclusion in this regard. Furthermore, similar to our answer to question number 9 from referee 1, we believe that a detailed characterization of the induced neurons, e.g. with specific channel blockers, and their putative integration into the existing networks would be interesting. Nevertheless, performing such experiments in a challenging *in vivo* setup would be beyond the scope of this study. Taken this into account, we modified the respective paragraphs in the electrophysiological part (*see line 258f, 270f*) as well as in the discussion (*see line 384ff*) and attenuated our interpretation of the data.

**FIGURES FOR REFEREE REPORT NOT SHOWN.**

4. Fig. 4d lacks quantification, although stated in the text that "the majority colocalized with the marker of GABAergic neurons". Somewhat surprisingly, little were Darpp32+ MSNs, which is the major population of neurons in striatum. In fact, the data indicate that the converted neurons do not seem to belong to any well-known major subtypes of GABAergic neurons. This seems incompatible to the postulated regional specificity.

As the reviewer suggested, we performed a quantification of the GABAergic marker GAD65/67 for *ALN* reprogrammed neurons in both the dCAM as well as the AAV-dCas setting. Both in neurons converted with the AAV-dCas as well as with the dCAM platform, we see around 90% overlap of GFP positive cells with a neuronal morphology with the GABAergic marker GAD6567. The results are included in the new *Figure 4e*. As the reviewer correctly pointed out, the induced neurons do not seem to belong to the most abundant GABAergic population of the striatum – the Darpp32 positive MSNs. The postulated regional specificity is referring to the general GABAergic fate of the neurons.

5. The scRNA-seq experiments revealed that a small fraction of GFP+ neurons also express Ascl1. As this TF is normally expressed in neuronal progenitors, does this result mean because the genes activated by AAV have returned to their grand state at the time of analysis?

We thank the referee for this comment and suggestion. Indeed we can detect Ascl1 scRNA-seq counts in both astrocyte and neuronal associated population (left violin plot, *see Response Figure 6*). Moreover, also in animals treated with the GFP-control AAV, Ascl1 expression can be detected to a certain extend in both clusters (see *Figure 5c*). Therefore, we conclude, that this expression is independent of ALN induction. As the referee pointed out, Ascl1 is in the adult mouse brain highly expressed in neural progenitors (Kim et al., 2011). For this reason, in the single cell transcriptomic experiment we excluded the subventricular zone as much as possible. Accordingly, also the immunohistological analyses excluded these regions (see *Supplement Figure 4b*). Also, we detect in a fraction of GFP+ neurons Ascl1 expression (right violin plot, *see Response Figure 6*). Since we cannot distinguish between endogenous or ectopic induced Ascl1 expression at this point of analysis, it is only speculative for us to say that these cells expressing Ascl1 are associated with genes or cells returning to a grand state. We would like to further deceiver the molecular marks of the newly generated neurons as well as the astrocytic population which appears more resistant to reprogramming. Nevertheless, to do so, the amount of reprogrammed neurons has to be increased. Last but not least, in this regard a direct effect of the 6-OHDA toxin model cannot be excluded since it has been shown that Ascl1 lineage cells are responsive to certain lesion (Zhang et al., 2011).

**FIGURES FOR REFEREE REPORT NOT SHOWN.**

6. From the scRNA-seq data, do all (if not, which fraction) astrocytes show elevated GFAP indicative of their state of reactive gliosis?

We thank the reviewer for this comment; indeed, we were wondering if differences in GFAP-activation –as a marker for glial activation – could give us a hint towards differences in the astrocytic subclusters. As shown in the corrected *Supplement Figure 3*, we have analyzed the dynamics of astroglial activation upon 6-OHDA treatment by Immunohistochemistry. Here we detected a maximum of gliosis at around 6 days after the toxin administration. The analysis of our scRNA-seq dataset is lacking samples of the contralateral side or from completely naïve animals. Therefore, a general assessment of the state of gliosis is not possible. In terms of absolute fractions of GFAP+ cells, when analyzing astrocytes and neurons we see, as expected, higher numbers of GFAP+ cells in astrocytes than in neurons (13% versus 0,8%, *see Response Figure 7a*). As also indicated by the expression of GFAP-Cre (*see Supplementary Figure 14b*), there is no bias towards one of the two astrocytic sublusters detectable. Additionally, when plotting GFAP-cre expression of the different clusters, the distribution in GFP+ cells is identical to the total distribution, which suggests, that AAV infection is not biased by the reactive state of the astrocytes (*see Response Figure 7b,c)*. However, the still low percentage of GFAP positive cells in the astrocytic cluster illustrate, that the numbers of reads per cell are not sufficient to detect slight differences in the expression level of GFAP.

**FIGURES FOR REFEREE REPORT NOT SHOWN.**

7. Is spontaneous action potential sufficient to indicate the integration within the existing neuronal network?

This point is well taken. Indeed, the action potentials alone are not a final prove for the functional integration - it would require additional experiments to prove this. We aimed to demonstrate that reprogrammed astrocytes exhibit hallmark components for electrical signal processing and propagation. First, the presence of presynaptic currents show that these cells are able to sense synaptic activity, thus being capable of decode electrical signals from neurotransmission. Second, the injection of positive currents was able to trigger action potentials, clearly showing the presence of functional voltage-gated sodium channels and the ability to propagate electrical signals. Our results support that reprogrammed astrocytes are able to be integrated into the neural circuitry. As also state in our answers to question #9 of referee #1, we will perform more advanced electrophysiological recordings in a follow up study.

8. Most converted neurons are GABAergic, not DAergic. Moreover, ALNe-218 was previously shown to be more efficient than ALN in inducing DA neurons in striatum. The opposite was observed here. Need some discussion.

We expanded the discussion in this regard in the revised version of the manuscript (*see line 359ff*). For more details, please also refer to our answers to question #10 of referee #1. The distinct differences between ALNe218 and ALN is an interesting additional point raised by the referee. As we could show in *Figure 3e*, the induction of the additional factor *NeuroD1* was extremely efficient. As we have also verified the correct expression of

microRNA218 (see *Response Figure 7*), we would exclude a basic technical problem. However, we cannot exclude differences in the dynamic and the absolute level of gene expression between CRISPRa and heterologous promoter mediated ectopic induced expression. Interestingly, the absolute reprogramming efficacy was, albeit lower, still comparable to the ALN condition. However, in the electrophysiological analysis, all of the analyzed ALNe-218 cells did exhibit properties of immature neurons. Based on this, we concluded, that the ALNe-218 reprogramming in comparison to the ALN factor combination is less efficient because of a putative slower or incomplete reprogramming process. If this is caused by the alternative reprogramming method using CRISPRa is likely. However, the underlying molecular mechanisms remain elusive.

*Other points:*

1. FRT recombination sites are diagrammed in Fig. 1a, but not described in the text.

Since the optional possibility of reducing the dCAM mouse to a pure dCAS-VPR system is not used in the present manuscript, we did not focus on this aspect. But of course, for the description of the dCAM mouse as a general tool, it is necessary to mention this aspect. Therefore, we included this sentence to the corresponding paragraph of the results part: "Optionally, if a lower level of gene induction is required, the FRT-flanked SAM components can be removed by flippase induced recombination, converting the dual dCAM activator mouse into a pure dCas-VPR line (*Supplementary Figure 1b*)."

2. Fig. S1b: missing qPCR analysis of miR-218.

Indeed, in *supplementary figure 2c* we focused on the validation of the CRISPRa gene activation showing for ALNe218 the successful induction of *Ascl1*, *Lmx1a* and *NeuroD1,* and the miRNA218 overexpression was described by Rivetti di Val Cervo et al., 2017. Nevertheless, we tested the functionality of the overexpression cassette in Neuro2A cells (see *Response Figure 8*). Since we had to use RNA purification methods optimized for short fragments, it was not possible to conduct this analysis in the same setting as for the validation of *Ascl1*, *Lmx1a* and *NeuroD1* induction.

**FIGURES FOR REFEREE REPORT NOT SHOWN.**

3. The legend in Fig. S2 does not match with the data (missing legend for b; current legend for b appears to be c).

Thank you for noticing this discrepancy. The *Supplementary Figure 2* has been adjusted in this regard.

4. Fig. S3 is a duplicate of main Fig. 3. The missing of the real data in Fig. S3 makes it impossible to verify various statements made in the text.

We are sorry for this mistake. In the submitted version of the manuscript, *Figure 3* instead of *Supplementary Figure 3* was included. The figure has been exchanged.

5. The legend of Fig. 6 indicates the panel a is for ALN and b for ALNe-218, while the data displayed are opposite.

Thank you for noticing this discrepancy. Instead of correcting the legend we adjusted the figure to keep the sequence "GFP-control, ALNe218, ALN" throughout the manuscript.

6. The numbers in Fig. 5c are confusing, difficult to match the numbers stated in the text. For example, it was stated that 11 out of 17 astrocytes were GFP+ and Ascl1+ in the astrocyte cluster. However, in the second row, right, it only labeled 66 (blue)/17 (green) in the middle. It would be helpful to indicate the numbers separately in the astrocyte and neuron clusters.

We apologize for the confusion. Indeed the corresponding text passage is misleading. We modified the results part to "The amount of GFP+/Ascl1+ cells is markedly increased from 3 double positive cells in the GFP control to 17 cells in the *ALN* condition. Interestingly, 11 out of the 17 GFP+/Ascl1+ cells are found in one of the astrocytic subclusters." and hope this is more accurate and easier to follow. Furthermore, we changed the figure following the reviewers suggestion. Now the numbers of GFP positive cells (red), marker gene positive cells (blue) and double positive cells (yellow) are indicated for the astrocytic and neuronal clusters separately.

7. For the benefits of future readers, it would be helpful to describe how the expression of Ascl1 and Mytl1 suggests a transition from astrocytes to neurons.

We did not intend to suggest a transition from astrocytes to neurons based on the expression of Ascl1 and Myt1l. The unbiased clustering is based on a variety of factors. When we manually checked for candidate genes, the clear separation of the expressions domains of Ascl1 and Myt1l was so obvious, that we used these prominent transcription factors involved in direct reprogramming (Vierbuchen et al., 2010) to specify the subclusters. To better illustrate this rationale, we added on sentence to the results part of the manuscript "Interestingly, the two neuronal subclusters are characterized amongst other genes, by high Ascl1 or Myt1l marker expression (Figure 5c)," (*see line 243f*)

8. The information of lentiviruses used to deliver dCAS system in vitro is missing. Which vector and promoter were employed? Are they Cre dependent? Why there is expression of dsRed (Fig. 3c, left panel) in the lenti-based dCAS system?

To address this point, we added the detailed description of the lentiviral vectors to the corresponding methods section. The figure legend was expanded by "(…) using CRISPRa. Astrocytes are infected with two lentiviruses expressing dCas9-VPR in a intein-split version similar to the AAV-dCAS system but driven by a Tet-O promoter. Tet-O driven dsRed and Ascl1 cDNA expressing construct were used as negative and positive controls respectively. (…)". As mentioned in in the response to question number 2 of reviewer #1, we have chosen Tet-O for comparison since it facilitates robust expression after lentiviral transduction.

**Referee #3**

Figure 1a.

- What is the polyA signal used? What is SAM-P2A and does it carry a stop codon?

- For B6.Cg-Tg(Gfap-cre) mouse, is this a knockin? Or is it a transgenic mouse with a GFAP promoter fragment? How big is the promoter fragment?

- For AAV (rAAV2/5): is there a polyA signal for GFP? Is there WPRE?

We used three different polyA signals in the various constructs used in this study: for the GFP reporter a SV40 polyA was used. For miR218 expressing constructs a bGH polyA was used. For the Cas9 and SAM expressing constructs, a short synthetic polyA was utilized. To give a comprehensive overview over the viral constructs and virus combinations used, we included the new *Supplement Figure 17*. Here, further information, e.g. about the position of (Pol-III-) promoters, WPRE and other elements, are depicted in detail. Moreover, we have included the complete sequences of all utilized constructs as *Supplementary Materials*.

Indeed, the B6.Cg-Tg(Gfap-cre) mouse is a transgenic model as described in Gregorian et al., 2009. For the generation of the line, a 15 kb promoter cassette containing the full-length sequence of the murine GFAP gene has been used. The Cre recombinase is inserted into the first exon of the expression cassette which contains all the introns, promoter regulatory elements, exons, and 2.5 kb of 5' and 2 kb of 3' flanking DNA of the astroglia-specific mouse glial fibrillary acidic protein gene. We added the original publication of this mouse line and the respective Jackson Laboratory line name to the results part of the manuscript: "(…) mice were crossed with an astrocyte-specific Cre (*Gfap-Cre,* B6.Cg-Tg(Gfap-cre)77.6Mvs/2J) transgenic mouse line (…)".

Figure 1b.

- Which promoter is used to drive which guide RNA?

- If there are six promoters, are these data from two different vectors each carrying three guide RNAs? What are encoded by the other three pol3 promoters?

This point is well taken. We presented the utilized viral vectors in the main figures and we included now the new *Supplement Figure 17* giving you detailed information and a complete overview over the virus combinations used. We hope that this supplementary figure helps to keep track about the vectors used for the different experiments.

Figure 2b.

- Why is the GFP control % only around 90%? Is there 10% of non-GFAP-specific expression of Are?

Figure 2c.

- Similar to question for 2b, why is there GFP fluorescence in NeuN+ cells in the control condition?

- Given the leakiness of targeting to GFAP+ cells, I suggest the authors conduct an in vitro experiment with cultured astrocyte cells and provide the efficiency of neuronal conversion in that setting.

Indeed, as indicated by the quantification of $GFP^+/NeuN^+$ double positive cells in the GFP control condition, around 5% of GFP-positive cells are expressing the neuronal marker NeuN as well. Interestingly, the identical rate of background expression has been described for cortical AAV injections in a previous study using the same GFAP-cre line (Mattugini et al., 2019). Another source of variation is the fluctuation of endogenous GFAP expression in

adult astrocytes. Moreover, the single cell transcriptomic analysis showed minor expression of GFP in the oligodendrocyte as well as in the monocyte (microglia) cluster, albeit the ratio of the detected cells might be overrepresented due to their compact size compared to astrocytes and neurons.

Following the reviewer's suggestion, we performed in vitro experiments to assess the efficacy and identity of the obtained neurons. For the results, please refer to *Response Figure 2 and Response Figure 4*. As shown in *Response Figure 2b*, the overall ratio of neuronal conversion is in the same range as observed *in vivo*. Based on this, we are convinced that the observed minor leakiness is based on brief transient activation of the GFAP promoter in a limited subpopulation of neurons and does not compromise the experimental outcomes.

Figure 4:

- What is the co-transduction efficiency for the same cell when using multiple vectors?

To address this point we extensively performed immunohistochemical stainings of AAV-dCas animals 13 weeks after injection. However, due to technical problems, the quality of the double and triple stainings for N-Cas9, d-Cas9 and SAM constructs was never sufficient to perform a reliable quantification. Nevertheless, data from our own previous published paper suggest that co-transfection efficacy can be up to 90% using split-intein Cas9 (Moretti et al., 2020). Although the respective model and approach was different, the utilized AAV plasmids are comparable in regard to their main features (backbone, promoter, intein split-Cas9). As the reviewers question correctly implies, a significant amount of co-infection is necessary to obtain successful reprogramming in case of the AAV-dCAS system. In contrast to that, in the ALN condition of the dCAM system, only a single vector for the delivery of gRNAs is necessary (see also the new *Supplement Figure 17*). Since the rate of observed conversion to neurons is not lower – in fact the ratio of $GFP^+$/$NeuN^+$ double positive neurons is even rather higher in the AAV-dCAS setting – we do not consider co-infection as a limiting factor. Furthermore, our *in vitro* experiments based on plasmid co-transfection (*Response Figure 1*) shows reprogramming at a similar level as observed *in vivo*.

- Can the authors provide larger field of view images to give a sense of how widely reprogrammed cells are distributed?

To give an overview over the regional distribution as well as the delimitation of the quantification field, the new *Supplement Figure 4b* is now included.

Figure 7:

- How many new neurons are generated in the brains of the mice undergoing these behavioral tests?

In the dCAM ALN setting, we infected 993.3±106.6 cells and obtained 177±5 NeuN positive neurons. The total amounts of cells is not high. Nevertheless, as detected in the behavioral analysis, these newly generated neurons are capable of modulating the imbalanced striatal circuits in a beneficial way. It is a fascinating observation, that this newly generated neurons can cause a significant amelioration of 6-OHDA induced behavioral changes. Nevertheless, in comparison to human transplantation approaches (for review see e.g. Björklund and Parmar, 2020) these numbers are still reasonable if considering the differences in size and neuron numbers (Herculano-Houzel, et al. 2006).

Overall comments:

- The behavioral rescue seems modest. Can the authors comment on how they envision this strategy, or derivatives, being applied at a high enough efficiency in a therapeutic context in patients?

Without a doubt, the presented system needs to be further modified and improved for therapeutic application. The observed neuronal reprogramming alleviates motor behavior defects but does not fully rescue all behavioral aspects. For this, we modified the introduction toning down the therapeutic aspect (*see line 71ff*). Nevertheless, we are convinced that the GABAergic component in PD pathogenesis is still underestimated and resembles a promising target for future therapeutic strategies. As we have indicated in the discussion, GABAergic reprogramming might be a complementary approach in combination with dopamine replacement. A long this line, we need to understand in the future better the conditions to induce specific neuronal subtypes in the appropriate numbers, in specific brain regions to replace the lost neurons. In addition, we need to understand their integration into existing neuronal circuitries to rescue complex behaviors. Nevertheless, we are convinced, that CRISPRa approaches, although they still require optimization, are a versatile, modular tool for cell reprogramming and replacement therapies in future.

Some additional aspects have also been discussed in our answers to question #10 of referee #1.

**Additional References**


Björklund, A., & Parmar, M. (2020). Neuronal Replacement as a Tool for Basal Ganglia Circuitry Repair: 40 Years in Perspective. Frontiers in cellular neuroscience, 14, 146. https://doi.org/10.3389/fncel.2020.00146

Herculano-Houzel, S., Mota, B. & Lent, R. (2006). Cellular scaling rules for rodent brains. Proc. Natl Acad. Sci. USA 103, 12138–12143.

Kim EJ, Ables JL, Dickel LK, Eisch AJ, Johnson JE. Ascl1 (Mash1) defines cells with long-term neurogenic potential in subgranular and subventricular zones in adult mouse brain (2011). PLoS One. 6(3):e18472.

Lee, Youngjin; Messing, Albee; Su, Mu; Brenner, Michael (2008): GFAP promoter elements required for region-specific and astrocyte-specific expression. Glia 56 (5), S. 481–493.

Liu, Y., Miao, Q., Yuan, J., Han, S., Zhang, P., Li, S., Rao, Z., Zhao, W., Ye, Q., Geng, J., Zhang, X., & Cheng, L. (2015). Ascl1 Converts Dorsal Midbrain Astrocytes into Functional Neurons In Vivo. J Neurosci., 35(25), 9336–9355.

Mattugini, N., Bocchi, R., Scheuss, V., Russo, G. L., Torper, O., Lao, C. L., & Götz, M. (2019). Inducing Different Neuronal Subtypes from Astrocytes in the Injured Mouse Cerebral Cortex. Neuron, 103(6), 1086–1095.e5.

*Rivetti di Val Cervo, P., et al., 2017: Induction of functional dopamine neurons from human astrocytes in vitro and mouse astrocytes in a Parkinson's disease model. Nat Biotechnol. 35, 444-452*.

Theodorou M, Rauser B, Zhang J, Prakash N, Wurst W, Schick JA (2015). Limitations of In Vivo Reprogramming to Dopaminergic Neurons via a Tricistronic Strategy. Hum Gene Ther Methods. 26(4):107-22.

Tiklová K, Nolbrant S, Fiorenzano A, Björklund ÅK, Sharma Y, Heuer A, Gillberg L, Hoban DB, Cardoso T, Adler AF, Birtele M, Lundén-Miguel H, Volakakis N, Kirkeby A, Perlmann T, Parmar M. Single cell transcriptomics identifies stem cell-derived graft composition in a model of Parkinson's disease. Nat Commun. 2020 May 15;11(1):2434.

Zhang, R. L., Chopp, M., Roberts, C., Jia, L., Wei, M., Lu, M., Wang, X., Pourabdollah, S., & Zhang, Z. G. (2011). Ascl1 lineage cells contribute to ischemia-induced neurogenesis and oligodendrogenesis. Journal of cerebral blood flow and metabolism: official journal of the International Society of Cerebral Blood Flow and Metabolism, 31(2), 614–625

4th Feb 2022

Dear Prof. Wurst,

Thank you for the submission of your revised manuscript to EMBO Molecular Medicine. We have now received the enclosed report from the referee who agreed to re-assess it. Since Referees #1 and #3 from the previous round of review were not available this time, we were obliged to seek advice from a new expert in the field and specifically asked them to evaluate whether the concerns raised by Referees #1 and #3 have been addressed appropriately. From the comments below, you will see that this expert supported the publication. While Referee #2 (who reviewed this manuscript before) was overall satisfied with the revisions, they still raised several issues. Given the overall positive comments from the referee and the expert advisor, I am pleased to inform you that we will be able to accept your manuscript pending the following amendments:

1. Please address the remaining concerns of Referee #2. In particular,
- Comment #1 regarding the comparison of CRISPRa and cDNA overexpression in cultured astrocytes: if you have such data at hand, we would encourage you to include them in the manuscript. Otherwise, please discuss this in writing.
- Comment #2 regarding the high viral titers needs to be carefully discussed in light of the Mol Ther paper mentioned by this Referee.

On a more editorial level:

I look forward to reading a new revised version of your manuscript as soon as possible.

Kind regards,
Jingyi

Jingyi Hou
Editor
EMBO Molecular Medicine

*** Instructions to submit your revised manuscript ***

*** PLEASE NOTE *** As part of the EMBO Publications transparent editorial process initiative (see our Editorial at https://www.embopress.org/doi/pdf/10.1002/emmm.201000094), EMBO Molecular Medicine will publish online a Review Process File to accompany accepted manuscripts.

In the event of acceptance, this file will be published in conjunction with your paper and will include the anonymous referee reports, your point-by-point response and all pertinent correspondence relating to the manuscript. If you do NOT want this file to be published, please inform the editorial office at contact@embomolmed.org.

To submit your manuscript, please follow this link:

https://embomolmed.msubmit.net/cgi-bin/main.plex

When submitting your revised manuscript, please include:

1) a .docx formatted version of the manuscript text (including Figure legends and tables)

2) Separate figure files*

3) supplemental information as Expanded View and/or Appendix. Please carefully check the authors guidelines for formatting Expanded view and Appendix figures and tables at
https://www.embopress.org/page/journal/17574684/authorguide#expandedview

4) a letter INCLUDING the reviewer's reports and your detailed responses to their comments (as Word file).

5) The paper explained: EMBO Molecular Medicine articles are accompanied by a summary of the articles to emphasize the major findings in the paper and their medical implications for the non-specialist reader. Please provide a draft summary of your article highlighting
- the medical issue you are addressing,
- the results obtained and
- their clinical impact.
This may be edited to ensure that readers understand the significance and context of the research.
Please refer to any of our published articles for an example.

6) For more information: There is space at the end of each article to list relevant web links for further consultation by our readers. Could you identify some relevant ones and provide such information as well? Some examples are patient associations, relevant databases, OMIM/proteins/genes links, author's websites, etc...

7) Author contributions: the contribution of every author must be detailed in a separate section.

8) EMBO Molecular Medicine now requires a complete author checklist (https://www.embopress.org/page/journal/17574684/authorguide) to be submitted with all revised manuscripts. Please use the checklist as guideline for the sort of information we need WITHIN the manuscript. The checklist should only be filled with page numbers were the information can be found. This is particularly important for animal reporting, antibody dilutions (missing) and exact values and n that should be indicted instead of a range.

9) Every published paper now includes a 'Synopsis' to further enhance discoverability. Synopses are displayed on the journal webpage and are freely accessible to all readers. They include a short stand first (maximum of 300 characters, including space) as well as 2-5 one sentence bullet points that summarise the paper. Please write the bullet points to summarise the key NEW findings. They should be designed to be complementary to the abstract - i.e. not repeat the same text. We encourage inclusion of key acronyms and quantitative information (maximum of 30 words / bullet point). Please use the passive voice. Please attach these in a separate file or send them by email, we will incorporate them accordingly.

You are also welcome to suggest a striking image or visual abstract to illustrate your article. If you do please provide a jpeg file 550 px-wide x 400-px high.

10) A Conflict of Interest statement should be provided in the main text

11) Please note that we now mandate that all corresponding authors list an ORCID digital identifier. This takes <90 seconds to complete. We encourage all authors to supply an ORCID identifier, which will be linked to their name for unambiguous name identification.

Currently, our records indicate that there is no ORCID associated with your account.

Please click the link below to provide an ORCID:
Link Not Available

12) The system will prompt you to fill in your funding and payment information. This will allow Wiley to send you a quote for the article processing charge (APC) in case of acceptance. This quote takes into account any reduction or fee waivers that you may be eligible for. Authors do not need to pay any fees before their manuscript is accepted and transferred to our publisher.

*Additional important information regarding Figures

Each figure should be given in a separate file and should have the following resolution:
Graphs 800-1,200 DPI
Photos 400-800 DPI
Colour (only CMYK) 300-400 DPI"

Figures are not edited by the production team. All lettering should be the same size and style; figure panels should be indicated by capital letters (A, B, C etc). Gridlines are not allowed except for log plots. Figures should be numbered in the order of their appearance in the text with Arabic numerals. Each Figure must have a separate legend and a caption is needed for each panel.

*Additional important information regarding figures and illustrations can be found at https://bit.ly/EMBOPressFigurePreparationGuideline. See also figure legend preparation guidelines: https://www.embopress.org/page/journal/17574684/authorguide#figureformat

The system will prompt you to fill in your funding and payment information. This will allow Wiley to send you a quote for the article processing charge (APC) in case of acceptance. This quote takes into account any reduction or fee waivers that you may

be eligible for. Authors do not need to pay any fees before their manuscript is accepted and transferred to our publisher.

***** Reviewer's comments *****

Referee #2 (Remarks for Author):

I have carefully gone through the revision. In general, the authors have adequately addressed most questions raised and substantially improved the manuscript to include more clear description of results and more balanced discussion on their findings in conjunction with the existing literature. Here, I spot a few remaining issues for the authors to consider in the phase of editorial revision/editing:

Related to my original question 2: One of the most surprising observations is the conversion of astrocytes to GABAergic, rather than anticipated DAergic neurons. The authors speculated that this might be due to insufficient overexpression of TFs by the CRISPRa system used compared to direct cDNA overexpression, but there is no direct evidence to support this speculation. This can be easily demonstrated by comparing ALN CRISPRa vs cDNA overexpression in cultured astrocytes.

In the rebuttal, the authors indicated that they used extremely high titer viruses (10e14 to 10e15 gc/ml) to drive neuronal reprogramming. Do they worry about potential toxic effects, which may selectively kill astrocytes by the reprogramming vector, but not by the control vector, thus leaving leaky neurons modestly expressing GFP mistakenly considered as converted ones? This has been an issue under active debate (see the Letter to the Editor recently published in Mol Ther, https://doi.org/10.1016/j.ymthe.2021.10.017).

Lastly, it may be relevant for the authors to comment on PTB depletion-induced DA neurons in striatum reported by Zhou et al (Cell, https://doi.org/10.1016/j.cell.2020.03.024), which is also distinct from GABAergic neurons seen in the current study. In this regard, Wang et al. (Cell, https://doi.org/10.1016/j.cell.2021.09.005) recently suggested that the observed DA neurons likely result from the toxic effect of the CRISPR-CasRx system used in the Zhou et al study.

------------
Comment from the expert advisor:

I read through the manuscript and the rebuttle and reviewer 2's comments and believe this MS is ready for publication in EMBOM.

I am not so concerned about the inevitable leakiness of the promoter in this case as the differences between the control GFP neurons and ALN as well as the transgene approach; in addition to the selective behavior effects.

I think this is a very useful paper including that fact that they are clear about not seeing conversion to DA neurons but rather Gaba neurons. I amazed that increasing Gaba neurons changes behavior.

**Point by point answers:**

**Referee #2**

I have carefully gone through the revision. In general, the authors have adequately addressed most questions raised and substantially improved the manuscript to include more clear description of results and more balanced discussion on their findings in conjunction with the existing literature. Here, I spot a few remaining issues for the authors to consider in the phase of editorial revision/editing:

Related to my original question 2: One of the most surprising observations is the conversion of astrocytes to GABAergic, rather than anticipated DAergic neurons. The authors speculated that this might be due to insufficient overexpression of TFs by the CRISPRa system used compared to direct cDNA overexpression, but there is no direct evidence to support this speculation. This can be easily demonstrated by comparing ALN CRISPRa vs cDNA overexpression in cultured astrocytes.

As the referee correctly pointed out, one of the most surprising observation was to generate GABAergic neurons although the initial aim to use the ALN factor combination was to generate dopaminergic neurons. Previously, we and others have shown that cDNA overexpression of ALN indeed is capable to reprogram primary cells into dopaminergic neurons (Addis et al, 2011; Theodorou et al, 2015). Therefore, we do not question the ability of the ALN factor combination to generate dopaminergic neurons. Following the reviewers suggestion, we performed CRISPRa induction of ALN in primary astrocytes and obtained GABAergic neurons as well – similar to our in vivo results. Since this is an interesting observation we have now included this datapoint as the new Appendix Figure S18 into the manuscript. Furthermore we extended the corresponding paragraph of the discussion as follows: "Interestingly, when performing in vitro reprogramming of primary murine astrocytes with the dCAS combination ALN, again virtually all induced neurons show GABAergic identity (Appendix Figure S18). Since the induction obtained by CRISPRa is lower compared to cDNA overexpression, it is possible that the unexpected GABAergic identity of the induced neurons is indeed based on the physiological, albeit lower levels of factor expression. On the other side, also the in vivo targeting of NG2 glia with a cDNA-based ALN expression system has been shown to generate GABAergic instead of dopaminergic neurons in the striatum (Pereira et al., 2017; Torper et al., 2015) indicating a strong influence of the specific region and the identity of the targeted glial cells." (see page 13, line 416ff).

In the rebuttal, the authors indicated that they used extremely high titer viruses (10e14 to 10e15 gc/ml) to drive neuronal reprogramming. Do they worry about potential toxic effects, which may selectively kill astrocytes by the reprogramming vector, but not by the control vector, thus leaving leaky neurons modestly expressing GFP mistakenly considered as converted ones? This has been an issue under active debate (see the Letter to the Editor recently published in Mol Ther, https://doi.org/10.1016/j.ymthe.2021.10.017).

This point is well taken. A possible toxic effect of high titer AAV virus should be considered. For this we expanded the corresponding part in the manuscript and discussed several aspects of the ongoing debate: "In light of the ongoing debate about direct reprogramming and the use of AAV virus (Chen, 2021; Wang et al, 2021; Wang & Zhang, 2022), we verified the FLEx system which is used in this study to control not only the GFP expression but in the AAV-dCAS system also the dCas9 expression. Injection into Gfap-Cre negative animals did not show leakiness of the reporter (Appendix Figure S19). Hence, unspecific promoter activation, reporter or reprogramming factor expression is unlikely to occur in our system, which is, in contrast to other approaches, not based on AAV delivery of transcription factor cDNA but on the activation of endogenous genes. Effects of high viral titer application is unlikely since in the dCAM setting, both the GFP-control and ALN animals received the identical amount of AAV. Batch differences of the AAV can be excluded as well, since in the dCAS setting both in GFP-control and ALN

condition, the identical reporter AAV has been used (Appendix Figure S17). In addition, aberrant activation of the GFAP-Cre promoter could not be observed in the single-cell RNA sequencing analysis. Moreover, with both induction systems, we obtained similar results albeit the experimental set up differed substantially in regard to dCas9 delivery (lox-stop-lox dCas9 transgene versus AAV FLEx dCas9)."(page 12, line 380ff). Along this line we included a new Appendix Figure S17 demonstrating the absence of leakiness of our reporter construct.

Furthermore, we did not detect any hints of a selective toxicity of our reprogramming vectors: especially in the dCAM setting, the GFP-control and ALN vector differ only in the absence or presence of pol-III driven gRNAs. Given the absence of leakiness both for dCas9 and the GFP reporter, selective toxicity would therefore solely be driven by gRNA expression. Nevertheless: since the total amount of GFP positive cells do not significantly differ between the conditions (see Appendix Figure S4), we do not assume selective toxicity in our systems.

Lastly, it may be relevant for the authors to comment on PTB depletion-induced DA neurons in striatum reported by Zhou et al (Cell, https://doi.org/10.1016/j.cell.2020.03.024), which is also distinct from GABAergic neurons seen in the current study. In this regard, Wang et al. (Cell, https://doi.org/10.1016/j.cell.2021.09.005) recently suggested that the observed DA neurons likely result from the toxic effect of the CRISPR-CasRx system used in the Zhou et al study.

Thank you for this comment. Indeed the work published by Zhou et al. published 2020 in Cell is based on the knockdown of Ptbp1 by CasRx also known as Cas13d and might result in unwanted toxic effects. However, the RNA targeting Cas system CasRx/Cas13d is substantially different from the CRISPRa approach utilized in our study, we decided not to discuss this in detail in our manuscript. Cas13d is active on the transcriptome level by selectively degrading target mRNA. On the one side, toxic effects could be triggered by excessive knockdown of the Ptbp1 target mRNA itself. On the other side, in contrast to initial reports which reported a high degree of specificity in eukaryotic cells, indications arise that collateral activity of the CasRx/Cas13d system, a phenomenon described for prokaryots, can also be observed in mammalian cells (e.g. Kelley, et al.; BioRxiv, doi: https://doi.org/10.1101/2021.12.20.473384). In addition to the on target effect, this could potentially lead to unwanted side effects. Nevertheless, since these open questions a far from the methodology of our manuscript, we decided not to address this topic.

10th Mar 2022

Dear Wolfgang,

We are pleased to inform you that your manuscript is accepted for publication and is now being sent to our publisher to be included in the next available issue of EMBO Molecular Medicine.

We would like to remind you that as part of the EMBO Publications transparent editorial process initiative, EMBO Molecular Medicine will publish a Review Process File online to accompany accepted manuscripts. If you do NOT want the file to be published or would like to exclude figures, please immediately inform the editorial office via e-mail.

Please read below for additional IMPORTANT information regarding your article, its publication and the production process.

Congratulations on this exicting work!

Best wishes,
Jingyi

Jingyi Hou
Editor
EMBO Molecular Medicine

Follow us on Twitter @EmboMolMed
Sign up for eTOCs at embopress.org/alertsfeeds

*** *** *** IMPORTANT INFORMATION *** *** ***

SPEED OF PUBLICATION
The journal aims for rapid publication of papers, using using the advance online publication "Early View" to expedite the process: A properly copy-edited and formatted version will be published as "Early View" after the proofs have been corrected. Please help the Editors and publisher avoid delays by providing e-mail address(es), telephone and fax numbers at which author(s) can be contacted.

Should you be planning a Press Release on your article, please get in contact with embomolmed@wiley.com as early as possible, in order to coordinate publication and release dates.

LICENSE AND PAYMENT:

All articles published in EMBO Molecular Medicine are fully open access: immediately and freely available to read, download and share.

EMBO Molecular Medicine charges an article processing charge (APC) to cover the publication costs. You, as the corresponding author for this manuscript, should have already received a quote with the article processing fee separately. Please let us know in case this quote has not been received.

Once your article is at Wiley for editorial production you will receive an email from Wiley's Author Services system, which will ask you to log in and will present you with the publication license form for completion. Within the same system the publication fee can be paid by credit card, an invoice, pro forma invoice or purchase order can be requested.

Payment of the publication charge and the signed Open Access Agreement form must be received before the article can be published online.

PROOFS

You will receive the proofs by e-mail approximately 2 weeks after all relevant files have been sent o our Production Office. Please return them within 48 hours and if there should be any problems, please contact the production office at

embopressproduction@wiley.com.

Please inform us if there is likely to be any difficulty in reaching you at the above address at that time. Failure to meet our deadlines may result in a delay of publication.

All further communications concerning your paper proofs should quote reference number EMM-2021-14797-V3 and be directed to the production office at embopressproduction@wiley.com.

Thank you,

Jingyi Hou
Editor
EMBO Molecular Medicine

EMBO PRESS

YOU MUST COMPLETE ALL CELLS WITH A PINK BACKGROUND

PLEASE NOTE THAT THIS CHECKLIST WILL BE PUBLISHED ALONGSIDE YOUR PAPER

Corresponding Author Name: Wolfgang Wurst
Journal Submitted to: EMBO Molecular Medicine
Manuscript Number: EMM-2021-14797-V2

## Reporting Checklist For Life Sciences Articles (Rev. June 2017)

This checklist is used to ensure good reporting standards and to improve the reproducibility of published results. These guidelines are consistent with the Principles and Guidelines for Reporting Preclinical Research issued by the NIH in 2014. Please follow the journal's authorship guidelines in preparing your manuscript.

### A- Figures
### 1. Data
### The data shown in figures should satisfy the following conditions:

- the data were obtained and processed according to the field's best practice and are presented to reflect the results of the experiments in an accurate and unbiased manner.
- figure panels include only data points, measurements or observations that can be compared to each other in a scientifically meaningful way.
- graphs include clearly labeled error bars for independent experiments and sample sizes. Unless justified, error bars should not be shown for technical replicates.
- if n< 5, the individual data points from each experiment should be plotted and any statistical test employed should be justified
- Source Data should be included to report the data underlying graphs. Please follow the guidelines set out in the author ship guidelines on Data Presentation.

### 2. Captions
### Each figure caption should contain the following information, for each panel where they are relevant:

- a specification of the experimental system investigated (eg cell line, species name).
- the assay(s) and method(s) used to carry out the reported observations and measurements
- an explicit mention of the biological and chemical entity(ies) that are being measured.
- an explicit mention of the biological and chemical entity(ies) that are altered/varied/perturbed in a controlled manner.

- the exact sample size (n) for each experimental group/condition, given as a number, not a range;
- a description of the sample collection allowing the reader to understand whether the samples represent technical or biological replicates (including how many animals, litters, cultures, etc.).
- a statement of how many times the experiment shown was independently replicated in the laboratory.
- definitions of statistical methods and measures:
  - common tests, such as t-test (please specify whether paired vs. unpaired), simple χ2 tests, Wilcoxon and Mann-Whitney tests, can be unambiguously identified by name only, but more complex techniques should be described in the methods section;
  - are tests one-sided or two-sided?
  - are there adjustments for multiple comparisons?
  - exact statistical test results, e.g., P values = x but not P values < x;
  - definition of 'center values' as median or average;
  - definition of error bars as s.d. or s.e.m.

Any descriptions too long for the figure legend should be included in the methods section and/or with the source data.

**In the pink boxes below, please ensure that the answers to the following questions are reported in the manuscript itself. Every question should be answered. If the question is not relevant to your research, please write NA (non applicable). We encourage you to include a specific subsection in the methods section for statistics, reagents, animal models and human subjects.**

USEFUL LINKS FOR COMPLETING THIS FORM

http://www.antibodypedia.com
http://1degreebio.org
http://www.equator-network.org/reporting-guidelines/improving-bioscience-research-repor
http://grants.nih.gov/grants/olaw/olaw.htm
http://www.mrc.ac.uk/Ourresearch/Ethicsresearchguidance/Useofanimals/index.htm
http://ClinicalTrials.gov
http://www.consort-statement.org
http://www.consort-statement.org/checklists/view/32-consort/66-title
http://www.equator-network.org/reporting-guidelines/reporting-recommendations-for-tumo
http://datadryad.org
http://figshare.com
http://www.ncbi.nlm.nih.gov/gap
http://www.ebi.ac.uk/ega
http://biomodels.net/
http://biomodels.net/miriam/
http://jjj.biochem.sun.ac.za
http://oba.od.nih.gov/biosecurity/biosecurity_documents.html
http://www.selectagents.gov/

### B- Statistics and general methods

Please fill out these boxes (Do not worry if you cannot see all your text once you press return)

| | |
|---|---|
| 1.a. How was the sample size chosen to ensure adequate power to detect a pre-specified effect size? | The sample size for the histological as well as the behavioral analysis has been set by an statistical expertise in the course of the animal testing application. |
| 1.b. For animal studies, include a statement about sample size estimate even if no statistical methods were used. | The sample size for the histological as well as the behavioral analysis has been set by an statistical expertise in the course of the animal testing application. |
| 2. Describe inclusion/exclusion criteria if samples or animals were excluded from the analysis. Were the criteria pre-established? | No animals were excluded from the analysis. |
| 3. Were any steps taken to minimize the effects of subjective bias when allocating animals/samples to treatment (e.g. randomization procedure)? If yes, please describe. | tester blinded to genotype and treatment performed all animal studies. Animals were tested in a random order. All histological countings were performed by tester blinded to genotype and treatment |
| For animal studies, include a statement about randomization even if no randomization was used. | Randomization was applied in all animal tests. |
| 4.a. Were any steps taken to minimize the effects of subjective bias during group allocation or/and when assessing results (e.g. blinding of the investigator)? If yes please describe. | tester blinded to genotype and treatment performed all animal studies. Animals were tested in a random order. All histological countings were performed by tester blinded to genotype and treatment |
| 4.b. For animal studies, include a statement about blinding even if no blinding was done | tester blinded to genotype and treatment performed all animal studies |
| 5. For every figure, are statistical tests justified as appropriate? | For every figure including qunatifications and/or statistics, the appropiate test has been utilized as described in figurre legend, main text and materials and method section |
| Do the data meet the assumptions of the tests (e.g., normal distribution)? Describe any methods used to assess it. | The normality of the distribution of data points was verified using Shapiro-Wilk test. Data was analyzed using either an unpaired t-test or a multiple comparison ANOVA, followed by a posthoc Tukey's multiple comparisons test. When normality tests did not indicate normal distribution, non-parametric Kruskall-Wallis test was performed. |
| Is there an estimate of variation within each group of data? | Yes,  by multiple comparison ANOVA, followed by a posthoc Tukey's multiple comparisons test |
| Is the variance similar between the groups that are being statistically compared? | Yes (unpaired t-test, multiple comparison ANOVA) |

### C- Reagents

| | |
|---|---|
| 6. To show that antibodies were profiled for use in the system under study (assay and species), provide a citation, catalog number and/or clone number, supplementary information or reference to an antibody validation profile. e.g., Antibodypedia (see link list at top right), 1DegreeBio (see link list at top right). | Primary antibodies: rabbit-anti-tyrosine hydroxylase 1:500 (Pel-Freeze, P40101, USA), mouse-anti-NeuN 1:1000 (Abcam, ab104224, USA), anti-chicken-GFP 1:1000 (Abcam, ab13970, USA), anti-rabbit GFAP 1:1000 (Abcam,ab7260, USA), anti-mouse-Parvalbumin 1:1000 (Sigma-Aldrich, P3088, USA), anti-rabbit-calretinin 1:1000 (Swant, CR7697, Switzerland), anti-goat-CHAT 1:100 (Merck-Millipore, AB144P, Germany), anti-rabbit-Gad65/67 1:500 (Abcam, ab49832, USA), anti-mouse-vGLUT1 1:1000 (Atlas, AMAb91041, USA), anti-rabbit-DARPP32 1:500 (Abcam, ab40801, USA), anti-rabbit-MAP2 1:500 (Merck-Millipore, ab5622, Germany), anti-rabbit-TUJ1 1:500 (Abcam, ab18207, USA). Secondary antibodies: Donkey anti-mouse IgG Alexa Fluor 594 1:500 (Thermo Fisher Scientific, A-21203, Germany), donkey anti-rabbit IgG Alexa Fluor 594 1:500 (Thermo Fisher Scientific, A-21207, Germany), donkey anti-chicken IgY Alexa Fluor 488 1:250 (Dianova, 703-546-155, Germany). |
| 7. Identify the source of cell lines and report if they were recently authenticated (e.g., by STR profiling) and tested for mycoplasma contamination. | Neuro2A cell line was purchased from ATCC (ATCC, CCL-131, USA). Cells are tested for mycoplasm infection routinously. |

* for all hyperlinks, please see the table at the top right of the document

## D- Animal Models

| | |
|---|---|
| 8. Report species, strain, gender, age of animals and genetic modification status where applicable. Please detail housing and husbandry conditions and the source of animals. | Animal housing and handling protocols were approved by the committee for the Care and Use of Laboratory animals of the Government of Upper Bavaria (Germany) and were carried out in accordance with the European Communities' Council Directive 2010/63/EU. During the work, all efforts were made to minimize animal suffering. All mouse lines were kept in a controlled pathogen free (SPF) hygiene standard environment on a 12 h light/dark cycle. The mice had access to ad libitum standard feed and water always. For the analysis the B6.Cg-Tg(Gfap-cre)77.6Mvs/2J (GFAP-Cre) was purchased from Jackson Laboratories (024098), the line was further bred on a C57BL/6N background. The Rosa26-dCas-activator mouse line (dCAM) was produced on a B6N background. For the analysis littermates of the B6.Cg-Tg(Gfap-cre)77.6Mvs/2J x dCAM/N line was used. When crossing the B6.Cg-Tg(Gfap-cre)77.6Mvs/2J line with transgenic animal carrying *LoxP* cassettes, it was payed attention to only breed female Cre mice, as it is known for this line to have Cre expression in the male germline |
| 9. For experiments involving live vertebrates, include a statement of compliance with ethical regulations and identify the committee(s) approving the experiments. | All tests were approved for the ethical treatment of animals by the Government of Upper Bavaria. |
| 10. We recommend consulting the ARRIVE guidelines (see link list at top right) (PLoS Biol. 8(6), e1000412, 2010) to ensure that other relevant aspects of animal studies are adequately reported. See author guidelines, under 'Reporting | The study is in accordance to the ARRIVE guidelines. |

## E- Human Subjects

| | |
|---|---|
| 11. Identify the committee(s) approving the study protocol. | NA |
| 12. Include a statement confirming that informed consent was obtained from all subjects and that the experiments conformed to the principles set out in the WMA Declaration of Helsinki and the Department of Health and Human Services Belmont Report. | NA |
| 13. For publication of patient photos, include a statement confirming that consent to publish was obtained. | NA |
| 14. Report any restrictions on the availability (and/or on the use) of human data or samples. | NA |
| 15. Report the clinical trial registration number (at ClinicalTrials.gov or equivalent), where applicable. | NA |
| 16. For phase II and III randomized controlled trials, please refer to the CONSORT flow diagram (see link list at top right) and submit the CONSORT checklist (see link list at top right) with your submission. See author guidelines, under 'Reporting Guidelines'. Please confirm you have submitted this list. | NA |
| 17. For tumor marker prognostic studies, we recommend that you follow the REMARK reporting guidelines (see link list at top right). See author guidelines, under 'Reporting Guidelines'. Please confirm you have followed these guidelines. | NA |

## F- Data Accessibility

| | |
|---|---|
| 18: Provide a "Data Availability" section at the end of the Materials & Methods, listing the accession codes for data generated in this study and deposited in a public database (e.g. RNA-Seq data: Gene Expression Omnibus GSE39462, Proteomics data: PRIDE PXD000208 etc.) Please refer to our author guidelines for 'Data Deposition'.<br><br>Data deposition in a public repository is mandatory for:<br>a. Protein, DNA and RNA sequences<br>b. Macromolecular structures<br>[illegible] | DNA Sequences of all applied constructs are included in the manuscript |
| 19. Deposition is strongly recommended for any datasets that are central and integral to the study; please consider the journal's data policy. If no structured public repository exists for a given data type, we encourage the provision of datasets in the manuscript as a Supplementary Document (see author guidelines under 'Expanded View' or in unstructured repositories such as Dryad (see link list at top right) or Figshare (see link list at top right). | **Scripts and instructions for reproducibility of the presented analyses, including input and output of relevant steps are available at http://github.com/theislab/astrocytes_reprogramming_analysis. The scRNA-seq raw and processed sequencing data has been deposited at the Gene Expression Omnibus. The raw sequencing data are available at the Gene Expression Omnibus (GEO) data repository under the accession number GSE149872.** |
| 20. Access to human clinical and genomic datasets should be provided with as few restrictions as possible while respecting ethical obligations to the patients and relevant medical and legal issues. If practically possible and compatible with the individual consent agreement used in the study, such data should be deposited in one of the major public access- | NA |
| 21. Computational models that are central and integral to a study should be shared without restrictions and provided in a machine-readable form. The relevant accession numbers or links should be provided. When possible, standardized format (SBML, CellML) should be used instead of scripts (e.g. MATLAB). Authors are strongly encouraged to follow the MIRIAM guidelines (see link list at top right) and deposit their model in a public database such as Biomodels (see link list at top [illegible] | NA |

## G- Dual use research of concern

| | |
|---|---|
| 22. Could your study fall under dual use research restrictions? Please check biosecurity documents (see link list at top right) and list of select agents and toxins (APHIS/CDC) (see link list at top right). According to our biosecurity guidelines, provide a statement only if it could. | NA |
